# The European guideline on management of major bleeding and coagulopathy following trauma: fifth edition

**DOI:** 10.1186/s13054-019-2347-3

**Published:** 2019-03-27

**Authors:** Donat R. Spahn, Bertil Bouillon, Vladimir Cerny, Jacques Duranteau, Daniela Filipescu, Beverley J. Hunt, Radko Komadina, Marc Maegele, Giuseppe Nardi, Louis Riddez, Charles-Marc Samama, Jean-Louis Vincent, Rolf Rossaint

**Affiliations:** 1 Institute of Anaesthesiology, University of Zurich and University Hospital Zurich, Raemistrasse 100, CH-8091 Zurich, Switzerland; 2 Department of Trauma and Orthopaedic Surgery, Cologne-Merheim Medical Centre (CMMC), University of Witten/Herdecke, Ostmerheimer Strasse 200, D-51109 Cologne, Germany; 3 Department of Anaesthesiology, Perioperative Medicine and Intensive Care, J.E. Purkinje University, Masaryk Hospital, Usti nad Labem, Socialni pece 3316/12A, CZ-40113 Usti nad Labem, Czech Republic; 4 Centre for Research and Development, University Hospital Hradec Kralove, Hradec Kralove, Czech Republic, Sokolska 581, CZ-50005 Hradec Kralove, Czech Republic; 5 Department of Anaesthesiology and Intensive Care Medicine, Faculty of Medicine in Hradec Kralove, Charles University, Simkova 870, CZ-50003 Hradec Kralove, Czech Republic; 6 Department of Anaesthesia, Pain Management and Perioperative Medicine, QE II Health Sciences Centre, Dalhousie University, Halifax, 10 West Victoria, 1276 South Park St, Halifax, NS B3H 2Y9 Canada; 7 Department of Anaesthesia and Intensive Care, Hôpitaux Universitaires Paris Sud, University of Paris XI, Faculté de Médecine Paris-Sud, 78 rue du Général Leclerc, F-94275 Le Kremlin-Bicêtre Cedex, France; 8 Department of Cardiac Anaesthesia and Intensive Care, C. C. Iliescu Emergency Institute of Cardiovascular Diseases, Sos Fundeni 256-258, RO-022328 Bucharest, Romania; 9 King’s College and Departments of Haematology and Pathology, Guy’s and St Thomas’ NHS Foundation Trust, Westminster Bridge Road, London, SE1 7EH UK; 10 Department of Traumatology, General and Teaching Hospital Celje, Medical Faculty Ljubljana University, SI-3000 Celje, Slovenia; 11 Department of Trauma and Orthopaedic Surgery, Cologne-Merheim Medical Centre (CMMC), Institute for Research in Operative Medicine (IFOM), University of Witten/Herdecke, Ostmerheimer Strasse 200, D-51109 Cologne, Germany; 12 Department of Anaesthesia and ICU, AUSL della Romagna, Infermi Hospital Rimini, Viale Settembrini, 2, I-47924 Rimini, Italy; 13 Department of Surgery and Trauma, Karolinska University Hospital, S-171 76 Solna, Sweden; 14 Hotel-Dieu University Hospital, 1, place du Parvis de Notre-Dame, F-75181 Paris Cedex 04, France; 15 Department of Intensive Care, Erasme University Hospital, Université Libre de Bruxelles, Route de Lennik 808, B-1070 Brussels, Belgium; 16 Department of Anaesthesiology, University Hospital Aachen, RWTH Aachen University, Pauwelsstrasse 30, D-52074 Aachen, Germany

**Keywords:** Coagulopathy, Emergency medicine, Haemostasis, Practice guideline, Trauma

## Abstract

**Background:**

Severe traumatic injury continues to present challenges to healthcare systems around the world, and post-traumatic bleeding remains a leading cause of potentially preventable death among injured patients. Now in its fifth edition, this document aims to provide guidance on the management of major bleeding and coagulopathy following traumatic injury and encourages adaptation of the guiding principles described here to individual institutional circumstances and resources.

**Methods:**

The pan-European, multidisciplinary Task Force for Advanced Bleeding Care in Trauma was founded in 2004, and the current author group included representatives of six relevant European professional societies. The group applied a structured, evidence-based consensus approach to address scientific queries that served as the basis for each recommendation and supporting rationale. Expert opinion and current clinical practice were also considered, particularly in areas in which randomised clinical trials have not or cannot be performed. Existing recommendations were re-examined and revised based on scientific evidence that has emerged since the previous edition and observed shifts in clinical practice. New recommendations were formulated to reflect current clinical concerns and areas in which new research data have been generated.

**Results:**

Advances in our understanding of the pathophysiology of post-traumatic coagulopathy have supported improved management strategies, including evidence that early, individualised goal-directed treatment improves the outcome of severely injured patients. The overall organisation of the current guideline has been designed to reflect the clinical decision-making process along the patient pathway in an approximate temporal sequence. Recommendations are grouped behind the rationale for key decision points, which are patient- or problem-oriented rather than related to specific treatment modalities. While these recommendations provide guidance for the diagnosis and treatment of major bleeding and coagulopathy, emerging evidence supports the author group’s belief that the greatest outcome improvement can be achieved through education and the establishment of and adherence to local clinical management algorithms.

**Conclusions:**

A multidisciplinary approach and adherence to evidence-based guidance are key to improving patient outcomes. If incorporated into local practice, these clinical practice guidelines have the potential to ensure a uniform standard of care across Europe and beyond and better outcomes for the severely bleeding trauma patient.

**Electronic supplementary material:**

The online version of this article (10.1186/s13054-019-2347-3) contains supplementary material, which is available to authorized users.

## Key messages

- Traumatically injured patients should be transported quickly and treated by a specialised trauma centre whenever possible.
- Measures to monitor and support coagulation should be initiated as early as possible and used to guide a goal-directed treatment strategy.
- A damage-control approach to surgical intervention should guide patient management.
- Coagulation support and thromboprophylactic strategies should consider trauma patients who have been pre-treated with anticoagulants or platelet inhibitors.
- Local adherence to a multidisciplinary, evidence-based treatment protocol should serve as the basis of patient management and undergo regular quality assessment.

## Background

Severe trauma is a major global public health issue, contributing to about 1 in 10 mortalities and resulting in the annual worldwide death of more than 5.8 million people [1, 2]. According to the World Health Organization (WHO), road traffic accidents, suicides and homicides are the three leading causes of injury and violence-related deaths [3]. In recent years, sudden mass casualties due to bombing and assaults have become an new phenomenon in Europe and other regions, resulting in hundreds of severely injured and bleeding patients within a very short period of time, thereby posing huge challenges for local healthcare systems [4–6].

**Fig. 1:**
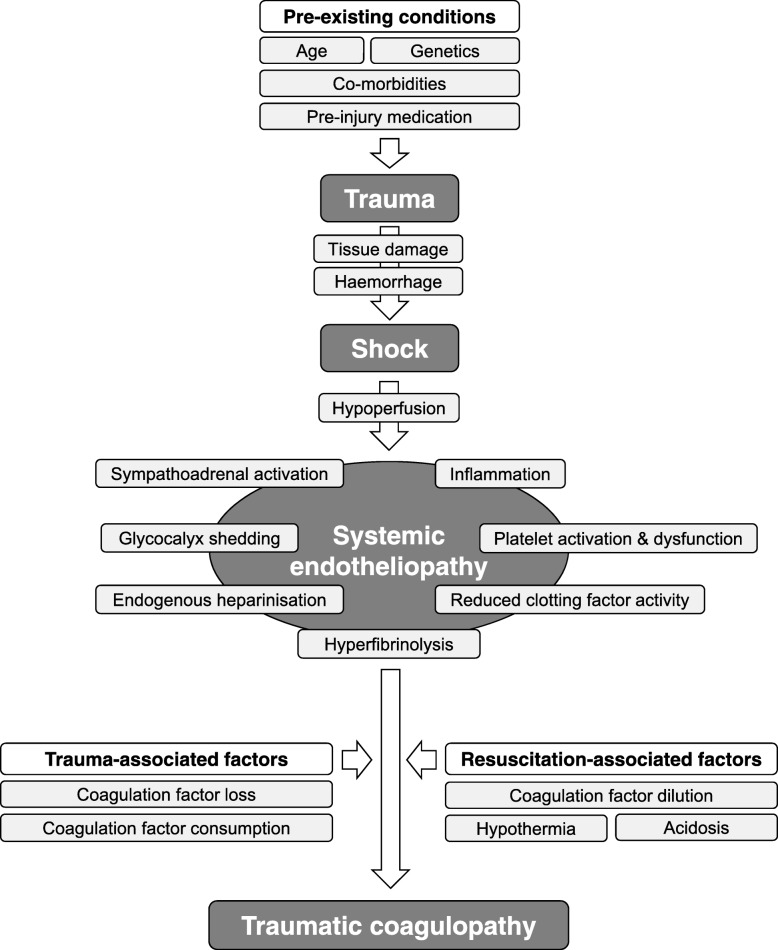
Schematic drawing of the factors, including those that are preexisting as well as those related to both trauma and resuscitation measures, that contribute to traumatic coagulopathy. Adapted from [20, 24, 30–32, 38]

Uncontrolled post-traumatic bleeding is still the leading cause of potentially preventable death among injured patients [7–9] and one third of all bleeding trauma patients show signs of coagulopathy at hospital admission [10–17]. These patients develop multiple organ failure and experience death more frequently than patients with similar injury patterns in the absence of coagulopathy [11, 13, 14, 18, 19]. The early acute coagulopathy associated with traumatic injury has recently been recognised as a multifactorial primary condition that results from a combination of bleeding-induced shock, tissue injury-related thrombomodulin upregulation, thrombin-thrombomodulin-complex generation and the activation of anticoagulant and fibrinolytic pathways (Fig. 1) [8, 10, 13–15, 20–26]. The severity of the coagulation disorder is influenced by environmental and therapeutic factors that result in acidaemia, hypothermia, dilution, hypoperfusion and consumption of coagulation factors [10, 14, 24, 27–32]. Moreover, the coagulopathy is modified by trauma-related factors such as brain injury [33] and individual patient-related factors that include age, genetic background, co-morbidities, inflammation and medication administered prior to becoming injured, especially oral anticoagulants, and pre-hospital fluid administration [28, 34, 35].

This European clinical practice guideline, originally published in 2007 [36] and updated in 2010 [37], 2013 [38] and 2016 [39], represents the fifth edition of the guideline and is part of the European “*STOP the Bleeding Campaign*”, an international initiative launched in 2013 to reduce morbidity and mortality associated with bleeding following traumatic injury [40]. In the last 3 years, a multitude of studies were published that enhance understanding of the pathophysiology of trauma-induced coagulopathy, fill important knowledge gaps about the mechanism and efficacy of trauma treatment strategies and provide evidence that individualised goal-directed trauma treatment improves the outcome of severely injured patients. This new information has been integrated in the current version of the guideline.

Although this set of recommendations outlines corridors for diagnosis and treatment, the author group believes that the greatest outcome improvement can be achieved through education and the establishment of local clinical management guidelines or algorithms. We believe that adherence to local management guidelines or algorithms should be assessed on a regular basis and will lead to greater adherence. If incorporated into local practice, these clinical practice guidelines have the potential to ensure a uniform standard of care across Europe and beyond and better outcomes for the severely bleeding trauma patient, as has indeed be found in three recent studies [41–43].

## Methods

The recommendations made in this guideline are graded according to the Grading of Recommendations Assessment, Development and Evaluation (GRADE) system [44], summarised in Table 1. According to the GRADE scheme, the number associated with each recommendation reflects the strength of the recommendation by the author group, with “we recommend” (Grade 1) being stronger and “we suggest” (Grade 2) being weaker, while the associated letter (A, B or C) reflects the quality of the scientific evidence. Comprehensive, structured, computer-based literature searches were performed using the indexed online database MEDLINE/PubMed, supplemented by screening of reference lists within relevant publications. The aim of each search strategy was to identify randomised controlled trials (RCTs), non-RCTs and systematic reviews that addressed specific scientific queries. In the absence of high-quality scientific support, case reports, observational studies and case control studies were also considered and the literature support for each recommendation graded accordingly.

**Table 1:** Grading of recommendations after [44]. RCT, randomised controlled trial. Table reprinted with permission

Grade of recommendation	Clarity of risk/benefit	Quality of supporting evidence	Implications
1A			
Strong recommendation, high-quality evidence	Benefits clearly outweigh risk and burdens, or vice versa	RCTs without important limitations or overwhelming evidence from observational studies	Strong recommendation, can apply to most patients in most circumstances without reservation
1B			
Strong recommendation, moderate-quality evidence	Benefits clearly outweigh risk and burdens, or vice versa	RCTs with important limitations (inconsistent results, methodological flaws, indirect or imprecise) or exceptionally strong evidence from observational studies	Strong recommendation, can apply to most patients in most circumstances without reservation
1C			
Strong recommendation, low-quality or very low-quality evidence	Benefits clearly outweigh risk and burdens, or vice versa	Observational studies or case series	Strong recommendation but may change when higher quality evidence becomes available
2A			
Weak recommendation, high-quality evidence	Benefits closely balanced with risks and burden	RCTs without important limitations or overwhelming evidence from observational studies	Weak recommendation, best action may differ depending on circumstances or patients’ or societal values
2B			
Weak recommendation, moderate-quality evidence	Benefits closely balanced with risks and burden	RCTs with important limitations (inconsistent results, methodological flaws, indirect or imprecise) or exceptionally strong evidence from observational studies	Weak recommendation, best action may differ depending on circumstances or patients’ or societal values
2C			
Weak recommendation, low-quality or very low-quality evidence	Uncertainty in the estimates of benefits, risks, and burden; benefits, risk and burden may be closely balanced	Observational studies or case series	Very weak recommendation; other alternatives may be equally reasonable

Boolean operators, medical subject headings (MeSH) and key terms were applied to structure each literature search. Searches were limited to a uniform human patient population defined by the search terms and the time period since 01 February 2015. The structured literature search strategies applied to each section of the guideline are listed in Additional file 1. Abstracts identified by each search strategy were screened by a subset of authors and if considered relevant, full publications evaluated. Evaluation of literature chosen for citation in the guideline was performed according to the 2011 Oxford Centre for Evidence-Based Medicine (OCEBM) working group levels of evidence (Table 2) [45]. Each literature citation included in this version of the guideline and the corresponding grading according to the OCEBM levels of evidence (Table 2) are listed in Additional file 2.

**Table 2:** Oxford Centre for Evidence-based Medicine (OCEBM) levels of evidence (2011) [45]

Question	Step 1 (level 1*)	Step 2 (level 2*)	Step 3 (level 3*)	Step 4 (level 4*)	Step 5 (level 5)
How common is the problem?	Local and current random sample surveys (or censuses)	Systematic review of surveys that allow matching to local circumstances**	Local non-random sample**	Case-series**	N/A
Is this diagnostic or monitoring test accurate? (diagnosis)	Systematic review of cross-sectional studies with consistently applied reference standard and blinding	Individual cross-sectional studies with consistently applied reference standard and blinding	Non-consecutive studies or studies without consistently applied reference standards**	Case-control studies or poor or non-independent reference standard**	Mechanism-based reasoning
What will happen if we do not add a therapy? (prognosis)	Systematic review of inception cohort studies	Inception cohort studies	Cohort study or control arm of randomised trial*	Case-series or case-control studies or poor-quality prognostic cohort study**	N/A
Does this intervention help? (treatment benefits)	Systematic review of randomised trials or n-of-1 trials	Randomised trial or observational study with dramatic effect	Non-randomised controlled cohort/follow-up study**	Case-series, case-control studies or historically controlled studies**	Mechanism-based reasoning
What are the common harms? (treatment harms)	Systematic review of randomised trials, systematic review of nested case-control studies, n-of-1 trial with the patient you are raising the question about, or observational study with dramatic effect	Individual randomised trial or (exceptionally) observational study with dramatic effect	Non-randomised controlled cohort/follow-up study (post-marketing surveillance) provided there are sufficient numbers to rule out a common harm. (For long-term harms the duration of follow-up must be sufficient.)**	Case-series, case-control or historically controlled studies**	Mechanism-based reasoning
What are the rare harms? (treatment harms)	Systematic review of randomised trials or n-of-1 trial	Randomised trial or (exceptionally) observational study with dramatic effect			
Is this (early detection) test worthwhile? (screening)	Systematic review of randomised trials	Randomised trial	Non-randomised controlled cohort/follow-up study**	Case-series, case-control or historically controlled studies**	Mechanism-based reasoning

*Level may be graded down on the basis of study quality, imprecision, indirectness [study PICO (patient, problem or population, intervention, comparison, control or comparator, outcome) does not match questions PICO)], because of inconsistency between studies, or because the absolute effect size is very small; level may be graded up if there is a large or very large effect size

**As always, a systematic review is generally better than an individual study

*N/A* not applicable

Selection of the scientific queries addressed, screening and evaluation of the literature, formulation of the recommendations and the supporting rationales was performed by members of the Task Force for Advanced Bleeding Care in Trauma, which was founded in 2004. The Task Force comprises a multidisciplinary team of pan-European experts representing the fields of emergency medicine, surgery, anaesthesiology, haematology and intensive care medicine. Among the authors are representatives of the European Society for Trauma and Emergency Surgery (ESTES), the European Society of Anaesthesiology (ESA), the European Shock Society (ESS), the European Society for Emergency Medicine (EuSEM), the Network for the Advancement of Patient Blood Management, Haemostasis and Thrombosis (NATA) and the European Society of Intensive Care Medicine (ESICM).

The guideline update process involved several remote (telephone and/or internet-based) meetings, extensive electronic communication and one face-to-face consensus conference. In December 2017, the authors participated in a web conference during which the queries to be addressed in the updated guideline were defined. Screening and evaluation of abstracts and full publications identified by the structured searches and formulation of draft recommendations and rationales was performed by working subgroups. Each chapter was reviewed by an assigned working subgroup and then the entire author group. The wording of each recommendation was finalised during a face-to-face consensus conference that took place in April 2018. Following revisions and approval by the author group, the manuscript was approved by the endorsing societies between August and November 2018. An update of this manuscript is anticipated in due time.

## Results

### I. Initial resuscitation and prevention of further bleeding

#### Minimal elapsed time

##### Recommendation 1

We recommend that severely injured patients be transported directly to an appropriate trauma facility. (Grade 1B)

We recommend that the time elapsed between injury and bleeding control be minimised. (Grade 1A)

#### Rationale

Because relatively few hospitals provide all of the services required to treat patients with multiple injuries, many healthcare systems have developed trauma networks or processes. The underlying aim of trauma care organisation is to move patients to multi-specialist care as early as possible, yet still provide immediate critical interventions. These aims can come into conflict, and there are a number of different means with which to resolve these issues, resulting in large variations in trauma care systems both between and within countries and a consequent significant heterogeneity in the literature. The evidence is weak, but there is a general consensus that the organisation of a group of hospitals into a “trauma system” leads to about a 15% reduction in trauma death, with about a 50% reduction in “preventable death” [46]. Inter-hospital transfer of patients does not seem to change overall mortality [47], and the evidence neither supports nor refutes direct transport from the accident scene to a major trauma centre [48]. However, there is some evidence that a lower threshold for trauma centre care should be used in patients aged > 65 years [49]. No definitive conclusion can be drawn about the relationship between a hospital’s trauma patient volume and outcomes [50, 51]. Despite a lack of evidence, there is a consensus that “systemised” trauma care that matches each patient to the most appropriate treatment facility in a timely manner is advantageous, whereby the definition of “appropriate” will depend on the patient profile, the nature of the injuries and the hospital facilities available [52].

Trauma patients in need of emergency surgery for ongoing haemorrhage have increased survival if the elapsed time between the traumatic injury and admission to the operating theatre is minimised. More than 50% of all trauma patients with a fatal outcome die within 24 h of injury [7]. Despite a lack of evidence from prospective RCTs, well-designed retrospective studies provide evidence for early surgical intervention in patients with traumatic haemorrhagic shock [53, 54]. In addition, studies that analyse trauma systems indirectly emphasise the importance of minimising the time between admission and surgical bleeding control in patients with traumatic haemorrhagic shock [55]. Minimisation of time to surgery is an accepted principle of trauma care and is unlikely to ever be tested in a clinical trial due to lack of equipoise.

#### Local bleeding management

##### Recommendation 2

We recommend local compression to limit life-threatening bleeding. (Grade 1A)

We recommend adjunct tourniquet use to stop life-threatening bleeding from open extremity injuries in the pre-surgical setting. (Grade 1B)

We recommend the adjunct use of a pelvic binder to limit life-threatening bleeding in the presence of a suspected pelvic fracture in the pre-surgical setting. (Grade 1B)

#### Rationale

Most life-threatening bleeding from extremities observed in the civilian setting can be controlled by local compression, by either manual compression or pressure bandages applied to the wounds. Extra local compression to the source of bleeding can also be achieved in certain penetrating injuries by Foley catheter insertion directly into the wound [56]. Foley catheter balloon tamponade was initially described in bleeding penetrating injuries of the neck [57, 58]. In addition, the use of topical haemostatic agents in combination with direct pressure enhances bleeding control in the pre-hospital setting [59] (see also R21).

When uncontrolled arterial bleeding occurs as a result of mangled extremity injuries, including penetrating or blast injuries or traumatic amputations, a tourniquet is a simple and efficient method with which to acutely control haemorrhage [60–64]. Tourniquet application has become the standard of care for the control of severe external haemorrhage following military combat injuries, and several publications report the effectiveness of tourniquets in this specific setting in adults [60–63, 65] and children [66]. A study of volunteers showed that any tourniquet device presently on the market works efficiently [64]. The study also showed that “pressure point control” was ineffective because collateral circulation was observed within seconds. Tourniquet-induced pain was not often reported by patients. No evidence or opinion supports the use of tourniquets in the context of closed injuries.

Tourniquets should be left in place until surgical control of bleeding is achieved [61, 63]; however, this time span should be restricted as much as possible. Improper or prolonged placement of a tourniquet can lead to complications such as nerve paralysis and limb ischaemia [67]; however, these effects are rare [65]. Some publications suggest a maximum application time of 2 h [67]. Reports from military settings describe cases in which tourniquets have remained in place for up to 6 h with survival of the extremity [61]. Much recent discussion has centred on the translation of this evidence to civilian practice, as little published evidence exists. Bleeding from most civilian wounds can be controlled using local pressure; however, uncontrolled external bleeding from either blunt [59] or penetrating [68] limb injury should be controlled with a tourniquet.

Patients with severe high-energy and complex pelvic trauma, haemodynamic instability and massive blood loss belong to the most severe and highly lethal group of trauma patients, and their management is time-sensitive and challenging [69]. Global mortality in polytraumatised patients presenting with pelvic ring fractures remains high (33%) despite improvements in management and treatment algorithms [70]. The pelvis can create a multifocal haemorrhage, including significant retroperitoneal haematoma, which may not be easily compressible or possible to manage using traditional surgical methods [71]. Treatment of pelvic ring fractures requires re-approximation of bony structures to address mechanical instability, damage-control resuscitation (DCR) to restore haemostasis, assessment for associated injuries and triage of investigations. In addition, multimodal haemorrhage control [external fixation and compression (damage-control orthopaedics), retroperitoneal packing (damage-control surgery), urgent radiologic angioembolisation or resuscitative endovascular balloon occlusion of the aorta (REBOA)] by multidisciplinary trauma specialists (general surgeons, orthopaedic surgeons, endovascular surgeons/interventional radiologists) is required [69, 72–75].

Correctly placed pelvic binders lead to anatomical closure of the pelvic ring, with a favourable haemodynamic effect. These devices are increasingly being used in the pre-hospital setting if a pelvic fracture is suspected [76, 77]. Unstable pelvic ring fractures may be clinically and radiologically overlooked during initial assessment, especially in unconscious patients, and the time point for opening and/or removal remains controversial. In-hospital external fixation stabilises anterior pelvic ring lesions and can be combined with posterior stabilisation using percutaneous sacro-iliac screws in the presence of associated lesions to the posterior ring. The external fixator is especially useful in the acute phase, acquiring an acceptable reduction and an adequate stability in the partially unstable lesions and also reduces pelvic volume and bleeding [78]. In a small quasi-randomised study, pelvic packing achieved more rapid control of severe pelvic trauma than angioembolisation [79]. The median time from admission to angiography was 102 min (range 76−214), and longer than 77 min (range 43–125) from admission to pelvic packing (*p* < 0.01). The procedure time for angioembolisation was 84 min (range 62–105), while the surgical time was 60 min (range 41–92; *p* < 0.001). Nine patients had to undergo pelvic packing for persistent bleeding after embolisation. If haemodynamic instability persists, a laparotomy for haemostasis according to damage-control principles to all potentially involved systems (digestive, vascular, urinary and bone) should be performed [80].

#### Ventilation

##### Recommendation 3

We recommend the avoidance of hypoxaemia. (Grade 1A)

We recommend normoventilation of trauma patients. (Grade 1B)

We suggest hyperventilation in the presence of signs of imminent cerebral herniation. (Grade 2C)

#### Rationale

Tracheal intubation of severely injured patients is a delicate procedure that involves risks and requires skill and proper training of the operator. The fundamental objective of intubation is to ensure adequate ventilation and oxygenation and to guarantee the patency of the airway. There are well-defined situations in which intubation is mandatory, for example, in the presence of airway obstruction, altered consciousness [Glasgow Coma Scale (GCS) ≤ 8], haemorrhagic shock, hypoventilation or hypoxaemia [81]; however, other aspects should also be considered. For example, the introduction of positive pressure can induce potentially life-threatening hypotension in hypovolaemic patients [82], and some authors have reported increased mortality associated with pre-hospital intubation [83].

Several factors influence the success of intubation and therefore patient prognosis. Rapid sequence induction appears to be the best method [84]; however, several aspects remain to be clarified, such as who is best suited to make the decision to intubate, which drugs and which rescue device to use and the ideal infrastructure of emergency services. Most of the available data come from retrospective studies, which are open to bias; therefore, controversy remains about the appropriate use of tracheal intubation in patients following traumatic injury [85].

The negative effects of hypoxaemia are well known, particularly in patients with traumatic brain injury (TBI) [86, 87]; therefore, high oxygen concentrations are generally targeted during the initial management of these patients to ensure oxygen delivery to ischaemic areas. Some studies, however, have suggested that prolonged hyperoxia is associated with increased mortality [88, 89]. A recent meta-analysis based on high-quality evidence [90] showed that prolonged liberal oxygen therapy in acutely ill adults increased mortality without improving other patient-important outcomes. Extreme hyperoxia (PaO_2_ > 487 mmHg [> 65 kPa]) should therefore be avoided in patients with TBI [91]. Another recent study showed that the mortality increase was related to the duration and extent of hyperoxia [92]. On the other hand, mechanical ventilation using settings that targeted an oxygen saturation of 88–92% compared with > 95% did not negatively influence survival in critical care patients [93]. The negative effects of hyperoxia are likely related to altered microcirculation associated with high PaO_2_ [94] and increased production of oxygen-free radicals [95] and patients with severe brain injury may be at particular risk [96]. Therefore, although hyperoxia may increase the oxygen content and delivery in an extremely anaemic trauma patient and be associated with a benefit in this specific situation, hyperoxia should be returned to normoxia as soon as the haemoglobin (Hb) level allows [91].

While adequate ventilation can affect the outcome of severe trauma patients, there is a tendency for rescue personnel to hyperventilate patients during initial resuscitation [97, 98]. Hyperventilated trauma patients appear to have increased mortality when compared with non-hyperventilated patients [88]. Target PaCO_2_ should be 5.0–5.5 kPa (35–40 mmHg).

The effect of hyperventilation on bleeding and outcome in patients with severe trauma without TBI is not known. There are several potential mechanisms by which the adverse effects of hyperventilation and hypocapnia could be mediated, including increased vasoconstriction with decreased cerebral blood flow and impaired tissue perfusion. Cerebral tissue lactic acidosis has been shown to occur almost immediately after induction of hypocapnia in children and adults with TBI and haemorrhagic shock [99]. In addition, even a modest level of hypocapnia [< 27 mmHg (3.6 kPa)] may result in neuronal depolarisation with glutamate release and exacerbation of the primary injury via apoptosis [100]. In the setting of absolute or relative hypovolaemia, an excessive rate of positive pressure ventilation may further compromise venous return and produce hypotension and even cardiovascular collapse [101, 102].

The only situation in which hyperventilation-induced hypocapnia may play a potential role is imminent cerebral herniation. The decrease in cerebral blood flow produced by acute hypocapnia during hyperventilation causes a decrease in intracranial pressure that can be used for short periods of time and in selected cases such as imminent brain herniation. The presence of signs such as unilateral or bilateral pupillary dilation or decerebrate posturing are indicators for an extreme risk of imminent death or irreversible brain damage. Hyperventilation may be used under these circumstances to try to gain time until other measures are effective [103, 104]. There are no clinical studies that evaluate this practice; however, there is a clear physiological rationale. Given the extreme risk of death if no measures are undertaken, the risk–benefit balance seems favourable; however, it is important to normalise PaCO_2_ as soon as feasible.

Ventilation with low tidal volume (around 6 mL/kg) is now recommended in all patients treated with mechanical ventilation, even during surgery [105]. Randomised studies demonstrate that short-term ventilation (< 5 h) with high tidal volume (12 mL/kg) without positive end-expiratory pressure (PEEP) may promote pulmonary inflammation and alveolar coagulation in patients with normal lung function [106]. The early use of protective ventilation with low tidal volume and moderate PEEP is recommended, particularly in bleeding trauma patients, who are all at risk of acute respiratory distress syndrome (ARDS).

### II. Diagnosis and monitoring of bleeding

#### Initial assessment

##### Recommendation 4

We recommend that the physician clinically assess the extent of traumatic haemorrhage using a combination of patient physiology, anatomical injury pattern, mechanism of injury and the patient response to initial resuscitation. (Grade 1C)

We suggest that the shock index (SI) be used to assess the degree of hypovolaemic shock. (Grade 2C)

#### Rationale

Trauma physicians must quickly and accurately assess and predict when a massive transfusion protocol, including corresponding logistics, should be activated [107] and terminated [108]. While blood loss may sometimes be obvious, neither visual estimation nor physiological parameters are satisfactory guides to estimate the degree of bleeding [109]. Knowledge about the mechanism of injury provides useful information to identify patients at risk of significant haemorrhage at an early stage. For example, the American College of Surgeons defined a threshold of 6 m (20 ft) as a “critical falling height” associated with major injuries, including haemorrhage [110]. Further critical mechanisms include high-energy deceleration impact as well as low-velocity versus high-velocity gunshot injuries. The mechanism of injury combined with injury severity and the patient’s physiological presentation should further guide the decision to initiate early surgical bleeding control as outlined in the Advanced Trauma Life Support (ATLS) survey [111–114]. Table 3 summarises estimated blood loss based on initial presentation according to the ATLS classification system of hypovolaemic shock. This classification has been shown to be useful as a rough estimation of sustained blood loss in patients with haemorrhagic shock [115]. However, several groups have highlighted discrepancies associated with the weight assigned to each parameter when assessing blood loss that makes it challenging to classify patients using this system. Mutschler and co-workers have analysed the adequacy of this classification and found that > 90% of all trauma patients could not be categorised according to the ATLS classification of hypovolaemic shock [116]. The same group analysed the validity of the ATLS classification and concluded that this system may underestimate mental disability in the presence of hypovolaemic shock, while overestimating the degree of tachycardia associated with hypotension [117]. A retrospective analysis of the validity of the ATLS classification showed that increasing blood loss produces an increase in heart rate and a decrease in blood pressure, but to a lesser degree than suggested by the ATLS classification. In addition, there are no significant changes in respiratory rate or in level of consciousness with bleeding [118]. Other parameters used for this classification, such as pulse pressure and urinary output, may not be adequately assessed during the initial phase of care. The individual response to fluid challenge as suggested by the ATLS survey should be viewed critically in the context of low-volume resuscitation and “permissive hypotension”, which is currently advocated in bleeding trauma patients.

**Table 3:** American College of Surgeons Advanced Trauma Life Support (ATLS) classification of blood loss based on initial patient presentation. Signs and symptoms of haemorrhage by class. Table reprinted with permission from the American College of Surgeons [111]

Parameter	Class I	Class II (mild)	Class III (moderate)	Class IV (severe)
Approximate blood loss	< 15%	15–30%	31–40%	> 40%
Heart rate	↔	↔ / ↑	↑	↑ / ↑↑
Blood pressure	↔	↔	↔ / ↓	↓
Pulse pressure	↔	↓	↓	↓
Respiratory rate	↔	↔	↔ / ↑	↑
Urine output	↔	↔	↓	↓↓
Glasgow Coma Scale score	↔	↔	↓	↓
Base deficit*	0 to − 2 mEq/L	– 2 to −6 mEq/L	– 6 to −10 mEq/L	– 10 mEq/L or less
Need for blood products	Monitor	Possible	Yes	Massive transfusion protocol

*Base excess is the quantity of base (HCO_3_^−^, in mEq/L) that is above or below the normal range in the body. A negative number is called a base deficit and indicates metabolic acidosis

Original data from Mutschler et al. [117]

Isolated vital signs, such as heart rate or systolic blood pressure, have been shown to be unreliable in the assessment of hypovolaemic shock. Heart rate alone has not been shown to predict the need for massive transfusion, in particular not in the geriatric trauma population [119]. In contrast, the SI, defined as the ratio of heart rate to systolic blood pressure, has been advocated to better risk-stratify patients for critical bleeding, increased transfusion requirements and early mortality [120, 121]. Paladino and co-workers found that this index may be useful to draw attention to abnormal values, but may be too insensitive to exclude disease and should not lower the suspicion of major injury [122]. Mutschler and co-workers have suggested a novel and clinically reliable classification of hypovolaemic shock based on four classes of worsening base deficit. The objective of this study was to correlate this classification with corresponding SI strata for the rapid assessment of trauma patients in the absence of laboratory parameters. Twenty-one thousand eight hundred fifty-three adult trauma patients were retrieved from the TraumaRegister DGU® database and divided into four strata of worsening SI at emergency department arrival (group I, SI < 0.6; group II, SI ≥ 0.6 to < 1.0; group III, SI ≥ 1.0 to < 1.4; and group IV, SI ≥ 1.4), and demographics, injury characteristics, transfusion requirements, fluid resuscitation and outcomes were assessed [123]. Worsening of SI was associated with increasing injury severity scores (ISS) from 19.3 (± 12.0) in group I to 37.3 (± 16.8) in group IV, while mortality increased from 10.9 to 39.8%. Increments in SI paralleled increasing fluid resuscitation, vasopressor use and decreasing Hb, platelet counts and Quick values. The number of blood units transfused increased from 1.0 (± 4.8) in group I to 21.4 (±2 6.2) in group IV patients. Of patients, 31% in group III and 57% in group IV required ≥ 10 red blood cell (RBC) units prior to intensive care unit (ICU) admission. Another retrospective database analysis of 10,234 patients has confirmed the role of SI either upon arrival or at departure from the emergency department as a detrimental sign of poor outcome in adult trauma patients [124].

A number of scoring systems that predict the risk of ongoing bleeding, transfusion requirements and coagulopathy have been introduced, but all of these lack prospective validation [108, 125–131]. Each scoring system has its unique advantages and disadvantages, and specific aspects of each scoring system may affect widespread applicability and statistical performance.

#### Immediate intervention

##### Recommendation 5

We recommend that patients with an obvious bleeding source and those presenting with haemorrhagic shock in extremis and a suspected source of bleeding undergo an immediate bleeding control procedure. (Grade 1C)

#### Rationale

The patient who presents in extremis is a patient who has already lost a large amount of blood and is in a severe shock. If bleeding continues, death in shock is an imminent risk. The source of bleeding may be immediately obvious, and penetrating injuries are more likely to require surgical bleeding control. In a retrospective study of 106 abdominal vascular injuries, all 41 patients arriving in shock following gunshot wounds were candidates for rapid transfer to the operating theatre for surgical bleeding control [132]. A similar observation in a study of 271 patients undergoing immediate laparotomy for gunshot wounds indicates that these wounds combined with signs of severe hypovolaemic shock specifically require early surgical bleeding control. This observation is true to a lesser extent for abdominal stab wounds [133]. Data on injuries caused by penetrating metal fragments from explosives or gunshot wounds during the Vietnam War confirm the need for early surgical control when patients present in shock [134]. Following blunt trauma, the mechanism of injury can to a certain extent determine whether the patient in haemorrhagic shock will be a candidate for surgical bleeding control. Only a few studies address the relationship between the mechanism of injury and the risk of bleeding, however, and none of these publications describes a randomised prospective trial with high-level evidence [135]. We have found no objective data describing the relationship between the risk of bleeding and the mechanism of injury resulting in skeletal fractures in general or of long-bone fractures in particular.

Traffic accidents are the leading cause of pelvic injury. Motor vehicle crashes cause approximately 60% of pelvic fractures followed by falls from great height (23%). Most of the remainder result from motorbike collisions and vehicle-pedestrian accidents [136, 137]. There is a correlation between “unstable” pelvic fractures and intra-abdominal injuries [136, 138]. An association between major pelvic fractures and severe head injuries, concomitant thoracic, abdominal, urological and skeletal injuries is also well described [136]. High-energy injuries produce greater damage to both the pelvis and organs. Patients with high-energy injuries require more transfusion units, and more than 75% have associated head, thorax, abdominal or genitourinary injuries [139]. It is well documented that “unstable” pelvic fractures are associated with massive haemorrhage [138, 140], and haemorrhage is the leading cause of death in patients with major pelvic fractures. Vertical shear pelvic ring fractures with caudal displacement of the hemi-pelvis may disrupt the pelvic floor and pelvic vasculature far more than standard vertical shear injuries. Inferior displacement of the hemi-pelvis using X-ray imaging should therefore alert the surgeon to the possible presence of severe arterial injuries [141].

In blunt chest trauma haemothoraces, > 500 mL should trigger chest tube insertion. Thoracotomy is indicated for ongoing bleeding and chest tube output > 1500 mL within 24 h or > 200 mL for three consecutive hours. Acute damage-control thoracotomy should be performed for refractive haemorrhagic shock due to persistent chest bleeding enhanced by initial chest tube output > 1500 mL [142, 143].

#### Further investigation

##### Recommendation 6

We recommend that patients without a need for immediate bleeding control and an unidentified source of bleeding undergo immediate further investigation. (Grade 1C)

#### Rationale

Haemodynamically stable patients, or patients who can be stabilised during initial resuscitation, with an unidentified bleeding source, but not in need of immediate bleeding control, should undergo further investigation of the chest, abdominal cavity and pelvic ring, which can be major sources of acute blood loss following traumatic injury. Besides clinical examination, imaging studies, including ultrasonography and computed tomography (CT) [144], as well as laboratory tests, including blood gas analysis and coagulation profiles, together with functional assays, are recommended diagnostic modalities during the primary survey [111, 145, 146].

As CT scanners are increasingly being advocated and integrated into modern resuscitation units and emergency departments, this technique may replace conventional radiographic imaging and ultrasound as diagnostic measures during the primary survey [147]. The diagnostic accuracy, safety and effectiveness of these immediate measures are dependent on sophisticated pre-hospital treatment by trained and experienced emergency personnel and short transportation times [148, 149]. Proximity of the CT scanner to the resuscitation room in the emergency department has been shown to have a significant positive effect on the probability of survival for the severely injured patient [150]. Distances of more than 50 m had a significant negative effect on outcome and should be considered when new emergency departments are planned and constructed. If a CT scanner is not available in the emergency department, CT scanning implies transportation of the patient to the CT room; therefore, the clinician must evaluate the implications and potential risks and benefits of the procedure. Transfer times to and from all forms of diagnostic imaging need to be considered in the context of haemodynamic stability. During transport, all vital signs should be closely monitored and resuscitation measures continued. If performed quickly within a well-structured environment and by a well-organised trauma team, CT seems to be safe, feasible and justified, even in severely injured haemodynamically unstable patients [151]. Among haemodynamically unstable haemoperitoneum patients, 17.2% had no documented intraperitoneal injury and over half of the patients were treated without emergent surgical intervention [152].

#### Imaging

##### Recommendation 7

We recommend the use of focused assessment with sonography in trauma (FAST) ultrasound for the detection of free fluid in patients with torso trauma. (Grade 1C)

We recommend early imaging using contrast-enhanced whole-body CT (WBCT) for the detection and identification of type of injury and potential source of bleeding. (Grade 1B)

#### Rationale

##### Focused assessment with sonography in trauma (FAST)

The FAST examination has developed into a key instrument in the acute evaluation of trauma patients with suspected abdominal and thoraco-abdominal injuries [153]. FAST techniques are being used with reduced examination times and a focused assessment of specific clinical issues using only a few standardised cross-sectional planes [154]. As a rapid and non-invasive diagnostic approach to the detection of haemorrhages in the peritoneal, pleural and pericardial cavities in the emergency department, FAST represents a cornerstone of the primary ATLS survey [153, 155–157]. Volume status can be assessed non-invasively using ultrasound of the inferior vena cava. Several studies have indicated the specificity and accuracy, but low sensitivity, of initial FAST for detecting and excluding free intraperitoneal fluid as well as intra-abdominal injuries [158–164] in both penetrating [165] and blunt abdominal trauma [166, 167]. Liu and colleagues [168] found a high sensitivity, specificity and accuracy of initial ultrasound examination for the detection of haemoperitoneum. In a retrospective registry study, free fluid or organ injury was detected in 72.4% of patients using FAST versus 84.3% using CT, yielding a sensitivity of 92% for initial FAST [169]. In another retrospective study that included 1540 hypotensive patients (1227 blunt, 313 penetrating trauma), ultrasound examination had a sensitivity and specificity close to 100% for free intra-abdominal fluid [170]. The double-line sign, which has been described as a wedge-shaped hypoechoic area in the Morison pouch, bounded on both sides by echogenic lines during FAST, may represent a false-positive finding for free intraperitoneal fluid with an overall prevalence of 27% [171].

A recent retrospective review examined the role of FAST as a screening tool for identifying intra-abdominal injuries [172]. A total of 1671 blunt-trauma patients were assessed over 1.5 years, and intra-abdominal injuries were confirmed in 146 patients using CT and/or laparotomy. Intraoperative findings included injuries to the liver, spleen, kidneys and bowels. Among 114 haemodynamically stable patients, FAST was positive in 25 patients, with a sensitivity of 22%. FAST was positive in 9 of 32 haemodynamically unstable patients, with a sensitivity of 28%. Free peritoneal fluid and splenic injury were associated with a positive FAST on univariate analysis and were independent predictors of a positive FAST on multiple logistic regression. An updated Cochrane review from 2015, including RCTs, assessed the effect of diagnostic algorithms using ultrasonography, including FAST examinations, in the emergency department relative to early, late and overall mortality of patients with suspected blunt abdominal trauma [173]. Four studies were identified, but the trials were of overall poor to moderate methodological quality. Mortality data were pooled from three trials involving 1254 patients; the risk ratio (RR) in favour of the FAST arm was 1.00 [95% confidence interval (CI) 0.50–2.00]. FAST-based pathways reduced the number of CT scans [random-effects model risk difference (RD) − 0.52, 95% CI − 0.83 to − 0.21], but the meaning of this result remained unclear. It is unlikely that FAST will ever be investigated by means of a confirmatory, large-scale RCT; therefore, this review may provide the best available evidence for clinical practice guidelines and management recommendations. From the few published head-to-head studies, it appears that negative ultrasound scans are likely to reduce the incidence of multidetector CT (MDCT) scans, which, given the low sensitivity of FAST (or reliability of negative results), may adversely affect the diagnostic yield of the trauma survey. At best, ultrasound has no negative impact on mortality or morbidity.

In haemodynamically stable patients, a negative FAST without a CT scan may result in missed intra-abdominal injuries and should direct further diagnostic investigations. A number of patients who present with free intra-abdominal fluid according to ultrasound can safely undergo further investigation using multislice CT (MSCT). Under normal circumstances, adult patients need to be haemodynamically stable when MSCT is performed outside of the emergency department [170]. Haemodynamically stable patients with a high-risk mechanism of injury, such as high-energy trauma, or even low-energy injuries in elderly individuals, should be scanned after ultrasound for additional injuries using MSCT. As CT scanners are integrated into resuscitation units, WBCT diagnosis may replace ultrasound as a diagnostic method. In haemodynamically unstable blunt-trauma patients with clear physical findings on examination, the decision to perform exploratory laparotomy should not be discouraged by a negative FAST [169, 172].

Follow-up sonography as part of secondary or tertiary surveys in patients without abdominal parenchymal organ lesions or free intra-abdominal fluid on initial WBCT is not routinely required, but should be performed if indicated on a clinical or laboratory basis due to its rapid and non-invasive character [174]. New ultrasound techniques using second-generation contrast agents [contrast-enhanced ultrasound (CEUS)] have been developed, allowing all of the vascular phase to be performed in real time, increasing ultrasound capability to detect parenchymal injuries, enhancing some qualitative findings, such as lesion extension, margins and its relationship with capsule and vessels [175]. These techniques are currently under investigation.

##### Computed tomography (CT)

The advantages of MSCT, including WBCT, among severely injured patients in time savings, diagnostic accuracy and potentially also survival have been documented [151, 176–184]. The integration of modern MSCT scanners in the emergency department area prompts immediate assessment of any trauma victim likely to survive the assessment following admission [177, 184], thereby allowing timely diagnosis, differentiation between various types of major vascular injury, identification of associated findings, specific localisation of the source of bleeding and planning for bleeding control [80, 185, 186]. A 1-year review of early management of pelvic fracture patients documented a significant delay in the recognition of (major) pelvic fractures, including those associated with hip dislocations and (potential) pelvic bleeding with selective pelvic X-ray versus CT scanning [187]. More than one third of patients with thoracic stab wounds presented with negative chest X-ray, but pathologies using CT [188].

MDCT is currently considered the “gold standard” in the assessment of intra-abdominal blunt-traumatic injury [189]. Mesenteric active bleeding, adjacent interloop-free fluid and bowel wall perfusion defects have been associated with surgically significant bowel injuries and an overall accuracy, sensitivity, specificity, positive predictive value (PPV) and negative predictive value (NPV) for 64-slice MDCT of 73.8%, 80.0%, 73.0%, 28.6%, and 96.4%, respectively [190]. Advancements in modern MDCT technology and an improved understanding of optimal protocols have enabled full-body scanning of adequate image quality and in less than 30 s. In a retrospective study comparing 370 patients in two groups, Weninger and colleagues [184] showed that faster diagnosis using MSCT led to shorter emergency department and operating room time and shorter ICU stays [184]. Huber-Wagner et al. also showed the benefit of WBCT integration into early trauma care as CT diagnosis significantly increased the probability of survival in patients with polytrauma [147, 150]. WBCT as a standard diagnostic tool during the earliest resuscitation phase provides the added benefit of identifying head and chest injuries and other bleeding sources in multiply injured patients. Nonselective throracic CT was superior to selective CT in detecting thoracic injuries in blunt trauma [191], and thoracic CT showed a NPV value of 99% in triaging haemodynamically normal patients with penetrating chest trauma [192]. A comparison between emergency physicians and on-call radiologists on the accuracy of CT interpretations showed that emergency physicians were successful in identifying fatal injuries on trauma scans following a short-term interpretation training [193].

A series of systematic reviews has assessed the benefits of WBCT in the early management of severely injured patients and all showed a survival benefit with the use of WBCT in trauma patients [194–197]. In contrast, the only prospective RCT conducted to date in this area compared immediate WBCT scanning versus conventional imaging and selective CT scanning in patients with severe trauma [A Multicenter, Randomised Study of Early Assessment by CT Scanning in Severely Injured Trauma Patients (REACT-2)] in four centres in the Netherlands and one in Switzerland and found no survival benefit with WBCT [198]. A total of 1403 trauma patients aged ≥ 18 years with compromised vital parameters, clinical suspicion of life-threatening injuries or severe injury were randomly assigned (1:1) to immediate WBCT scanning or to a standard work-up with conventional imaging supplemented with selective CT scanning. The primary analysis included 541 patients in the immediate WBCT scanning group and 542 in the standard work-up group. In-hospital mortality did not differ between groups (WBCT 86 [16%] of 541 vs standard work-up 85 [16%] of 542; *p* = 0.92). In-hospital mortality also did not differ in subgroup analyses among patients with polytrauma (WBCT 81 [22%] of 362 vs standard work-up 82 [25%] of 331; *p* = 0.46) and TBI (68 [38%] of 178 vs 66 [44%] of 151; *p* = 0.31).

The WBCT protocol usually includes a non-contrast scan of the brain and neck followed by a contrast-enhanced scan of the chest, abdomen and pelvis. Several authors have emphasised the benefit of contrast medium-enhanced CT scanning. MSCT is the “gold standard” for the identification of retroperitoneal haemorrhage (RPH). After injection of intravenous (i.v.) contrast media, CT identified RPH in all cases (100%) and may detect the source of bleeding (40%) by extravasation of contrast media [199]. Dual-phase contrast-enhanced CT (CECT) without CT angiography showed a high sensitivity (93.9%) and PPV (88.6%) compared with digital subtraction angiography for the detection of active haemorrhage in patients with blunt abdominopelvic trauma [200]. Anderson et al. [201, 202] found high accuracy in the evaluation of splenic injuries resulting from trauma after administration of an i.v. contrast medium. Delayed-phase CT may be used to detect active bleeding in solid organs. Fang et al. [203] demonstrated that the pooling of contrast material within the peritoneal cavity in blunt liver injuries indicates active and massive bleeding. Patients with this finding showed rapid deterioration of haemodynamic status, and most required emergent surgery. Intra-parenchymal pooling of contrast material with an unruptured liver capsule often indicates a self-limited haemorrhage, and these patients respond well to non-operative treatment. Tan and colleagues [204] found that patients with hollow viscus and mesenteric injuries following blunt abdominal trauma exhibited an abnormal preoperative CT scan. Wu et al. [205] confirmed the accuracy of CT in identifying severe, life-threatening mesenteric haemorrhage and blunt bowel injuries. Although contrast extravasation (CE) in CT scans of pelvises with blunt trauma may be common, many patients will not require intervention such as angioembolisation [206]. The negative predicted value of 100% should be reassuring to trauma surgeons such that if a modern CT scanner is used, and no CE is detected using CT, then the pelvis is unlikely to be a source of haemorrhagic shock. All of these findings are attributable to both increased comfort with observing CEs and the increased sensitivity of modern CT scanners.

The issue of radiation is still debated, but iterative as well as split-bolus protocols can now significantly reduce radiation exposure [207]. Imaging algorithms including WBCT in multi-trauma patients are standardised but may vary substantially between centres [208]. An online survey among level-1 trauma centres in Switzerland revealed radiation doses ranging from 1268 to 3988 mGy × cm per WBCT. Including WBCT in the initial work-up of trauma patients results in higher radiation doses, but fewer additional CT examinations are needed, and the time to complete trauma-related imaging is shorter [209]. Risk-stratification criteria based upon documented suspected injuries during the primary survey at the site of the accident or the emergency department may identify high-energy trauma patients not in need of extended radiological imaging, including WBCT [210]. To a large extent, WBCT in high-energy trauma patients does not affect patient care if the patient is mentally alert, not intoxicated or showing signs of more than minor injuries when clinically evaluated. The risk of missing important traumatic findings in these patients is very low. Observation of the patient with re-examination instead of imaging may be considered in this group, often young patients, for whom radiation dose is an issue [210]. Davies and co-workers have developed a scoring system with a sensitivity of 97% (95% CI 88–99%) and a specificity of 56% (95% CI 49–64%) for significant injury to stratify the use of trauma radiographs, focused on CT and WBCT, and which may add an objective component to decision-making to reduce unnecessary scans [211]. Regression modelling identified clinical signs in more than one body region, reduced GCS, haemodynamic abnormality, respiratory abnormality and mechanism of injury as independent predictors of polytrauma.

#### Haemoglobin

##### Recommendation 8

We recommend that a low initial Hb be considered an indicator for severe bleeding associated with coagulopathy. (Grade 1B)

We recommend the use of repeated Hb measurements as a laboratory marker for bleeding, as an initial Hb value in the normal range may mask bleeding. (Grade 1B)

#### Rationale

Hb or haematocrit (Hct) assays are part of the basic diagnostic work-up for trauma patients. Recently, non-invasive Hb monitoring has also been tested and showed high precision compared with laboratory measurements [212, 213]. Currently, the use of Hb rather than Hct is widespread, and the latter is a calculated parameter derived from the Hb. However, most studies on which these recommendations are based analysed Hct rather than Hb. Because both parameters are used interchangeably in clinical practice, in these guidelines, we refer to both parameters according to the parameter described by the literature.

The diagnostic value of the Hb or Hct for detecting trauma patients with severe injury and occult bleeding sources has been a topic of debate [214–216]. A major limitation of the diagnostic value is the confounding influence of resuscitation measures on the Hb/Hct due to administration of i.v. fluids and erythrocyte concentrates [217–219]. In addition, initial Hb or Hct measurements may not accurately reflect blood loss, because patients bleed whole blood and compensatory mechanisms that move fluids from interstitial spaces require time. The suggestion that initial Hb/Hct for the detection of severe bleeding is associated with low sensitivity has been challenged. In a retrospective study of 196 trauma patients, Ryan et al. [220] found that Hct at admission closely correlates with haemorrhagic shock. Knottenbelt et al. evaluated 1000 trauma patients and found lower initial Hb level in moderately and severely shocked patients [221]. Other authors also recommended that the initial Hct play a greater role in the assessment of blood loss in trauma patients. In a retrospective analysis of 1492 consecutive trauma patients, Thorson et al. found that the initial Hct is associated more closely with the need for transfusion than other parameters such as heart rate, blood pressure or acidaemia, suggesting that fluid shifts are rapid following traumatic injury and imply a more important role for Hct in the initial assessment of trauma victims [222]. An initial low Hb level is one of the predictive criteria for massive transfusion using the trauma-associated severe haemorrhage (TASH) [126] and Vandromme [223] scores.

Thorson et al. [224] analysed changes in Hct in two successive determinations and concluded that the change in Hct is a reliable parameter with which to detect blood loss. Two prospective observational diagnostic studies also showed the sensitivity of serial Hct measurements for the detection of patients with severe injury [214, 216]. Holstein and co-workers showed that a Hb level below 80 g/L in patients with pelvic trauma was associated with non-survival [225]. Decreasing serial Hct measurements may reflect continued bleeding. However, a patient with significant bleeding may maintain the serial Hct in the context of ongoing resuscitation and physiological compensatory mechanisms. Acute anaemia may play an adverse role in the clotting process, because a low Hct may reduce platelet marginalisation, with a potentially negative impact on platelet activation. Moreover, Schlimp et al. [226] demonstrated strong correlation between fibrinogen levels and Hb.

#### Serum lactate and base deficit

##### Recommendation 9

We recommend serum lactate and/or base deficit measurements as a sensitive test to estimate and monitor the extent of bleeding and shock. (Grade 1B)

#### Rationale

Serum lactate has been used as a diagnostic parameter and prognostic marker of haemorrhagic shock since the 1960s [227]. The amount of lactate produced by anaerobic glycolysis is an indirect marker of oxygen debt, tissue hypoperfusion and the severity of haemorrhagic shock [228–231]. Similarly, base deficit values derived from arterial blood gas analysis provide an indirect estimation of global tissue acidosis due to impaired perfusion [230, 231]. Vincent and colleagues [232] showed the value of serial lactate measurements for predicting survival in a prospective study in patients with circulatory shock. This study showed that changes in lactate concentration provide an early and objective evaluation of patient response to therapy and suggested that repeated lactate determinations represent a reliable prognostic index for patients with circulatory shock [232]. Abramson and colleagues [233] performed a prospective observational study in patients with multiple traumatic injuries to evaluate the correlation between the time course of blood lactate levels and survival. All patients in whom lactate levels returned to the normal range (≤ 2 mmol/L) within 24 h survived. Survival decreased to 77.8% if normalisation occurred within 48 h and to 13.6% in those patients in whom lactate levels were elevated above 2 mmol/L for more than 48 h [233]. These findings were confirmed in a study by Manikis et al., who showed that initial lactate levels were higher in non-survivors after major trauma and that prolongation of time to normalisation of lactate levels of more than 24 h was associated with the development of post-traumatic organ failure [234]. The determination of lactate and/or base deficit may be particularly important in penetrating trauma. Following this type of injury, triage vital signs, such as blood pressure, heart rate and respiratory rate, do not reflect the severity of injury and are not related to lactate or base deficit levels [235]. A systemic review on the value of blood lactate kinetics in critically ill patients has been published recently [236].

The reliability of lactate determination may be lower when traumatic injury is associated with alcohol consumption. Ethanol metabolism induces the conversion of pyruvate to lactate via lactate dehydrogenase, causing an increase in the level of lactate in the blood. In alcohol-associated trauma, therefore, base deficit may be a better predictor of prognosis than lactate [237], although some authors suggest that ethanol-induced acidosis may also affect base deficit, masking the prognosis of trauma patients [238]. Therefore, in the case of traumatic injury associated with alcohol consumption, the results of the lactate measurements should be interpreted with caution.

Similar to the predictive value of lactate levels, the initial base deficit, obtained either from arterial or peripheral venous blood [239] has been established as a potent independent predictor of mortality in patients with traumatic haemorrhagic shock [237]. Davis and colleagues stratified the extent of base deficit into three categories: mild (− 3 to − 5 mEq/L), moderate (− 6 to − 9 mEq/L) and severe (<− 10 mEq/L) and established a significant correlation between the admission base deficit, transfusion requirements within the first 24 h and the risk of post-traumatic organ failure or death [240]. The same group of authors showed that the base deficit is a better prognostic marker of death than the pH in arterial blood gas analyses [241]. Mutschler et al. [123] analysed a cohort of 16,305 severely injured patients derived from the German Trauma Registry database and concluded that the determination of base deficit upon emergency department admission predicts transfusion requirements and mortality better than ATLS classification [123]. Furthermore, the base deficit was shown to represent a highly sensitive marker for the extent of post-traumatic shock and mortality, both in adult and paediatric patients [242, 243].

Although both the base deficit and serum lactate levels are well correlated with shock and resuscitation, these two parameters do not strictly correlate with each other in severely injured patients [244], and lactate levels more specifically reflect the degree of tissue hypoperfusion [230, 231, 244].

#### Coagulation monitoring

##### Recommendation 10

We recommend that routine practice include the early and repeated monitoring of haemostasis, using either a combined traditional laboratory determination [prothrombin time (PT), platelet counts and Clauss fibrinogen level] and/or point-of-care (POC) PT/international normalised ratio (INR) and/or a viscoelastic method (VEM). (Grade 1C)

We recommend laboratory screening of patients treated or suspected of being treated with anticoagulant agents. (Grade 1C)

#### Rationale

Standard coagulation monitoring comprises early and repeated determination of PT, platelet counts and Clauss fibrinogen level. The PT measures the activity of the extrinsic coagulation pathway (factors II, VII, and X), resulting in a prolonged PT value when any of these factors is low. There is frequently confusion in the literature over the terms PT and INR, because they are often used interchangeably, despite being based on different comparative values. Strictly speaking, PT is the ratio of the patient’s PT compared with a PT performed using pooled plasma from healthy individuals. Conventionally, PT testing has been used for all patients except those treated with a vitamin K antagonist (VKA). The INR, on the other hand, represents a PT in which the activating tissue factor used in the assay is assigned a value such that the effect of the VKA is consistent across laboratories.

Because the definition of traumatic coagulopathy is equivalent to a prolongation of the PT [11], PT values on admission have been shown to correlate with the degree of shock and to be predictive of clinical outcome in the presence of traumatic haemorrhage. Peltan et al., for example, found that acute traumatic coagulopathy affected 50% of patients with traumatic bleeding, defined as a PT:INR ratio > 1.2 and 21% of subjects if traumatic coagulopathy was defined as an INR > 1.5 [245]. The latter was significantly associated with all-cause death, haemorrhagic shock-associated death, venous thromboembolism (VTE) and multiple organ failure. As a result, PT/INR is used to assess the severity of traumatic coagulopathy and the need for transfusion.

Recently, POC monitors (portable coagulometers) that assess the INR have improved in quality and ease of use. They are widely applied by professionals in anticoagulant clinics and at home by patients to monitor the effect of VKAs. Use may be more common in the emergency department to identify patients with significant coagulopathy compared with laboratory-based methods [246, 247]. It is, however, important to note that variation between these devices and a laboratory-based PT may be 15% [246, 248]. David et al. suggest that a near-patient INR value of 1.5 could be used to guide fresh frozen plasma (FFP) or prothrombin complex concentrate (PCC) administration [247]. Goodman et al. demonstrated that POC INR testing was more rapid and cheaper than a modified thrombelastography [TEG®; rapid TEG® (r-TEG®)] and correlated not only with r-TEG® values, but also with blood product transfusion [249].

It is often misunderstood that the conventional coagulation screens [PT and activated partial thromboplastin time (APTT)] only provide information on levels of coagulation factor [250]. These values, therefore, will typically appear normal during early blood loss, despite the potential for an underlying activation of coagulation and thrombus formation [251–254]. The turnaround time for results of VEM [TEG®, rotational thromboelastometry (ROTEM®)], as for POC PT/INR, has been shown to be significantly shorter than conventional laboratory testing, with a time saving of 30–60 min [251, 255, 256]. VEM may also be useful in the detection of coagulation abnormalities associated with the use of direct thrombin inhibitors such as dabigatran, argatroban, bivalirudin or hirudin, although these tests cannot discriminate between the effects of inhibitors and the impact of traumatic coagulopathy [257].

VEM provides a rapid assessment of haemostasis to support clinical decision-making. This in turn has generated a growing confidence in these methods and increased use in children, adolescent and adult patients [29, 256, 258]. To date, however, only one open randomised controlled study has been completed, which involved 111 injured patients from an academic level-1 trauma centre meeting criteria for massive transfusion protocol activation [259]. Patients were randomised to receive either a massive transfusion protocol goal-directed using TEG® or by conventional coagulation assays (CCA). Survival at 28 days in the TEG® group was significantly higher than the CCA group, with 20 deaths in the CCA group (36.4%) compared with 11 in the TEG® group (19.6%) (*p* = 0.049). Most bleeding deaths occurred within the first 6 h following patient arrival at the clinic (21.8% CCA group vs 7.1% TEG® group) (*p* = 0.032). CCA patients required a similar number of RBC units as the TEG® patients but more plasma units [CCA, 2.0 (0–4); TEG®, 0.0 (0–3)] (*p* = 0.022), and more platelet units [CCA, 0.0 (0–1); TEG®, 0.0 (0–0)] (*p* = 0.041) in the first 2 h of resuscitation. Despite these very promising results, it should be noted that this study was open, unblinded, and that randomisation into either of the two treatment modalities was based on alternating weeks, which potentially introduces a bias into the care of the patients.

r-TEG® is a new variant of VEM in which coagulation is initiated by the addition of kaolin and tissue factor, which appears to reduce the measurement time compared with conventional TEG® in adults [260, 261] and children [262, 263]. One of several validation studies included 808 adult trauma patients in a prospective international multicentre cohort study from four major trauma centres. The authors demonstrated that a ROTEM® clot amplitude of 5 mm was a valid marker for acute traumatic coagulopathy and a predictor of massive transfusion [22]. Meyer et al. evaluated fibrinogen levels in trauma patients determined using two whole-blood VEM, TEG® functional fibrinogen (FF) and ROTEM® FIBTEM (FIBTEM, fibrin-based extrinsically activated test) and compared these with the plasma-based Clauss method. Both methods correlated with the Clauss fibrinogen level, without variation in the strength of these correlations [264].

Recent discussion has focused on the specific usefulness of VEM in the detection of early fibrinolysis. On the one hand, Moore et al. found that VEM only demonstrates hyperfibrinolytic traces in a minority of those with traumatic bleeding [265]. On the other hand, Brohi et al. have shown that VEM is a poor detector of fibrinolytic activation, which they suggest may be due to the production of soluble S100A10 from the endothelium, thereby blocking detection of tissue plasminogen activator by VEM [266]. The widespread use of tranexamic acid (TXA) in trauma patients may be expected to counteract acute fibrinolysis in these patients. At this time, therefore, it is not possible to support the use of VEM as a superior option over conventional coagulation tests. Results from the global multicentre Implementing Treatment Algorithms for the Correction of Trauma Induced Coagulopathy (iTACTIC) study are expected to reveal how the use of VEM might impact clinical outcomes [267].

Despite the widespread use of VEM, their usefulness is still being evaluated. In a recent systematic Cochrane review, Hunt et al. [268] found no evidence for the accuracy of TEG®, and very little evidence to support the accuracy of ROTEM®, therefore were unable to offer any advice about the use of these methods [268]. In another systematic review, Da Luz et al. [269] concluded that only limited evidence from observational studies was available to support the use of VEM in the diagnosis of early traumatic coagulopathy. While these tests may be used to predict blood product transfusion, mortality and other important patient outcomes may be unaffected [269]. A number of other limitations associated with the use of VEM have been described elsewhere. TEG® may lead to unnecessary transfusion with platelets, whereas the application of ROTEM® may result in goal-directed fibrinogen substitution. Although use is rapidly increasing, controversy remains at present regarding the utility of VEM for the detection of posttraumatic coagulopathy.

Agreement between the results of VEM and standard coagulation tests also remains a matter of debate. Some studies find acceptable agreement between results [261, 263, 270], while a number of other studies show significant discrepancies, even among different VEM (TEG® and ROTEM®) [29, 248, 271, 272]. In one instance, Agren et al. suggest that TEG® functional analyses may have overestimated fibrinogen levels (by more than one gram per litre) [272]. Elsewhere, Hagemo et al. found that the correlation was highly variable at different stages of the clotting process and between centres [273], highlighting the need for clarification and standardisation of these techniques. One additional potential limitation of VEM may be the lack of sensitivity in detecting and monitoring platelet dysfunction due to antiplatelet drugs. If platelet dysfunction is expected, POC platelet function tests, for example whole-blood impedance aggregometry, should be used in addition to VEM. More research is required to understand these variations, and in the meantime, physicians should use their own judgement when developing local policies.

Eventually, new POC devices to measure fibrinogen concentration could represent a new means with which to assess traumatic coagulopathy. Several monitors are in development [274] and may compete with VEM in the near future.

The increasing use of pre-injury anticoagulants and, in particular, the so-called direct (non-vitamin K-dependent) oral anticoagulants (DOACs) pose an increasing challenge in the setting of trauma haemorrhage, as these agents can substantially complicate the extent and dynamics of bleeding [275]. Retrospectively, preexisting coagulation disorders, either congenital or acquired, e.g. due to anticoagulant intake, were associated with an elevated mortality in trauma patients with and without head injury (43% versus 17% [276–279]). While VKAs and antiplatelet agents (APA) can be assessed using INR measurements and platelet function assays, to date there is no universally available and validated (rapid) test system for any of the DOACs that is associated with meaningful sensitivity and specificity [275]. The standard PT (preferably the INR) is prolonged in VKA-treated patients. If time and amount of the most recent dose of dabigatran are unknown, normal values for thrombin time, ecarin clotting time and diluted thrombin time suggest the absence of dabigatran in clinically relevant concentrations [275]. A normal standard anti-Xa test may also exclude intake (or efficacy) of an anti-Xa agent (rivaroxaban, apixaban, edoxaban, betrixaban). If these tests are prolonged, a diluted thrombin time (Hemoclot® for dabigatran) or a specific anti-Xa test (for anti-Xa agents) should be performed [280]. Chromogenic anti-factor-Xa-activity tests can be used to estimate the plasma concentrations of factor Xa-inhibitors (apixaban, edoxaban, rivaroxaban), but require calibration with substance-specific reagents [275, 281, 282].

#### Platelet function monitoring

##### Recommendation 11

We suggest the use of POC platelet function devices as an adjunct to standard laboratory and/or POC coagulation monitoring in patients with suspected platelet dysfunction. (Grade 2C)

#### Rationale

Traumatic injury has been associated with platelet dysfunction [283–285]. Unfortunately, neither CCAs nor standard VEM reliably reflect platelet function status [286, 287]. Light transmission aggregometry (LTA), considered the gold standard for the assessment of platelet function, is inadequate in the acute setting [288]. Several POC platelet function devices are available, such as the platelet function analyser (PFA-100®), whole-blood multiple electrode impedance aggregometry (MEA), platelet reactivity assay (e.g. VerifyNow®), vasodilator-stimulated phosphoprotein (VASP) or VEM devices with channels for measuring platelet function. Different POC tests capture different aspects of platelet function and are therefore not interchangeable in the assessment of platelet reactivity. However, these devices may be of value in detecting pharmacologically induced platelet inhibition in trauma patients for whom prior intake of antiplatelet agents (APA) is unknown, for example in unconscious or confused patients, and in patients with uncertain treatment compliance.

The VerifyNow® platelet reactivity test for aspirin (VN®-ASA) successfully identified TBI patients who reported using aspirin therapy [289, 290]. The MEA device allowed rapid assessment of APA activity in patients admitted for intracranial haemorrhage (ICH) requiring urgent neurosurgical intervention [291] and in TBI [283, 292–294]. The thrombelastography platelet mapping (TEG®-PM®) assay also identified APA use in trauma patients [286, 295]; however, PFA-100 showed low sensitivity and PPVs (48.6% and 63.4%, respectively) for detecting pharmacologically induced platelet dysfunction in trauma patients on APA [296]. In one study comparing MEA, VerifyNow® and TEG®-PM® in adult trauma patients, specific tests for the arachidonic acid (AA) pathway in all three devices accurately identified any APA use (either aspirin or clopidogrel) [286]. AA tests to identify platelet dysfunction performed with TEG®-PM® and VerifyNow® devices correlated well with MEA [area under the curve (AUC) 0.78, 0.89, respectively]. However, MEA and VerifyNow® had superior AUCs compared with the TEG®-PM® percent inhibition AUC (both 0.90 vs 0.77). The adenosine diphosphate (ADP)-specific assays on these three devices did not correlate with APA use; however, the number of patients pre-treated with clopidogrel was small [286]. Trauma patients with normal platelet activity despite a positive history of APA intake (“non-responders”) or patients with high on-treatment platelet reactivity (HTPR) can also be identified using VerifyNow® [289, 290, 293, 297, 298]. In these patients, empiric administration of haemostatic substances would unnecessarily increase the risk of thrombotic events.

VerifyNow® [286, 289, 290, 293, 297, 298], MEA [283, 284, 286, 292, 294, 299–301] and TEG®-PM® [286, 287, 295, 302–305] can also be used to detect platelet dysfunction in trauma patients in the absence of APA intake. A coagulopathy POC panel consisting of r-TEG® and VN®-ASA successfully identified a subset of TBI patients with an occult coagulopathy that would otherwise have been missed [290]. Platelet dysfunction, as indicated by MEA, exhibits a temporal profile whereby MEA values are low initially and subsequently increase during the days following TBI [286, 289, 290, 292, 293, 297], similar to the changes observed perioperatively in elective hip arthroplasty [306]. Interestingly, both the ADP pathway and the thrombin receptor pathway measured using a thrombin receptor activating peptide (TRAP) test are significantly affected in trauma patients [301].

Distinguishing pharmacologic from trauma-induced platelet receptor hypofunction is not easy, as both conditions are associated with assay values below the reference interval. Moreover, diagnostic cut-offs for pathologic platelet dysfunction after traumatic injury have not been established. For example, ADP inhibition measured by TEG®-PM® was 42.5% in one study [287] and as high as 86% in another [285], compared with only 4% in healthy volunteers [287]. Over 75% of the TBI patients had impairment of the ADP pathway in one study [265] and the severity of brain injury appeared to correlate with ADP inhibition on TEG®-PM® (severe TBI 93.1%, mild TBI 56.5%, control 15.5%; *p* < 0.01) [302]. When TEG®-PM® and MEA were compared in severely injured trauma patients, results correlated poorly with the ADP pathway and moderately with the AA pathway [299].

The utility of POC platelet function assays to predict outcome or stratify trauma patients at a higher risk of bleeding who may benefit subsequently from transfusion is uncertain. By using a composite outcome, one study found no difference in bleeding complications in trauma patients on clopidogrel who presented with high or low platelet inhibition measured using VerifyNow® [298]. Similarly, progression of ICH and the need for neurosurgical intervention was independent of platelet activity assessed using VerifyNow® [307]. MEA values were also not predictive of haemorrhagic progression [292] or outcome [289, 292, 294] in some studies in trauma patients; however, 87% of patients received haemostatic therapy following detection of impaired platelet function, and this strategy could have influenced the results in one study [294]. In contrast, the MEA TRAP [283] and the AA receptor aspirin inhibition (ASPI) test [299] were independent predictors of mortality. In another study that included a mixed trauma population, which was not adjusted for confounders, ADP and TRAP values were also different between survivors and non-survivors [284]. Others have found ADP, but not the AA test, to be a predictor of mortality [303].

TEG®-PM® was found to be a superior indicator of haemorrhagic shock in trauma patients compared with MEA [299]. TEG®-PM® AA-induced platelet activity reduction identified TBI patients with a high risk of bleeding complications [304] and TEG®-PM® ADP-induced platelet activity reduction [285] or inhibition in both pathways [299] was predictive of blood product transfusion in severe trauma. Another study demonstrated that the MEA and VerifyNow® AA tests were not predictive of ICH, whereas the TEG®-PM® AA percent inhibition may be associated with ICH progression, with 71% specificity at 32% inhibition [286]. Studies reporting ADP receptor inhibition measured using TEG®-PM® also showed an association between this parameter and mortality [287] and significant correlations between the severity of TBI, the degree of ADP inhibition and increased risk of mortality [302–304]. In one study, platelet ADP inhibition exceeding 60% independently predicted in-hospital mortality amongst patients with TBI, while controlling for age, gender, the presence of hypotension, pre-injury APA, GCS and ISS [295]. In contrast, others found no correlation between TEG®-PM® values and ISS, length of hospital stay or mortality in trauma patients with or without TBI [305].

The role of POC platelet function devices in guiding haemostatic therapy is not established. One study showed no impact of platelet transfusion on platelet activity in patients with traumatic ICH with pre-injury aspirin treatment assessed using the VerifyNow® assay. There was also no difference in ICH progression or neurosurgical intervention in functional and non-functional platelet groups after platelet transfusion [307]. Further studies using the VerifyNow® assay showed that a single platelet apheresis unit was not sufficient to reverse platelet inhibition in almost half of patients [297] and a trend toward increased mortality in patients whose platelet function failed to normalise with transfusion [289]. A dose–response relationship between the quantity of platelets transfused and reversal of VN®-ASA inhibition was observed [289].

In contrast, haemostatic measures significantly increased AA-induced platelet activity measured using MEA by 100 ± 66% [291]. Others showed that platelet transfusion improved aspirin-induced platelet dysfunction but did not recover traumatic platelet dysfunction measured using MEA [308]. TEG®-PM® was also not supported as a solitary tool to guide platelet transfusions in trauma patients [287, 305]. It seems that although platelet transfusion may improve platelet function via AA receptor-mediated pathways, it has little, if any, impact on ADP receptor-mediated pathways [305]. Moreover, TBI patients who received platelet transfusion had significant reductions in the degree of platelet inhibition detected using the AA TEG®-PM® assay, but no change in outcomes [309].

The lack of congruency among the studies summarised above indicates that there is a pressing need for future prospective studies that investigate the potential benefit of platelet function monitoring in trauma patients. Although these devices are capable of measuring platelet receptor inhibition to detect pre-treatment with APA, their role in identifying trauma-induced platelet dysfunction and in guiding haemostatic therapy remains unclear and their use can only be recommended as an adjunct to standard laboratory monitoring.

### III. Tissue oxygenation, volume, fluids and temperature

#### Tissue oxygenation

##### Recommendation 12

We recommend permissive hypotension with a target systolic blood pressure of 80–90 mmHg (mean arterial pressure 50–60 mmHg) until major bleeding has been stopped in the initial phase following trauma without brain injury. (Grade 1C)

In patients with severe TBI (GCS ≤ 8), we recommend that a mean arterial pressure ≥ 80 mmHg be maintained. (Grade 1C)

#### Restricted volume replacement

##### Recommendation 13

We recommend use of a restricted volume replacement strategy to achieve target blood pressure until bleeding can be controlled. (Grade 1B).

#### Vasopressors and inotropic agents

##### Recommendation 14

In the presence of life-threatening hypotension, we recommend administration of vasopressors in addition to fluids to maintain target arterial pressure. (Grade 1C)

We recommend infusion of an inotropic agent in the presence of myocardial dysfunction. (Grade 1C)

#### Rationale

At present, the initial treatment of trauma-induced hypotension uses the concept of DCR, with restricted volume replacement and permissive hypotension. Although the general effectiveness of such a restricted volume replacement, resulting in permissive hypotension, remains to be confirmed in RCTs, two studies published in the 1990s demonstrated increased survival when a low and delayed fluid volume resuscitation concept was used in penetrating [310] or penetrating and blunt [311] trauma. A further small pilot RCT published in 2015 demonstrated a 24-h survival benefit for hypotensive patients with blunt trauma initially treated with a restrictive volume administration when compared with standard volume replacement [312]. However, in contrast to these studies, no significant differences in survival were found in two non-randomised controlled trials examining patients with either penetrating and blunt trauma [313] or blunt trauma alone [314].

Moreover, future RCTs must also confirm whether the present more or less arbitrary recommendation for systolic and mean arterial blood pressures for permissive hypotension are safe for all trauma patients or whether target blood pressures should be different in specific subgroups, e.g. in blunt or penetrating trauma patients. Existing data already show that the concept of permissive hypotension should be carefully considered in the elderly patient [315] and may be contraindicated if the patient suffers from chronic arterial hypertension [316].

Nevertheless, the concept of DCR is supported by several retrospective studies demonstrating that aggressive resuscitation techniques, often initiated in the pre-hospital setting, may be detrimental for trauma patients [14, 34, 317–325]. It has been shown that aggressive volume administration increased the incidence of secondary abdominal compartment syndrome (ACS) [324], damage-control laparotomy [322], coagulopathy [14], multiple organ failure [323], nosocomial infections [323], the number of blood as well as mass transfusions [319, 323] and prolonged the length of ICU and hospital stays [323]. At the same time, increased volume administration decreased the likelihood of survivial [34, 320, 321, 323].

The timing and volume of i.v. fluid administration in bleeding trauma patients was assessed in a meta-analysis by Kwan et al. [326]. Three trials, including a total of 1957 patients, were identified that addressed the timing of administration, and three other studies investigated volume load, but included only 171 patients. In contrast to the retrospective analysis described above, the meta-analysis failed to demonstrate an advantage associated with delayed compared to early fluid administration, nor of smaller compared to larger volume fluid administration in this small group of prospective studies that included only a very limited number of patients. A further meta-analysis that assessed seven retrospective observational studies that included a total of 13,687 patients and three prospective studies that included 798 patients estimated a small benefit in favour of a restricted volume replacement strategy [327]. However, the authors cautioned that the available studies were subject to a high risk of selection bias and clinical heterogeneity.

It should be noted that DCR strategies using restrictive volume replacement affecting hypotensive blood pressure are contraindicated in patients with TBI and spinal injuries. This is because an adequate perfusion pressure is crucial to ensure tissue oxygenation of the injured central nervous system [328]. However, it remains unclear how to attain the best balance between volume resuscitation and vasopressor administration in order to achieve an adequate perfusion pressure. [315, 316]

In conclusion, a DCR strategy using a concept of restricted fluid replacement that aims to achieve a lower than normal systolic blood pressure of 80–90 mmHg in patients without TBI and/or spinal injury is supported by the literature. However, strong evidence from sufficiently robust RCTs is lacking.

Vasopressors may also be required transiently, even when fluid expansion is in progress and hypovolaemia has not yet been corrected, to sustain life and maintain tissue perfusion in the presence of life-threatening hypotension. Norepinephrine is commonly used to restore arterial pressure in septic and haemorrhagic shock and is now considered by many to be the agent of choice for this purpose during septic shock [329]. Although norepinephrine has some β-adrenergic effects, it acts predominantly as a vasoconstrictor. Arterial α-adrenergic stimulation increases arterial resistance and may increase cardiac afterload, while norepinephrine exerts both arterial and venous α-adrenergic stimulation [330]. Indeed, in addition to its arterial vasoconstrictor effect, norepinephrine induces venoconstriction at the level of the splanchnic circulation in particular, which increases the pressure in capacitance vessels and actively shifts splanchnic blood volume to the systemic circulation [331]. This venous adrenergic stimulation may to some extent recruit blood from the venous unstressed volume, thereby filling the blood vessels without generating intravascular pressure. Moreover, stimulation of β_2_-adrenergic receptors decreases venous resistance and increases venous return [331]. Animal studies of uncontrolled haemorrhage have suggested that norepinephrine infusion reduces the amount of fluid resuscitation required to achieve a given arterial pressure target associated with lower blood loss and improved survival [332, 333].

Despite a general paucity of research into the use of vasopressors in hypotensive trauma patients, a double-blind randomised trial has assessed the safety and efficacy of adding vasopressin to resuscitative fluid. Patients were administered fluid alone or fluid plus vasopressin (bolus 4 IU) and i.v. infusion of 200 mL/h (vasopressin 2.4 IU/h) for 5 h. The fluid plus vasopressin group needed a significantly lower total resuscitation fluid volume over 5 days than the control group (*p* = 0.04). The rates of adverse events, organ dysfunction and 30-day mortality were similar [334].

An interim analysis performed during an ongoing multicentre prospective cohort study has suggested that the early use of vasopressors for haemodynamic support after haemorrhagic shock may be deleterious in comparison to aggressive volume resuscitation and should be used cautiously [335]. However, the study was limited in that it was a secondary analysis of a prospective cohort study and not designed to answer the specific hypothesis tested. Moreover, the group receiving vasopressors had a higher rate of thoracotomy. A second study retrospectively analysed the records from 225 patients who received different vasopressor therapies during emergency trauma surgery [336]. Whereas the use of epinephrine was independently associated with increased mortality, there was no difference in the mortality rate compared with other vasopressors. The most recent paper on vasopressor use in massively transfused trauma patients retrospectively analysed 120 trauma patients and described, not surprisingly, an association between the use of vasopressor and mortality [337]. However, on hospital arrival, the patients receiving a vasopressor had a much lower GCS and a higher lactate level and showed a trend toward the transfusion of more blood products.

In conclusion, the effects of vasopressors have not yet been rigorously investigated in humans during haemorrhagic shock and prospective studies to define the effect of vasopressors on patients during haemorrhagic shock are warranted. Current evidence suggests that vasopressors may be useful if used transiently to sustain arterial pressure and maintain tissue perfusion in the face of life-threatening hypotension. However, if used, it is essential to respect the recommended objectives for systolic arterial pressure (80–90 mmHg) in patients without TBI. Because vasopressors may increase cardiac afterload if the infusion rate is excessive or left ventricular function is already impaired, an assessment of cardiac function during the initial ultrasound examination is essential. Cardiac dysfunction could be altered in the trauma patient following cardiac contusion, pericardial effusion or secondary to brain injury with intracranial hypertension [338]. The presence of myocardial dysfunction requires treatment with an inotropic agent such as dobutamine or epinephrine. In the absence of an evaluation of cardiac function or cardiac output monitoring, as is often the case in the early phase of haemorrhagic shock management, cardiac dysfunction must be suspected if there is a poor response to fluid expansion and norepinephrine.

#### Type of fluid

##### Recommendation 15

We recommend that fluid therapy using isotonic crystalloid solutions be initiated in the hypotensive bleeding trauma patient. (Grade 1A)

We recommend the use of balanced electrolyte solutions and the avoidance of saline solutions. (Grade 1B)

We recommend that hypotonic solutions such as Ringer’s lactate be avoided in patients with severe head trauma. (Grade 1B)

We recommend that the use of colloids be restricted due to the adverse effects on haemostasis. (Grade 1C)

#### Rationale

It is widely accepted that during the initial phase of haemorrhagic trauma shock, a restrictive volume strategy be supported with crystalloid solutions. The main reason for this is that all colloid solutions can alter haemostasis. However, if the bleeding is excessive and if crystalloids in combination with vasopressors are not able to maintain basic tissue perfusion, colloid infusions represent a further, however controversial, option to restore perfusion. If a colloid solution is administered, it is still unclear which colloid solution should be used in the initial treatment of the bleeding trauma patient.

In most trauma studies, 0.9% sodium chloride was used as the crystalloid solution. However, at least seven studies in both non-critically and critically ill patients suggest that the use of this crystalloid solution as the main i.v. fluid source results in harm to patients, e.g. reduced renal blood flow velocity and renal cortical tissue perfusion, hyperchloraemic acidosis, increased incidence of kidney injury or even reduced survival [339–347]. In contrast to 0.9% sodium chloride, balanced electrolyte solutions comprise physiological or near-physiological concentrations of chloride and may therefore be advantageous. Similarly, in a retrospective analysis of ICU patients receiving more than 60 mL/kg 0.9% sodium solution over a 24 h period, each 100 mEq increase in chloride load was associated with a 5.5% increase in the risk of death, even after controlling for total fluid volume, age, and severity (*p* = 0.0015) over a 1-year period [344]. The two most recent RCTs comparing balanced crystalloids vs 0.9% sodium chloride, one including 13,347 non-critically ill adults [342] and the other including 15,802 critically ill patients [343], found variation in the negative side-effects of 0.9% sodium chloride, depending on the health status, and thereby on the physiological ability of each patient to compensate. In one of the studies, non-critically ill patients receiving balanced crystalloid solutions compared with saline were shown to have a lower incidence of major kidney-related adverse events within 30 days, without an influence on the length of hospital stay [342]. In the other study, critically ill patients receiving balanced crystalloid solutions compared with saline were shown to have a lower rate of composite outcome death from any cause, new renal replacement therapy or persistent renal dysfunction [343]. In a small prospective randomised trial involving 46 trauma patients, a balanced electrolyte solution improved acid-base status and resulted in less hyperchloraemia at 24 h post-injury compared with 0.9% sodium chloride [346]. Moreover, a secondary analysis demonstrated that the use of balanced electrolyte solutions resulted in a net cost benefit in comparison to the use of 0.9% saline chloride [345]. On the other hand, another recently published study could not exclude the possibility that an acetate-based balanced crystalloid solution increased patient bleeding during cardiac surgery, which warrants further investigation [348]. In conclusion, for critically ill patients such as trauma patients, a balanced electrolyte solution should be favoured over 0.9% sodium chloride, and if a 0.9% sodium chloride solution is used, it should be limited to a maximum of 1–1.5 L.

Hypotonic crystalloid solutions, such as Ringer’s lactate, should be avoided in patients with TBI in order to minimise a fluid shift into the damaged cerebral tissue. A secondary analysis from the Prospective, Observational, Multicenter, Major Trauma Transfusion (PROMMTT) study revealed that Ringer’s lactate solutions were associated with higher adjusted mortality compared with normal saline (HR 1.78; CI 1.04–3.04; *p* = 0.035) [349].

A recent study has suggested that solutions with the potential to restore pH may also be advantageous. It was shown that Ringer’s acetate solution ameliorated splanchnic dysoxia more rapidly, as evidenced by gastric tonometry, than Ringer’s lactate [350]. Whether there are benefits associated with the use of certain isotonic balanced crystalloids with respect to a reduced morbidity or mortality, however, is not clear and remains to be evaluated [339, 344, 347].

Colloid solutions have been used more effectively to restore intravascular volume, as would be expected from basic physiologic concept of fluid exchange across the vasculature. A review of RCTs indicated that colloid solutions can result in lower fluid requirements than crystalloids in all types of patient, including trauma victims, with a ratio of 1.5:1 [351]. A large pragmatic study prospectively comparing colloids to crystalloids reported the same 1.5:1 ratio [352].

Particularly in situations in which there is a need for rapid volume replacement due to severe shock, colloids have often been administered. However, it is still unclear whether colloids really have a beneficial effect on morbidity or mortality. The most recent meta-analysis comparing colloids or crystalloids failed to demonstrate that any colloid reduces morbidity or mortality compared to resuscitation with crystalloids in critically ill or elective surgical patients [353, 354]. The authors concluded that there is no evidence that resuscitation with colloids has any beneficial effect on survival [355]. However, neither the time point of fluid resuscitation nor the duration and dosages of fluid resuscitation have been analysed or openly discussed. Nevertheless, at the present time, good data are lacking to demonstrate the survival benefit of colloids compared with other types of solutions.

Conflicting meta-analyses have shown increased kidney injury and increased mortality in critically ill patients treated with hydroxyethyl starch (HES) solutions [355–357]. On the other hand, it has also been shown that there is no difference in the incidence of death or acute kidney failure in surgical patients receiving HES solutions [358]. It seems doubtful that any conclusions can be drawn from these studies, which were performed mostly under different conditions than are present in the acute hypovolaemic trauma patient. In addition to these conflicting results, an in vitro study using blood from healthy volunteers demonstrated that coagulation and platelet function are impaired by all HES and gelatine solutions [359]. However, gelatine-induced coagulopathy was reversible with the administration of fibrinogen, whereas HES-induced coagulopathy was not. So far, only one small RCT described a benefit for a HES solution in trauma patients. HES (130/0.4) provided a significantly more rapid decline in blood lactate levels and less renal injury than saline solution in penetrating trauma patients [360]. However, because only 42 blunt trauma patients were included in the study, no differences in these parameters could be demonstrated using the different solutions. At present, other colloids, including gelatine solutions, cannot be recommended without restrictions [361].

A number of studies have investigated hypertonic solutions. In 2008, a double-blinded RCT in 209 patients with blunt traumatic injuries analysed the effect of treatment with 250 mL 7.5% hypertonic saline and 6% dextran 70 compared to lactated Ringer’s solution on organ failure [362]. The intention-to-treat analysis demonstrated no significant difference in organ failure and in ARDS-free survival. However, there was improved ARDS-free survival in the subset (19% of the population) requiring 10 U or more of packed RBC [362]. A relatively small clinical trial involving nine patients with intracranial pressure > 20 mmHg found that hypertonic saline reduced intracranial pressure more effectively than dextran solutions with 20% mannitol when compared in equimolar dosing [363]. However, Cooper et al. found almost no difference in neurological function 6 months after TBI in 229 patients who had received pre-hospital hypertonic saline resuscitation compared to conventional fluid [364]. Moreover, two large prospective randomised multicentre studies reported by Bulger and co-workers [365, 366] analysed the effect of out-of-hospital administration of hypertonic fluids on neurological outcome following severe TBI and survival after traumatic hypovolaemic shock. These studies were not able to demonstrate any advantage compared to normal 0.9% saline among the 2184 patients included. In contrast, a recent retrospective analysis in 34 trauma patients demonstrated that hypertonic solutions interfere with coagulation [367]. Two recently published meta-analyses, one including nine trials with 3490 trauma patients and one including 12 trials including 2932 haemorrhagic shock patients, confirmed that there is no beneficial effect of hypertonic saline with or without dextran in general trauma patients [368, 369].

In conclusion, at least during the initial treatment phase and as part of the restricted volume replacement strategy, administration of crystalloids is advocated. The data published to date demonstrate that balanced crystalloid solutions are preferable to 0.9% saline solution, especially if administered in larger amounts. In patients with TBI, hypotonic solutions, crystalloids as well as colloids, should be avoided. If small-volume resuscitation fails to restore the target blood pressure in spite of additional use of norepinephrine, or if extensive volume resuscitation is necessary in the intra-hospital phase of initial trauma management, this can be achieved either with large-volume balanced crystalloid administration or with colloids. Large-volume balanced crystalloid solutions are not independently associated with multiple organ failure [370]. In contrast, a retrospective study showed that resuscitation with at least 1 L crystalloid per unit RBC seems to be associated with reduced overall mortality [371]. However, at present, it is not clear whether colloids should be used if crystalloids fail to restore target blood pressure. Hypertonic saline solutions do not demonstrate any advantage to other less expensive crystalloids. The evidence suggests that hypertonic saline solutions are safe, but will neither improve survival nor improve neurological outcome after TBI.

#### Erythrocytes

##### Recommendation 16

We recommend a target Hb of 70 to 90 g/L. (Grade 1C)

#### Rationale

Oxygen delivery to tissues is the product of blood flow and arterial oxygen content, which is directly related to the Hb concentration; therefore, decreasing Hb might be expected to increase the risk of tissue hypoxia. However, compensatory responses to acute normovolaemic anaemia occur, including macro- and microcirculatory changes in blood flow and capillary recruitment, so the consequences of low Hb in terms of tissue oxygenation are difficult to predict based on macrocirculatory haemodynamic parameters and Hb levels. This has been demonstrated in haemorrhagic shock patients, in whom RBC transfusion was able to improve microcirculation and tissue oxygenation independent of macrocirculation and Hb level [372, 373]. However, the transfusion of RBCs containing methaemoglobin and thus not participating in oxygen delivery also improved microcirculation [372], most likely due to increased blood viscosity [374].

Erythrocytes are oxygen sensors and modulators of vascular tone and microcirculation. Erythrocytes play a fundamental role in matching microvascular oxygen supply with local tissue oxygen demand. Although a number of theories to explain this critical function have been proposed [transport of nitric oxide (NO) in the form of S-nitrosothiol by erythrocyte, deoxyhaemoglobin acting as a nitrite reductase converting nitrite to NO and release of adenosine triphosphate (ATP) from the erythrocyte, resulting in the production of mediators], none has been either universally accepted or fully tested in the intact microcirculation [375]. In addition, erythrocytes may contribute to haemostasis by influencing the biochemical and functional responsiveness of activated platelets through the rheological effect on platelet margination and by supporting thrombin generation [376].

The effects of the Hct level on blood coagulation have not been fully elucidated [377]. An acute reduction of the Hct level results in an increase in the bleeding time [378], with restoration upon re-transfusion [379]. This may relate to the presence of the enzyme elastase on the surface of RBC membranes, which may activate coagulation factor IX [380, 381]. However, an animal model showed that a moderate reduction in Hct level does not increase blood loss from a standard spleen injury [379], and an isolated in vitro reduction of the Hct level did not compromise blood coagulation as assessed using TEG® [382].

RCTs that have evaluated Hb thresholds for transfusion in critically ill patients have consistently found that restrictive transfusion strategies (Hb thresholds between 70 and 90 g/L) are as safe as, or safer than, liberal strategies (thresholds ≥ 90 g/L) [383–387] with the possible exception of patients with acute coronary syndrome. Recently, in high-risk patients undergoing cardiac surgery, a restrictive strategy regarding red cell transfusion was non-inferior to a liberal strategy with respect to the composite outcome of death from any cause, myocardial infarction, stroke or new-onset renal failure with dialysis, with fewer RBCs transfused [388]. These studies excluded patients with massive bleeding and no prospective RCT has compared restrictive and liberal transfusion regimens in trauma patients. A subset of 203 trauma patients from the Transfusion Requirements in Critical Care (TRICC) trial [384] was re-analysed [389]. A restrictive transfusion regimen (Hb transfusion trigger < 70.0 g/L) resulted in fewer transfusions compared with the liberal transfusion regimen (Hb transfusion trigger < 100 g/L) and appeared to be safe. However, no statistically significant benefit in terms of multiple organ failure or post-traumatic infections was observed. It should be emphasised that this study was neither designed nor powered to answer these questions with precision. In addition, it cannot be ruled out that the number of RBC units transfused merely reflects the severity of injury. Nevertheless, RBC transfusions have been shown in multiple studies to be associated with increased mortality [390–394], lung injury [391, 395, 396], increased infection rates [397, 398] and renal failure in trauma victims [394].

Because anaemia is a possible cause of secondary ischaemic damage, concerns have been raised about the safety of restrictive transfusion strategies in the subpopulation of patients with TBI. Most early clinical information comes from retrospective observational studies with important methodological limitations. These data have yielded inconsistent results on the effects of RBC transfusion on markers of cerebral perfusion and metabolism in patients with isolated severe TBI. Two systematic reviews published in 2012 stressed the lack of high-level scientific evidence for a specific Hb transfusion trigger in this setting [399, 400]. A retrospective review of data collected prospectively in 1158 patients with a GCS ≤ 8 in the absence of haemorrhagic shock found that RBC transfusion was associated with worse outcomes (28-day survival, ARDS-free survival, 6-month neurological outcome) when the initial Hb was > 100 g/L [401]. No relationship between RBC transfusion and outcomes was found in patients with an initial Hb ≤ 100 g/L [401]. In a RCT of 200 patients with TBI at two clinical sites, Robertson et al. compared two Hb transfusion thresholds (70 or 100 g/L), and separately compared administration of erythropoietin or placebo [402]. Patients were enrolled within 6 h of injury and 99 patients were assigned to the 70 g/L transfusion threshold and 101 patients to the 100 g/L threshold. Neither the administration of erythropoietin nor maintenance of Hb concentration > 100 g/L resulted in improved neurological outcome at 6 months, and the 100 g/L threshold was associated with a higher incidence of adverse events [402].

Alternative methods of increasing Hb have been studied. The erythropoietic response is blunted in trauma patients [403]; therefore, the administration of erythropoietin appears an attractive option. In a first prospective randomised trial in ICU patients (*n* = 1302, 48% being trauma patients), a significant reduction in RBC transfusion percentage from 60.4 to 50.5% (*p* < 0.001) and reduction in the median number of RBC units transfused from two to one (*p* < 0.001) was observed [404]. In the subgroup of trauma patients, 28-day mortality was also reduced [odds ratio (OR) 0.43 (0.23–0.81)] [404]. In a subsequent prospective randomised trial in ICU patients (*n* = 1460, 54% being trauma patients), no significant reduction in RBC transfusions was observed [405]. Thrombotic complications were higher in erythropoietin-treated patients [HR 1.58 (1.09 to 2.28)]; however, this difference was observed exclusively in patients without heparin prophylaxis [405]. Recently, in a double-blind, placebo-controlled trial undertaken in 29 centres within 24 h of moderate or severe TBI, 606 patients were randomly assigned to receive erythropoietin (40,000 units subcutaneously) or placebo once per week for a maximum of three doses. Erythropoietin did not reduce the number of patients with severe neurological dysfunction (GOS-E level 1–4), the transfusion of RBC or increase the incidence of deep venous thrombosis (DVT) of the lower limbs [406]. Mortality at 6 months tended to be lower in patients treated with erythropoietin (11%) than in control patients with a mortality of 16% (RR 0.68; 95% CI 0·44–1·03; *p* = 0.07) [406]. Interestingly, erythropoietin treatment of critically ill trauma patients resulted in a substantial reduction of mortality (RR 0.63; 0.49–0.79, *p* = 0.0001) in a recent meta-analysis [407].

The limited effect of erythropoietin treatment on transfusion needs may be surprising given the blunted response in trauma patients [403]. However, iron metabolism is also altered after trauma, with iron not being fully available for haematopoiesis [403]. Neither iron metabolism nor availability are fully understood following traumatic injury and complicated by the fact that certain proteins such as ferritin are massively upregulated after trauma as part of the acute phase response [403]. Intravenous iron may therefore represent another attractive option with which to foster haematopoiesis. Indeed, studies that assess the effect of i.v. iron (with [408, 409] or without [410] concomitant epoetin alpha) showed reduced RBC transfusions [408–410], postoperative infections [409, 410], length of hospital stay [409] and mortality in patients with hip fractures [409]. While i.v. iron appears to be promising, oral iron is largely ineffective. However, a recent multicentre, randomised, double-blind, trial during the perioperative period of hip fracture did not find that ferric carboxymaltose with or without erythropoietin induced a reduction of RBC transfusion despite obtaining significant increases in Hb levels at discharge and 60 days after discharge [411]. In a randomised, placebo-controlled, blinded study in anaemic intensive care patients, early administration of low-dose i.v. ferric carboxymaltose, compared with placebo, did not result in a significant lowering of RBC transfusion requirements during hospital stay [412]. Patients who received i.v. iron had a significantly higher Hb concentration at hospital [412].

#### Temperature management

##### Recommendation 17

In order to optimise coagulation, we recommend early application of measures to reduce heat loss and warm the hypothermic patient to achieve and maintain normothermia. (Grade 1C)

#### Rationale

Since coagulopathy following traumatic injury increases mortality [39], it is recommended to target “normothermia”, with a core temperature between 36 and 37 °C in order to create optimal preconditions for coagulation. Hypothermia, a core body temperature <  35 °C, is associated with acidosis, hypotension and coagulopathy in severely affected patients. The effects of hypothermia include altered platelet function, impaired coagulation factor function (a 1 °C drop in temperature is associated with a 10% drop in function), enzyme inhibition and fibrinolysis [413–415]. Body temperatures below 34 °C compromise blood coagulation, but this has only been observed when coagulation tests (PT and APTT) are performed at the low temperatures present in patients with hypothermia, but not when assessed at 37 °C, as is routine practice for such laboratory tests.

The profound clinical effects of hypothermia ultimately lead to higher morbidity and mortality [416], and hypothermic patients require more blood products [417]. In a retrospective study of 604 trauma patients who required massive transfusion, a logistic regression analysis demonstrated that a temperature lower than 34 °C was associated with a greater independent risk of mortality of more than 80% after controlling for differences in shock, coagulopathy, injury severity and transfusion requirements [OR 1.87; 95% CI 1.18–3.0; *p* = 0.007] [418]. A recent study performed a secondary analysis using 10 years of data from the Pennsylvania Trauma Outcome Study (PTOS). It analysed 11,033 patients with severe TBI and demonstrated that spontaneous hypothermia at hospital admission was associated with a significant increase in the risk of mortality [419]. Steps to prevent hypothermia and the risk of hypothermia-induced coagulopathy include removing wet clothing, covering the patient to avoid additional heat loss, increasing the ambient temperature, forced air warming, warm fluid therapy, and, in extreme cases, extracorporeal re-warming devices [420–422]. Recently, the use of a hypothermia prevention and management kit has been advocated [423]. This kit is a low-cost, lightweight, low-volume commercial product that sustains 10 h of continuous dry heat with an oxygen-activated, self-heating liner and provides thermal insulation due to the multi-layer composite construction of the outer shell. The kit was designed to prevent hypothermia during tactical casualty evacuation; however, application in the civilian sector for the active re-warming of trauma patients is conceivable. In comparison to other methods and devices, the hypothermia prevention and management kit achieved and maintained significantly higher temperatures than all other methods and controls at 120 min [424].

### IV. Rapid control of bleeding

#### Damage-control surgery

##### Recommendation 18

We recommend that damage-control surgery be employed in the severely injured patient presenting with deep haemorrhagic shock, signs of ongoing bleeding and coagulopathy. (Grade 1B)

Other factors that should trigger a damage-control approach are hypothermia, acidosis, inaccessible major anatomic injury, a need for time-consuming procedures or concomitant major injury outside the abdomen. (Grade 1C)

We recommend primary definitive surgical management in the haemodynamically stable patient and in the absence of any of the factors above. (Grade 1C)

#### Rationale

The severely injured patient arriving at the hospital with continuing bleeding or deep haemorrhagic shock generally has a poor chance of survival without early control of bleeding, proper resuscitation and blood transfusion. This is particularly true for patients who present with uncontrolled bleeding due to multiple penetrating injuries or patients with major abdominal injury and unstable pelvic fractures with bleeding from fracture sites and retroperitoneal vessels. The final common pathway in these patients is the exhaustion of physiological reserves, with resulting profound acidosis, hypothermia and coagulopathy, also known as the “bloody vicious cycle” or “lethal triad”.

In 1983, Stone et al. described the techniques of abbreviated laparotomy, packing to control haemorrhage and of deferred definitive surgical repair until coagulation has been established [425]. Several articles have since described the beneficial results of this approach, now referred to as “damage control” [426–428]. This approach should be considered in patients with major abdominal injury and a need for adjunctive angioembolisation, major abdominal injury and a need to evaluate other injuries as early as possible, major abdominal injury and traumatic amputation of a limb. Factors that should trigger a damage-control approach in the operating theatre are temperature ≤ 34 °C, pH ≤ 7.2, an inaccessible major venous injury, a need for time-consuming procedures in a patient with suboptimal response to resuscitation or inability to achieve haemostasis due to recalcitrant coagulopathy [429, 430].

Damage-control surgery of the abdomen consists of three components: the first component is an abbreviated resuscitative laparotomy for control of bleeding, the restitution of blood flow where necessary and the control of contamination. This should be achieved as rapidly as possible without spending unnecessary time on traditional organ repairs that can be deferred to a later phase. The abdomen is packed and temporary abdominal closure is performed. Packing aims to compress liver ruptures or exert direct pressure on the sources of bleeding and abdominal packing may permit further attempts to achieve total haemostasis through angiography and/or correction of the “lethal triad”. The removal of packs should preferably be deferred for at least 48 h to lower the risk of re-bleeding.

The second component of damage-control surgery is intensive care treatment, focused on core re-warming, correction of the acid-base imbalance and coagulopathy, as well as optimising the ventilation and the haemodynamic status. If complementary angiography and/or further injury investigation is needed, it should be performed during this phase.

The third component is the definitive surgical repair that is performed only when target parameters have been achieved [133, 426–428, 431–433]. Although the concept of “damage control” intuitively makes sense, no RCTs exist to support it. Retrospective studies support the concept showing reduced morbidity and mortality rates in selective populations [428].

The same “damage control” principles have been applied to orthopaedic injuries in severely injured patients. Scalea et al. were the first to coin the term “damage control orthopaedics” [434]. Relevant fractures are primarily stabilised with external fixators rather than primary definitive osteosynthesis [434–436]. The less traumatic nature and shorter duration of the surgical procedure aims to reduce the secondary procedure-related trauma. Definitive osteosynthesis surgery can be performed after 4–14 days when the patient has recovered sufficiently. Retrospective clinical studies and prospective cohort studies seem to support the concept of damage control. The only available randomised study shows an advantage for this strategy in “borderline” patients [436]. The damage-control concept has also been described for thoracic and neurosurgery [437, 438]. In addition to damage-control surgical approaches, damage-control anaesthesia or resuscitation comprises a number of important measures described in the other recommendations within this document.

#### Pelvic ring closure and stabilisation

##### Recommendation 19

We recommend that patients with pelvic ring disruption in haemorrhagic shock undergo immediate pelvic ring closure and stabilisation. (Grade 1B)

#### Packing, embolisation and surgery

##### Recommendation 20

We recommend that patients with ongoing haemodynamic instability, despite adequate pelvic ring stabilisation, receive early surgical bleeding control and/or pre-peritoneal packing and/or angiographic embolisation. (Grade 1B)

We suggest that the use of aortic balloon occlusion be considered only under extreme circumstances in patients with pelvic fracture in order to gain time until appropriate bleeding control measures can be implemented. (Grade 2C)

#### Rationale

The mortality rate for patients with severe pelvic ring disruptions and haemodynamic instability remains high [439, 440]. There is no consensus as to the optimal treatment paradigm for patients presenting with haemorrhage from severe pelvic fractures. Angioembolisation and an external fixator are the most common approaches. REBOA is considered by some practitioners to be an important adjunct in the treatment of patients with severe pelvic fracture and in shock. However, this method is still in the early stages of development and is not currently used widely across trauma centres [441]. Pelvic ring injuries are associated with a high mortality rate within the first 24 h, due mainly to exsanguinations. Injured patients are managed using a multidisciplinary damage-control strategy. Unstable patients should undergo surgical haemostasis control immediately. Arterial embolisation is an effective means of achieving this and justifies the permanent availability of this approach in level-1 trauma centres. Following CT assessment of injuries, stable patients can undergo arterial embolisation if active arterial bleeding or vascular damage is present. The selective or nonselective embolisation methods and agents used depend on the patient’s haemodynamic status and an assessment of the injury whenever possible [442]. The early detection of these injuries and initial efforts to reduce disruption and stabilise the pelvis as well as containing bleeding is therefore crucial.

Severe pelvic trauma is a particularly challenging condition, requiring a multidisciplinary trauma team, including a general surgeon, orthopaedic surgeon, endovascular surgeon/interventional radiologist. The pelvis can harbour a multifocal haemorrhage that is not easily compressible or managed using traditional surgical methods such as tying off a blood vessel or removing an organ. Treatment often requires a triage of multiple investigations that can lead to the re-approximation of bony structures, including DCR, assessment of associated injuries and multimodal haemorrhage control via external fixation, pre-peritoneal packing, angioembolisation and/or REBOA, for example [71]. Markers of pelvic haemorrhage include anterior-posterior and vertical shear deformations on standard roentgenograms, CT “blush” (active arterial extravasation), bladder compression pressure, pelvic haematoma evident using CT and ongoing haemodynamic instability, despite adequate fracture stabilisation [443–445].

If the patient is haemodynamically unstable and in haemorrhagic shock, the urgent treatment goal is rapid achievement of haemostasis. An initial strategy, performed while DCR is ongoing and before proceeding to arteriography, relies on the insertion of an intra-aortic occlusion balloon and/or extra-peritoneal pelvic packing. If haemodynamic instability persists, a laparotomy for haemostasis should be performed without delay. In a haemodynamically stable patient, contrast-enhanced systematic CT is required to obtain a comprehensive assessment of the lesions prior to surgery [80].

The initial therapy for pelvic fractures includes control of venous and/or cancellous bone bleeding by pelvic closure as a first step [446]. Some institutions use primarily external fixators to control haemorrhage from pelvic fractures [443], but pelvic closure may also be achieved using a pelvic binder, a pelvic C-clamp or improvised methods such as a bed sheet [446, 447]. Based on the available literature, pelvic circumferential compression devices are widely used in the initial management of patients with suspected pelvic bleeding. There is evidence to suggest that external compression reduces disrupted pelvic rings. However, some complications have been reported following the application of pelvic circumferential compression devices. Until this can be clarified, judicious application of pelvic circumferential compression devices will continue to be recommended [448]. In addition to the pelvic closure, fracture stabilisation and the tamponade effect of the haematoma, pre-, extra- or retroperitoneal packing may reduce or control the venous bleeding [449–451]. Pre-peritoneal packing is used to decrease the need for pelvic embolisation and may be performed simultaneously, or soon after, initial pelvic fracture stabilisation. The most commonly embolised vascular bed and therefore the most studied is the pelvis [452]. Pelvic packing could potentially aid in early intra-pelvic bleeding control and provide crucial time for more selective haemorrhage management [449, 451].

Delayed interventions are common in damage-control laparotomy, with abdominal interventions often spread over multiple explorations. In such cases, mortality has been shown to increase in patients undergoing emergent re-exploration, or to delay the repair of major vascular injuries. Ideal treatment of damage-control laparotomy patients may include addressing injuries more completely at the first laparotomy instead of deferring care for other priorities [453].

REBOA has been used in patients with end-stage shock following blunt and penetrating trauma, together with embolisation of the vascular bed in the pelvis. In a military setting with hand-held ultrasound, it has been reported that 7 Fr femoral sheath access ER-REBOA® were positioned and inflated in the aorta without radiography. In all reported cases, REBOA resulted in immediate normalisation of blood pressure and permitted induction of anaesthesia, initiation of whole-blood transfusion, damage-control laparotomy and attainment of surgical haemostasis (range of inflation time 18–65 min). No access- or REBOA-related complications were reported, and all patients survived to achieve transport to the next echelon of care in stable condition. It has been suggested that the use of this device by non-surgeons and surgeons not specially trained in vascular surgery in the non-hospital setting may be useful as a stabilising and damage-control adjunct, allowing time for resuscitation, laparotomy and surgical haemostasis [454]. However, some authors, such as Maruhashi et al. [455], advise the use of REBOA with caution on the basis that it may increase the bleeding of minor thoracic injury in severe multiple trauma patients.

In the case of major pelvic injury, it is nevertheless agreed that damage-control interventional radiology and urgent resuscitative surgery should be initiated early and simultaneously [456]. Adjunct techniques can be combined with a consecutive laparotomy if deemed necessary [451]. This may decrease the high mortality rate observed in patients with major pelvic injuries who have undergone laparotomy as the primary intervention. However, non-therapeutic laparotomy should be avoided [457]. Time to pelvic embolisation for haemodynamically unstable pelvic fractures may impact survival [439, 458].

Angiography and embolisation are currently accepted as highly effective means with which to control arterial bleeding that cannot be controlled by fracture stabilisation [73, 443, 447, 449, 457, 459, 460]. Radiological management can also be usefully applied to abdominal and thoracic bleeding [461–465]. Martinelli et al. [466] reported the use of intra-aortic balloon occlusion to reduce bleeding and permit transport to an angiography theatre. In contrast, Morozumi et al. suggested the use of mobile digital subtraction angiography for arterial embolisation performed in the clinic by trauma surgeons [467]. A number of authors argue that permissive hypotension could achieve better survival by achieving pelvic stabilisation and/or angiography through DCR, hypertonic solutions and controlled hypothermia. Institutional differences in the capacity to perform timely angiography and embolisation may explain the different treatment algorithms suggested by many authors. Reports on transcatheter angiographic embolisation suggest a 100% higher mortality during off-hours due to lack of radiological service [468]. Therefore, a multidisciplinary approach to these severe injuries is required.

#### Local haemostatic measures

##### Recommendation 21

We recommend the use of topical haemostatic agents in combination with other surgical measures or with packing for venous or moderate arterial bleeding associated with parenchymal injuries. (Grade 1B)

#### Rationale

A wide range of local haemostatic agents is currently available for use as adjuncts to traditional surgical techniques to obtain haemorrhagic control. These topical agents can be particularly useful when access to the site of bleeding is difficult. Local haemostatic agents include collagen, gelatine or cellulose-based products, fibrin and synthetic glues or adhesives that can be used for both external and internal bleeding while polysaccharide-based and inorganic haemostatics are still mainly used and approved for external bleeding.

The use of topical haemostatic agents should consider several factors, such as the type of surgical procedure, cost, bleeding severity, coagulation status and each agent’s specific characteristics. Some of these agents should be avoided when auto-transfusion is applied, and several other contraindications need to be considered [469, 470]. The capacity of each agent to control bleeding was initially studied in animals, but increasing experience in humans is now available [469–484].

The different types of local haemostatic agents are briefly presented according to their basis and haemostatic capacity.

- Collagen-based agents trigger platelet aggregation, resulting in clot formation when in contact with a bleeding surface. They are often combined with a procoagulant substance such as thrombin to enhance the haemostatic effect. A positive haemostatic effect has been shown in several human studies [471, 479, 480, 485].
- Gelatine-based products can be used alone or in combination with a procoagulant substance [470]. Swelling of the gelatine in contact with blood reduces the blood flow and, in combination with a thrombin-based component, enhances haemostasis [476, 477, 482]. These products have been successfully used for local bleeding control in brain or thyroid surgery when electrocautery may cause damage to nerves [481] or to control bleeding from irregular surfaces such as during post-sinus surgery [484].
- Absorbable cellulose-based haemostatic agents have been widely used to treat bleeding for many years, and case reports as well as a prospective observational human study support their effectiveness [483]. The oxidised cellulose-based product can be impregnated with polyethylene glycol and other salts and achieve comparable and more rapid haemostasis compared to the combined products described below [475].
- Fibrin and synthetic glues or adhesives have both haemostatic and sealant properties, and their significant effect on haemostasis has been shown in several randomised controlled human studies involving vascular, bone, skin and visceral surgery [472, 474, 478].
- Polysaccharide-based haemostatics can be divided into two broad categories [470]: *N*-acetyl-glucosamine-containing glycosaminoglycans purified from microalgae and diatoms, and microporous polysaccharide haemospheres produced from potato starch. Some minerals, such as kaolin, also seem to have haemostatic effects. The mechanism of action is complex and depends on the purity or combination with other substances such as cellulose or fibrin. A number of different products in the form of pads, patches or bandages are currently available and have been shown to be efficient for external use and for splanchnic bleeding. Observational studies have shown that haemorrhage control is achieved using a poly-*N*-acetyl glucosamine- or kaolin-based bandage applied to patients with severe hepatic and abdominal injuries, acidosis and clinical coagulopathy [480, 486].

### V. Initial management of bleeding and coagulopathy

#### Antifibrinolytic agents

##### Recommendation 22

We recommend that TXA be administered to the trauma patient who is bleeding or at risk of significant haemorrhage as soon as possible and within 3 h after injury at a loading dose of 1 g infused over 10 min, followed by an i.v. infusion of 1 g over 8 h. (Grade 1A)

We recommend that protocols for the management of bleeding patients consider administration of the first dose of TXA en route to the hospital. (Grade 1C)

We recommend that the administration of TXA not await results from a viscoelastic assessment. (Grade 1B)

#### Rationale

Tranexamic acid (trans-4-aminomethyl cyclohexane-1-carboxylic acid, TXA) is a synthetic lysine analogue that is a competitive inhibitor of plasminogen. TXA is distributed throughout all tissues, and the plasma half-life is 120 min [487]. The Clinical Randomisation of Antifibrinolytic therapy in Significant Haemorrhage (CRASH-2) trial [488] assessed the effects of early administration of a short course of TXA on death, vascular occlusive events and the administration of blood product transfusion to trauma patients who were bleeding or at risk of significant bleeding. The trial randomised 20,211 adult trauma patients with or at risk of significant bleeding to either TXA (loading dose 1 g over 10 min followed by infusion of 1 g over 8 h) or matching placebo within 8 h of injury. The primary outcome was in-hospital death within 4 weeks of injury. All analyses assessed the intention-to-treat population. All-cause mortality was significantly reduced with TXA by 1.5%; the risk of death due to bleeding was significantly reduced by 0.8% and a reduction in bleeding deaths by one third, mainly through preventing exsanguination within the first 24 h [489, 490]. Paediatric patients were not included in the CRASH-2 study; however, an ongoing study is investigating the use of TXA in children [491] and a study in children undergoing craniosynostosis surgery [492, 493] administered an initial bolus of 15–30 mg/kg followed by 2−10 mg/kg/h. One retrospective study has suggested that TXA is of no benefit in patients with viscoelastic hyperfibrinolysis [494] and another found TXA to reduce multiple organ failure and mortality in severely injured shocked patients [495]. This discrepancy is probably attributable to methodological limitations.

The risk of thrombosis after the use of the lysine analogues TXA and ε-aminocaproic acid had been of major theoretical concern; however, CRASH-2 showed that the rate of VTE was not altered, while arterial thromboses, especially myocardial infarction, were lower with the use of TXA. TXA use to prevent or manage haemorrhage has been studied in approximately one million patients without increased rates of thrombosis [496–499].

No adverse events were described with the use of TXA in CRASH-2, although an increased rate of seizures has been described in patients undergoing cardiac surgery receiving considerably higher doses of TXA than recommended here [500]. This may reflect the role of fibrinolytic molecules as neurotransmitters.

An unplanned subgroup analysis of the CRASH-2 data [501] showed that early treatment (≤ 1 h from injury) significantly reduced the risk of death due to bleeding by 2.5%. Treatment administered between 1 and 3 h also reduced the risk of death due to bleeding by 1.3%. Treatment given after 3 h increased the risk of death due to bleeding by 1.3%; therefore, we recommend that TXA be administered within 3 h following injury. Further data from over 20,000 patients randomised to TXA versus placebo in post-partum haemorrhage has also shown that benefit is most apparent within the first 3 h. Gayet-Ageron et al. showed that the benefits of TXA were more marked when given as soon as possible after injury and its efficacy decreased by 10% every 15 min from time of injury [502].

In order to ensure that TXA is administered early, TXA administration at the pre-hospital site of injury needs to be planned, and we recommend that protocols for the management of bleeding patients strongly consider administration of the first dose of TXA at the site of injury. El-Menyar et al. looked at the efficacy of pre-hospital TXA in a meta-analysis and showed that it reduced 24-h and 30-day mortality and thromboembolic events. However, the authors found only two studies and concluded that further RCTs are required [503]. This is in keeping with a recent study demonstrating the benefit of on-scene administration of TXA in patients with major trauma. In this study, the normally occurring deterioration of the coagulation status between the site of injury and hospital admission was clearly mitigated by the on-scene TXA administration [504].

If TXA is restricted to massive transfusion protocols or only used in patients clinically judged to be at “high risk”, it is estimated that only 40% of the potential population who would benefit from this treatment will be treated [505]. For all potential patients to receive TXA, TXA should therefore be administered to all patients with trauma and significant bleeding. Thus, TXA should be included as part of each institutional “trauma management protocol” not the “massive blood loss” or “major haemorrhage” protocols. The benefit of on-scene TXA administration has recently been shown to be independent of the severity of injury [504].

Secondly, Moore et al. suggested that TXA be administered only in those patients with hyperfibrinolysis determined using TEG®, as many patients who have traumatic injuries lack a hyperfibrinolytic trace, a so-called “hypofibrinolytic shutdown” [506]. However, Raza et al. have clearly shown that TEG® is poor at detecting fibrinolytic activation when compared with more sensitive assays [507]. Furthermore, support for the unreliability of ROTEM® in detecting hyperfibrinolysis comes from Gall et al., who showed that S100A10, an endothelial receptor for plasminogen, leaches off the endothelium during trauma and interferes with detection of fibrinolysis using ROTEM® [266]. We therefore recommend that TXA be administered as soon as possible, without waiting for viscoelastic results.

The cost-effectiveness of TXA in patients with traumatic injury has been calculated in three countries [508, 509]: Tanzania as an example of a low-income country, India as a middle-income country and the UK as a high-income country. The cost of TXA administration to 1000 patients was US$17,483 in Tanzania, US$19,550 in India and US$30,830 in the UK. The estimated incremental cost of administering TXA per life-year gained was $48, $66 and $64 in Tanzania, India and the UK, respectively.

ε-Aminocaproic acid is also a synthetic lysine analogue that has a potency ten-fold weaker than that of TXA. It is administered at a loading dose of 150 mg/kg, followed by a continuous infusion of 15 mg/kg/h. The initial elimination half-life is 60–75 min and must therefore be administered by continuous infusion in order to maintain therapeutic drug levels until the bleeding risk has diminished. This agent is a potential alternative to TXA if TXA is not available. Due to concerns about safety [510], the use of aprotinin is not advised in bleeding trauma patients, now that TXA has been shown to be efficacious and safe.

#### Coagulation support

##### Recommendation 23

We recommend that monitoring and measures to support coagulation be initiated immediately upon hospital admission. (Grade 1B)

#### Rationale

While several general pathophysiological mechanisms have been described that result in trauma-related coagulopathy, including hyperfibrinolysis [16, 29, 252, 511], it is essential to quickly determine the type and degree of coagulopathy in the individual patient in order to determine the most prominent cause or causes to be treated specifically in a goal-directed manner [512]. Early therapeutic intervention improves coagulation [513], which can reduce the need for transfusion of RBC, FFP and platelets [17, 42, 43, 259, 514], the incidence of post-traumatic multi-organ failure [42] and length of hospital stay [17], as well as improving survival [41, 43, 259, 515, 516]. The success of early algorithm-based and goal-directed coagulation management in reducing transfusions and improving outcomes, including mortality, has also been demonstrated in patients undergoing cardiac surgery [517–519]. It is, therefore, expected that early algorithm-based and goal-directed coagulation management treatment would also improve the outcome of severely injured patients [41–43, 259, 520, 521]. This has indeed been shown in a prospective randomised study [522] and in two studies assessing the introduction of such a concept in two large Italian and one Swiss trauma centre [43, 523]. In other studies, however, no survival benefit could be shown [513, 524, 525]. These variations may be because studies that failed to show an effect tended to base decisions on traditional laboratory values such as PT, APTT and platelet count, and therapies that were often limited to FFP and platelet transfusions.

#### Initial coagulation resuscitation

##### Recommendation 24

In the initial management of patients with expected massive haemorrhage, we recommend one of the two following strategies:

- FFP or pathogen-inactivated FFP in a FFP:RBC ratio of at least 1:2 as needed. (Grade 1C)
- Fibrinogen concentrate and RBC. (Grade 1C)

#### Rationale

The current concept for the resuscitation of patients with massive bleeding with immediate coagulation support was introduced in May 2005, based on reports from the ongoing conflict in Iraq. The US Army’s Institute of Surgical Research recommended the immediate administration of coagulation components in a 1:1:1 ratio for RBC, plasma and platelets until laboratory measurements to adjust therapy were available [526]. In the following years, the best initial strategy to support coagulation became a matter of debate, and two different strategies were proposed. Based on the results of 37 studies, recent guidelines from the Eastern Association for the Surgery of Trauma recommend the transfusion of equal amounts of RBC, plasma and platelets during the early, empiric phase of resuscitation [527]. However, other authors, mainly in Europe, strongly support the use of factor concentrate as the first line of initial coagulation resuscitation in patients with significant bleeding. A few European studies have tried to compare these two strategies; however, there are no good data to date, and no definitive conclusion can be reached. The Reversal of Trauma Induced Coagulopathy Using Coagulation Factor Concentrates or Fresh Frozen Plasma (RETIC) trial, a small single-centre RCT comparing plasma to factor concentrate-based resuscitation, has recently been terminated early after an interim analysis revealed potential harm to patients randomised to the plasma arm [42].

Initial resuscitation is usually defined as the period between arrival in the emergency department and availability of results from coagulation monitoring (coagulation screen, fibrinogen level and/or VEM and platelet count). However, in recent years, studies have focused on the potential advantage of supporting coagulation already in the pre-hospital setting either by plasma transfusion (pre-thawed [528, 529] or freeze-dried [530, 531]) or by administration of fibrinogen [532].

Many authors agree that early and aggressive plasma transfusion reduces mortality [533]. A prospective multicentre study that included a large population of patients undergoing massive transfusion showed that high FFP:RBC and platelet:RBC ratios are associated with a survival benefit, also when time-dependency is accounted for [317]. However, the optimal FFP:RBC and platelet:RBC ratio remained controversial. The Pragmatic, Randomized Optimal Platelet and Plasma Ratios (PROPPR) trial, a randomised clinical trial in 680 trauma patients who were suspected to have sustained or had experienced massive blood loss [534] reported that there was no difference in overall survival between early administration of plasma, platelets and red blood cells in a 1:1:1 ratio (FFP:platelets:RBC) compared with 1:1:2. However, more patients in the 1:1:1 group achieved “anatomic” haemostasis and fewer experienced death due to exsanguination by 24 h. The early use of platelets and a high level of FFP use in the 1:1:1 group was not associated with a significantly increased rate of complications.

FFP transfusion is not free of risk. Complications associated with FFP treatment include transfusion-associated circulatory overload (TACO), ABO blood group incompatibility, transmission of infectious diseases (including prion diseases) and mild allergic reactions. Transfusion-related acute lung injury (TRALI) [535] is a severe adverse effect associated with the presence of leucocyte antibodies in transfused plasma. The risk of TRALI has been greatly reduced by avoiding the use of plasma from women with a history of pregnancy. Transmission of infectious diseases can be minimised by the use of pathogen-inactivated plasma (industrial purified plasma).

Further controversy concerns the use of plasma to correct the decreased fibrinogen levels associated with haemorrhagic shock. Haemostasis is critically dependent on fibrinogen as a substrate for clot formation and the ligand for platelet aggregation. Fibrinogen is the single coagulation factor that is affected more and earlier in association with trauma-induced coagulopathy. Many bleeding trauma patients with trauma-induced coagulopathy present with a fibrinogen depletion, below levels currently recommended for therapeutic supplementation. Schlimp et al. [226] demonstrated that levels of fibrinogen lower than 1.5 g/L are detected in as many as 73% of patients with an admission Hb lower than 100 g/L and in 63% of those with a base excess (BE) lower than − 6. Moreover, Rourke et al. [536] found low fibrinogen in 41% of the patients who were hypotensive on admission. In this study, hypotension, increasing shock severity and a high degree of injury (ISS ≥ 25), were all associated with a reduction in fibrinogen levels.

Although plasma contains all coagulation factors, administration of plasma to bleeding patients brings no consistent correction of any measure of clot function and may dilute fibrinogen levels, but cannot contribute to a significant increase [12]. Moreover resuscitation with a large amount of plasma is associated with dilution of RBC and platelets [12]. Several authors, mainly in Europe, strongly disagree with the initial transfusion of patients based on an empirical ratio rather than guided by concurrent laboratory data (goal-directed therapy) [537]. Only in the absence of rapid near-patient coagulation testing to facilitate goal-directed therapy may initial treatment with blood components in a fixed ratio constitute a reasonable approach. If concurrent coagulation results are available, they should be used to guide therapy.

Initial fibrinogen levels below the normal range are independently associated with higher in-hospital mortality [538] and survival improves with fibrinogen administration [539]. Unless pre-thawed plasma is available [540], plasma transfusion cannot be initiated at the same time as universal RBC transfusion and significant delays have been reported in achieving the targeted plasma:RBC ratio [541]. During this interval, the fibrinogen level is likely to be lower than desired. Fibrinogen concentrate is widely used in Europe to rapidly restore fibrinogen levels. For very initial coagulation support, while waiting for the results of viscoelastic or laboratory tests, it has been proposed to administer 2 g of fibrinogen to mimic the expected 1:1 ratio corresponding to the first four units of RBC and potentially correct hypofibrinogenaemia, if already present [523]. Experimental data show that administration of fibrinogen does not suppress endogenous fibrinogen synthesis [542].

### VI. Further goal-directed coagulation management

#### Goal-directed therapy

##### Recommendation 25

We recommend that resuscitation measures be continued using a goal-directed strategy, guided by standard laboratory coagulation values and/or VEM. (Grade 1B)

#### Rationale

There is increasing interest in the use of goal-directed strategies guided by either POC VEM [543–549] or CCAs [522, 550] to augment DCR during the acute care of bleeding trauma patients [551–554]. A recent survey among surgeons and anaesthesiologists in Germany revealed that 90% used CCA to guide decision-making in the diagnosis and treatment of bleeding trauma patients, whereas 56% reported that they also used extended VEM such as ROTEM® or TEG® [555], and this predominantly in advanced trauma centres [556]. POC VEM may provide more rapid information about the underlying haemostatic deficiencies, including information on which part of the clotting process is disrupted, as well as on the dynamics of clot formation and lysis [557, 558], thereby allowing optimised and targeted coagulation therapy according to individual deficits, particularly with respect to the early use of coagulation factor concentrates (CFC) [544, 547, 559].

A recent retrospective military study, involving 134 patients requiring transfusion over 6 months, compared transfusion practices before and after incorporation of ROTEM® measurement into DCR protocols at a US Role-3 hospital in Afghanistan. The study showed an improved adherence to DCR practices after the introduction of ROTEM®, suggesting that DCR without viscoelastic data may result in reduced haemostatic support and underestimate the need for platelet and fibrinogen administration [553]. Goal-directed administration of fibrinogen concentrate and other coagulation factors (e.g. PCC) in a retrospective observational civilian study resulted in significant improvements in fibrin polymerisation as measured by an increase in ROTEM®-FIBTEM maximum clot firmness (MCF) and a normalisation in ROTEM®-EXTEM (EXTEM, extrinsically activated test) clotting times below the upper threshold [560] and a reduction in transfusion needs [561].

VEM are highly specific for hyperfibrinolysis, which is considered the much more lethal and resource-intense phenotype of fibrinolysis compared with shutdown [562], and these tests should be used during early trauma resuscitation to identify injured patients with systemic hyperfibrinolysis [544]. Recent clinical and experimental work suggests that antifibrinolytic therapy should be employed in acutely injured patients and optimally guided by ROTEM® or TEG® [563]. To date, a number of algorithms including treatment thresholds have been proposed for both ROTEM® [544, 546, 564] and TEG® [565, 566]; however, these are based largely upon retrospective data or expert opinion [33, 273, 544, 565–567]. ROTEM® and TEG® assays show similar clinical performance; however, results are not interchangeable, arguably due to different coagulation triggers, different coagulation activators and reagents [567]. Of further note, the initial correlation between CCA, for example INR, and ROTEM® parameters such as EXTEM clotting time at admission, may decrease over time, possibly due to injury severity, base deficit or the administration of blood products, particularly fibrinogen concentrate, during therapy [568].

The first Cochrane review on the diagnostic accuracy of ROTEM® and TEG® for trauma-induced coagulopathy in adult trauma patients with bleeding during the period between 1970 and 2013 identified three studies, but found no evidence on the accuracy of TEG® and only very limited evidence on the accuracy of ROTEM®, suggesting that these tests be reserved for research use only [268]. An updated Cochrane review from 2016 on the use of ROTEM® and TEG® to monitor and guide haemostatic treatment and transfusion versus usual care in adults and children with bleeding included 15 studies, but was not limited to trauma patients and included only two trials judged to be at low risk of bias [569]. Compared with transfusion guided by any method, ROTEM® or TEG® appeared to reduce overall mortality (7.4% vs 3.9%; RR 0.52, 95% CI 0.28–0.95; *I*^2^ = 0%, 8 studies, 717 participants); however, only eight trials provided data on mortality, and two were zero-event trials. A statistically significant effect of ROTEM® or TEG® was observed relative to the proportion of participants transfused with pooled RBC (RR 0.86, 95% CI 0.79–0.94; *I*^2^ = 0%, 10 studies, 832 participants), FFP (RR 0.57, 95% CI 0.33–0.96; *I*^2^ = 86%, 8 studies, 761 participants) or platelets (RR 0.73, 95% CI 0.60–0.88; *I*^2^ = 0%, 10 studies, 832 participants), as well as overall haemostatic transfusion with FFP or platelets. Meta-analyses also showed fewer participants with dialysis-dependent renal failure. A recent meta-analysis systematically reviewed and assessed 15 RCTs (including 1238 patients) performed with patients in acute need of blood transfusion due to bleeding to evaluate the effect of viscoelastic test guidance on bleeding, transfusion requirements and mortality. While only one trial included trauma patients, this study confirmed the transfusion-saving effect associated with ROTEM® and TEG® guidance, including reduced bleeding volume [570]. A systematic literature update of the 2011 Australian National Blood Authority patient blood management guidelines for critical bleeding confirmed substantial evidence gaps, in particular with regard to the effect of component therapies, including the ratio of RBCs to component therapies [571]. Overall, viscoelastic test-based restrictive transfusion management may prevent unnecessary plasma and platelet transfusion, thereby reducing the risk of transfusion-related adverse events and transfusion-associated hospital costs [572, 573].

Curry and co-workers have calculated that a 4 g dose of fibrinogen concentrate is equal to two pools of cryoprecipitate via ex vivo ROTEM® spiking data and results in a clinically meaningful increase in clot strength reflected by ROTEM®-EXTEM and ROTEM®-FIBTEM clot firmness [574]. The authors used this dose of cryoprecipitate in their prospective, randomised multicentre study in the UK to assess the feasibility of administering cryoprecipitate within 90 min of hospital admission in bleeding trauma patients. Eighty-five percent (95% CI 69–100%) of patients received cryoprecipitate in addition to standard treatment within 90 min of hospital admission. Fibrinogen concentrations were maintained under supplementation above 1.8 g/L at all time-points during active haemorrhage, while 28-day mortality showed a non-significant trend towards reduced mortality with early fibrinogen supplementation [cryoprecipitate, 2 (10%) vs standard, 6 (28.6%)].

Early goal-directed haemostatic resuscitation of trauma-induced coagulopathy was further explored recently in a single-centre, pragmatic prospective RCT in the USA that tested whether a massive transfusion protocol goal-directed by TEG® could improve survival compared with a massive transfusion protocol guided by CCA [259]. One hundred and eleven patients were included in the intent-to-treat analysis (TEG®, *n* = 56; CCA, *n* = 55). Survival in the TEG® group was significantly higher than the CCA group (log-rank *p* = 0.032, Wilcoxon *p* = 0.027); there were 20 deaths in the CCA group (36.4%) compared with 11 in the TEG® group (19.6%) (*p* = 0.049). Most deaths occurred within the first 6 h after arrival (21.8% CCA group vs 7.1% TEG® group) (*p* = 0.032). CCA patients required a similar number of RBC units as the TEG® patients [CCA, 5.0 (2–11); TEG® 4.5 (2–8)] (*p* = 0.317), but more plasma [CCA, 2.0 (0–4); TEG®, 0.0 (0–3)] (*p* = 0.022) and more platelet units [CCA, 0.0 (0–1); TEG®, 0.0 (0–0)] (*p* = 0.041) during the first 2 h of resuscitation. This was the first prospective RCT that demonstrated that utilisation of a goal-directed, TEG®-guided massive transfusion protocol to resuscitate severely injured patients improves survival compared with a protocol guided by CCA and utilises less plasma and platelet transfusions during the early phase of resuscitation.

The RETIC study using first-line CFC or FFP in bleeding trauma patients or patients presumed to bleed was conducted as a single-centre, parallel group, open-label, randomised trial at a level-1 trauma centre in Austria. In the study, trauma patients with a coagulopathy identified by abnormal fibrin polymerisation or prolonged clotting time using ROTEM® received either FFP (15 mL/kg of bodyweight) or CFC (primarily fibrinogen concentrate [50 mg/kg of bodyweight]) [42]. The study was terminated early, for futility and safety reasons, with 100 patients allocated (FFP, *n* = 48 and CFC, *n* = 52) due to the high proportion of patients in the FFP group who required rescue therapy compared with those in the CFC group (23 [52%] in the FFP group vs 2 [4%] in the CFC group; OR 25.34 [95% CI 5.47–240.03], *p* < 0.0001) and an increased need for massive transfusion in the FFP group (13 [30%] in the FFP group vs 6 [12%] in the CFC group; OR 3.04 [0.95–10.87], *p* = 0·042). The interim analysis for the predefined endpoint upon premature study termination showed multiple organ failure in 29 (66%) patients in the FFP group and in 25 (50%) patients in the CFC group (OR 1.92 [95% CI 0.78–4.86], *p* = 0.15).

In another RCT, Nascimento and co-workers assessed the effects of a transfusion strategy guided by laboratory results versus a fixed-ratio (1:1:1) transfusion protocol in patients with severe trauma [522]. At a single centre, 78 patients were randomly assigned to either a transfusion protocol guided by laboratory results (*n* = 38) or the fixed-ratio (1:1:1) transfusion protocol (*n* = 40). Plasma wastage was higher with the fixed-ratio protocol (22% [86/390] of FFP units vs 10% [30/289]). In the intention-to-treat analysis, all-cause 28-day mortality was 5/35 (14.3%) in the laboratory-result-guided transfusion group versus 13/40 (32.5%) in the group that was treated according to the fixed-ratio 1:1:1 protocol [RR 2.27 (95% CI 0.98–9.63)]. The introduction of a novel standard operating procedure (SOP) using a Hb/CCA-oriented and coagulation-factor-based algorithm for the early correction of trauma-induced coagulopathy in patients requiring a massive transfusion was retrospectively assessed using a pre- and post-implementation approach at a single centre in Germany [550]. The main objective was the effect on transfusion requirements and the standardised mortality ratio (SMR), which is the ratio of observed deaths to expected/predicted deaths. Eighty-seven patients were assessed. The SMR decreased from 0.95 before to 0.72 after SOP implementation, which was not statistically significant (*p* = 0.16) due to the small sample size, but was considered clinically relevant. However, a significant reduction in the requirement of RBC transfusions (22.8 ± 8.1 units vs 17.6 ± 7.6 units) was observed (*p* = 0.003) as well as a faster correction of laboratory coagulopathy upon ICU admission for fibrinogen and Quick value and a clear trend to better results for INR and PTT.

The introduction of an early coagulation support protocol, including POC testing, which replaced the former high plasma:RBC ratio strategy in two Italian trauma centres was associated with a marked reduction in blood-product consumption, reaching statistical significance for plasma (65%) and platelets (52%), and with a non-significant trend toward a reduction in early and 28-day mortality [523]. The overall costs for transfusion and coagulation support, including POC tests, decreased by €76,340 (23%) after early coagulation support protocol implementation in 2013. Whiting and co-workers assessed the clinical effectiveness and cost-effectiveness of viscoelastic test devices to assist with the diagnosis, management and monitoring of haemostasis disorders during and after a variety of bleeding entities, including trauma-induced coagulopathy [558], based upon an extended search of sixteen databases up to December 2013. Of note, only a few trauma studies could be retrieved. Nevertheless, apart from the well-known reduction in RBC, platelet and FFP transfusion in the groups that used viscoelastic test devices and the absence of differences in clinical outcomes, the use of viscoelastic testing was associated with cost-savings and more effective than CCAs. For the trauma population, the cost-savings owing to viscoelastic testing devices were more substantial, amounting to per-patient savings of £688 for ROTEM® and £721 for TEG® compared with CCA. This finding was entirely dependent on material costs, which are slightly higher for ROTEM®.

#### Fresh frozen plasma-based management

##### Recommendation 26

If a FFP-based coagulation resuscitation strategy is used, we recommend that further use of FFP be guided by standard laboratory coagulation screening parameters (PT and/or APTT > 1.5 times normal and/or viscoelastic evidence of a coagulation factor deficiency). (Grade 1C)

We recommend that FFP transfusion be avoided in patients without major bleeding. (Grade 1B)

We recommend that the use of FFP be avoided for the treatment of hypofibrinogenaemia. (Grade 1C)

#### Rationale

Plasma (thawed FFP or pathogen-inactivated plasma) has been used for many years and throughout the world as a source of coagulation factors, physiological anticoagulants and other haemostatic factors. FFP contains > 70% the normal level of all clotting factors. Preclinical studies have shown the protective and regenerative effects of plasma on haemorrhage-induced glycocalyx disruption [575] and endothelial damage. Retrospective studies [576] and the PROPPR study have suggested that early transfusion of plasma in a balanced ratio of 1:1 to 1:2 is associated with lower mortality in patients with critical haemorrhage, although the optimal ratio has not yet been established [534].

Plasma transfusions, however, are not free of risk and in patients without substantial bleeding, the risk of TACO, multiple organ dysfunction syndrome (MODS), ARDS and infections may exceed the potential benefits [577, 578]. Moreover, a recent retrospective study supported FFP transfusion as an independent risk factor for increased mortality or worse outcomes across a spectrum of surgical risk profiles including TBI [579].

Different plasma preparations show great variability. FFP contains a variable amount of fibrinogen and is associated with significant risk of allergic reactions and TRALI [580]. Pathogen-inactivated plasma has a more standardised content of fibrinogen and minimises the risk of TRALI and exogenous infection compared with FFP [581].

Frozen plasma products must be thawed in preparation for transfusion and this time-consuming process may delay plasma transfusion. The use of readily transfusable thawed liquid plasma has been shown to allow a higher plasma:RBC ratio within the first hour of transfusion, thus potentially increasing its efficacy in preventing coagulopathy. However, liquid plasma is only available in a few high-volume trauma centres [540]. With a relative shortage of type AB plasma, to allow plasma transfusion for resuscitation of patients whose blood type is unknown, the use of type A plasma has been proposed [582–584]. Preliminary data show that transfusion of incompatible type A plasma to patients with blood groups B and AB as part of a massive transfusion protocol does not appear to be associated with significant increases in morbidity or mortality [585]. Freeze-dried plasma use has been recently implemented in the military setting [530] as well as in civilian pre-hospital care [531] and might help to reduce the time needed to start plasma transfusion.

Although plasma transfusion may support coagulation, Khan and Brohi observed that there was no consistent correction of any measure of clot function nor any large increase in the procoagulant factor level when FFP was delivered during the acute phase of ongoing bleeding [12, 586]. Moreover resuscitation with large amounts of plasma is associated with dilution of RBC and platelets [539]. Anaemia may further contribute to a reduction in platelet marginalisation, with a potentially negative impact on platelet activation.

We recommend the use of FFP if a plasma-based coagulation strategy is applied and there is evidence of coagulation factor deficiency as evidenced by a PT and/or APTT > 1.5 times the normal control. A prolongation of “clotting time” or “reaction time” using VEM may also be considered as an indication for the administration of FFP; however, the scientific evidence for this is scarce and a normalisation of fibrinogen level as described in R28 will often normalise these parameters.

#### Coagulation factor concentrate-based management

##### Recommendation 27

If a CFC-based strategy is used, we recommend treatment with factor concentrates based on standard laboratory coagulation parameters and/or viscoelastic evidence of a functional coagulation factor deficiency. (Grade 1C)

Provided that fibrinogen levels are normal, we suggest that PCC is administered to the bleeding patient based on evidence of delayed coagulation initiation using VEM. (Grade 2C)

We suggest that monitoring of FXIII be included in coagulation support algorithms and that FXIII be supplemented in bleeding patients with a functional FXIII deficiency. (Grade 2C)

#### Rationale

Traumatic coagulopathy is characterised by an increased fibrinolytic activity and a low fibrinogen concentration [8, 22, 23, 29, 226, 266, 536, 587–589]. Besides early administration of TXA (see recommendation R22) early fibrinogen administration is also of key importance, ideally guided by a fibrinogen concentration < 1.5 g/L or viscoelastic evidence of a functional fibrinogen deficiency [41–43, 590, 591]. Exogenous sources of fibrinogen comprise FFP, cryoprecipitate and fibrinogen concentrate [592]. Because the fibrinogen concentration in FFP is highly variable and often relatively low, administration may further dilute the in vivo fibrinogen level, and FFP administration is also associated with adverse outcomes [395, 593]. Therefore, most trauma centres administer cryoprecipitate or fibrinogen concentrate to treat low fibrinogen levels. An individualised CFC-based strategy targets the specific needs of each individual patient based on standard laboratory coagulation parameters and/or viscoelastic evidence of a functional coagulation factor deficiency [41–43, 590].

Injury severity is often inversely correlated with low fibrinogen levels at hospital admission [23, 226, 594, 595]. Rugeri et al. described a median fibrinogen level of 0.9 g/L [interquartile range (IQR) 0.5–1.5 g/L] in trauma patients with coagulopathy [29]. Schöchl and co-workers found approximately 50% of patients with an admission fibrinogen level < 1.5 g/L [587]. Rourke and colleagues reported 40% of hypotensive trauma patients with a fibrinogen concentration < 1.5 g/L [536] and Schlimp et al. reported that 54% of patients with a Hb < 120 g/L also had a fibrinogen concentration < 1.5 g/L, increasing to approximately 75% with an admission Hb < 100 g/L or progressively negative BE [226]. An additional recent study describes median fibrinogen levels of 0.9 g/L (IQR 0.6–1.2 g/L) [588].

Fibrinogen concentrations are variable among different FFP preparations [511], influenced by donation altitude [596] and dependent on the type of pathogen inactivation method applied [597–599]. Final fibrinogen concentrations range from 1.0 to 3.0 g/L, most often approximately 2 g/L for non-pathogen-inactivated FFP [592] and below 2 g/L for pathogen-inactivated FFP [597, 598]. With such a low fibrinogen concentration, FFP administration will not rapidly increase fibrinogen levels in the bleeding trauma patient; indeed, in reality, fibrinogen concentration decreased significantly after the transfusion of 4 units of FFP in trauma patients [600]. In addition, the use of FFP has been associated with increased multi-organ failure [593] and TRALI [395, 593] in trauma patients. Finally, transfusion of FFP inevitably results in a decrease in Hb concentration that may trigger RBC transfusion, which will dilute the coagulation potential of the blood further, thus aggravating traumatic coagulopathy [601, 602]. Cryoprecipitate or fibrinogen concentrate are therefore preferred by many trauma specialists for the treatment of low fibrinogen levels. The application of individualised CFC-based strategies result in favourable clinical outcomes, including reduced mortality [41–43, 590, 601, 602]. Fibrinogen concentrate has been adopted by the Canadian Armed Forces as part of a remote DCR strategy in Special Operating Forces operating in austere environments [603].

The usefulness of PCC has been demonstrated, with evidence of reduced haematoma formation in patients with head injury [604, 605], and is preferable to FFP for the rapid reversal of the effects of VKAs [606–609] (for further details, see recommendation R33). Thromboelastometry (TEM) is useful to guide individualised goal-directed coagulation therapy in patients with traumatic coagulopathy [17, 560, 610, 611]. In the initial phase, a low fibrinogen concentration is expected [8, 22, 23, 29, 226, 266, 536, 587–589]. However, thrombin generation is preserved or even increased [612, 613]. Initial treatment should therefore comprise fibrinogen administration, which not only increases the MCF in FIBTEM, but also shortens the clotting time in EXTEM [560]. Only if the EXTEM clotting time remains prolonged, despite a fibrinogen level > 1.5 g/L should PCC be administered to normalise the EXTEM clotting time [516, 614].

It is important to avoid the overly liberal use of PCC in trauma patients, because PCC administration results in increased thrombin potential over days that is not reflected by standard laboratory tests and might expose the trauma patient to an increased risk of delayed thrombotic complications [613]. Therefore, the risk of thrombotic complications resulting from PCC treatment should be weighed against the need for rapid and effective correction of coagulopathy [615–620]. While the incidence of thrombotic complications mirrors that of patients treated with FFP [621, 622], safety data on the use of PCC in trauma patients are scarce beyond the emergency reversal of pre-treatment with a VKA [623]. A higher incidence of thromboembolic events has been reported in trauma patients with the use of three-factor PCC compared to four-factor PCC [624]. According to some expert opinion, activated PCC (aPCC) may be associated with a higher risk of thrombosis compared to non-activated PCC [625] due to the presence of activated factor IX, because the thrombogenic trigger associated with PCC infusion occurs at the level of factor X activation as a part of aPCC [626]. Therefore, in patients who have received PCC, the application of thromboprophylactic measures as early as possible after bleeding has been controlled is prudent.

Coagulation factor XIII (FXIII), formerly known as a “fibrin stabilising factor”, is a transglutaminase circulating in tetrameric form consisting of two A and two B subunits. The A subunit of FXIII is activated to FXIIIa by thrombin and FXIIIa catalyses the cross-linking of fibrin. [627] Strong cross-linking of fibrin prevents fibrinolysis [628] and FXIII activity seems to be an important independent modulator of clot firmness [592, 629].

In the neurosurgical setting, a postoperative FXIII level < 60% was found to be an independent risk factor for postoperative intracranial bleeding in an observational study of 876 patients [630] and in another study with 1264 neurosurgical patients [631]. In patients undergoing cardiac surgery, low FXIII levels were also associated with increased postoperative blood loss [632, 633].

FXIII is present in different concentrations in cryoprecipitate, FFP and FXIII concentrate [592, 627]. Recombinant FXIII-A_2_ (rFXIII-A_2_) has been developed for the prophylaxis and treatment of bleeding in patients with inherited FXIII A-subunit deficiency [634]. Levy et al. reported a preliminary study of rFXIII-A_2_ in cardiac surgery. Patients scheduled for coronary artery bypass grafting were randomly assigned to receive a single dose of rFXIII-A_2_ or placebo following cardiopulmonary bypass after an initial dose of protamine. Treatment with rFXIII-A_2_ restored levels of FXIII to preoperative levels was well tolerated but did not reduce the need for RBC transfusion [635]. Efficacy and safety of the prophylactic use of rFXIII-A_2_ on blood transfusions were also evaluated in an RCT in adult patients undergoing cardiac surgery, but no reduction in transfusion requirements was found [636].

There are no specific RCTs evaluating FXIII levels and/or FXIII replacement therapy in trauma patients. However, acquired low levels of FXIII have been found in patients with major trauma and coagulopathy [637]. If cryoprecipitate is not available, as in most European countries, and a CFC-based strategy is used, very little if any factor XIII is administered. Monitoring factor XIII levels and replacement below a certain threshold therefore is suggested as part of coagulation support algorithms. At present, however, the need for and a defined optimal level of FXIII replacement in major trauma patients has not been determined. The updated guidelines for the management of severe perioperative bleeding from ESA suggests the administration of FXIII concentrate in the presence of bleeding and a FXIII level < 30% [638]. The use of FXIII concentrate at a FXIII level < 60% was part of multimodal algorithms in two recent studies in major trauma patients, resulting in major reductions in transfusion requirements and improvements in clinical outcomes, including a reduction in the duration of stay in the ICU, organ dysfunction and hospital mortality in one study [42, 43].

#### Fibrinogen supplementation

##### Recommendation 28

We recommend treatment with fibrinogen concentrate or cryoprecipitate if major bleeding is accompanied by hypofibrinogenaemia (viscoelastic signs of a functional fibrinogen deficit or a plasma Clauss fibrinogen level ≤ 1.5 g/L). (Grade 1C)

We suggest an initial fibrinogen supplementation of 3–4 g. This is equivalent to 15–20 single-donor units of cryoprecipitate or 3–4 g fibrinogen concentrate. Repeat doses should be guided by VEM and laboratory assessment of fibrinogen levels. (Grade 2C)

#### Rationale

Fibrinogen is the final component in the coagulation cascade, the ligand for platelet aggregation and therefore key to effective coagulation and platelet function [377, 639]. Hypofibrinogenaemia is a common component of the coagulopathy associated with massive bleeding [640], fibrinogen is the first coagulation factor to fall below critical levels [641] and hypofibrinogenaemia is associated with increased mortality [642]. There are no fibrinogen reserves outside the plasma, which means that a sharp fall in fibrinogen level cannot be quickly compensated. However, plasma fibrinogen levels do increase with age and atherosclerosis and behave as an acute phase protein. Interestingly, in accord with this, Ohmori et al. found that, unlike young people, those > 65 years, had higher fibrinogen levels following major blood loss, suggesting that fibrinogen level is a poor early indicator of blood loss in the older population [643]. Schlimp et al. and others have demonstrated that fibrinogen levels upon admission strongly correlate with rapidly obtainable routine laboratory parameters such as Hb and BE [226, 589]. Fibrinogen levels lower than 1.5 g/L were detected in as many as 73% of trauma patients with an admission Hb lower than 100 g/L and in 63% of those with a BE lower than − 6 [644]. Rourke et al. [536] observed low fibrinogen levels in 41% of hypotensive patients on admission.

Coagulopathic civilian trauma patients had a median fibrinogen concentration of 0.9 g/L (IQR 0.5–1.5 g/L) in conjunction with a maximum fibrinogen thromboelastometric MCF of 6 mm (IQR 0–9 mm) using TEM, whereas only 2.5% of healthy volunteers had a MCF of < 7 mm [29]. In trauma patients, a MCF of 7 mm was associated with a fibrinogen level of approximately 1.5–2.0 g/L [587]. During postpartum haemorrhage, fibrinogen plasma concentration is the only coagulation parameter independently associated with progress towards severe bleeding, with a level < 2 g/L having a PPV of 100% [645].

Our recommendation to supplement fibrinogen in patients with traumatic bleeding when levels fall below 1.5 g/L is supported by other international guidelines [591]. The required fibrinogen dosage may be estimated based on the results of thromboelastometric monitoring using a simple formula. The administration of 0.5 g fibrinogen to an 80 kg patient may increase the A10 MCF by 1 mm, the application of which may facilitate a rapid and predictable increase in plasma fibrinogen to a target level [602].

There are methodological issues with all of the techniques applied to measure fibrinogen concentration [646, 647]. The Clauss method is the gold standard laboratory assay; however, in the presence of artificial colloids such as HES this method may overestimate the actual fibrinogen concentration [647]. Functional fibrinogen measurement using TEM is also influenced by Hct [648] and FXIII levels [649].

Three questions remain unanswered with respect to the use of supplementary fibrinogen. First, it is not clear whether use, especially early use, of fibrinogen reduces mortality. An early observational study suggested that fibrinogen substitution can improve survival in combat-related trauma [650]. In the civilian setting, the use of TEM-guided fibrinogen replacement reduced exposure to allogeneic blood products [17, 516, 523]. Retrospective reviews of single-centre experiences in the management of massive blood loss in trauma patients have also suggested a reduced mortality when compared with expected mortality [516] and increased 30-day survival [651]. Data from an open-label single-centre RCT that investigated the use of factor concentrates versus FFP suggested that early use of fibrinogen was more effective than early use of FFP, as fewer patients required rescue therapy [42].

In contrast, a retrospective analysis of those who received cryoprecipitate versus those who did not showed no improved outcome, although cryoprecipitate was only administered to patients with fibrinogen < 1.0 g/L and this group showed no improved clinical outcome [652]. The retrospective Military Application of Tranexamic Acid in Trauma Emergency Resuscitation (MATTERs II) study of massive military bleeding suggested that cryoprecipitate may independently add to the survival benefit of TXA in the seriously injured patient who requires transfusion [653]. However, cryoprecipitate is often administered with great delay. In the PROMMTT study [654], the median time from admission to the first cryoprecipitate unit was 2.8 h (IQR 1.7–4.5), and in the Activation of Coagulation and Inflammation in Trauma (ACIT) study [586], cryoprecipitate was administered only after the first six units of blood.

As yet, there are still no adequately powered prospective clinical trials to demonstrate the risk–benefit relationship associated with administration of additional fibrinogen from other sources to manage bleeding trauma patients [264, 655]. A small randomised, controlled feasibility trial suggested that the early administration of cryoprecipitate in trauma patients is possible and 85% of recipients received cryoprecipitate within 90 min. The same group have organised an ongoing multicentre randomised trial known as CRYOSTAT II, which will investigate the effect of early cryoprecipitate on clinical outcome [574].

Second, the effect of fibrinogen supplement use on the rate of post-traumatic VTE has never been systematically addressed. Post-traumatic fibrinogen levels are expected to rise as part of the acute phase response after major surgery and trauma [613, 656–658], even without intraoperative fibrinogen administration. Interestingly, intraoperative administration of fibrinogen concentrate in trauma patients [613] or in patients undergoing cardiac surgery resulted in higher intra- and early postoperative fibrinogen levels, but fibrinogen levels were identical on postoperative days 1–7 in patients with and without intraoperative fibrinogen administration [658, 659].

Finally, no studies to date have adequately addressed whether cryoprecipitate and fibrinogen concentrates show similar efficacy and safety profiles; therefore, there is insufficient evidence to support a firm statement about which of the two strategies is best, or whether a combined used of both strategies could be beneficial [660].

The rationale for fibrinogen administration should be read in conjunction with that for FFP (R26).

#### Platelets

##### Recommendation 29

We recommend that platelets be administered to maintain a platelet count above 50 × 10^9^/L. (Grade 1C)

We suggest maintenance of a platelet count above 100 × 10^9^/L in patients with ongoing bleeding and/or TBI. (Grade 2C)

If administered, we suggest an initial dose of four to eight single platelet units or one aphaeresis pack. (Grade 2C)

#### Rationale

Although platelets play a pivotal role in haemostasis after injury, the threshold and timing of platelet transfusion in trauma patients is controversial. In initial acute blood loss, the bone marrow and spleen variably release platelets into the circulation, and therefore their decrease in the peripheral blood is delayed. As a result, platelet counts are typically within the normal range (150 × 10^9^/L to 400 × 10^9^/L) during early traumatic coagulopathy [661, 662]. Upon admission, platelet count < 150 × 10^9^/L has been reported in only 4% of trauma patients with an ISS of 5 and in 18% of patients with ISS > 5 [661]. Platelet counts decreased markedly in the 2 h following hospital admission and 1 × 10^9^/L/h over the next 22 h, suggesting an important role for the treatment administered [662]. A platelet count of 50 × 10^9^/L may be anticipated when approximately two blood volumes have been replaced by fluid or red cell components [641].

However, platelet count on admission may be predictive of outcome, as documented in some cohorts of massively transfused trauma patients, in which platelet count was inversely correlated with injury severity [661], morbidity [663] and mortality [661]. A low or decreasing platelet count also predicts greater mortality in trauma patients [662] and a lower than normal platelet count predicts progression of ICH [663–665], need for neurosurgical intervention [664] and mortality after TBI [663–665].

Although the platelet count may have a predictive value for outcome, platelet transfusion to increase the number of platelets has contradictory effects. Proactive administration of platelets in patients with massive bleeding due to ruptured aortic abdominal aneurysms increased survival from 30 to 45% when the platelet count was > 50 × 10^9^/L as compared with < 50 × 10^9^/L and further increased to 69% for those with platelet count > 100 × 10^9^/L [666]. In contrast, platelet transfusion did not restore the platelet count in trauma patients in one study [667] and did not influence the outcome in patients with TBI and moderate thrombocytopenia (50–107 × 10^9^/L) [668]. Accordingly, at this time, there is weak scientific evidence to support a particular platelet count threshold for platelet transfusion in the severe bleeding trauma patient [669, 670].

The fact that the association between lower platelet counts and higher mortality applies to platelet counts well into the normal range [662] suggests that platelet dysfunction may play a role [285]. Moderate or even mildly decreased platelet aggregation has been shown to be strongly associated with mortality [283, 284, 671]. However, platelet transfusion did not improve platelet dysfunction in general trauma [667] or TBI patients [308]. Repeated measurements after platelet transfusion demonstrated that non-responders to transfusion had a trend towards higher mortality compared with patients with increased platelet function after transfusion [289]. Notably, platelets may attenuate fibrinolysis by providing an additional source of plasminogen activator inhibitor-1, which may be beneficial during the early treatment of traumatic coagulopathy [667]. As platelet dysfunction may be present after injury, even before substantial fluid or blood products have been administered, and continues during the resuscitation period, there is a potential role for early platelet transfusion in the management of traumatic coagulopathy [285].

Early, up-front administration or higher doses of platelets given in predefined ratios with other blood products in trauma patients with massive bleeding who are not yet thrombocytopenic is controversial. Although most of the combat [672, 673] and civilian studies [651, 674–676], one meta-analysis [677] and one systematic review [678] that investigated the impact of platelet transfusion in severe trauma and massive transfusion, showed an improved survival rate among patients receiving high platelet:RBC ratios, such evidence provided by retrospective and observational studies may be subject to serious confounding factors, such as survivorship bias [677] or co-interventions [679]. The timing of platelet transfusion relative to the initiation of RBC and FFP transfusion was not reported in most of the studies, and there may be more than one optimal ratio depending on trauma severity, degree and dynamics of blood loss and previous fluid administration [677]. Considering that significant dilution of platelet count resulted when a 1:1:1 ratio of RBC, plasma and platelets was administered [539], the actual number of platelets transfused to each patient is unknown because blood bank standards estimate only the minimum number of platelets contained in apheresis and pooled platelet units [678].

One additional reason for the lack of clarity on the role of platelet transfusion is the difficulty in separating the effect of a high platelet:RBC ratio from the effect of a high plasma:RBC ratio in most observational studies. In comparison with increased plasma:RBC ratios, the impact exerted by platelets on survival was not as strong [680, 681], higher than the impact of high amount of plasma [651, 673] or even absent [682, 683]. In patients with TBI, transfusion of a high platelet:RBC ratio and a low plasma:RBC ratio was found to be associated with improved survival [684]. Interestingly, patients with penetrating injuries [681] and females [680] do not benefit from high platelet:RBC ratios, and no difference in mortality was observed in patients with non-massive transfusion receiving higher platelet:RBC ratios [685].

However, large prospective cohort studies showed that a high platelet:RBC ratio was associated with survival benefit as early as 6 h after admission, suggesting that survivor bias is unlikely [576, 679]. Significant protective association between higher platelet ratios and mortality was concentrated during the first 6 h only, in contrast to high plasma ratios, which were protective throughout the first 24 h [576]. A recent observational study confirms that a high platelet:RBC ratio (≥ 1:1.5) within 4 h post-injury but not later (4–24 h) is significantly associated with a lower rate of multiple organ failure and mortality within 30 days post-injury, although with higher rates of wound infection and pneumonia [686].

A large multicentre RCT (PROPPR), designed to evaluate the benefit of blood product ratios (1:1:1 or 1:1:2 FFP:platelets:RBC) on patient outcome indicated no survival benefit at 24 h or 30 days of empiric immediate (within minutes of arrival to a trauma centre) platelet transfusion [534]. Of note, the intervention in this trial differed both in the ratio of FFP and platelets, but also the order of administration, with the 1:1:1 group receiving platelets as the first product and the 1:1:2 not receiving platelets until the second batch of blood product was provided, after six RBC and three FFP units had been administered. More patients in the 1:1:1 group achieved haemostasis and fewer experienced death as a result of exsanguination at 24 h. However, these outcomes were not pre-specified outcomes of the trial and had the analysis not combined exsanguination with other causes of death, exsanguination alone would not have emerged as a significant cause of death [687]. Unfortunately, this study did not independently examine the effects of plasma and platelets on outcomes.

A systematic review of six RCTs, five in trauma patients, on the optimal dose, timing and ratio of blood products for massive transfusion, found no evidence of a mortality or morbidity benefit using higher ratios. The authors concluded that there is insufficient evidence to recommend a 1:1:1 plasma and platelet to RBC ratio over a 1:1:2 ratio or standard care [688].

The discrepancies between observational and randomised trials on the benefit of early administration of high doses of platelets are not fully explained. Interestingly, trauma patients receiving massive transfusion, specifically platelet transfusions, showed a further reduction in platelet count, with a disproportionate decrease in function that was donor-related [300], putting the quality of the platelet product administered in question [689].

A theoretical shortcoming of ratio-driven resuscitation is over-transfusion with plasma and platelets, resulting in no benefit or in added morbidity such as multiple organ failure [578, 675]. Recent observations suggest that both early FFP (0–6 h) and delayed (7–24 h) platelet transfusions are risk factors for hypoxaemia and ARDS after 24 h, respectively [690], and non-massively transfused blunt trauma patients receiving > 250 mL platelets were more likely to develop ARDS [691]. A recent prospective multicentre cohort study concluded that in critically ill patients, transfusion of platelets, but not of RBC and plasma, is an independent risk factor for acquiring a nosocomial infection [692]. The introduction of pathogen-reduction technologies may abrogate many of the adverse effects associated with pathogen contamination of platelet products, but with a risk of decreased platelet transfusion effectiveness [693, 694].

The storage of transfused platelets may also play a role in the effectiveness of platelet transfusion. After adjustment for confounders, patients receiving aphaeresis platelets stored for 5 days had a 2.4-fold higher risk of developing complications, including acute renal failure, ARDS and sepsis, than patients transfused with fresher platelets [695]. Although cold-stored platelets may have a reduced circulating life, they have superior haemostatic effects, compared with room-temperature platelets, and therefore potential benefits in the treatment of critical traumatic bleeding [696].

The therapeutic dose of platelets is one aphaeresis platelet product, which is approximately equivalent to four to six units of pooled platelets, and contains approximately 3–4 × 10^11^ platelets [669, 670]. The platelet-rich plasma used in the USA contains fewer platelets than the high-output platelet concentrate manufactured by apheresis or pooling five buffy coats mainly used in Europe [697]. This difference should be considered when analysing the results of studies supporting higher levels of platelet transfusion. Furthermore, the buffy coat method has been shown to contain higher quantities of platelet-derived micro-particles versus leuko-reduced platelets from apheresis or platelet-rich plasma preparation. Even with apheresis-derived platelet products, metabolites accumulating during storage may have an impact on platelet recovery and survival, which could impact clinical outcomes [689].

A dose of four to eight platelet units or a single-donor aphaeresis unit is usually sufficient to provide haemostasis in a thrombocytopenic bleeding patient and should increase the platelet count by 30–50 × 10^9^/L. However, the usual 60–70% recovery rate in peripheral blood may be lower under conditions associated with increased platelet consumption [697]. Although ABO-identical, or at least ABO-compatible platelets are recommended in order to provide a good yield, it is acceptable to use ABO incompatible platelets to reduce waste [669].

#### Calcium

##### Recommendation 30

We recommend that ionised calcium levels be monitored and maintained within the normal range during massive transfusion. (Grade 1C)

We suggest the administration of calcium chloride to correct hypocalcaemia. (Grade 2C)

#### Rationale

The normal concentration of the ionised form of calcium ranges from 1.1–1.3 mmol/L and is influenced by the pH; a 0.1 unit increase in pH decreases the ionised calcium concentration by approximately 0.05 mmol/L [698]. Ionised calcium plays an essential role in the formation and stabilisation of fibrin polymerisation sites; therefore, a reduction in the concentration of calcium has an impact on all platelet-related functions [698]. In addition, contractility of the heart and systemic vascular resistance are low in the presence of reduced ionised calcium levels. Laboratory tests may not reflect the negative impact of hypocalcemia on the clotting process, as blood samples are recalcified before being analysed.

Acute hypocalcaemia is a common complication of massive transfusion [699] and low ionised calcium levels at admission are associated with increased mortality [700]. In patients receiving blood transfusions, hypocalcaemia results from the citrate chelation of serum Ca^2+^. Each unit of packed RBC and FFP contains approximately 3 g of citrate used as a preservative and anticoagulant. Normally, the liver metabolises and clears citrate in a matter of minutes. However, in patients who are in haemorrhagic shock and require massive transfusion, liver function is often impaired due to hypoperfusion. Hypocalcaemia in critically ill patients requiring massive transfusion is detrimental, because Ca^2+^ plays a crucial role in normal coagulation. Ca^2+^ is a cofactor in the activation of factor II, VII, IX, and X, along with protein C and protein S of the coagulation cascade, and it also contributes to platelet adhesion at the site of vessel injury. Hypocalcaemia during the first 24 h can predict mortality and the need for multiple transfusions better than the lowest fibrinogen concentrations, acidosis and the lowest platelet counts [701]. Measurement of ionised calcium levels can easily be performed together with a blood gas analysis by the majority of blood gas analysers available on the market.

A retrospective study on patients who received massive transfusion and who developed hypocalcaemia (Ca^2+^ < 1.12) showed that severe hypocalcaemia (Ca^2+^ < 0.90) was associated with a significantly higher APTT, higher blood lactate levels, lower platelet count and lower blood pH compared with moderate hypocalcaemia (Ca^2+^ ≥ 0.90). Patients in the Ca^2+^ < 0.90 group received more blood products (34 vs 22 units) and mortality was significantly higher (49% vs 24%) [702].

Transfusion-induced hypocalcaemia with ionised Ca^2+^ levels below 0.9 mmol/L or serum total corrected calcium levels of 7.5 mg/dL or less requires prompt calcium replacement, as ionised Ca^2+^ levels below 0.8 mmol/L are associated with cardiac dysrhythmias. The ionised calcium concentration should therefore be maintained within the normal range. However, no data are available to demonstrate that the prevention of ionised hypocalcaemia reduces mortality among patients with critical bleeding who require massive transfusion.

To correct hypocalcaemia, calcium chloride is preferred to calcium gluconate, as 10% calcium chloride contains 270 mg of elemental calcium per 10 mL, whereas 10% calcium gluconate contains 90 mg of elemental calcium per 10 mL [703]. Calcium chloride may also be preferable to calcium gluconate in the presence of abnormal liver function, because decreased citrate metabolism results in slower release of ionised calcium.

#### Recombinant activated coagulation factor VII

##### Recommendation 31

We do not recommend the use of recombinant activated coagulation factor VII (rFVIIa) as first-line treatment. (Grade 1B)

We suggest that the off-label use of rFVIIa be considered only if major bleeding and traumatic coagulopathy persist despite all other attempts to control bleeding and best-practice use of conventional haemostatic measures. (Grade 2C)

#### Rationale

rFVIIa acts on the endogenous coagulation system, but depends on adequate numbers of platelets and fibrinogen to support effective activity [704, 705]. Following trauma, pH and body temperature should be restored as near to physiological levels as possible, since even small reductions in pH and temperature result in slower coagulation enzyme kinetics [413, 415, 706]. This is of particular importance because thrombin generation is typically normal in patients following major trauma [613]. Predictors of a poor response to rFVIIa are pH < 7.2 (*p* < 0.0001), platelet count < 100 × 10^9^/L (*p* = 0.046) and blood pressure ≤ 90 mmHg (*p* < 0.0001) [707]. Initial research based on data from the Australian and New Zealand Haemostasis Registry suggests that administration of rFVIIa in patients with blood pH < 6.9 is of no value [708]. Subsequent research showed that pH < 7.1 prior to rFVIIa administration was independently associated with an increased 28 day mortality [709–711].

rFVIIa should be considered only if treatment with a combination of surgical approaches, best-practice use of blood products, the use of antifibrinolytics and correction of severe acidosis, severe hypothermia and hypocalcaemia fail to control bleeding. Best-practice use of blood products includes RBC, platelets, FFP and cryoprecipitate/fibrinogen resulting in a Hct above 24%, platelets above 50 × 10^9^/ L and fibrinogen above 1.5–2.0 g/L. A recent French cohort study that included patients with severe blunt or penetrating trauma treated with rFVIIa for persistent massive bleeding showed that adherence to the above criteria prior to the administration of rFVIIa was associated with lower mortality and fewer transfusions [712].

Despite a large number of case studies and series reporting that treatment with rFVIIa is beneficial in the treatment of bleeding following trauma, there have been only a limited number of high-quality studies supporting these claims [713–715]. In a multicentre, randomised, double-blind, placebo-controlled study the efficacy of high-dose rFVIIa in patients with blunt (*n* = 143) or penetrating (*n* = 134) trauma was reported. After receiving eight units of RBC, patients assigned to the rFVIIa arm received 200 μg/kg initially with a second and third rFVIIa dose of 100 μg/mg 1 and 3 h later. Patients with blunt trauma, who survived for more than 48 h and were assigned to receive rFVIIa, were shown to require fewer RBC transfusions and fewer massive transfusions (> 20 units of RBC) compared with placebo. In contrast, there were no significant effects in the penetrating trauma patients, although trends towards reduced RBC requirements and fewer massive transfusions were observed [716]. Similar results and trends were observed in other retrospective studies and case reports [717–719]. A further randomised clinical trial aimed to evaluate rFVIIa as an adjunct to direct haemostasis in major trauma patients who bled four to eight RBC units within 12 h of injury and were still bleeding despite strict DCR and operative management. Patients were treated with rFVIIa (200 μg/kg initially; 100 μg/kg at 1 and 3 h) or placebo. The trial was terminated early (*n* = 573) due to challenges in obtaining informed consent and enrolling more severely injured patients, resulting in low mortality rates that prompted a futility analysis. Thrombotic adverse events were similar across study cohorts [720]. A recent Cochrane meta-analysis concluded that the efficacy of rFVIIa outside its current licensed indications is unproven and even associated with an increased incidence of arterial thromboses. Therefore, rFVIIa should only be used for licensed indications or in the context of a study [721]. Similarly, another meta-analysis found more arterial thromboses in rFVIIa-treated patients [722].

Indeed, the use of rFVIIa to treat traumatic coagulopathy represents an “off-label” indication and administration may increase the risk of thromboembolic complications [723]. A meta-analysis showed a higher risk of arterial thromboembolic adverse events (5.6% in patients receiving rFVIIa versus 3.0% in placebo-treated patients) among over 2000 patients enrolled in placebo-controlled trials outside currently approved indications in various clinical settings [724]. This result, however, was contradicted in a study of trauma patients, in which rFVIIa use was not associated with an increased risk of thromboembolic complications [725]. A higher dose of rFVIIa (100 μg/kg) was not associated with greater incidence of thromboembolic events when compared to lower dose (30 μg/kg) in a retrospective single-centre cohort study that analysed 152 surgical and trauma patients. However, a higher incidence of thromboembolic events (approximately 21%) was observed in patients undergoing cardiothoracic surgery and suffering penetrating trauma [726].

In patients with isolated head injury and traumatic ICH, the use of rFVIIa was shown to have no positive effect on patient outcomes, and even found to be harmful [727, 728]. Current evidence does not support the use of any haemostatic drugs, including rFVIIa, for reducing mortality or disability in patients with TBI and related ICH [729].

### VII. Reversal of antithrombotic agents

#### Antithrombotic agent reversal

##### Recommendation 32

We recommend reversal of the effect of antithrombotic agents in patients with ongoing bleeding. (Grade 1C)

1. VKAs
2. Direct oral anticoagulants—FXa inhibitor
3. Direct oral anticoagulants—Thrombin inhibitor
4. Antiplatelet agents

#### Rationale

Reversal of the different anticoagulants is considered in separate chapters in this guideline, as the strategy and mechanism behind the reversal of each is different. We wish to remind the reader that patients take antithrombotic medication because they have an underlying thrombotic risk. The need for reversal thus needs to be weighed against the prothrombotic state of the individual. For example, a patient with an old-fashioned prosthetic mitral valve will have a high risk of thrombosis once anticoagulation is counteracted; therefore, full reversal of the anticoagulant is only justified in presence of life-threatening bleeding.

Once the antithrombotic agent has been reversed then the patient is at risk of thrombosis, due to the absence of anticoagulation, to the acute phase response that occurs following trauma and possibly to prothrombotic effects of the reversing agent. Appropriate thromboprophylaxis should therefore be initiated as soon as possible after bleeding has been controlled.

#### Reversal of vitamin K-dependent oral anticoagulants

##### Recommendation 33

In the bleeding trauma patient, we recommend the emergency reversal of vitamin K-dependent oral anticoagulants with the early use of both PCC and 5 mg i.v. phytomenadione (vitamin K_1_). (Grade 1A)

#### Rationale

Coumarin (more accurately, 4-hydroxycoumarin) derivatives are VKAs and are still widely used, despite the growing use of DOACs. Warfarin is the most commonly used VKA in the world, and the coumarins have a similar effect but a shorter (acenocoumarol) or longer (phenprocoumon) half-life [730].The most common use for VKAs is the prevention of stroke in patients with atrial fibrillation. Other indications include prevention of thrombosis in those with previous venous or arterial thromboembolism or with mechanical heart valves. There are three therapeutic options for the reversal of VKAs such as warfarin: vitamin K, PCC and FFP.

The biochemical reversal of VKA can be achieved rapidly with the administration of PCC [607, 731]. All modern guidelines on warfarin use advise the rapid restoration of a normal INR, although evidence that this reduces intracranial haematoma growth in those with ICH or improves clinical outcome is limited to case series. [604, 732–735], one suggesting more improvement if PCC was administered rapidly [734].

For immediate reversal of VKAs, the missing coagulation factors, FII, FIX and FX, can be replaced with PCC [736]. However, in the past, there has been significant variability in the FVII content of different formulations and three-factor PCC has very little FVII. Modern PCC formulations contain significant amounts of FVII and can completely reverse the effect of VKAs upon infusion. Unfortunately, some countries only have access to three-factor PCC, which achieves poor correction of the INR, and is therefore not recommended if four-factor PCC is available. Because the half-life of administered FVII is only about 6 h, it is important that phytomenadione (vitamin K_1_) is administered with PCC to stimulate physiological generation of the vitamin K-dependent coagulation factors after this time [736].

The alternative to PCC is FFP, which contains the missing coagulation factors diluted among all the other constituents of plasma. However, large volumes of FFP are required, reversal is not always achieved and there are risks of TACO and TRALI [736]. A recent systematic review and meta-analysis of 19 studies (18 cohort and one RCT) that included 2878 patients demonstrated that PCC provides more rapid and complete factor replacement [OR 0.64 (95% CI 0.27–1.5) for PCC versus FFP] [731]. In addition, thromboembolic complications were observed in fewer PCC recipients (2.5%) than FFP recipients (6.4%). However, similar poor clinical outcomes were seen in both groups [731].

Four-factor PCC is administered intravenously in a dose of 25–50 U/kg, and there are algorithms available with which to calculate the most appropriate dose based on bodyweight and INR level [736]. A stepwise dosage is recommended, e.g. 25 U/kg if INR is 2–4.0, 35 U/kg if INR is 4–6.0 and 50 U/kg if INR is > 6.0 [737].

Vitamin K should also be administered intravenously. After reversal, it is important to check INR regularly for the next week, as a minority of patients require over a week to clear warfarin from their blood and thus may require additional vitamin K [738]. A rare and unpredictable but important side effect of i.v. vitamin K is an anaphylactic reaction, in some cases resulting in cardiac arrest, with an incidence of 3 per 100,000 doses via a non-immunoglobulin E (IgE) mechanism, possibly due to the solubiliser in the vitamin K solution [739].

“Overcorrection” of warfarin reversal with additional PCC and vitamin K_1_ can lead to harm. More than 10 mg vitamin K_1_ can prevent re-warfarinisation for days and overuse of PCC (administration of further PCC when INR is in the normal range) may create a prothrombotic state, which could lead to further thrombosis [736].

The use of PCC is associated with an increased risk of both venous and arterial thrombosis during the recovery period, which is related to preexisting risk and possibly the use of PCC [736]. A higher incidence of thromboembolic events has been reported in trauma patients with the use of three-factor PCC compared with four-factor PCC [624]. Therefore, in patients who have received PCC, thromboprophylaxis is prudent as early as possible after bleeding has been controlled.

#### Direct oral anticoagulants—factor Xa inhibitors

##### Recommendation 34

We suggest the measurement of plasma levels of oral direct anti-factor Xa agents such as apixaban, edoxaban or rivaroxaban in patients treated or suspected of being treated with one of these agents. (Grade 2C)

We suggest that measurement of anti-Xa activity be calibrated for the specific agent. If measurement is not possible or available, we suggest that advice from an expert haematologist be sought. (Grade 2C)

If bleeding is life-threatening, we suggest administration of TXA 15 mg/kg (or 1 g) intravenously and that the use of PCC (25–50 U/kg) be considered until specific antidotes are available. (Grade 2C)

#### Direct oral anticoagulants—direct thrombin inhibitors

##### Recommendation 35

We suggest the measurement of dabigatran plasma levels using diluted thrombin time in patients treated or suspected of being treated with dabigatran. (Grade 2C)

If measurement is not possible or available, we suggest measurement of the standard thrombin time to allow a qualitative estimation of the presence of dabigatran. (Grade 2C)

If bleeding is life-threatening in those receiving dabigatran, we recommend treatment with idarucizumab (5 g intravenously) (Grade 1B) and suggest treatment with TXA 15 mg/kg (or 1 g) intravenously. (Grade 2C)

#### Rationale

In recent years, DOACs have been approved for the treatment and prevention of VTE, prevention of stroke in atrial fibrillation and acute coronary syndrome. These anticoagulants function primarily as direct factor Xa inhibitors (apixaban, edoxaban, rivaroxaban) or as a thrombin inhibitor (dabigatran) [740]. These agents account for the majority of DOAC prescriptions in many countries, and there is limited but increasing clinical experience with trauma patients treated with one of these drugs [741–747]. Reassuringly, most studies have shown that patients on DOAC do not suffer from excessive general or intra-cerebral bleeding or higher mortality. It should be emphasised, however, that most of these studies included elderly patients (median age 67–85 years) suffering minimal blunt trauma, ranging from falls (without a formal ISS evaluation) to median ISS values of a maximum of 9 points. Moreover, comparison has been made with patients on VKAs, who also exhibit a higher mortality after trauma than non-anticoagulated patients [741]. In a single study that described a standard trauma population (median age 42 years), a similar mortality in dabigatran- and VKA-treated patients (13.9%) was observed for each group, which was higher than in those taking aspirin or clopidogrel in the control groups (6–8%) [741].

The DOAC plasma concentration is the most important factor that determines whether an active reversal of medication is necessary. Increasing DOAC plasma levels have been shown to progressively affect laboratory [748] and viscoelastic coagulation tests [257, 749–751] and produce increased bleeding volume in laboratory animals with standardised injuries [752]. Early assessment of both laboratory coagulation tests and direct measurements of DOAC levels, therefore, is crucial in trauma patients receiving or suspected of having received DOAC [753]. It is important to remain highly cognizant that any presenting patient may be anticoagulated, since most severely injured patients are too unwell to relay this information to the treating emergency physician. Measurement using three generally available laboratory tests, PT, anti-factor Xa and thrombin time, allows for the assessment of whether a patient is anticoagulated, and if so, by which agent, VKA, a FXa inhibitor or a thrombin inhibitor, respectively. Viscoelastic coagulation tests may also be helpful, since DOAC, but not therapeutic levels of VKA, prolong the clotting time (ROTEM®) and reaction (R) time (TEG®) progressively, along with increasing DOAC plasma concentration. However, if the patient has a trauma-induced coagulopathy, it will not be possible to discriminate between the effects of each possible prior treatment [257, 749–751].

If anti-factor Xa activity has been detected, PCC (25–50 U/kg) treatment may be initiated. We suggest an initial dose of 25 U/kg, repeated if necessary, as a cautious approach given the possible thrombotic potential of PCC products [620]. The co-administration 15 mg/kg (or 1 g) of TXA is indicated in trauma patients (see R22). In May 2018, the US Food and Drug Administration (FDA) approved andexanet alpha [754] as an antidote for the urgent reversal of rivaroxaban and apixaban. However, such approval by the European Medicines Agency (EMA) is lacking for the time being.

Whether PCC treatment results in improved haemostasis with reduced bleeding may depend on the level of anti-factor Xa activity. No effect on bleeding was seen in rabbits with a rivaroxaban plasma concentration of approximately 500–700 ng/mL [755]. In rats, progressive doses of four-factor PCC, however, reduced the volume bled. In these experiments, bleeding volume was normalised in animals with a rivaroxaban plasma concentration of approximately 150 ng/mL by administering a PCC dose of 25 U/kg. At a higher rivaroxaban plasma concentration of approximately 280 ng/mL, normalisation of bleeding required a PCC dose of 50 U/kg. However, at a rivaroxaban plasma concentration of approximately 480 ng/mL, even the administration of 100 U/kg PCC was unable to reduce the elevated blood loss [752].

In the presence of life-threatening bleeding and anti-FIIa activity due to dabigatran, treatment with idarucizumab (5 g i.v.) should be initiated [756, 757]. Because the effect of idarucizumab is short-lived, repeated doses may be necessary [758]. Once idarucizumab has been administered, it is also important to repeat all coagulation tests (laboratory and viscoelastic tests) within 5–10 min, because these tests are affected by dabigatran. Hence, only after dabigatran neutralisation are they able to show the underlying trauma-induced coagulopathy usually present in patients following major trauma [759].

#### Antiplatelet agents

##### Recommendation 36

We suggest treatment with platelet concentrates if platelet dysfunction is documented in a patient with continued bleeding who has been treated with APA. (Grade 2C)

We suggest administration of platelets in patients with ICH who have been treated with APA and will undergo surgery. (Grade 2B)

We suggest that the administration of platelets in patients with ICH who have been treated with APA and will not undergo surgical intervention be avoided. (Grade 2B)

We suggest that the administration of desmopressin (0.3 μg/kg) be considered in patients treated with platelet-inhibiting drugs or von Willebrand disease. (Grade 2C)

#### Rationale

Conflicting data exist about the effects of APA on traumatic bleeding. Patients with ongoing antithrombotic treatment admitted for any bleeding event to the emergency department [760], as well as general trauma patients without brain injury [761], did not show a significant increase in mortality risk. In elderly patients (≥ 65 years of age) with severe trauma and pre-injury anticoagulants and APA, only the warfarin group had a significantly higher risk of bleeding [762], but in other studies pre-injury APA usage was significantly associated with massive transfusion [763] and haemostatic treatments within 24 h [764], but without an impact on survival. Prior use of APA was also a risk factor for the development of complications in blunt chest trauma [765]. In contrast, geriatric (60 to 80 years) traumatic fall patients on clopidogrel, but not on other APA (aspirin or dypiridamole) or anticoagulants, had a higher mortality, length of hospital stay and complication rate compared with controls [766].

Data from non-elective orthopaedic procedures are also divergent. Aspirin was associated with increased need for postoperative blood transfusion and higher all-cause mortality during the first year after hip fracture surgery [767], but did not independently affect morbidity and mortality in patients with pelvic fractures, despite the increase in RBC transfusion [768]. Two meta-analyses of case series with controls [769, 770], and one systematic review [771], found a similar risk of bleeding or transfusion volume in patients with hip fracture surgery on pre-injury clopidogrel, compared with those not taking the agent at the time of surgery performed within 48 h. The authors recommend usual protocols with early surgery in all patients, and although there may have been a small increase (5%) in the proportion of patients requiring transfusion [769], mortality was not increased [769]. Further studies documented the safety (no increase in mortality or complications) of early operation for hip fracture on patients taking clopidogrel [772, 773]. However, a higher drop in Hb is expected in those on dual APA therapy [773, 774]. In contrast, in another study, patients who underwent surgery for intertrochanteric fracture while on clopidogrel had a poorer prognosis compared with controls: intraoperative blood transfusion, ICU and hospital stays and 1-year mortality were higher, despite having the same rate of postoperative complications [775].

The discrepant results in the above studies may reflect the differences in patient- and region-related factors, perioperative continuation or short (48–72 h) interruption of the APA and the lack of a confounding factor analysis. The possible explanations for the low risk of bleeding in patients receiving clopidogrel found in some studies include individual responsiveness to the agent and interactions with other drugs, such as proton pump inhibitors, administered perioperatively [771].

The role of pre-injury APA treatment in the genesis of ICH in patients with blunt head trauma is also controversial. A meta-analysis of cohort and case-control studies found that APA (mainly clopidogrel) use was associated with an increased risk of ICH in patients with head injury and the association was most relevant in patients with mild TBI [776], thus supporting the addition of the APA-use criterion to improve the out-of-hospital identification of older adults requiring trauma-centre care [777]. The risk of subdural haematoma is particularly marked for the combined treatment of clopidogrel with a VKA [778]. However, due to the limited literature, the association with traumatic ICH could not be established in patients receiving aspirin monotherapy [776]. Interestingly, aspirin and not clopidogrel use was significantly associated with intracranial bleeding in older patients (> 60 years) admitted after ground level fall in one study [779]. In contrast, a prospective study performed in head-injured older adults transported by Emergency Medicine Services in 11 US hospitals found no difference in the incidence of ICH between those with or without pre-injury anticoagulant or APA use [780] and a longer period of observation for delayed ICH in patients on APA or anticoagulant medication is not supported [781]. Another prospective cohort study showed a low incidence of traumatic ICH after ground level fall and no difference in patients on APA or anticoagulants [782].

The relationship between outcome and pre-injury APA is conflicting in the setting of both non-trauma and trauma-related ICH. In patients hospitalised for first-time ICH, aspirin with or without clopidogrel users had a 20–25% increased 30-day stroke mortality compared with non-users [783] and increased ICH volume and mortality was also shown in one RCT performed in patients treated with aspirin [784]. Fatal subdural haematoma was more strongly associated with antithrombotic drug use than non-fatal subdural haematoma [778]. Similarly, patients with APA pre-treatment had a significantly higher risk of death during hospital stay after haematoma evacuation (OR 2.5; 95% CI 1.24–4.97, *p* < 0.01) compared with patients without APA pre-treatment; however, no difference was registered in patients with or without APA receiving conservative therapy [785]. Prior APA use was independently associated with haematoma volume in a prospective study [786], but not in a further retrospective study using propensity score matching [787]. However, a meta-analysis of seven trials in patients on different APA showed a higher risk of prior APA use for haematoma growth but not for mortality [786]. Another meta-analysis of 22 trials on patients with primary ICH showed that prior APA treatment was associated with high mortality that might be attributed mainly to its strong effect on early time [788]. In contrast, only dual APA treatment (mainly aspirin and clopidogrel), but no single agent was associated with higher in-hospital mortality in a large database of patients with ICH [789].

In patients with blunt head trauma, a previous meta-analysis of case-control and cohort studies showed only a slight and non-significant increased risk of death in patients who were taking pre-injury APA [790]. Further studies found both an association with worsening of the lesion [791, 792], need for neurosurgical intervention [791], prolonged hospital stay and increased rate of disability [793], or no influence on survival [794, 795], neurological outcome [786, 796], need for neurosurgical intervention [794, 797], haemorrhagic complications and need of re-operation after decompressive craniectomy [798], questioning the need for routine neurosurgical consultation [797] or repeat CT [799] in cases of mild head trauma or low-altitude falls in patients treated with APA (mainly aspirin, clopidogrel or both).

Those that have specifically analysed the use of clopidogrel prior to traumatic ICH reported an increased risk for unfavorable long-term neurological outcomes [800], progression of the lesion and need for neurosurgical intervention [801]. In contrast, aspirin exposure was not associated with progression of haemorrhage on CT, clinical deterioration or mortality in traumatic ICH [289, 802]. In older patients on preoperative low-dose aspirin undergoing emergency neurosurgery for traumatic ICH, there was also no increased perioperative bleeding, length of hospital stay or in-hospital mortality, but these results should be corroborated with the higher perioperative platelet transfusion rate in patients receiving aspirin therapy [803]. A recent meta-analysis confirmed a statistical association between clopidogrel and clinical deterioration or neurosurgical intervention, but no association between aspirin use and these outcomes in TBI patients [804].

Lower platelet counts add additional risks. A platelet count of < 100 × 10^9^/L is associated with progression of haemorrhagic injury in TBI (pooled OR 4.74 [95% CI 2.44–9.20], *p* < 0.001) [805] and patients with haemorrhagic progression contusion and a platelet count of < 150 × 10^9^/L exhibited a faster rate of expansion [806]. TBI patients on pre-hospital APA with a platelet count of < 135 × 10^9^/L were 12.4 times (95% CI 7.1–18.4) more likely to experience progression of initial ICH on repeated head CT scan; those with a platelet count of 95 × 10^9^/L or less were 31.5 times (95% CI 19.7–96.2) more likely to require neurosurgical intervention [665].

These variable findings, coupled with the fact that some patients are non-responders to aspirin, clopidogrel or both agents, suggest that reliable measures of platelet function would be useful to guide reversal therapies in the setting of the bleeding trauma patient. Patients with occult platelet dysfunction who would benefit from platelet transfusion could be identified or unnecessary platelet transfusion avoided [289]. Currently, there is no agreement on the optimal assay for platelet function (see R11) and controversy exists as to whether bleeding in the setting of APA use warrants platelet transfusion.

Studies addressing the ability of platelet transfusion to improve the laboratory metrics of platelet function have yielded mixed results. An in vitro study performed in healthy volunteers taking aspirin and clopidogrel showed that an equivalent of one or two to three platelet pools (apheresis units) could normalise platelet function in patients on aspirin and aspirin plus clopidogel, respectively [807]. However, in further ex vivo studies, platelet supplementation completely reversed the effect of aspirin [808, 809], but had a limited effect on ADP-dependent aggregation inhibited by clopidogrel [808, 810], prasugrel [810, 811] or ticagrelor [808–810, 812], even at high doses of platelets (up to five apheresis units) [808]. Platelet transfusion also failed to restore platelet aggregation inhibited by ticagrelor and had a small reversing effect in clopidogrel-treated healthy subjects [813]. In TBI patients, platelet transfusion improved or restored platelet function in patients on aspirin [289, 292, 308], but only minimally in others [307] or only partially in both aspirin or clopidogrel [297] but not in collagen trauma-induced platelet dysfunction [308] or only in patients on aspirin but not on clopidogrel [309]. The same contrasting effect of platelet transfusion between aspirin and clopidogrel responders was shown prior to emergency surgery [814]. However, the multiple platelet function assays and different platelet suspensions (of potentially variable quality) used in these studies make comparisons difficult. Another explanation for the observation that platelet transfusion shows no obvious benefit is that the inhibitory effect of the APA is not normalised due to recent ingestion of APA, which may also inactivate transfused platelets [815]. The time-dependent effect of platelet transfusion has been shown for both clopidogrel [808], prasugrel [811] and ticagrelor [812, 815] and recurring platelet transfusion may be justified. A dose response to platelet transfusion was noted in one study, and two thirds of aspirin-treated initial non-responders corrected with the second platelet transfusion [289].

Biological plausibility coupled with assay results indicating the presence of significant platelet inhibition make it difficult not to treat these patients with platelet transfusions when injured, although current evidence to support this practice is conflicting. Successful perioperative management of patients on aspirin and clopidogrel requiring urgent surgery by administering two apheresis platelet units preoperatively was recently reported [816]. However, the bleeding and re-intervention rate was 12.2% and 6.6%, respectively.

A systematic review of five retrospective registry studies on traumatic ICH [817] and a meta-analysis that included six small studies on the impact of platelet transfusion on survival in patients treated with pre-injury APA who experienced ICH, either spontaneous or traumatic, found no clear benefit [276]. In a further systematic review of seven retrospective studies of APA-associated ICH in both trauma and non-trauma settings, the pooled in-hospital mortality for platelet transfusion in traumatic ICH patients was 1.77 (95% CI 1.00–3.13), whereas the pooled in-hospital mortality for platelet transfusion in primary intra-parenchymal haemorrhage was 0.49 (95% CI 0.24–0.98). However, due to the methodological limitations of the reviewed studies, no conclusions were drawn by the authors [818]. One important potential confounding factor in these studies is the dose of platelets, which was either not reported or suboptimal for normalisation of platelet function. The timing of platelet administration could be another confounding factor but this was not confirmed in a large retrospective study in patients with TBI while on APA [819]. Patients receiving early platelet transfusion within 4 h were more severe and had a higher rate of worsening haematoma and mortality.

In a double-blind RCT in patients with ICH while on aspirin and undergoing craniotomy, those aspirin-sensitive who received platelet transfusion (either one unit, two units vs no units of frozen apheresis platelets) had less ICH recurrence, lower postoperative haematoma volume, lower mortality and better functional outcome at 6 months compared with those who did not [784]. In contrast, fresh apheresis platelet transfusion was inferior to standard of care for patients taking aspirin or clopidogrel in a recent multicentre, randomised, open-label phase 3 trial in 190 patients with acute stroke due to spontaneous ICH associated with APA (platelet transfusion in cerebral haemorrhage, PATCH) [820]. Notably, the PATCH trial did not include patients with a GCS < 8 or those thought to need operative intervention. The odds of death and dependence at 3 months were higher in the platelet transfusion group (OR 2.05; 95% CI 1.18–3.56). There was also an increase in haematoma growth at 24 h in patients treated with platelet transfusion (2.01 mL vs 1.16 mL, *p* = 0.08), which was reflected in more severe adverse events due to haematoma expansion.

Further observational studies in ICH patients have shown either that platelet transfusion had no negative effects [821] or was not associated with improved outcome [822]. Platelet transfusion given before cranial decompressive surgery was partially effective in patients on clopidogrel [823]. In TBI patients on APA pre-treatment, platelet transfusion did not improve the neurological outcome [309, 794, 803], but was associated with increased infections and complications [794].

The limited evidence that APA use prior to ICH actually has any impact on haemorrhage expansion or outcome, together with the lack of substantive data to suggest that platelet transfusion improves outcome and the risks associated with platelet transfusion, do not favour platelet transfusion in patients with APA-associated ICH who will not undergo a neurosurgical procedure [824].

Besides platelet transfusion, potential antiplatelet reversal therapies include rFVIIa, TXA and desmopressin. In healthy volunteers, rFVIIa reversed the inhibitory effects of aspirin [825] and clopidogrel [826]. The effective dose was lower than the dose used in haemophilia patients [826]. Interestingly, TXA was shown to partially improve platelet function both in patients treated with dual antiplatelet therapy as measured using MEA [827] and in aspirin-free patients [828].

Desmopressin (1-deamino-8-d-arginine vasopressin, DDAVP) releases endothelial von Willebrand factor and factor VIII, enhances platelet adherence and platelet aggregate growth on human artery subendothelia and is the first choice in the treatment of bleeding in patients with von Willebrand disease, a disorder which occurs in roughly 1 in 100 patients [829]. It is also beneficial perioperatively in patients with mild inherited platelet defects [830].

Two meta-analyses [831, 832] and a Cochrane analysis [833] showed modest but significant reduction in perioperative RBC transfusion needs due to treatment with desmopressin. The more recent meta-analysis focused on platelet dysfunction and reversal of APA in cardiac surgery [832] and was able to demonstrate a reduction in blood loss [− 254 (− 408 to − 100) mL], a reduction in blood transfusion requirements [− 0.65 (− 1.16 to − 0.13) units per patient] and a reduced risk of re-operation due to bleeding [OR 0.39 (0.18 to 0.84)]. Identification of impaired platelet function with a PFA-100® [834] or MEA [835] might be helpful in the identification of patients who could benefit from desmopressin therapy. In contrast, desmopressin did not improve platelet function measured with three different devices (MEA, ROTEM® and Sonoclot®) in vitro [836].

Desmopressin has been shown to improve platelet function in volunteers on aspirin [837] or clopidogrel [838]. It had no effect on inhibition of platelet aggregation by ticagrelor, although primary haemostatic activity was significantly increased [839]. Equivalent data for prasugrel appear not to have been published, and there is evidence from a recent animal study that bleeding was not reduced in prasugrel-treated animals due to desmopressin [840].

Two small prospective studies have shown that desmopressin can improve platelet function in patients with ICH who have received aspirin [841] or not [842] prior to the event. However, desmopressin and platelet administration was not associated with either a decreased risk of early radiographic haemorrhage progression (OR 1.40; 95% CI 0.80–2.40; *p* = 0.2) or mortality (OR 1.50; 95% CI 0.60–4.30; *p* = 0.4) in patients with traumatic ICH [843]. Interestingly, desmopressin prevents the development of hypothermia-induced impairment of primary haemostasis [837, 844] and significantly increases platelet aggregation during hypothermia and acidosis [845].

Although the evidence is scarce, desmopressin has been recommended in ICH patients treated with APA [824] and in trauma patients with von Willebrand disease [846]. The recommended dose in ICH is 0.4 μg/kg [824], but the usual dose in von Willebrand disease is 0.3 μg/kg diluted in 50 mL saline and infused over 30 min [829]. When administering desmopressin, an antifibrinolytic (e.g. TXA) should be administered in parallel.

### VIII. Thromboprophylaxis

#### Thromboprophylaxis

##### Recommendation 37

We recommend early mechanical thromboprophylaxis with intermittent pneumatic compression (IPC) while the patient is immobile and has a bleeding risk. (Grade 1C)

We recommend combined pharmacological and IPC thromboprophylaxis within 24 h after bleeding has been controlled and until the patient is mobile. (Grade 1B)

We do not recommend the use of graduated compression stockings for thromboprophylaxis. (Grade 1C)

We do not recommend the routine use of inferior vena cava filters as thromboprophylaxis. (Grade 1C)

#### Rationale

The risk of hospital-acquired VTE is high, exceeding 50%, following multiple trauma. Pulmonary embolism (PE) is the third leading cause of death in those who survive beyond the third day [847]. There are few RCTs that have investigated thromboprophylaxis in trauma patients, and the use of graduated compression stockings has never been evaluated in this group. A meta-analysis was unable to show any reduction in the rate of DVT with IPC [848]; however, mechanical methods are widely used due to the low bleeding risk.

There is inadequate research on the use of mechanical thromboprophylaxis in critical care. Of note, there is no evidence to show graduated compression stockings reduce the risk of death due to a PE in any area. The clots in legs or stockings after stroke (CLOTS 3) study was the first large RCT to investigate the utility of IPC alone, without pharmacological thromboprophylaxis in 2876 stroke patients. The study showed a clear benefit, with a reduction in DVT from 12.1 to 8.5% and an absolute reduction of 3.6% (95% CI 1.4 to 5.8), with a non-significant reduction in death [849], suggesting that IPC alone was ideal for patients who are not yet ready for combined pharmacological and IPC thromboprophylaxis after admission. While the population in this study was different from those in critical care, both populations have similar risk factors (immobility and acute phase response). The recent Cochrane review [850] on the use of combined IPC and pharmacological thromboprophylaxis compared to either alone concluded that there is moderate evidence to suggest a significantly better reduction in VTE with combination therapy.

A systematic review and meta-analysis [851] showed that any type of heparin thromboprophylaxis decreases DVT and PE in critically ill medical and surgical patients, and low molecular weight heparin (LMWH) compared with twice daily unfractionated heparin (UFH) decreases both the overall rate and symptomatic rate of PE. Major bleeding and mortality rates did not appear to be significantly influenced by heparin thromboprophylaxis in the ICU setting. Another study of 289 patients who developed VTE during or after a critical care stay showed that thromboprophylaxis failure was more likely in association with an elevated body mass index, a personal or family history of VTE and those administered vasopressors [852].

LMWH has an efficacy similar to UFH but is associated with a lower rate of heparin-induced thrombocytopenia. In addition, less frequent injections are required due to its longer half-life and more reliable pharmacokinetics; therefore, LMWH is also preferred due to ease of administration. The severity of trauma has been associated with the risk of heparin-induced thrombocytopenia; therefore, the greater the risk, the greater the importance of monitoring platelet counts in trauma patients [853]. In those with a bleeding risk, mechanical methods alone are preferable until the bleeding risk recedes. LMWH is mainly excreted renally, unlike UFH, which is excreted via the liver as well; therefore, there is risk of LMWH accumulation in patients with renal failure. According to the manufacturer’s instructions, dose adjustments and/or anti-factor Xa monitoring should be performed if patients with renal failure require LMWH for longer than one week and have a bleeding tendency.

There has been some interest in the administration of a monitored dose of LMWH to trauma patients with a high risk of VTE. Connelly et al. administered more LMWH based on TEG® monitoring; however, the study showed no difference in VTE rates among the 89 patients who were not and the 96 who were monitored [854]. Ko et al. dosed LMWH based on anti-factor Xa levels in 87 patients versus 118 in the control group [855]. Most of the monitored group had enoxaparin doses increased from 30 mg to 40 mg twice daily. The incidence of VTE fell from 7.6% in the control group to 1.1% in the monitored group (*p* = 0.46) and no significant difference was noted in the transfusion of RBC or Hct at discharge. Singer et al. noted a fall in VTE rates in 131 patients in whom anti-factor Xa activity was monitored, but the study was flawed due to comparison with a historical control group [856]. Some authors have expressed interest in using DOAC instead of LMWH for thromboprophylaxis; however, no adequately powered RCT has addressed this issue [857].

Contraindications to pharmacological thromboprophylaxis include patients who are already receiving full-dose anticoagulation, those with significant thrombocytopenia (platelet count < 50 × 10^9^/L), an untreated inherited or acquired bleeding disorder, evidence of active bleeding, uncontrolled hypertension (blood pressure > 230/120), a lumbar puncture/spinal analgesia expected within the next 12 h or performed within the last 4 h (24 h if traumatic), procedures with a high bleeding risk or a new haemorrhagic stroke. However, a recent systematic review found that pharmacological thromboprophylaxis appears to be safe among patients with TBI and stabilised haemorrhagic patterns [858].

The optimal timing for the initiation of pharmacological thromboprophylaxis may be difficult to estimate. Data from 175,000 critical care admissions showed that the risk of mortality was higher in those who did not receive thromboprophylaxis during the first 24 h [859]. This reflects the observation that those who bleed have a higher rate of VTE than those who do not [860]. For trauma patients with TBI, we suggest that pharmacological VTE prophylaxis be initiated with either LMWH, or low-dose UFH in patients with renal failure, only after a head CT confirms that ICH is stable and the absence of persistent bleeding.

The use of prophylactic inferior vena cava filters is common in some units; however, no evidence of added benefit when used in combination with pharmacological thromboprophylaxis exists. PE still occur despite the presence of a filter, and filters have short- and long-term complication rates, are associated with high cost and often provide a false sense of security, delaying the use of effective pharmacological thromboprophylaxis. Furthermore, inferior vena cava filters require a second invasive procedure to remove.

### IX. Guideline implementation and quality control

#### Guideline implementation

##### Recommendation 38

We recommend the local implementation of evidence-based guidelines for management of the bleeding trauma patient. (Grade 1B)

#### Assessment of bleeding control and outcome

##### Recommendation 39

We recommend that local clinical quality and safety management systems include parameters to assess key measures of bleeding control and outcome. (Grade 1B)

#### Rationale

Implementation of treatment guidelines in complex areas of clinical care, such as the management of trauma patients, is challenging [861–865]. However, repetitive educational activities addressing all healthcare providers involved have been shown to be successful in increasing guideline adherence [862, 865]. Therefore, the evaluation of healthcare provider perspectives on the guideline quality plays an important role in a successful implementation process [862]. High guideline credibility, as well as a strong and well-communicated leadership commitment to the guidelines, can increase adherence [862]. Furthermore, monitoring of guideline adherence, via chart review [865] or video recording in the trauma bay or the emergency department [861], with feedback to all healthcare providers involved has been found to improve guideline adherence.

Higher guideline adherence in turn results in improved survival in adult [863] and paediatric [862] patients suffering from TBI. Additionally, in general trauma, adherence to these European guidelines on the management of bleeding trauma patients resulted in higher patient survival [41]. In a multivariate analysis after adjustment for the ISS, the per-patient guideline adherence rate was a highly significant factor for a decreased mortality at 30 days (OR 0.47 (0.31–0.72, *p* = 0.0004) [41]. In a similar study, the outcome of major trauma patients was compared before and after the implementation of strict trauma treatment guidelines, mostly identical to these guidelines, in particular regarding goal-directed coagulation and transfusion protocols, primary WBCT, the use of TXA, restrictive fluid therapy (preferably crystalloids), permissive hypovolemia/hypotension and damage-control surgery [43]. The primary outcome was the observed vs the TASH [866] score-predicted incidence of massive transfusion from emergency department/operating room arrival until ICU admission. The observed incidence of massive transfusion (12.4%) was similar to the TASH prediction (12.1%) prior to the introduction of trauma treatment guidelines. However, with the introduction of treatment guidelines, the observed massive transfusion incidence of 3.7% was significantly lower than the TASH prediction of 7.5% (*p* < 0.01). Interestingly, the percentage of transfused patients and the amount of transfused blood products were also significantly decreased, as was hospital mortality [43]. Thus, implementation of evidence-based guidelines for management of the bleeding trauma patient is likely to improve the outcome.

The implementation of our recommendations might be facilitated by a checklist approach analogous to the Safe Surgery Initiative [867], which led to fewer postoperative complications [868]. In addition or alternatively, it may be possible to implement our recommendations using “bundles”, as has been successfully achieved during implementation of the Surviving Sepsis Campaign guidelines [869, 870]. Suggested items that should be included in such a checklist are summarised in Table 4. Suggested patient management bundles are listed in Table 5.

**Table 4:** Treatment pathway checklist. Hb, haemoglobin; TBI, traumatic brain injury

Treatment phase	Yes	No	N/A	Reason for variance
Initial assessment and management				
Extent of traumatic haemorrhage assessed	☐	☐	☐	
Patient in shock with identified source of bleeding treated immediately	☐	☐	☐	
Patient in shock with unidentified source of bleeding sent for further investigation	☐	☐	☐	
Coagulation, haematocrit, serum lactate, base deficit assessed	☐	☐	☐	
Antifibrinolytic therapy initiated	☐	☐	☐	
Patient history of anticoagulant therapy assessed (vitamin K antagonists, antiplatelet agents, oral anticoagulants)	☐	☐	☐	
Resuscitation				
Systolic blood pressure of 80–90 mmHg achieved in absence of TBI	☐	☐	☐	
Measures to achieve normothermia implemented	☐	☐	☐	
Target Hb level 70–90 g/L achieved	☐	☐	☐	
Surgical intervention				
Abdominal bleeding control achieved	☐	☐	☐	
Pelvic ring closed and stabilised	☐	☐	☐	
Peritoneal packing, angiographic embolisation or surgical bleeding control completed in haemodynamically unstable patient	☐	☐	☐	
Damage-control surgery performed in haemodynamically unstable patient	☐	☐	☐	
Local haemostatic measures applied	☐	☐	☐	
Thromboprophylactic therapy recommended	☐	☐	☐	
Coagulation management				
Coagulation, haematocrit, serum lactate, base deficit, calcium reassessed	☐	☐	☐	
Target fibrinogen level 1.5–2 g/L achieved	☐	☐	☐	
Target platelet level achieved	☐	☐	☐	
Prothrombin complex concentrate administered if indicated due to vitamin K antagonist, oral anticoagulant or evidence from viscoelastic methods	☐	☐	☐

**Table 5:** Suggested management bundles. BE, base excess; CT, computed tomography; FAST, focused assessment with sonography in trauma; Hb, haemoglobin; PT, prothrombin time

Pre-hospital bundle	Intra-hospital bundle	Coagulation bundle
• Pre-hospital time minimised • Tourniquet employed in case of life-threatening bleeding from extremities • Damage-control resuscitation concept applied • Trauma patient transferred directly to an adequate trauma specialty centre	• Full blood count, PT, fibrinogen, calcium, viscoelastic testing, lactate, BE and pH assessed within the first 15 min • Immediate intervention applied in patients with haemorrhagic shock and an identified source of bleeding unless initial resuscitation measures are successful • Immediate further investigation undertaken using FAST, CT or immediate surgery if massive intra-abdominal bleeding is present in patients presenting with haemorrhagic shock and an unidentified source of bleeding • Damage-control surgery concept applied if shock or coagulopathy are present • Damage-control resuscitation concept continued until the bleeding source is identified and controlled • Restrictive erythrocyte transfusion strategy (Hb 70–90 g/L) applied	• Tranexamic acid administered as early as possible • Acidosis, hypothermia and hypocalcaemia treated • Fibrinogen maintained at 1.5–2 g/L • Platelets maintained at > 100 × 10^9^/L • Prothrombin complex concentrate administered in patients pre-treated with warfarin or direct-acting oral coagulants (until antidotes are available)

Training in trauma care should emphasise the key role of coagulation in determining outcome. Increasing clinician knowledge and understanding in this area should be an integral part of the implementation of the algorithm. All trauma care centres should evaluate their own performance using a routine institutional quality management programme. An audit of adherence to best practice, including feedback and practice change where needed, should be included as part of the local implementation of these guidelines. In order to evaluate the quality of care provided to the patient who is bleeding after major trauma, we suggest that adherence to the following quality standards be assessed:

- Time from injury to the initiation of intervention to stop bleeding (surgery or embolisation) in hypotensive patients who do not respond to initial resuscitation
- Time from hospital arrival to availability of a full set of blood results [full blood count, PT, fibrinogen, calcium, viscoelastic testing (if available)]
- Proportion of patients receiving TXA within 3 h after injury
- Time from hospital arrival to CT scan in bleeding patients without an obvious source of haemorrhage
- Damage-control surgical techniques used in accordance with R18
- Thromboprophylaxis commenced in accordance with R37

## Discussion

These guidelines (summarised in Additional file 3) reflect the management of significant bleeding and coagulopathy following major trauma based on the current published scientific evidence. Expert opinion and current clinical practice were also considered, particularly in areas in which randomised clinical trials have not or cannot be performed for practical or ethical reasons. Recommendations published in previous editions of the guideline [36–39] were reconsidered and revised as appropriate.

The recommendations included in the guideline are intended to guide the management of patients during the early phase of hospital care following traumatic injury. However, some of the recommendations and principles discussed may also apply to the pre-hospital setting. Specific examples include the use of tourniquets (R2) and the first administration of TXA (R22) at the site of injury.

**Fig. 2:**
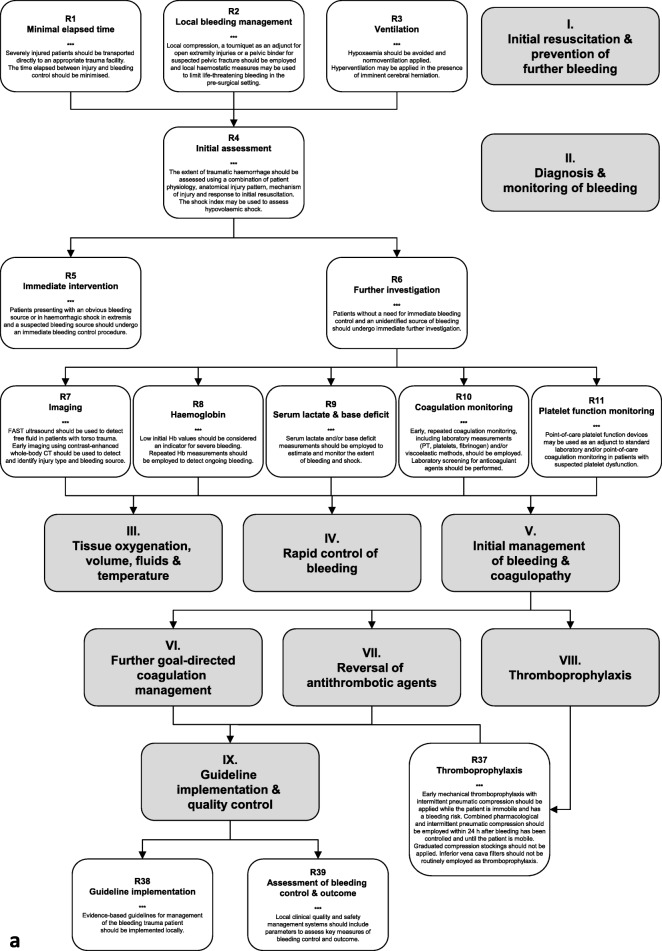

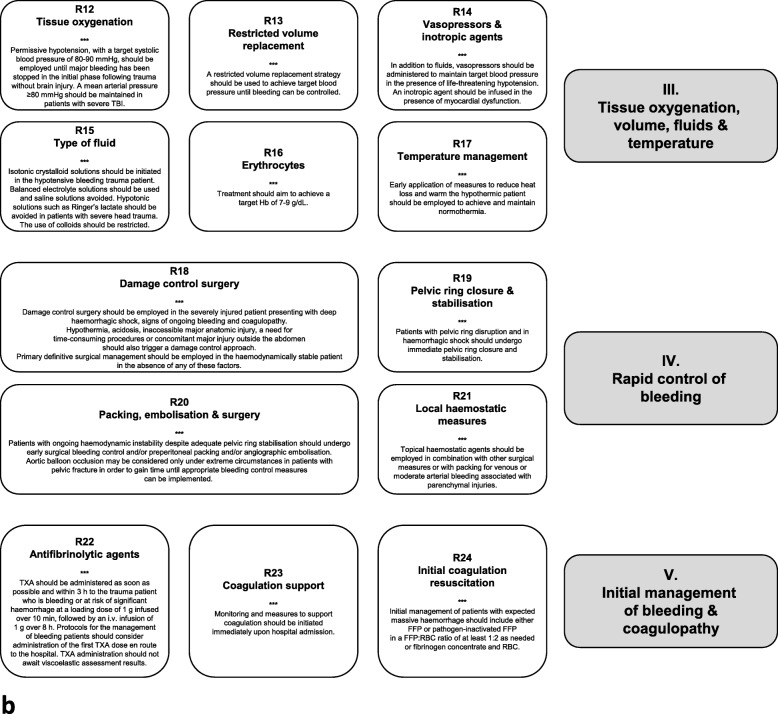

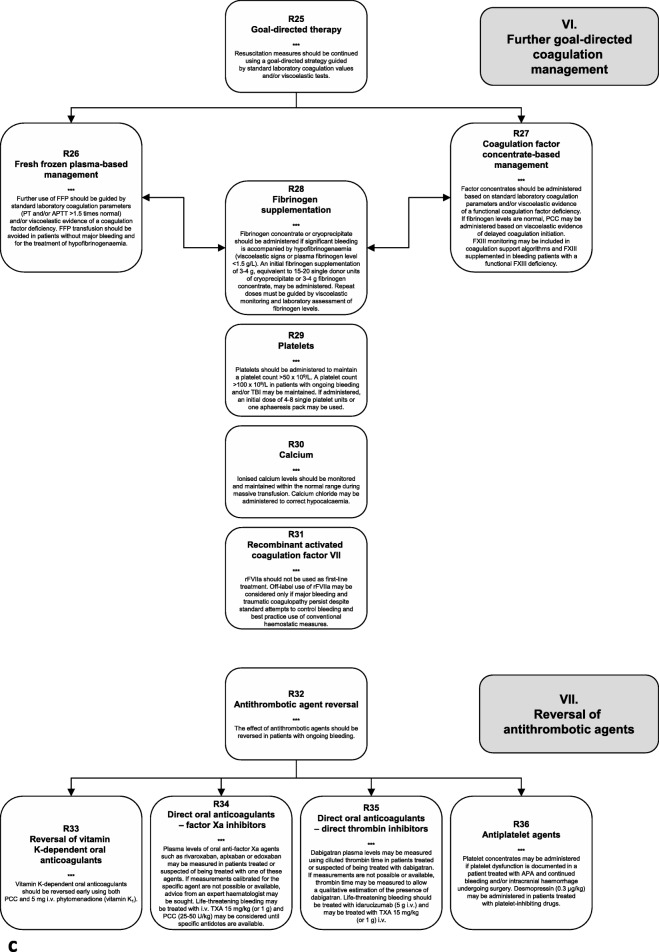
**a** Summary of treatment modalities for the bleeding trauma patients included in this guideline. CT, computed tomography; FAST, focused assessment with sonography in trauma; Hb, haemoglobin; PT, prothrombin time. **b** Summary of treatment modalities for the bleeding trauma patients included in this guideline. FFP, fresh frozen plasma; Hb, haemoglobin; RBC, red blood cells; TBI, traumatic brain injury; TXA, tranexamic acid. **c** Summary of treatment modalities for the bleeding trauma patients included in this guideline. APA, antiplatelet agent; APTT, activated partial thromboplastin time; FFP, fresh frozen plasma; FXIII, factor XIII; PCC, prothrombin complex concentrate; PT, prothrombin time; rFVIIa, recombinant activated coagulation factor VII; TBI, traumatic brain injury; TXA, tranexamic acid

In this fifth version, the overall organisation of the guideline has been revised to better reflect the decision-making process along the patient pathway and group recommendations behind the rationale for key decision points. The guideline now has nine separate chapters, organised in approximate temporal sequence (Fig. 2). These chapters are now patient- or problem-oriented rather than related to treatment modalities. In particular, the former chapter on further resuscitation measures has now been reorganised into three separate chapters (chapters VI, VII, VIII).

Chapter VI (“Further goal-directed coagulation management”) now discusses goal-directed therapy (R25), which comprises either an FFP-based strategy (R26) or CFC-based management (R27), including a new statement about the use of FXIII replacement therapy. This chapter comprises recommendations regarding fibrinogen supplementation (R28), platelet administration (R29), calcium (R30) and rFVIIa (R31). Chapter VII (“Reversal of antithrombotic agents”) discusses monitoring and treatment of trauma patients who are anticoagulated (R33, R34, R35) or being treated with platelet inhibitors (R36). The number of patients in this group is rapidly increasing, and their treatment represents a significant challenge if such patients suffer major trauma. Finally, chapter VIII (“Thromboprophylaxis”) provides a recommendation for the prophylactic prevention of thromboembolic complications (R37) in major trauma patients, which is increasingly recognised as important, particularly in patients treated prior to traumatic injury with oral anticoagulants and/or platelet inhibitors.

The present guideline represents an educational aid to improve and standardise the care of the bleeding trauma patient across Europe and beyond. The recommendations that comprise the final chapter IX (“Implementation & quality control”) continue to encourage the local implementation (R38) of evidence-based guidelines for the management of the bleeding patient following traumatic injury and that local quality and safety management systems (R39) specifically assess key measures of bleeding control and outcome.

We continue to concur that both children and elderly adults who have not been pre-treated with anticoagulant or antiplatelet agents should generally be managed in the same manner as the normal adult patient. However, most clinical studies investigate standard size; otherwise, healthy adults and do not stratify by characteristics that might justify more nuanced recommendations. Therefore, except where addressed for specific recommendations in the guideline, we are unable to make informed recommendations for the treatment of many subpopulations that may include children, women, specific ethnic groups, individuals with high or low body mass indices or many other co-morbidities or conditions.

The frequent scientific citations and downloads of the previous editions of the guideline [36–39] demonstrate the interest in the subject matter and popularity of these guidelines. Only reports showing improved outcomes can serve as final proof of the usefulness of these guidelines, however, and publications from Italian, French and Swiss trauma centres [41, 43, 523] are promising.

## Conclusions

The appropriate management of trauma patients with massive bleeding and coagulopathy remains a major challenge in routine clinical practice. A multidisciplinary approach and adherence to evidence-based guidance are key to improving patient outcomes, which could now be shown in the first outcome studies.

## Additional files

Additional file 1: Structured literature search strategies. (PDF 34 kb)

Additional file 2: Levels of evidence according to [45] for evidence cited in this guideline. (PDF 794 kb)

Additional file 3: Summary of recommendations. (PDF 103 kb)

## Abbreviations

AA: Arachidonic acid
ACIT: Activation of Coagulation and Inflammation in Trauma
ACS: Abdominal compartment syndrome
ADP: Adenosine diphosphate
APA: Antiplatelet agents
aPCC: Activated prothrombin complex concentrate
APTT: Activated partial thromboplastin time
ARDS: Acute respiratory distress syndrome
ASPI: Arachidonic acid receptor aspirin inhibition
ATLS: Advanced Trauma Life Support
ATP: Adenosine triphosphate
AUC: Area under the curve
BE: Base excess
CCA: Conventional coagulation assays
CE: Contrast extravasation
CECT: Contrast-enhanced computed tomography
CEUS: Contrast-enhanced ultrasound
CFC: Coagulation factor concentrates
CI: Confidence interval
CRASH-2: Clinical Randomisation of Antifibrinolytic in Significant Haemorrhage
CT: Computed tomography
DCR: Damage-control resuscitation
DDAVP: 1-Deamino-8-d-arginine vasopressin
DOAC: Direct oral anticoagulant
DVT: Deep venous thrombosis
EMA: European Medicines Agency
ESA: European Society of Anaesthesiology
ESICM: European Society of Intensive Care Medicine
ESS: European Shock Society
ESTES: European Society for Trauma and Emergency Surgery
EuSEM: European Society for Emergency Medicine
EXTEM: Extrinsically activated test
FAST: Focused assessment with sonography in trauma
FDA: US Food and Drug Administration
FF: Functional fibrinogen
FFP: Fresh frozen plasma
FIBTEM: Fibrin-based extrinsically activated test
FXIII: Factor XIII
GCS: Glasgow Coma Scale
GRADE: Grading of Recommendations Assessment, Development and Evaluation
Hb: Haemoglobin
Hct: Haematocrit
HES: Hydroxyethyl starch
HTPR: High on-treatment platelet reactivity
i.v.: Intravenous
ICH: Intracranial haemorrhage
ICU: Intensive care unit
IgE: Immunoglobulin E
INR: International normalised ratio
IPC: Intermittent pneumatic compression
IQR: Interquartile range
ISS: Injury severity score
iTACTIC: Implementing Treatment Algorithms for the Correction of Trauma Induced Coagulopathy
LMWH: Low molecular weight heparin
LTA: Light transmission aggregometry
MATTERs II: Military Application of Tranexamic Acid in Trauma Emergency Resuscitation
MCF: Maximum clot firmness
MDCT: Multidetector computed tomography
MEA: Multiple electrode impedance aggregometry
MeSH: Medical subject headings
MODS: Multiple organ dysfunction syndrome
MSCT: Multislice computed tomography
NATA: Network for the Advancement of Patient Blood Management, Haemostasis and Thrombosis
NO: Nitric oxide
NPV: Negative predictive value
OCEBM: Oxford Centre for Evidence-Based Medicine
OR: Odds ratio
PATCH: Platelet transfusion in cerebral haemorrhage
PCC: Prothrombin complex concentrate
PE: Pulmonary embolism
PEEP: Positive end-expiratory pressure
PFA: Platelet function analyser
PICO: Patient, problem or population, intervention, comparison, control or comparator, outcome
POC: Point-of-care
PPV: Positive predictive value
PROMMTT: Prospective, Observational, Multicenter, Major Trauma Transfusion
PROPPR: Pragmatic, Randomized Optimal Platelet and Plasma Ratios
PT: Prothrombin time
PTOS: Pennsylvania Trauma Outcome Study
RBC: Red blood cells
RCT: Randomised controlled trial
RD: Risk difference
REBOA: Resuscitative endovascular balloon occlusion of the aorta
RETIC: Reversal of Trauma Induced Coagulopathy Using Coagulation Factor Concentrates or Fresh Frozen Plasma
rFVIIa: Recombinant activated coagulation factor VII
ROTEM: Rotational thromboelastometry
RPH: Retroperitoneal haemorrhage
RR: Risk ratio
r-TEG: Rapid thromboelastography
SI: Shock index
SMR: Standardised mortality ratio
SOP: Standard operating procedure
TACO: Transfusion-associated circulatory overload
TASH: Trauma-associated severe haemorrhage
TBI: Traumatic brain injury
TEG: Thrombelastography
TEG-PM: Thrombelastography platelet mapping
TEM: Thromboelastometry
TRALI: Transfusion-related acute lung injury
TRAP: Thrombin receptor activating peptide
TRICC: Transfusion Requirements in Critical Care
TXA: Tranexamic acid
UFH: Unfractionated heparin
VASP: Vasodilator-stimulated phosphoprotein
VEM: Viscoelastic methods
VKA: Vitamin K antagonist
VN®-ASA: VerifyNow® platelet reactivity test for aspirin
VTE: Venous thromboembolism
WBCT: Whole-body computed tomography
WHO: World Health Organization
