# Supplementary material for: The European guideline on management of major bleeding and coagulopathy following trauma: fifth edition
Source: Crit Care. 2019 Mar 27;23:98. doi: 10.1186/s13054-019-2347-3 (PMC6436241; doi:10.1186/s13054-019-2347-3)
Supplement: Supplementary file 1 — Structured literature search strategies. (PDF 34 kb) [file 13054_2019_2347_MOESM1_ESM.pdf]

The European guideline on management of major bleeding and coagulopathy following trauma: Fifth edition
Spahn DR, Bouillon B, Cerny V, Duranteau J, Filipescu D, Hunt BJ, Komadina R, Maegele M, Nardi G, Riddez L, Samama C-M, Vincent J-L, Rossaint R

I. INITIAL RESUSCITATION AND PREVENTION OF FURTHER BLEEDING

Search performed 31.01.2018
824 hits

("Wounds and Injuries"[Mesh] OR "Trauma Severity Indices"[Mesh] OR injur*[tiab] OR trauma*[tiab] OR polytrauma*[tiab]) AND ("Hemorrhage"[Mesh] OR "Blood Coagulation Disorders"[Mesh] OR "Disseminated Intravascular Coagulation"[Mesh] OR bleed*[tiab] OR hemorrhag*[tiab] OR haemorrhag*[tiab] OR coagulation disorder*[tiab] OR coagulopath*[tiab] OR disseminated intravascular coagulation[tiab] OR DIC[tiab]) AND ("Resuscitation"[Mesh] OR "Tourniquets"[Mesh] OR "Intubation, Intratracheal"[Mesh] OR resuscitation*[tiab] OR tourniquet*[tiab] OR wound compression*[tiab] OR ventilation*[tiab] OR (tracheal [tiab] OR intratracheal[tiab] OR endotracheal[tiab] AND intubation*[tiab]) OR triage[tiab] OR triage[mesh] OR overtriage[tiab] OR ((selecting[tiab] OR choosing[tiab] OR selection[tiab] OR choice[tiab] OR prioritize[tiab] OR prioritise[tiab] OR prioritizing[tiab]) AND (Hospital[tiab] OR hospitals[tiab] OR clinic[tiab] OR center[tiab] OR centre[tiab] OR clinics[tiab] OR centers[tiab] OR centres[tiab] OR „Emergency Service, Hospital"[mesh] OR trauma room*[tiab] OR resuscitation area*[tiab]))) AND humans[mesh] AND ("2015/02/01"[Date - Publication] : "3000"[Date - Publication]) NOT ("Comment" [Publication Type] OR "Letter" [Publication Type] OR "Editorial" [Publication Type])

II. DIAGNOSIS AND MONITORING OF BLEEDING

Search performed 06.02.2018 (imaging)
3841 hits

("Wounds and Injuries"[Mesh] OR "Trauma Severity Indices"[Mesh] OR injur*[tiab] OR trauma*[tiab] OR polytrauma*[tiab]) AND ("Hemorrhage"[Mesh] OR "Blood Coagulation Disorders"[Mesh] OR "Disseminated Intravascular Coagulation"[Mesh] OR bleed*[tiab] OR hemorrhag*[tiab] OR haemorrhag*[tiab] OR coagulation disorder*[tiab] OR coagulopath*[tiab] OR disseminated intravascular coagulation[tiab] OR DIC[tiab]) AND ("Diagnosis"[Majr] OR "diagnosis" [Subheading] OR "Diagnostic Imaging"[Majr] OR "Tomography, X-Ray Computed"[Majr] OR "diagnostic imaging" [Subheading] OR "Ultrasonography"[Majr] OR "Signs and Symptoms"[Majr] OR "Monitoring, Physiologic"[Majr] OR clinical signs[tiab] OR ((diagnosis[tiab]OR diagnostic[tiab]) AND (tool[tiab] OR tools[tiab] OR score[tiab] OR scores[tiab])) OR ATLS shock classification[tiab] OR imaging[tiab] OR computer tomography[tiab] OR computer tomographies[tiab] OR CT[tiab] OR computed tomography[tiab] OR computed tomographies[tiab] OR ultrasonography[tiab] OR ultrasonographies[tiab] OR ultrasound[tiab] OR (hybrid[tiab] AND ((shock[tiab] OR resuscitation[tiab] OR operating[tiab]) AND (room[tiab] OR facility[tiab] OR facilities[tiab])))) AND humans[mesh] AND ("2015/02/01"[Date - Publication] : "3000"[Date - Publication]) NOT ("Comment" [Publication Type] OR "Letter" [Publication Type] OR "Editorial" [Publication Type])

Search performed 06.02.2018 (laboratory)
928 hits

("Wounds and Injuries"[Mesh] OR "Trauma Severity Indices"[Mesh] OR injur*[tiab] OR trauma*[tiab] OR polytrauma*[tiab]) AND ("Hemorrhage"[Mesh] OR "Blood Coagulation Disorders"[Mesh] OR "Disseminated Intravascular Coagulation"[Mesh] OR bleed*[tiab] OR hemorrhag*[tiab] OR haemorrhag*[tiab] OR coagulation disorder*[tiab] OR coagulopath*[tiab] OR disseminated intravascular coagulation[tiab] OR DIC[tiab]) AND ("Chemistry, Clinical"[Majr] OR "Clinical Chemistry Tests"[Majr] OR "Hematologic Tests"[Majr] OR "Blood Coagulation Tests"[Majr] OR "Point-of-Care Testing"[Majr] OR "Platelet Aggregation Inhibitors"[Mesh] OR "Antithrombins"[Mesh] OR "Factor Xa Inhibitors"[Mesh] OR ((laboratory[tiab] OR coagulation[tiab] OR point of care[tiab] OR platelet function[tiab]) AND (test[tiab] OR tests[tiab] OR testing[tiab] OR parameter[tiab] OR parameters[tiab] OR monitoring[tiab])) OR hematocrit[tiab] OR haematocrit[tiab] OR hemoglobin[tiab] OR haemoglobin [tiab] OR serum lactate[tiab] OR base defict[tiab] OR D-dimer[tiab] OR (viscoelastic[tiab] AND (test[tiab] OR tests[tiab] OR testing[tiab] OR monitoring[tiab])) OR anticoagulan*[tiab] OR DOACs[tiab] OR antiplatelet*[tiab] OR antithrombin*[tiab] OR anti factor Xa[tiab] OR ((platelet[tiab] OR thrombin[tiab] OR factor Xa[tiab]) AND inhibitor*[tiab]) OR rivaroxaban[tiab] OR apixaban[tiab] OR edoxaban [tiab] OR betrixaban[tiab] OR dabigatran[tiab]) AND humans[mesh] AND ("2015/02/01"[Date - Publication] : "3000"[Date - Publication]) NOT ("Comment" [Publication Type] OR "Letter" [Publication Type] OR "Editorial" [Publication Type])

III. Tissue oxygenation, type of fluid and temperature management

Search performed 31.01.2018
843 hits

("Wounds and Injuries"[Mesh] OR "Trauma Severity Indices"[Mesh] OR injur*[tiab] OR trauma*[tiab] OR polytrauma*[tiab]) AND ("Hemorrhage"[Mesh] OR "Blood Coagulation Disorders"[Mesh] OR "Disseminated Intravascular Coagulation"[Mesh] OR bleed*[tiab] OR hemorrhag*[tiab] OR haemorrhag*[tiab] OR coagulation disorder*[tiab] OR coagulopath*[tiab] OR disseminated intravascular coagulation[tiab] OR DIC[tiab]) AND ("Blood Pressure"[Mesh] OR "Hematocrit"[Mesh] OR "Hypotension"[Mesh] OR "Vasoconstrictor Agents"[Mesh] OR "Cardiotonic Agents"[Mesh] OR "Fluid Therapy"[Mesh] OR "Hypertonic Solutions"[Mesh] OR "Hypotonic Solutions"[Mesh] OR "Isotonic Solutions"[Mesh] OR "Colloids"[Mesh] OR "crystalloid solutions" [Supplementary Concept] OR "Electrolytes"[Mesh] OR "Sodium Chloride"[Mesh] OR "Plasma Substitutes"[Mesh] OR "Blood Substitutes"[Mesh] OR "Erythrocytes"[Mesh] OR "Erythrocyte Transfusion"[Mesh] OR "Epoetin Alfa"[Mesh] OR "Iron/therapy"[Mesh] OR "Hypothermia"[Mesh] OR "Hypothermia, Induced"[Mesh] OR blood pressure[tiab] OR hematocrit[tiab] OR haematocrit[tiab] OR hypotension[tiab] OR vasopressor*[tiab] OR vasoconstrict*[tiab] OR inotropic agent*[tiab] OR cardiotonic drug*[tiab] OR fluid therap*[tiab] OR fluid resuscitation*[tiab] OR volume resuscitation*[tiab] OR fluid replacement*[tiab] OR volume replacement*[tiab] OR rehydration therap*[tiab] OR fluid retention*[tiab] OR sodium chlorid*[tiab] OR saline solution*[tiab] OR hypertonic solution*[tiab] OR isotonic solution*[tiab] OR hypotonic solution*[tiab] OR crystalloid*[tiab] OR colloid*[tiab] OR electrolyte*[tiab] OR plasma substitut*[tiab] OR ringer solution*[tiab] OR ringer's solution*[tiab] OR ringer lactat*[tiab] OR blood substitute*[tiab] OR erythrocyte*[tiab] OR erythrocyte transfusion*[tiab] OR epoetin alfa[tiab] OR epoetin alpha[tiab] OR iron therap*[tiab] OR hypotherm*[tiab]) AND humans[mesh] AND ("2015/02/01"[Date - Publication] : "3000"[Date - Publication]) NOT ("Comment" [Publication Type] OR "Letter" [Publication Type] OR "Editorial" [Publication Type])

IV. Rapid control of bleeding

Search performed 31.01.2018 (general)
322 hits

("Wounds and Injuries"[Mesh] OR "Trauma Severity Indices"[Mesh] OR injur*[tiab] OR trauma*[tiab] OR polytrauma*[tiab]) AND ("Hemorrhage"[Mesh] OR "Blood Coagulation Disorders"[Mesh] OR "Disseminated Intravascular Coagulation"[Mesh] OR bleed*[tiab] OR hemorrhag*[tiab] OR haemorrhag*[tiab] OR coagulation disorder*[tiab] OR coagulopath*[tiab] OR disseminated intravascular coagulation[tiab] OR DIC[tiab]) AND (damage control[tiab] OR abdominal packing[tiab] OR "Hemostatics"[Mesh] OR ((hemostatic[tiab] OR haemostatic[tiab]) AND (agent*[tiab] OR drug*[tiab]))) AND humans[mesh] AND ("2015/02/01"[Date - Publication] : "3000"[Date - Publication]) NOT ("Comment" [Publication Type] OR "Letter" [Publication Type] OR "Editorial" [Publication Type])

Search performed 31.01.2018 (pelvic injury)
205 hits

("Wounds and Injuries"[Mesh] OR "Trauma Severity Indices"[Mesh] OR injur*[tiab] OR trauma*[tiab] OR polytrauma*[tiab]) AND ("Hemorrhage"[Mesh] OR "Blood Coagulation Disorders"[Mesh] OR "Disseminated Intravascular Coagulation"[Mesh] OR bleed*[tiab] OR hemorrhag*[tiab] OR haemorrhag*[tiab] OR coagulation disorder*[tiab] OR coagulopath*[tiab] OR disseminated intravascular coagulation[tiab] OR DIC[tiab]) AND ((pelvis [mesh] OR "pelvic bones" [mesh] OR "Pubic Symphysis" [mesh] OR "Sacroiliac Joint" [mesh] OR "hip fractures" [mesh] OR acetabulum [mesh] OR ((pelvic [ti] OR pelvis [ti] OR hip [ti] OR acetabul* [ti] OR pubic* [ti] OR sacroiliac* [ti] OR symphys* [ti]) AND (fracture* [tiab] OR injur* [tiab] OR trauma* [tiab] OR disrupt* [tiab])))) AND humans[mesh] AND ("2015/02/01"[Date - Publication] : "3000"[Date - Publication]) NOT ("Comment" [Publication Type] OR "Letter" [Publication Type] OR "Editorial" [Publication Type])

V. Initial management of bleeding and coagulopathy

Search performed 31.01.2018
1253 hits

("Wounds and Injuries"[Mesh] OR "Trauma Severity Indices"[Mesh] OR injur*[tiab] OR trauma*[tiab] OR polytrauma*[tiab]) AND ("Hemorrhage"[Mesh] OR "Blood Coagulation Disorders"[Mesh] OR "Disseminated Intravascular Coagulation"[Mesh] OR bleed*[tiab] OR hemorrhag*[tiab] OR haemorrhag*[tiab] OR coagulation disorder*[tiab] OR coagulopath*[tiab] OR disseminated intravascular coagulation[tiab] OR DIC[tiab]) AND ("Blood Coagulation"[Majr] OR "Hemostasis"[Majr] OR "Blood Coagulation Tests"[Majr] OR "Plasma"[Mesh] OR "Erythrocyte Transfusion"[Mesh] OR "Platelet Transfusion"[Mesh] OR "Fibrinogen"[Mesh] OR "Antifibrinolytic Agents"[Mesh] OR "Tranexamic Acid"[Mesh] OR coagula*[tiab] OR hemosta*[tiab] OR haemosta*[tiab] OR ((laboratory[tiab] OR coagulation[tiab] OR point of care[tiab] OR platelet function[tiab]) AND (test[tiab] OR tests[tiab] OR testing[tiab] OR parameter[tiab] OR parameters[tiab] OR monitoring[tiab] OR tool[tiab] OR tools[tiab])) OR (viscoelastic[tiab] AND (test[tiab] OR tests[tiab] OR testing[tiab] OR monitoring[tiab])) OR ((plasma[tiab] OR FFP[tiab] OR erythrocyte*[tiab] OR red blood cells[tiab] OR platelet*[tiab] OR thrombocyte*[tiab]) AND transfusion*[tiab]) OR fibrinogen[tiab] OR (antifibrinolytic[tiab] AND (agent*[tiab] OR drug*[tiab])) OR tranexamic acid[tiab]) AND humans[mesh] AND ("2015/02/01"[Date - Publication] : "3000"[Date - Publication]) NOT ("Comment" [Publication Type] OR "Letter" [Publication Type] OR "Editorial" [Publication Type])

VI. FURTHER RESUSCITATION

Search performed 06.02.2018 (anticoagulants)
533 hits

("Wounds and Injuries"[Mesh] OR "Trauma Severity Indices"[Mesh] OR injur*[tiab] OR trauma*[tiab] OR polytrauma*[tiab]) AND ("Hemorrhage"[Mesh] OR "Blood Coagulation Disorders"[Mesh] OR "Disseminated Intravascular Coagulation"[Mesh] OR bleed*[tiab] OR hemorrhag*[tiab] OR haemorrhag*[tiab] OR coagulation disorder*[tiab] OR coagulopath*[tiab] OR disseminated intravascular coagulation[tiab] OR DIC[tiab]) AND ("Platelet Aggregation Inhibitors"[Mesh] OR "Antithrombins"[Mesh] OR "Factor Xa Inhibitors"[Mesh] OR anticoagulan*[tiab] OR DOACs[tiab] OR antiplatelet*[tiab] OR antithrombin*[tiab] OR anti factor Xa[tiab] OR ((platelet[tiab] OR thrombin[tiab] OR factor Xa[tiab]) AND inhibitor*[tiab]) OR rivaroxaban[tiab] OR apixaban[tiab] OR edoxaban[tiab] OR betrixaban[tiab] OR dabigatran[tiab] OR TXA[tiab] OR idarucizumab[tiab] OR PCC[tiab] OR PCCs[tiab] OR prothrombin complex concentrate*[tiab]) AND humans[mesh] AND ("2015/02/01"[Date - Publication] : "3000"[Date - Publication]) NOT ("Comment" [Publication Type] OR "Letter" [Publication Type] OR "Editorial" [Publication Type])

Search performed 06.02.2018 (coagulants)
578 hits

("Wounds and Injuries"[Mesh] OR "Trauma Severity Indices"[Mesh] OR injur*[tiab] OR trauma*[tiab] OR polytrauma*[tiab]) AND ("Hemorrhage"[Mesh] OR "Blood Coagulation Disorders"[Mesh] OR "Disseminated Intravascular Coagulation"[Mesh] OR bleed*[tiab] OR hemorrhag*[tiab] OR haemorrhag*[tiab] OR coagulation disorder*[tiab] OR coagulopath*[tiab] OR disseminated intravascular coagulation[tiab] OR DIC[tiab]) AND ("Fibrinogen"[Mesh] OR "cryoprecipitate coagulum" [Supplementary Concept] OR "Blood Platelets"[Mesh] OR "Platelet Transfusion"[Mesh] OR "Calcium"[Mesh] OR "Calcium Chloride"[Mesh] OR "Hypocalcemia"[Mesh] OR "Deamino Arginine Vasopressin"[Mesh] OR "prothrombin complex concentrates" [Supplementary Concept] OR "recombinant FVIIa" [Supplementary Concept] OR "Intermittent Pneumatic Compression Devices"[Mesh] OR "Stockings, Compression"[Mesh] OR fibrinogen[tiab] OR cryoprecipitate*[tiab] OR ((platelet*[tiab] OR thrombocyte*[tiab]) AND (transfusion*[tiab] OR administration[tiab] OR substitution[tiab])) OR thrombocytopenia[tiab] OR thrombocytopaenia[tiab] OR (calcium[tiab] AND (transfusion*[tiab] OR administration[tiab] OR treatment[tiab])) OR hypocalcemia[tiab] OR hypocalcaemia[tiab] OR desmopressin[tiab] OR PCC[tiab] OR PCCs[tiab] OR prothrombin complex concentrate*[tiab] OR rFVIIa[tiab] OR recombinant factor VIIa[tiab] OR recombinant activated factor VII[tiab] OR recombinant activated coagulation factor VII[tiab] OR FXIII[tiab] OR factor XIII[tiab] OR thromboprophylaxis[tiab] OR intermittent pneumatic compression[tiab] OR stockings[tiab] OR dabigatran[tiab]) AND humans[mesh] AND ("2015/02/01"[Date - Publication] : "3000"[Date - Publication]) NOT ("Comment" [Publication Type] OR "Letter" [Publication Type] OR "Editorial" [Publication Type])

VII. GUIDELINE IMPLEMENTATION AND QUALITY CONTROL

Search performed 31.01.2018
953 hits

("Wounds and Injuries"[Mesh] OR "Trauma Severity Indices"[Mesh] OR injur*[tiab] OR trauma*[tiab] OR polytrauma*[tiab]) AND ("Hemorrhage"[Mesh] OR "Blood Coagulation Disorders"[Mesh] OR "Disseminated Intravascular Coagulation"[Mesh] OR bleed*[tiab] OR hemorrhag*[tiab] OR haemorrhag*[tiab] OR coagulation disorder*[tiab] OR coagulopath*[tiab] OR disseminated intravascular coagulation[tiab] OR DIC[tiab]) AND ("Practice Guidelines as Topic"[Mesh] OR "Clinical Protocols"[Mesh] OR "Guideline Adherence"[Mesh] OR "Evidence-Based Medicine"[Mesh] OR "Evidence-Based Emergency Medicine"[Mesh] OR "Evidence-Based Practice"[Mesh] OR "Patient Care Bundles"[Mesh] OR "Education"[Mesh] OR "Patient Simulation"[Mesh] OR "Practice Patterns, Physicians'"[Mesh] OR guideline*[tiab] OR protocol*[tiab] OR evidence based[tiab] OR evidence-based[tiab] OR checklist*[tiab] OR education*[tiab] OR simulation*[tiab] OR "treatment bundle"[tiab] OR "treatment bundles"[tiab] OR "patient care bundle"[tiab] OR "patient care bundles"[tiab] OR massive transfusion[tiab]) AND humans[mesh] AND ("2015/02/01"[Date - Publication] : "3000"[Date - Publication]) NOT ("Comment" [Publication Type] OR "Letter" [Publication Type] OR "Editorial" [Publication Type])
