# Supplementary material for: The European guideline on management of major bleeding and coagulopathy following trauma: fifth edition
Source: Crit Care. 2019 Mar 27;23:98. doi: 10.1186/s13054-019-2347-3 (PMC6436241; doi:10.1186/s13054-019-2347-3)
Supplement: Supplementary file 2 — Levels of evidence according to [45] for evidence cited in this guideline. (PDF 794 kb) [file 13054_2019_2347_MOESM2_ESM.pdf]

LEVELS OF EVIDENCE

R1 Minimal elapsed time		
**Reference**	**Citation**	**LoE**
1	Celso B, Tepas J, Langland-Orban B, Pracht E, Papa L, Lottenberg L, Flint L: **A systematic review and meta-analysis comparing outcome of severely injured patients treated in trauma centers following the establishment of trauma systems**. *J Trauma* 2006, **60**(2):371-378; discussion 378.	3
2	Hill AD, Fowler RA, Nathens AB: **Impact of interhospital transfer on outcomes for trauma patients: a systematic review**. *J Trauma* 2011, **71**(6):1885-1900; discussion 1901.	3
3	Williams T, Finn J, Fatovich D, Jacobs I: **Outcomes of different health care contexts for direct transport to a trauma center versus initial secondary center care: a systematic review and meta-analysis**. *Prehosp Emerg Care* 2013, **17**(4):442-457.	3
4	Calland JF, Ingraham AM, Martin N, Marshall GT, Schulman CI, Stapleton T, Barraco RD, Eastern Association for the Surgery of T: **Evaluation and management of geriatric trauma: an Eastern Association for the Surgery of Trauma practice management guideline**. *J Trauma Acute Care Surg* 2012, **73**(5 Suppl 4):S345-350.	4
5	Caputo LM, Salottolo KM, Slone DS, Mains CW, Bar-Or D: **The relationship between patient volume and mortality in American trauma centres: a systematic review of the evidence**. *Injury* 2014, **45**(3):478-486.	3
6	Calland JF, Stukenborg GJ: **Trauma centre patient volume and inpatient mortality risk reconsidered**. *Injury* 2016, **47**(5):1072-1077.	2
7	MacKenzie EJ, Rivara FP, Jurkovich GJ, Nathens AB, Frey KP, Egleston BL, Salkever DS, Scharfstein DO: **A national evaluation of the effect of trauma-center care on mortality**. *N Engl J Med* 2006, **354**(4):366-378.	2
8	Cothren CC, Moore EE, Hedegaard HB, Meng K: **Epidemiology of urban trauma deaths: a comprehensive reassessment 10 years later**. *World J Surg* 2007, **31**(7):1507-1511.	4
9	Martin M, Oh J, Currier H, Tai N, Beekley A, Eckert M, Holcomb J: **An analysis of in-hospital deaths at a modern combat support hospital**. *J Trauma* 2009, **66**(4 Suppl):S51-60; discussion S60-51.	4
10	Smith W, Williams A, Agudelo J, Shannon M, Morgan S, Stahel P, Moore E: **Early predictors of mortality in hemodynamically unstable pelvis fractures**. *J Orthop Trauma* 2007, **21**(1):31-37.	3
11	Harmsen AM, Giannakopoulos GF, Moerbeek PR, Jansma EP, Bonjer HJ, Bloemers FW: **The influence of prehospital time on trauma patients outcome: a systematic review**. *Injury* 2015, **46**(4):602-609.	3

**R2 Local bleeding management**		
**Reference**	**Citation**	**LoE**
1	Singh RA, Asprou F, Patel A, Trickett RW: **Haemorrhage control in extremity stab injury**. *J Surg Case Rep* 2013, **2013**(12).	3
2	Demetriades D, Asensio JA, Velmahos G, Thal E: **Complex problems in penetrating neck trauma**. *Surg Clin North Am* 1996, **76**(4):661-683.	3
3	Van Waes OJ, Cheriex KC, Navsaria PH, van Riet PA, Nicol AJ, Vermeulen J: **Management of penetrating neck injuries**. *Br J Surg* 2012, **99 Suppl 1**:149-154.	3
4	Lakstein D, Blumenfeld A, Sokolov T, Lin G, Bssorai R, Lynn M, Ben-Abraham R: **Tourniquets for hemorrhage control on the battlefield: a 4-year accumulated experience**. *J Trauma* 2003, **54**(5 Suppl):S221-225.	2
5	Beekley AC, Sebesta JA, Blackbourne LH, Herbert GS, Kauvar DS, Baer DG, Walters TJ, Mullenix PS, Holcomb JB, st Combat Support Hospital Research G: **Prehospital tourniquet use in Operation Iraqi Freedom: effect on hemorrhage control and outcomes**. *J Trauma* 2008, **64**(2 Suppl):S28-37; discussion S37.	2
6	Brodie S, Hodgetts TJ, Ollerton J, McLeod J, Lambert P, Mahoney P: **Tourniquet use in combat trauma: UK military experience**. *J R Army Med Corps* 2007, **153**(4):310-313.	3
7	Kragh JF, Jr., Walters TJ, Baer DG, Fox CJ, Wade CE, Salinas J, Holcomb JB: **Survival with emergency tourniquet use to stop bleeding in major limb trauma**. *Ann Surg* 2009, **249**(1):1-7.	3
8	Swan KG, Jr., Wright DS, Barbagiovanni SS, Swan BC, Swan KG: **Tourniquets revisited**. *J Trauma* 2009, **66**(3):672-675.	4
9	Kragh JF, Jr., O'Neill ML, Walters TJ, Jones JA, Baer DG, Gershman LK, Wade CE, Holcomb JB: **Minor morbidity with emergency tourniquet use to stop bleeding in severe limb trauma: research, history, and reconciling advocates and abolitionists**. *Mil Med* 2011, **176**(7):817-823.	4
10	Kragh JF, Jr., Cooper A, Aden JK, Dubick MA, Baer DG, Wade CE, Blackbourne LH: **Survey of trauma registry data on tourniquet use in pediatric war casualties**. *Pediatr Emerg Care* 2012, **28**(12):1361-1365.	3
11	Dayan L, Zinmann C, Stahl S, Norman D: **Complications associated with prolonged tourniquet application on the battlefield**. *Mil Med* 2008, **173**(1):63-66.	2
12	Bulger EM, Snyder D, Schoelles K, Gotschall C, Dawson D, Lang E, Sanddal ND, Butler FK, Fallat M, Taillac P *et al*: **An evidence-based prehospital guideline for external hemorrhage control: American College of Surgeons Committee on Trauma**. *Prehosp Emerg Care* 2014, **18**(2):163-173.	3
13	Fox N, Rajani RR, Bokhari F, Chiu WC, Kerwin A, Seamon MJ, Skarupa D, Frykberg E, Eastern Association for the Surgery of T: **Evaluation and management of penetrating lower extremity arterial trauma: an Eastern Association for the Surgery of Trauma practice management guideline**. *J Trauma Acute Care Surg* 2012, **73**(5 Suppl 4):S315-320.	3
14	Coccolini F, Stahel PF, Montori G, Biffl W, Horer TM, Catena F, Kluger Y, Moore EE, Peitzman AB, Ivatury R *et al*: **Pelvic trauma: WSES classification and guidelines**. *World J Emerg Surg* 2017, **12**:5.	4
15	Ruatti S, Guillot S, Brun J, Thony F, Bouzat P, Payen JF, Tonetti J: **Which pelvic ring fractures are potentially lethal?** *Injury* 2015, **46**(6):1059-1063.	3
16	Skitch S, Engels PT: **Acute management of the traumatically injured pelvis**. *Emerg Med Clin North Am* 2018, **36**(1):161-179.	4
17	Chao NS, Liu CS, Chung KL, Tang PM, Tai DK, Lee KY, Chang A, Leung MW, Liu KK: **Retroperitoneal pelvic packing for haemodynamically unstable pelvic fractures in children and adolescents: a level-one trauma-centre experience**. *J Pediatr Surg* 2012, **47**(12):2244-2250.	4
18	Hauschild O, Aghayev E, von Heyden J, Strohm PC, Culemann U, Pohlemann T, Suedkamp NP, Schmal H: **Angioembolization for pelvic hemorrhage control: results from the German pelvic injury register**. *J Trauma Acute Care Surg* 2012, **73**(3):679-684.	3

**R2 Local bleeding management**		
**Reference**	**Citation**	**LoE**
19	Heetveld MJ, Harris I, Schlaphoff G, Balogh Z, D'Amours SK, Sugrue M: **Hemodynamically unstable pelvic fractures: recent care and new guidelines**. *World J Surg* 2004, **28**(9):904-909.	4
20	Rudloff MI, Triantafillou KM: **Management of pelvic ring injuries in unstable patients**. *Orthop Clin North Am* 2016, **47**(3):551-563.	4
21	Schweigkofler U, Wohlrath B, Paffrath T, Flohe S, Wincheringer D, Hoffmann R, Trentzsch H: **[Recommendations for Releasing the Pelvic Binder After a Non-Invasive Pelvic Stabilisation Procedure Under Emergency Room Conditions]**. *Z Orthop Unfall* 2016, **154**(5):470-476.	4
22	Wohlrath B, Trentzsch H, Hoffmann R, Kremer M, Schmidt-Horlohe K, Schweigkofler U: **[Preclinical and clinical treatment of instable pelvic injuries : Results of an online survey]**. *Unfallchirurg* 2016, **119**(9):755-762.	4
23	Poenaru DV, Popescu M, Anglitoiu B, Popa I, Andrei D, Birsasteanu F: **Emergency pelvic stabilization in patients with pelvic posttraumatic instability**. *Int Orthop* 2015, **39**(5):961-965.	4
24	Li Q, Dong J, Yang Y, Wang G, Wang Y, Liu P, Robinson Y, Zhou D: **Retroperitoneal packing or angioembolization for haemorrhage control of pelvic fractures--Quasi-randomized clinical trial of 56 haemodynamically unstable patients with Injury Severity Score >/=33**. *Injury* 2016, **47**(2):395-401.	3
25	Hornez E, Monchal T, Boddaert G, Chiron P, Danis J, Baudoin Y, Daban JL, Balandraud P, Bonnet S: **Penetrating pelvic trauma: Initial assessment and surgical management in emergency**. *J Visc Surg* 2016, **153**(4 Suppl):79-90.	4

**R3 Ventilation**		
**Reference**	**Citation**	**LoE**
1	Mayglothling J, Duane TM, Gibbs M, McCunn M, Legome E, Eastman AL, Whelan J, Shah KH, Eastern Association for the Surgery of Trauma: **Emergency tracheal intubation immediately following traumatic injury: an Eastern Association for the Surgery of Trauma practice management guideline**. *J Trauma Acute Care Surg* 2012, **73**(5 Suppl 4):S333-340.	4
2	Shafi S, Gentilello L: **Pre-hospital endotracheal intubation and positive pressure ventilation is associated with hypotension and decreased survival in hypovolemic trauma patients: an analysis of the National Trauma Data Bank**. *J Trauma* 2005, **59**(5):1140-1145; discussion 1145-1147.	4
3	Bukur M, Kurtovic S, Berry C, Tanios M, Margulies DR, Ley EJ, Salim A: **Pre-hospital intubation is associated with increased mortality after traumatic brain injury**. *J Surg Res* 2011, **170**(1):e117-121.	4
4	Bernard SA, Nguyen V, Cameron P, Masci K, Fitzgerald M, Cooper DJ, Walker T, Std BP, Myles P, Murray L *et al*: **Prehospital rapid sequence intubation improves functional outcome for patients with severe traumatic brain injury: a randomized controlled trial**. *Ann Surg* 2010, **252**(6):959-965.	2
5	Boer C, Franschman G, Loer SA: **Prehospital management of severe traumatic brain injury: concepts and ongoing controversies**. *Curr Opin Anaesthesiol* 2012, **25**(5):556-562.	5
6	Jeremitsky E, Omert L, Dunham CM, Protetch J, Rodriguez A: **Harbingers of poor outcome the day after severe brain injury: hypothermia, hypoxia, and hypoperfusion**. *J Trauma* 2003, **54**(2):312-319.	3
7	Chi JH, Knudson MM, Vassar MJ, McCarthy MC, Shapiro MB, Mallet S, Holcroft JJ, Moncrief H, Noble J, Wisner D *et al*: **Prehospital hypoxia affects outcome in patients with traumatic brain injury: a prospective multicenter study**. *J Trauma* 2006, **61**(5):1134-1141.	4
8	Damiani E, Adrario E, Girardis M, Romano R, Pelaia P, Singer M, Donati A: **Arterial hyperoxia and mortality in critically ill patients: a systematic review and meta-analysis**. *Crit Care* 2014, **18**(6):711.	2
9	Page D, Ablordeppey E, Wessman BT, Mohr NM, Trzeciak S, Kollef MH, Roberts BW, Fuller BM: **Emergency department hyperoxia is associated with increased mortality in mechanically ventilated patients: a cohort study**. *Crit Care* 2018, **22**(1):9.	2
10	Chu DK, Kim LH, Young PJ, Zamiri N, Almenawer SA, Jaeschke R, Szczeklik W, Schunemann HJ, Neary JD, Alhazzani W: **Mortality and morbidity in acutely ill adults treated with liberal versus conservative oxygen therapy (IOTA): a systematic review and meta-analysis**. *Lancet* 2018, **391**(10131):1693-1705.	1
11	Vincent JL, Taccone FS, He X: **Harmful effects of hyperoxia in postcardiac arrest, sepsis, traumatic brain injury, or stroke: The importance of individualized oxygen therapy in critically ill patients**. *Can Respir J* 2017, **2017**:2834956.	3
12	Aggarwal NR, Brower RG, Hager DN, Thompson BT, Netzer G, Shanholtz C, Lagakos A, Checkley W, National Institutes of Health Acute Respiratory Distress Syndrome Network I: **Oxygen exposure resulting in arterial oxygen tensions above the protocol goal was associated with worse clinical outcomes in acute respiratory distress syndrome**. *Crit Care Med* 2018, **46**(4):517-524.	2
13	Panwar R, Hardie M, Bellomo R, Barrot L, Eastwood GM, Young PJ, Capellier G, Harrigan PW, Bailey M, Investigators CS *et al*: **Conservative versus liberal oxygenation targets for mechanically ventilated patients. A pilot multicenter randomized controlled trial**. *Am J Respir Crit Care Med* 2016, **193**(1):43-51.	2
14	Brugniaux JV, Coombs GB, Barak OF, Dujic Z, Sekhon MS, Ainslie PN: **Highs and lows of hyperoxia: physiological, performance, and clinical aspects**. *Am J Physiol Regul Integr Comp Physiol* 2018.	3

**R3 Ventilation**		
**Reference**	**Citation**	**LoE**
15	Smit B, Smulders YM, van der Wouden JC, Oudemans-van Straaten HM, Spoelstra-de Man AME: **Hemodynamic effects of acute hyperoxia: systematic review and meta-analysis**. *Crit Care* 2018, **22**(1):45.	2
16	Harutyunyan G, Harutyunyan G, Mkhoyan G: **New viewpoint in exaggerated increase of PtiO2 with normobaric hyperoxygenation and reasons to limit oxygen use in neurotrauma patients**. *Front Med (Lausanne)* 2018, **5**:119.	4
17	Aufderheide TP, Sigurdsson G, Pirrallo RG, Yannopoulos D, McKnite S, von Briesen C, Sparks CW, Conrad CJ, Provo TA, Lurie KG: **Hyperventilation-induced hypotension during cardiopulmonary resuscitation**. *Circulation* 2004, **109**(16):1960-1965.	3
18	Davis DP, Hoyt DB, Ochs M, Fortlage D, Holbrook T, Marshall LK, Rosen P: **The effect of paramedic rapid sequence intubation on outcome in patients with severe traumatic brain injury**. *J Trauma* 2003, **54**(3):444-453.	3
19	Manley GT, Hemphill JC, Morabito D, Derugin N, Erickson V, Pitts LH, Knudson MM: **Cerebral oxygenation during hemorrhagic shock: perils of hyperventilation and the therapeutic potential of hypoventilation**. *J Trauma* 2000, **48**(6):1025-1032; discussion 1032-1023.	3
20	Blomgren K, Zhu C, Hallin U, Hagberg H: **Mitochondria and ischemic reperfusion damage in the adult and in the developing brain**. *Biochem Biophys Res Commun* 2003, **304**(3):551-559.	4
21	Davis DP: **Early ventilation in traumatic brain injury**. *Resuscitation* 2008, **76**(3):333-340.	3
22	Davis DP, Idris AH, Sise MJ, Kennedy F, Eastman AB, Velky T, Vilke GM, Hoyt DB: **Early ventilation and outcome in patients with moderate to severe traumatic brain injury**. *Crit Care Med* 2006, **34**(4):1202-1208.	3
23	Curley G, Kavanagh BP, Laffey JG: **Hypocapnia and the injured brain: more harm than benefit**. *Crit Care Med* 2010, **38**(5):1348-1359.	3
24	Stevens RD, Shoykhet M, Cadena R: **Emergency neurological life support: Intracranial hypertension and herniation**. *Neurocrit Care* 2015, **23 Suppl 2**:76-82.	3
25	The Acute Respiratory Distress Syndrome Network, Brower RG, Matthay MA, Morris A, Schoenfeld D, Thompson BT, Wheeler A: **Ventilation with lower tidal volumes as compared with traditional tidal volumes for acute lung injury and the acute respiratory distress syndrome**. *N Engl J Med* 2000, **342**(18):1301-1308.	1
26	Wolthuis EK, Choi G, Dessing MC, Bresser P, Lutter R, Dzoljic M, van der Poll T, Vroom MB, Hollmann M, Schultz MJ: **Mechanical ventilation with lower tidal volumes and positive end-expiratory pressure prevents pulmonary inflammation in patients without preexisting lung injury**. *Anesthesiology* 2008, **108**(1):46-54.	2

**R4 Initial assessment**		
**Reference**	**Citation**	**LoE**
1	Cantle PM, Cotton BA: **Prediction of massive transfusion in trauma**. *Crit Care Clin* 2017, **33**(1):71-84.	5
2	Foster JC, Sappenfield JW, Smith RS, Kiley SP: **Initiation and termination of massive transfusion protocols: Current strategies and future prospects**. *Anesth Analg* 2017, **125**(6):2045-2055.	5
3	Frank M, Schmucker U, Stengel D, Fischer L, Lange J, Grossjohann R, Ekkernkamp A, Matthes G: **Proper estimation of blood loss on scene of trauma: tool or tale?** *J Trauma* 2010, **69**(5):1191-1195.	2
4	Liu CC, Wang CY, Shih HC, Wen YS, Wu JJ, Huang CI, Hsu HS, Huang MH, Huang MS: **Prognostic factors for mortality following falls from height**. *Injury* 2009, **40**(6):595-597.	4
5	American College of Surgeons Committee on Trauma: **ATLS® Student Manual 10th Edition** Chicago, IL: American College of Surgeons; 2018.	5
6	Cinelli SM, Brady P, Rennie CP, Tuluca C, Hall TS: **Comparative results of trauma scoring systems in fatal outcomes**. *Conn Med* 2009, **73**(5):261-265.	4
7	Moore L, Lavoie A, Turgeon AF, Abdous B, Le Sage N, Emond M, Liberman M, Bergeron E: **The trauma risk adjustment model: a new model for evaluating trauma care**. *Ann Surg* 2009, **249**(6):1040-1046.	3
8	Narci A, Solak O, Turhan-Haktanir N, Aycicek A, Demir Y, Ela Y, Ozkaraca E, Terzi Y: **The prognostic importance of trauma scoring systems in pediatric patients**. *Pediatr Surg Int* 2009, **25**(1):25-30.	4
9	Lawton LD, Roncal S, Leonard E, Stack A, Dinh MM, Byrne CM, Petchell J: **The utility of Advanced Trauma Life Support (ATLS) clinical shock grading in assessment of trauma**. *Emerg Med J* 2014, **31**(5):384-389.	3
10	Mutschler M, Paffrath T, Wolfl C, Probst C, Nienaber U, Schipper IB, Bouillon B, Maegele M: **The ATLS((R)) classification of hypovolaemic shock: A well established teaching tool on the edge?** *Injury* 2014, **45 Suppl 3**:S35-38.	5
11	Mutschler M, Nienaber U, Brockamp T, Wafaisade A, Wyen H, Peiniger S, Paffrath T, Bouillon B, Maegele M, TraumaRegister DGU: **A critical reappraisal of the ATLS classification of hypovolaemic shock: does it really reflect clinical reality?** *Resuscitation* 2013, **84**(3):309-313.	3
12	Guly HR, Bouamra O, Spiers M, Dark P, Coats T, Lecky FE: **Vital signs and estimated blood loss in patients with major trauma: testing the validity of the ATLS classification of hypovolaemic shock**. *Resuscitation* 2011, **82**(5):556-559.	3
13	Fligor SC, Hamill ME, Love KM, Collier BR, Lollar D, Bradburn EH: **Vital signs strongly predict massive transfusion need in geriatric trauma patients**. *Am Surg* 2016, **82**(7):632-636.	4
14	DeMuro JP, Simmons S, Jax J, Gianelli SM: **Application of the Shock Index to the prediction of need for hemostasis intervention**. *Am J Emerg Med* 2013, **31**(8):1260-1263.	3
15	Olaussen A, Blackburn T, Mitra B, Fitzgerald M: **Review article: shock index for prediction of critical bleeding post-trauma: a systematic review**. *Emerg Med Australas* 2014, **26**(3):223-228.	2
16	Paladino L, Subramanian RA, Nabors S, Sinert R: **The utility of shock index in differentiating major from minor injury**. *Eur J Emerg Med* 2011, **18**(2):94-98.	4
17	Mutschler M, Nienaber U, Brockamp T, Wafaisade A, Fabian T, Paffrath T, Bouillon B, Maegele M, TraumaRegister DGU: **Renaissance of base deficit for the initial assessment of trauma patients: a base deficit-based classification for hypovolemic shock developed on data from 16,305 patients derived from the TraumaRegister DGU(R)**. *Crit Care* 2013, **17**(2):R42.	3
18	Lai WH, Wu SC, Rau CS, Kuo PJ, Hsu SY, Chen YC, Hsieh HY, Hsieh CH: **Systolic blood pressure lower than heart rate upon arrival at and**	3

**R4 Initial assessment**		
**Reference**	**Citation**	**LoE**
	**departure from the emergency department indicates a poor outcome for adult trauma patients**. *Int J Environ Res Public Health* 2016, **13**(6).	
19	Brockamp T, Nienaber U, Mutschler M, Wafaisade A, Peiniger S, Lefering R, Bouillon B, Maegele M, TraumaRegister DGU: **Predicting on-going hemorrhage and transfusion requirement after severe trauma: a validation of six scoring systems and algorithms on the TraumaRegister DGU**. *Crit Care* 2012, **16**(4):R129.	3
20	Maegele M: **Frequency, risk stratification and therapeutic management of acute post-traumatic coagulopathy**. *Vox Sang* 2009, **97**(1):39-49.	3
21	Maegele M, Paffrath T, Bouillon B: **Acute traumatic coagulopathy in severe injury: incidence, risk stratification, and treatment options**. *Dtsch Arztebl Int* 2011, **108**(49):827-835.	5
22	Mitra B, Cameron PA, Mori A, Maini A, Fitzgerald M, Paul E, Street A: **Early prediction of acute traumatic coagulopathy**. *Resuscitation* 2011, **82**(9):1208-1213.	4
23	Mutschler M, Brockamp T, Wafaisade A, Lipensky A, Probst C, Bouillon B, Maegele M: **'Time to TASH': how long does complete score calculation take to assess major trauma hemorrhage?** *Transfus Med* 2014, **24**(1):58-59.	2
24	Ogura T, Lefor AK, Masuda M, Kushimoto S: **Modified traumatic bleeding severity score: early determination of the need for massive transfusion**. *Am J Emerg Med* 2016, **34**(6):1097-1101.	4
25	Peltan ID, Rowhani-Rahbar A, Vande Vusse LK, Caldwell E, Rea TD, Maier RV, Watkins TR: **Development and validation of a prehospital prediction model for acute traumatic coagulopathy**. *Crit Care* 2016, **20**(1):371.	4

R5 Immediate intervention		
**Reference**	**Citation**	**LoE**
1	Jackson MR, Olson DW, Beckett WC, Jr., Olsen SB, Robertson FM: **Abdominal vascular trauma: a review of 106 injuries**. *Am Surg* 1992, **58**(10):622-626.	3
2	Johnson JW, Gracias VH, Schwab CW, Reilly PM, Kauder DR, Shapiro MB, Dabrowski GP, Rotondo MF: **Evolution in damage control for exsanguinating penetrating abdominal injury**. *J Trauma* 2001, **51**(2):261-269; discussion 269-271.	4
3	Billy LJ, Amato JJ, Rich NM: **Aortic injuries in Vietnam**. *Surgery* 1971, **70**(3):385-391.	3
4	Dean NR, Ledgard JP, Katsaros J: **Massive hemorrhage in facial fracture patients: definition, incidence, and management**. *Plast Reconstr Surg* 2009, **123**(2):680-690.	4
5	Frakes MA, Evans T: **Major pelvic fractures**. *Crit Care Nurse* 2004, **24**(2):18-30; quiz 31-12.	3
6	Grotz MR, Gummerson NW, Gansslen A, Petrowsky H, Keel M, Allami MK, Tzioupis C, Trentz O, Krettek C, Pape HC *et al*: **Staged management and outcome of combined pelvic and liver trauma. An international experience of the deadly duo**. *Injury* 2006, **37**(7):642-651.	4
7	Cryer HM, Miller FB, Evers BM, Rouben LR, Seligson DL: **Pelvic fracture classification: correlation with hemorrhage**. *J Trauma* 1988, **28**(7):973-980.	3
8	Burgess AR, Eastridge BJ, Young JW, Ellison TS, Ellison PS, Jr., Poka A, Bathon GH, Brumback RJ: **Pelvic ring disruptions: effective classification system and treatment protocols**. *J Trauma* 1990, **30**(7):848-856.	3
9	Eastridge BJ, Starr A, Minei JP, O'Keefe GE, Scalea TM: **The importance of fracture pattern in guiding therapeutic decision-making in patients with hemorrhagic shock and pelvic ring disruptions**. *J Trauma* 2002, **53**(3):446-450; discussion 450-441.	3
10	Manson TT, Nascone JW, O'Toole RV: **Traction vertical shear pelvic ring fracture: a marker for severe arterial injury? A case report**. *J Orthop Trauma* 2010, **24**(10):e90-94.	4
11	Karmy-Jones R, Jurkovich GJ, Shatz DV, Brundage S, Wall MJ, Jr., Engelhardt S, Hoyt DB, Holcroft J, Knudson MM: **Management of traumatic lung injury: a Western Trauma Association multicenter review**. *J Trauma* 2001, **51**(6):1049-1053.	4
12	de Lesquen H, Avaro J-P, Gust L, Ford RM, Beranger F, Natale C, Bonnet P-M, D'Journo X-B: **Surgical management for the first 48 h following blunt chest trauma: state of the art (excluding vascular injuries)**. *Interactive CardioVascular and Thoracic Surgery* 2015, **20**(3):399-408.	4

**R6 Further investigation**		
**Reference**	**Citation**	**LoE**
1	Gillman LM, Ball CG, Panebianco N, Al-Kadi A, Kirkpatrick AW: **Clinician performed resuscitative ultrasonography for the initial evaluation and resuscitation of trauma**. *Scand J Trauma Resusc Emerg Med* 2009, **17**:34.	5
2	American College of Surgeons Committee on Trauma: **ATLS® Student Manual 9th Edition** Chicago, IL: American College of Surgeons; 2012.	5
3	Stahel PF, Heyde CE, Wyrwich W, Ertel W: **[Current concepts of polytrauma management: from ATLS to "damage control"]**. *Orthopade* 2005, **34**(9):823-836.	5
4	Gebhard F, Huber-Lang M: **Polytrauma--pathophysiology and management principles**. *Langenbecks Arch Surg* 2008, **393**(6):825-831.	5
5	Huber-Wagner S, Lefering R, Qvick LM, Korner M, Kay MV, Pfeifer KJ, Reiser M, Mutschler W, Kanz KG: **Effect of whole-body CT during trauma resuscitation on survival: a retrospective, multicentre study**. *Lancet* 2009, **373**(9673):1455-1461.	3
6	Albrecht T, von Schlippenbach J, Stahel PF, Ertel W, Wolf KJ: **[The role of whole body spiral CT in the primary work-up of polytrauma patients--comparison with conventional radiography and abdominal sonography]**. *Rofo* 2004, **176**(8):1142-1150.	2
7	Linsenmaier U, Krotz M, Hauser H, Rock C, Rieger J, Bohndorf K, Pfeifer KJ, Reiser M: **Whole-body computed tomography in polytrauma: techniques and management**. *Eur Radiol* 2002, **12**(7):1728-1740.	5
8	Huber-Wagner S, Mand C, Ruchholtz S, Kuhne CA, Holzapfel K, Kanz KG, van Griensven M, Biberthaler P, Lefering R, TraumaRegister DGU: **Effect of the localisation of the CT scanner during trauma resuscitation on survival -- a retrospective, multicentre study**. *Injury* 2014, **45 Suppl 3**:S76-82.	3
9	Huber-Wagner S, Biberthaler P, Haberle S, Wierer M, Dobritz M, Rummeny E, van Griensven M, Kanz KG, Lefering R, TraumaRegister DGU: **Whole-body CT in haemodynamically unstable severely injured patients--a retrospective, multicentre study**. *PLoS One* 2013, **8**(7):e68880.	3
10	Kim YJ, Kim JS, Cho SH, Bae JI, Sohn CH, Lee YS, Lee JH, Lim KS, Kim WY: **Characteristics of computed tomography in hemodynamically unstable blunt trauma patients: Experience at a tertiary care center**. *Medicine (Baltimore)* 2017, **96**(49):e9168.	4

**R7 Imaging**		
**Reference**	**Citation**	**LoE**
1	Wongwaisayawan S, Suwannanon R, Prachanukool T, Sricharoen P, Saksobhavivat N, Kaewlai R: **Trauma ultrasound**. *Ultrasound Med Biol* 2015, **41**(10):2543-2561.	5
2	Schieb E, Greim CA: **[Emergency sonography]**. *Anaesthesist* 2015, **64**(4):329-342; quiz 343-324.	5
3	Brenchley J, Walker A, Sloan JP, Hassan TB, Venables H: **Evaluation of focussed assessment with sonography in trauma (FAST) by UK emergency physicians**. *Emerg Med J* 2006, **23**(6):446-448.	3
4	Kretschmer KH, Hauser H: **[Radiologic diagnosis of abdominal trauma]**. *Radiologe* 1998, **38**(8):693-701.	5
5	Rozycki GS, Newman PG: **Surgeon-performed ultrasound for the assessment of abdominal injuries**. *Adv Surg* 1999, **33**:243-259.	3
6	Richards JR, Knopf NA, Wang L, McGahan JP: **Blunt abdominal trauma in children: evaluation with emergency US**. *Radiology* 2002, **222**(3):749-754.	4
7	Richards JR, Schleper NH, Woo BD, Bohnen PA, McGahan JP: **Sonographic assessment of blunt abdominal trauma: a 4-year prospective study**. *J Clin Ultrasound* 2002, **30**(2):59-67.	2
8	Rose JS, Levitt MA, Porter J, Hutson A, Greenholtz J, Nobay F, Hilty W: **Does the presence of ultrasound really affect computed tomographic scan use? A prospective randomized trial of ultrasound in trauma**. *J Trauma* 2001, **51**(3):545-550.	2
9	Shackford SR, Rogers FB, Osler TM, Trabulsy ME, Clauss DW, Vane DW: **Focused abdominal sonogram for trauma: the learning curve of nonradiologist clinicians in detecting hemoperitoneum**. *J Trauma* 1999, **46**(4):553-562; discussion 562-554.	3
10	Stengel D, Bauwens K, Porzsolt F, Rademacher G, Mutze S, Ekkernkamp A: **[Emergency ultrasound for blunt abdominal trauma--meta-analysis update 2003]**. *Zentralbl Chir* 2003, **128**(12):1027-1037.	1
11	Stengel D, Bauwens K, Rademacher G, Mutze S, Ekkernkamp A: **Association between compliance with methodological standards of diagnostic research and reported test accuracy: meta-analysis of focused assessment of US for trauma**. *Radiology* 2005, **236**(1):102-111.	1
12	Stengel D, Bauwens K, Sehouli J, Porzsolt F, Rademacher G, Mutze S, Ekkernkamp A: **Systematic review and meta-analysis of emergency ultrasonography for blunt abdominal trauma**. *Br J Surg* 2001, **88**(7):901-912.	1
13	Quinn AC, Sinert R: **What is the utility of the Focused Assessment with Sonography in Trauma (FAST) exam in penetrating torso trauma?** *Injury* 2011, **42**(5):482-487.	5
14	Dammers D, El Moumni M, Hoogland, II, Veeger N, Ter Avest E: **Should we perform a FAST exam in haemodynamically stable patients presenting after blunt abdominal injury: a retrospective cohort study**. *Scand J Trauma Resusc Emerg Med* 2017, **25**(1):1.	3
15	Fox JC, Boysen M, Gharahbaghian L, Cusick S, Ahmed SS, Anderson CL, Lekawa M, Langdorf MI: **Test characteristics of focused assessment of sonography for trauma for clinically significant abdominal free fluid in pediatric blunt abdominal trauma**. *Acad Emerg Med* 2011, **18**(5):477-482.	5
16	Liu M, Lee CH, P'Eng F K: **Prospective comparison of diagnostic peritoneal lavage, computed tomographic scanning, and ultrasonography for the diagnosis of blunt abdominal trauma**. *J Trauma* 1993, **35**(2):267-270.	4
17	Heyn J, Ladurner R, Ozimek A, Burklein D, Huber-Wagner SM, Hallfeldt KK, Mussack T: **Diagnosis and pre-operative management of multiple injured patients with explorative laparotomy because of blunt abdominal trauma**. *Eur J Med Res* 2008, **13**(11):517-524.	3
18	Rozycki GS, Ballard RB, Feliciano DV, Schmidt JA, Pennington SD: **Surgeon-performed ultrasound for the assessment of truncal injuries: lessons learned from 1540 patients**. *Ann Surg* 1998, **228**(4):557-567.	4

**R7 Imaging**		
**Reference**	**Citation**	**LoE**
19	Patwa AS, Cipot S, Lomibao A, Nelson M, Bramante R, Modayil V, Haines C, Ash A, Raio C: **Prevalence of the "double-line" sign when performing focused assessment with sonography in trauma (FAST) examinations**. *Intern Emerg Med* 2015, **10**(6):721-724.	2
20	Carter JW, Falco MH, Chopko MS, Flynn WJ, Jr., Wiles Iii CE, Guo WA: **Do we really rely on fast for decision-making in the management of blunt abdominal trauma?** *Injury* 2015, **46**(5):817-821.	4
21	Stengel D, Rademacher G, Ekkernkamp A, Guthoff C, Mutze S: **Emergency ultrasound-based algorithms for diagnosing blunt abdominal trauma**. *Cochrane Database Syst Rev* 2015(9):CD004446.	1
22	Schneck E, Koch C, Borgards M, Reichert M, Hecker A, Heiss C, Padberg W, Alejandre-Lafont E, Rohrig R, Krombach GA *et al*: **Impact of abdominal follow-up sonography in trauma patients without abdominal parenchymal organ lesion or free intraabdominal fluid in whole-body computed tomography**. *Rofo* 2017, **189**(2):128-136.	4
23	Miele V, Piccolo CL, Galluzzo M, Ianniello S, Sessa B, Trinci M: **Contrast-enhanced ultrasound (CEUS) in blunt abdominal trauma**. *Br J Radiol* 2016, **89**(1061):20150823.	5
24	Atri M, Hanson JM, Grinblat L, Brofman N, Chughtai T, Tomlinson G: **Surgically important bowel and/or mesenteric injury in blunt trauma: accuracy of multidetector CT for evaluation**. *Radiology* 2008, **249**(2):524-533.	2
25	Becker CD, Poletti PA: **The trauma concept: the role of MDCT in the diagnosis and management of visceral injuries**. *Eur Radiol* 2005, **15 Suppl 4**:D105-109.	5
26	Boehm T, Alkadhi H, Schertler T, Baumert B, Roos J, Marincek B, Wildermuth S: **[Application of multislice spiral CT (MSCT) in multiple injured patients and its effect on diagnostic and therapeutic algorithms]**. *Rofo* 2004, **176**(12):1734-1742.	5
27	Gunn ML, Kool DR, Lehnert BE: **Improving outcomes in the patient with polytrauma: A review of the role of whole-body computed tomography**. *Radiol Clin North Am* 2015, **53**(4):639-656, vii.	5
28	Heyer CM, Rduch G, Kagel T, Lemburg SP, Theisinger A, Bauer TT, Muhr G, Nicolas V: **[Prospective randomized trial of a modified standard multislice CT protocol for the evaluation of multiple trauma patients]**. *Rofo* 2005, **177**(2):242-249.	1
29	Huber-Wagner S, Biberthaler P, Haberle S, Wierer M, Dobritz M, Rummeny E, van Griensven M, Kanz KG, Lefering R, TraumaRegister DGU: **Whole-body CT in haemodynamically unstable severely injured patients--a retrospective, multicentre study**. *PLoS One* 2013, **8**(7):e68880.	3
30	Marmery H, Shanmuganathan K: **Multidetector-row computed tomography imaging of splenic trauma**. *Semin Ultrasound CT MR* 2006, **27**(5):404-419.	2
31	Navarrete-Navarro P, Vazquez G, Bosch JM, Fernandez E, Rivera R, Carazo E: **Computed tomography vs clinical and multidisciplinary procedures for early evaluation of severe abdomen and chest trauma--a cost analysis approach**. *Intensive Care Med* 1996, **22**(3):208-212.	1
32	Rohrl B, Sadick M, Diehl S, Obertacke U, Duber C: **[Whole-body MSCT of patients after polytrauma: abdominal injuries]**. *Rofo* 2005, **177**(12):1641-1648.	4
33	Weninger P, Mauritz W, Fridrich P, Spitaler R, Figl M, Kern B, Hertz H: **Emergency room management of patients with blunt major trauma: evaluation of the multislice computed tomography protocol exemplified by an urban trauma center**. *J Trauma* 2007, **62**(3):584-591.	4
34	Baghdanian AH, Armetta AS, Baghdanian AA, LeBedis CA, Anderson SW, Soto JA: **CT of major vascular injury in blunt abdominopelvic trauma**. *Radiographics* 2016, **36**(3):872-890.	5

**R7 Imaging**		
**Reference**	**Citation**	**LoE**
35	Frandon J, Arvieux C, Thony F: **Indications for embolization in a French level 1 trauma center**. *J Visc Surg* 2016, **153**(4 Suppl):25-31.	4
36	Hornez E, Monchal T, Boddaert G, Chiron P, Danis J, Baudoin Y, Daban JL, Balandraud P, Bonnet S: **Penetrating pelvic trauma: Initial assessment and surgical management in emergency**. *J Visc Surg* 2016, **153**(4 Suppl):79-90.	5
37	Verbeek DO, Burgess AR: **Importance of pelvic radiography for initial trauma assessment: An orthopedic perspective**. *J Emerg Med* 2016, **50**(6):852-858.	4
38	Nguyen BM, Plurad D, Abrishami S, Neville A, Putnam B, Kim DY: **Utility of chest computed tomography after a "normal" chest radiograph in patients with thoracic stab wounds**. *Am Surg* 2015, **81**(10):965-968.	4
39	Cinquantini F, Tugnoli G, Piccinini A, Coniglio C, Mannone S, Biscardi A, Gordini G, Di Saverio S: **Educational review of predictive value and findings of computed tomography scan in diagnosing bowel and mesenteric injuries after blunt trauma: Correlation with trauma surgery findings in 163 patients**. *Can Assoc Radiol J* 2017, **68**(3):276-285.	4
40	Steenburg SD, Petersen MJ, Shen C, Lin H: **Multi-detector CT of blunt mesenteric injuries: usefulness of imaging findings for predicting surgically significant bowel injuries**. *Abdom Imaging* 2015, **40**(5):1026-1033.	4
41	Huber-Wagner S, Lefering R, Qvick LM, Korner M, Kay MV, Pfeifer KJ, Reiser M, Mutschler W, Kanz KG: **Effect of whole-body CT during trauma resuscitation on survival: a retrospective, multicentre study**. *Lancet* 2009, **373**(9673):1455-1461.	3
42	Huber-Wagner S, Mand C, Ruchholtz S, Kuhne CA, Holzapfel K, Kanz KG, van Griensven M, Biberthaler P, Lefering R, TraumaRegister DGU: **Effect of the localisation of the CT scanner during trauma resuscitation on survival -- a retrospective, multicentre study**. *Injury* 2014, **45 Suppl 3**:S76-82.	5
43	Corbacioglu SK, Er E, Aslan S, Seviner M, Aksel G, Dogan NO, Guler S, Bitir A: **The significance of routine thoracic computed tomography in patients with blunt chest trauma**. *Injury* 2015, **46**(5):849-853.	2
44	Strumwasser A, Chong V, Chu E, Victorino GP: **Thoracic computed tomography is an effective screening modality in patients with penetrating injuries to the chest**. *Injury* 2016, **47**(9):2000-2005.	4
45	Kartal ZA, Kozaci N, Cekic B, Beydilli I, Akcimen M, Guven DS, Toslak IE: **CT interpretations in multiply injured patients: comparison of emergency physicians and on-call radiologists**. *Am J Emerg Med* 2016, **34**(12):2331-2335.	4
46	Caputo ND, Stahmer C, Lim G, Shah K: **Whole-body computed tomographic scanning leads to better survival as opposed to selective scanning in trauma patients: a systematic review and meta-analysis**. *J Trauma Acute Care Surg* 2014, **77**(4):534-539.	1
47	Hajibandeh S, Hajibandeh S: **Systematic review: effect of whole-body computed tomography on mortality in trauma patients**. *J Inj Violence Res* 2015, **7**(2):64-74.	1
48	Long B, April MD, Summers S, Koyfman A: **Whole body CT versus selective radiological imaging strategy in trauma: an evidence-based clinical review**. *Am J Emerg Med* 2017, **35**(9):1356-1362.	5
49	Surendran A, Mori A, Varma DK, Gruen RL: **Systematic review of the benefits and harms of whole-body computed tomography in the early management of multitrauma patients: are we getting the whole picture?** *J Trauma Acute Care Surg* 2014, **76**(4):1122-1130.	1
50	Sierink JC, Treskes K, Edwards MJ, Beuker BJ, den Hartog D, Hohmann J, Dijkgraaf MG, Luitse JS, Beenen LF, Hollmann MW *et al*: **Immediate total-body CT scanning versus conventional imaging and selective CT**	2

**R7 Imaging**		
**Reference**	**Citation**	**LoE**
	**scanning in patients with severe trauma (REACT-2): a randomised controlled trial**. *Lancet* 2016, **388**(10045):673-683.	
51	Caleo O, Bocchini G, Paoletta S, Ierardi AM, Scionti A, Tonerini M, Guida F, Sica G, Perillo A, Carrafiello G *et al*: **Spontaneous non-aortic retroperitoneal hemorrhage: etiology, imaging characterization and impact of MDCT on management. A multicentric study**. *Radiol Med* 2015, **120**(1):133-148.	3
52	Hallinan JT, Tan CH, Pua U: **The role of multidetector computed tomography versus digital subtraction angiography in triaging care and management in abdominopelvic trauma**. *Singapore Med J* 2016, **57**(9):497-502.	4
53	Anderson SW, Soto JA, Lucey BC, Burke PA, Hirsch EF, Rhea JT: **Blunt trauma: feasibility and clinical utility of pelvic CT angiography performed with 64-detector row CT**. *Radiology* 2008, **246**(2):410-419.	3
54	Anderson SW, Varghese JC, Lucey BC, Burke PA, Hirsch EF, Soto JA: **Blunt splenic trauma: delayed-phase CT for differentiation of active hemorrhage from contained vascular injury in patients**. *Radiology* 2007, **243**(1):88-95.	3
55	Fang JF, Chen RJ, Wong YC, Lin BC, Hsu YB, Kao JL, Chen MF: **Classification and treatment of pooling of contrast material on computed tomographic scan of blunt hepatic trauma**. *J Trauma* 2000, **49**(6):1083-1088.	4
56	Tan BK, Pothiawala S, Ong ME: **Emergency thoracotomy: a review of its role in severe chest trauma**. *Minerva Chir* 2013, **68**(3):241-250.	5
57	Wu CH, Wang LJ, Wong YC, Fang JF, Lin BC, Chen HW, Huang CC, Hung SC: **Contrast-enhanced multiphasic computed tomography for identifying life-threatening mesenteric hemorrhage and transmural bowel injuries**. *J Trauma* 2011, **71**(3):543-548.	5
58	Juern JS, Milia D, Codner P, Beckman M, Somberg L, Webb T, Weigelt JA: **Clinical significance of computed tomography contrast extravasation in blunt trauma patients with a pelvic fracture**. *J Trauma Acute Care Surg* 2017, **82**(1):138-140.	4
59	Huber-Wagner S, Kanz KG, Hanschen M, van Griensven M, Biberthaler P, Lefering R: **Whole-body computed tomography in severely injured patients**. *Curr Opin Crit Care* 2018, **24**(1):55-61.	3
60	Hinzpeter R, Boehm T, Boll D, Constantin C, Del Grande F, Fretz V, Leschka S, Ohletz T, Bronnimann M, Schmidt S *et al*: **Imaging algorithms and CT protocols in trauma patients: survey of Swiss emergency centers**. *Eur Radiol* 2017, **27**(5):1922-1928.	5
61	Gordic S, Alkadhi H, Hodel S, Simmen HP, Brueesch M, Frauenfelder T, Wanner G, Sprengel K: **Whole-body CT-based imaging algorithm for multiple trauma patients: radiation dose and time to diagnosis**. *Br J Radiol* 2015, **88**(1047):20140616.	3
62	Linder F, Mani K, Juhlin C, Eklof H: **Routine whole body CT of high energy trauma patients leads to excessive radiation exposure**. *Scand J Trauma Resusc Emerg Med* 2016, **24**:7.	3
63	Davies RM, Scrimshire AB, Sweetman L, Anderton MJ, Holt EM: **A decision tool for whole-body CT in major trauma that safely reduces unnecessary scanning and associated radiation risks: An initial exploratory analysis**. *Injury* 2016, **47**(1):43-49.	4

**R8 Haemoglobin**		
**Reference**	**Citation**	**LoE**
1	Gamal M, Abdelhamid B, Zakaria D, Dayem OAE, Rady A, Fawzy M, Hasanin A: **Evaluation of noninvasive hemoglobin monitoring in trauma patients with low hemoglobin levels**. *Shock* 2018, **49**(2):150-153.	2
2	Ryan ML, Maxwell AC, Manning L, Jacobs JD, Bachier-Rodriguez M, Feliz A, Williams RF: **Noninvasive hemoglobin measurement in pediatric trauma patients**. *J Trauma Acute Care Surg* 2016, **81**(6):1162-1166.	2
3	Paradis NA, Balter S, Davison CM, Simon G, Rose M: **Hematocrit as a predictor of significant injury after penetrating trauma**. *Am J Emerg Med* 1997, **15**(3):224-228.	4
4	Snyder HS: **Significance of the initial spun hematocrit in trauma patients**. *Am J Emerg Med* 1998, **16**(2):150-153.	4
5	Zehtabchi S, Sinert R, Goldman M, Kapitanyan R, Ballas J: **Diagnostic performance of serial haematocrit measurements in identifying major injury in adult trauma patients**. *Injury* 2006, **37**(1):46-52.	3
6	Greenfield RH, Bessen HA, Henneman PL: **Effect of crystalloid infusion on hematocrit and intravascular volume in healthy, nonbleeding subjects**. *Ann Emerg Med* 1989, **18**(1):51-55.	3
7	Kass LE, Tien IY, Ushkow BS, Snyder HS: **Prospective crossover study of the effect of phlebotomy and intravenous crystalloid on hematocrit.** *Acad Emerg Med* 1997, **4**(3):198-201.	3
8	Stamler KD: **Effect of crystalloid infusion on hematocrit in nonbleeding patients, with applications to clinical traumatology**. *Ann Emerg Med* 1989, **18**(7):747-749.	2
9	Ryan ML, Thorson CM, Otero CA, Vu T, Schulman CI, Livingstone AS, Proctor KG: **Initial hematocrit in trauma: a paradigm shift?** *J Trauma Acute Care Surg* 2012, **72**(1):54-59; discussion 59-60.	4
10	Knottenbelt JD: **Low initial hemoglobin levels in trauma patients: an important indicator of ongoing hemorrhage**. *J Trauma* 1991, **31**(10):1396-1399.	4
11	Thorson CM, Van Haren RM, Ryan ML, Pereira R, Olloqui J, Guarch GA, Barrera JM, Busko AM, Livingstone AS, Proctor KG: **Admission hematocrit and transfusion requirements after trauma**. *J Am Coll Surg* 2013, **216**(1):65-73.	4
12	Maegele M: **Frequency, risk stratification and therapeutic management of acute post-traumatic coagulopathy**. *Vox Sang* 2009, **97**(1):39-49.	4
13	Vandromme MJ, Griffin RL, Kerby JD, McGwin G, Jr., Rue LW, 3rd, Weinberg JA: **Identifying risk for massive transfusion in the relatively normotensive patient: utility of the prehospital shock index**. *J Trauma* 2011, **70**(2):384-388; discussion 388-390.	3
14	Thorson CM, Ryan ML, Van Haren RM, Pereira R, Olloqui J, Otero CA, Schulman CI, Livingstone AS, Proctor KG: **Change in hematocrit during trauma assessment predicts bleeding even with ongoing fluid resuscitation**. *Am Surg* 2013, **79**(4):398-406.	4
15	Holstein JH, Culemann U, Pohlemann T, Working Group Mortality in Pelvic Fracture P: **What are predictors of mortality in patients with pelvic fractures?** *Clin Orthop Relat Res* 2012, **470**(8):2090-2097.	3
16	Schlimp CJ, Voelckel W, Inaba K, Maegele M, Ponschab M, Schochl H: **Estimation of plasma fibrinogen levels based on hemoglobin, base excess and Injury Severity Score upon emergency room admission**. *Crit Care* 2013, **17**(4):R137.	2

**R9 Serum lactate and base deficit**		
**Reference**	**Citation**	**LoE**
1	Broder G, Weil MH: **Excess lactate: An index of reversibility of shock in human patients**. *Science* 1964, **143**(3613):1457-1459.	2
2	Baron BJ, Scalea TM: **Acute blood loss**. *Emerg Med Clin North Am* 1996, **14**(1):35-55.	5
3	Bilkovski RN, Rivers EP, Horst HM: **Targeted resuscitation strategies after injury**. *Curr Opin Crit Care* 2004, **10**(6):529-538.	4
4	Porter JM, Ivatury RR: **In search of the optimal end points of resuscitation in trauma patients: a review**. *J Trauma* 1998, **44**(5):908-914.	4
5	Wilson M, Davis DP, Coimbra R: **Diagnosis and monitoring of hemorrhagic shock during the initial resuscitation of multiple trauma patients: a review**. *J Emerg Med* 2003, **24**(4):413-422.	3
6	Vincent JL, Dufaye P, Berre J, Leeman M, Degaute JP, Kahn RJ: **Serial lactate determinations during circulatory shock**. *Crit Care Med* 1983, **11**(6):449-451.	2
7	Abramson D, Scalea TM, Hitchcock R, Trooskin SZ, Henry SM, Greenspan J: **Lactate clearance and survival following injury**. *J Trauma* 1993, **35**(4):584-588; discussion 588-589.	4
8	Manikis P, Jankowski S, Zhang H, Kahn RJ, Vincent JL: **Correlation of serial blood lactate levels to organ failure and mortality after trauma**. *Am J Emerg Med* 1995, **13**(6):619-622.	3
9	Caputo N, Fraser R, Paliga A, Kanter M, Hosford K, Madlinger R: **Triage vital signs do not correlate with serum lactate or base deficit, and are less predictive of operative intervention in penetrating trauma patients: a prospective cohort study**. *Emerg Med J* 2013, **30**(7):546-550.	3
10	Vincent JL, Quintairos ESA, Couto L, Jr., Taccone FS: **The value of blood lactate kinetics in critically ill patients: a systematic review**. *Crit Care* 2016, **20**(1):257.	2
11	Herbert HK, Dechert TA, Wolfe L, Aboutanos MB, Malhotra AK, Ivatury RR, Duane TM: **Lactate in trauma: a poor predictor of mortality in the setting of alcohol ingestion**. *Am Surg* 2011, **77**(12):1576-1579.	3
12	Gustafson ML, Hollosi S, Chumbe JT, Samanta D, Modak A, Bethea A: **The effect of ethanol on lactate and base deficit as predictors of morbidity and mortality in trauma**. *Am J Emerg Med* 2015.	4
13	Arnold TD, Miller M, van Wessem KP, Evans JA, Balogh ZJ: **Base deficit from the first peripheral venous sample: a surrogate for arterial base deficit in the trauma bay**. *J Trauma* 2011, **71**(4):793-797; discussion 797.	4
14	Davis JW, Parks SN, Kaups KL, Gladen HE, O'Donnell-Nicol S: **Admission base deficit predicts transfusion requirements and risk of complications**. *J Trauma* 1996, **41**(5):769-774.	2
15	Davis JW, Kaups KL, Parks SN: **Base deficit is superior to pH in evaluating clearance of acidosis after traumatic shock**. *J Trauma* 1998, **44**(1):114-118.	2
16	Mutschler M, Nienaber U, Brockamp T, Wafaisade A, Fabian T, Paffrath T, Bouillon B, Maegele M, TraumaRegister DGU: **Renaissance of base deficit for the initial assessment of trauma patients: a base deficit-based classification for hypovolemic shock developed on data from 16,305 patients derived from the TraumaRegister DGU(R)**. *Crit Care* 2013, **17**(2):R42.	3
17	Davis JW, Kaups KL: **Base deficit in the elderly: a marker of severe injury and death**. *J Trauma* 1998, **45**(5):873-877.	3
18	Randolph LC, Takacs M, Davis KA: **Resuscitation in the pediatric trauma population: admission base deficit remains an important prognostic indicator**. *J Trauma* 2002, **53**(5):838-842.	2
19	Mikulaschek A, Henry SM, Donovan R, Scalea TM: **Serum lactate is not predicted by anion gap or base excess after trauma resuscitation**. *J Trauma* 1996, **40**(2):218-222; discussion 222-214.	2

R10 Coagulation monitoring		
**Reference**	**Citation**	**LoE**
1	Frith D, Goslings JC, Gaarder C, Maegele M, Cohen MJ, Allard S, Johansson PI, Stanworth S, Thiemermann C, Brohi K: **Definition and drivers of acute traumatic coagulopathy: clinical and experimental investigations.** *Journal of thrombosis and haemostasis : JTH* 2010, **8**(9):1919-1925.	4
2	Peltan ID, Vande Vusse LK, Maier RV, Watkins TR: **An international normalized ratio-based definition of acute traumatic coagulopathy is associated with mortality, venous thromboembolism, and multiple organ failure after injury**. *Crit Care Med* 2015, **43**(7):1429-1438.	3
3	Celenza A, Skinner K: **Comparison of emergency department point-of-care international normalised ratio (INR) testing with laboratory-based testing**. *Emerg Med J* 2011, **28**(2):136-140.	3
4	David JS, Levrat A, Inaba K, Macabeo C, Rugeri L, Fontaine O, Cheron A, Piriou V: **Utility of a point-of-care device for rapid determination of prothrombin time in trauma patients: a preliminary study**. *J Trauma Acute Care Surg* 2012, **72**(3):703-707.	4
5	Gauss T, Hamada S, Jurcisin I, Dahmani S, Boudaoud L, Mantz J, Paugam-Burtz C: **Limits of agreement between measures obtained from standard laboratory and the point-of-care device Hemochron Signature Elite(R) during acute haemorrhage**. *Br J Anaesth* 2014, **112**(3):514-520.	4
6	Goodman MD, Makley AT, Hanseman DJ, Pritts TA, Robinson BR: **All the bang without the bucks: Defining essential point-of-care testing for traumatic coagulopathy**. *J Trauma Acute Care Surg* 2015, **79**(1):117-124; discussion 124.	4
7	Mann KG, Butenas S, Brummel K: **The dynamics of thrombin formation**. *Arterioscler Thromb Vasc Biol* 2003, **23**(1):17-25.	4
8	Davenport R, Manson J, De'Ath H, Platton S, Coates A, Allard S, Hart D, Pearse R, Pasi KJ, MacCallum P *et al*: **Functional definition and characterization of acute traumatic coagulopathy**. *Crit Care Med* 2011, **39**(12):2652-2658.	4
9	Levrat A, Gros A, Rugeri L, Inaba K, Floccard B, Negrier C, David JS: **Evaluation of rotation thrombelastography for the diagnosis of hyperfibrinolysis in trauma patients**. *Br J Anaesth* 2008, **100**(6):792-797.	4
10	Tauber H, Innerhofer P, Breitkopf R, Westermann I, Beer R, El Attal R, Strasak A, Mittermayr M: **Prevalence and impact of abnormal ROTEM(R) assays in severe blunt trauma: results of the 'Diagnosis and Treatment of Trauma-Induced Coagulopathy (DIA-TRE-TIC) study'**. *Br J Anaesth* 2011, **107**(3):378-387.	4
11	Theusinger OM, Wanner GA, Emmert MY, Billeter A, Eismon J, Seifert B, Simmen HP, Spahn DR, Baulig W: **Hyperfibrinolysis diagnosed by rotational thromboelastometry (ROTEM) is associated with higher mortality in patients with severe trauma**. *Anesth Analg* 2011, **113**(5):1003-1012.	4
12	Haas T, Spielmann N, Mauch J, Madjdpour C, Speer O, Schmugge M, Weiss M: **Comparison of thromboelastometry (ROTEM(R)) with standard plasmatic coagulation testing in paediatric surgery**. *Br J Anaesth* 2012, **108**(1):36-41.	4
13	Haas T, Spielmann N, Mauch J, Speer O, Schmugge M, Weiss M: **Reproducibility of thrombelastometry (ROTEM(R)): point-of-care versus hospital laboratory performance**. *Scand J Clin Lab Invest* 2012, **72**(4):313-317.	4
14	Bliden KP, Chaudhary R, Mohammed N, Muresan AA, Lopez-Espina CG, Cohen E, Raviv G, Doubleday M, Zaman F, Mathew B *et al*: **Determination of non-Vitamin K oral anticoagulant (NOAC) effects using a new-generation thrombelastography TEG 6s system**. *J Thromb Thrombolysis* 2017, **43**(4):437-445.	4
15	Chitlur M, Sorensen B, Rivard GE, Young G, Ingerslev J, Othman M, Nugent D, Kenet G, Escobar M, Lusher J: **Standardization of**	3

R10 Coagulation monitoring		
**Reference**	**Citation**	**LoE**
	**thromboelastography: a report from the TEG-ROTEM working group**. *Haemophilia* 2011, **17**(3):532-537.	
16	Rugeri L, Levrat A, David JS, Delecroix E, Floccard B, Gros A, Allaouchiche B, Negrier C: **Diagnosis of early coagulation abnormalities in trauma patients by rotation thrombelastography**. *J Thromb Haemost* 2007, **5**(2):289-295.	4
17	Gonzalez E, Moore EE, Moore HB, Chapman MP, Chin TL, Ghasabyan A, Wohlauer MV, Barnett CC, Bensard DD, Biffl WL *et al*: **Goal-directed hemostatic resuscitation of trauma-induced coagulopathy: A pragmatic randomized clinical trial comparing a viscoelastic assay to conventional coagulation assays**. *Ann Surg* 2016, **263**(6):1051-1059.	2
18	Cotton BA, Faz G, Hatch QM, Radwan ZA, Podbielski J, Wade C, Kozar RA, Holcomb JB: **Rapid thrombelastography delivers real-time results that predict transfusion within 1 hour of admission**. *J Trauma* 2011, **71**(2):407-414; discussion 414-407.	4
19	Holcomb JB, Minei KM, Scerbo ML, Radwan ZA, Wade CE, Kozar RA, Gill BS, Albarado R, McNutt MK, Khan S *et al*: **Admission rapid thrombelastography can replace conventional coagulation tests in the emergency department: experience with 1974 consecutive trauma patients**. *Ann Surg* 2012, **256**(3):476-486.	4
20	Liras IN, Caplan HW, Stensballe J, Wade CE, Cox CS, Cotton BA: **Prevalence and impact of admission acute traumatic coagulopathy on treatment intensity, resource use, and mortality: An evaluation of 956 severely injured children and adolescents**. *J Am Coll Surg* 2017, **224**(4):625-632.	4
21	Vogel AM, Radwan ZA, Cox CS, Jr., Cotton BA: **Admission rapid thrombelastography delivers real-time "actionable" data in pediatric trauma**. *J Pediatr Surg* 2013, **48**(6):1371-1376.	4
22	Hagemo JS, Christiaans SC, Stanworth SJ, Brohi K, Johansson PI, Goslings JC, Naess PA, Gaarder C: **Detection of acute traumatic coagulopathy and massive transfusion requirements by means of rotational thromboelastometry: an international prospective validation study**. *Crit Care* 2015, **19**:97.	4
23	Meyer MA, Ostrowski SR, Sorensen AM, Meyer AS, Holcomb JB, Wade CE, Johansson PI, Stensballe J: **Fibrinogen in trauma, an evaluation of thrombelastography and rotational thromboelastometry fibrinogen assays**. *J Surg Res* 2015, **194**(2):581-590.	4
24	Moore HB, Moore EE, Chapman MP, Gonzalez E, Slaughter AL, Morton AP, D'Alessandro A, Hansen KC, Sauaia A, Banerjee A *et al*: **Viscoelastic measurements of platelet function, not fibrinogen function, predicts sensitivity to tissue-type plasminogen activator in trauma patients**. *J Thromb Haemost* 2015, **13**(10):1878-1887.	4
25	Gall LS, Vulliamy P, Gillespie S, Jones TF, Pierre RSJ, Breukers SE, Gaarder C, Juffermans NP, Maegele M, Stensballe J *et al*: **The S100A10 pathway mediates an occult hyperfibrinolytic subtype in trauma patients**. *Annals of Surgery* 2018:1.	3
26	Baksaas-Aasen K, Van Dieren S, Balvers K, Juffermans NP, Naess PA, Rourke C, Eaglestone S, Ostrowski SR, Stensballe J, Stanworth S et al: **Data-driven Development of ROTEM and TEG Algorithms for the Management of Trauma Hemorrhage: A Prospective Observational Multicenter Study.** *Ann Surg* 2018.	2
27	Hunt H, Stanworth S, Curry N, Woolley T, Cooper C, Ukoumunne O, Zhelev Z, Hyde C: **Thromboelastography (TEG) and rotational thromboelastometry (ROTEM) for trauma induced coagulopathy in adult trauma patients with bleeding**. *Cochrane Database Syst Rev* 2015, **2**:CD010438.	1
28	Da Luz LT, Nascimento B, Shankarakutty AK, Rizoli S, Adhikari NK: **Effect of thromboelastography (TEG(R)) and rotational thromboelastometry**	2

R10 Coagulation monitoring		
**Reference**	**Citation**	**LoE**
	**(ROTEM(R)) on diagnosis of coagulopathy, transfusion guidance and mortality in trauma: descriptive systematic review**. *Crit Care* 2014, **18**(5):518.	
29	Harr JN, Moore EE, Ghasabyan A, Chin TL, Sauaia A, Banerjee A, Silliman CC: **Functional fibrinogen assay indicates that fibrinogen is critical in correcting abnormal clot strength following trauma**. *Shock* 2013, **39**(1):45-49.	4
30	Agren A, Wikman AT, Holmstrom M, Ostlund A, Edgren G: **Thromboelastography (TEG(R)) compared to conventional coagulation tests in surgical patients--a laboratory evaluation**. *Scand J Clin Lab Invest* 2013, **73**(3):214-220.	4
31	Agren A, Wikman AT, Ostlund A, Edgren G: **TEG(R) functional fibrinogen analysis may overestimate fibrinogen levels**. *Anesth Analg* 2014, **118**(5):933-935.	4
32	Hagemo JS, Naess PA, Johansson P, Windelov NA, Cohen MJ, Roislien J, Brohi K, Heier HE, Hestnes M, Gaarder C: **Evaluation of TEG((R)) and RoTEM((R)) inter-changeability in trauma patients**. *Injury* 2013, **44**(5):600-605.	4
33	Schlimp CJ, Khadem A, Klotz A, Solomon C, Hochleitner G, Ponschab M, Redl H, Schochl H: **Rapid measurement of fibrinogen concentration in whole blood using a steel ball coagulometer**. *J Trauma Acute Care Surg* 2015, **78**(4):830-836.	4
34	Maegele M, Grottke O, Schochl H, Sakowitz OA, Spannagl M, Koscielny J: **Direct Oral Anticoagulants in Emergency Trauma Admissions**. *Dtsch Arztebl Int* 2016, **113**(35-36):575-582.	5
35	Batchelor JS, Grayson A: **A meta-analysis to determine the effect on survival of platelet transfusions in patients with either spontaneous or traumatic antiplatelet medication-associated intracranial haemorrhage**. *BMJ Open* 2012, **2**(2):e000588.	2
36	Inui TS, Parina R, Chang DC, Inui TS, Coimbra R: **Mortality after ground-level fall in the elderly patient taking oral anticoagulation for atrial fibrillation/flutter: a long-term analysis of risk versus benefit**. *J Trauma Acute Care Surg* 2014, **76**(3):642-649; discussion 649-650.	3
37	Peck KA, Calvo RY, Sise CB, Johnson J, Yen JW, Sise MJ, Dunne CE, Badiee J, Shackford SR, Lobatz MA: **Death after discharge: predictors of mortality in older brain-injured patients**. *J Trauma Acute Care Surg* 2014, **77**(6):978-983.	3
38	Wutzler S, Maegele M, Marzi I, Spanholtz T, Wafaisade A, Lefering R, Trauma Registry of the German Society for Trauma S: **Association of preexisting medical conditions with in-hospital mortality in multiple-trauma patients**. *J Am Coll Surg* 2009, **209**(1):75-81.	3
39	Douxfils J, Ageno W, Samama CM, Lessire S, Ten Cate H, Verhamme P, Dogne JM, Mullier F: **Laboratory testing in patients treated with direct oral anticoagulants: a practical guide for clinicians**. *J Thromb Haemost* 2018, **16**(2):209-219.	3
40	Baglin T: **The role of the laboratory in treatment with new oral anticoagulants**. *J Thromb Haemost* 2013, **11 Suppl 1**:122-128.	5
41	Cuker A, Siegal DM, Crowther MA, Garcia DA: **Laboratory measurement of the anticoagulant activity of the non-vitamin K oral anticoagulants**. *J Am Coll Cardiol* 2014, **64**(11):1128-1139.	5

R11 Platelet function monitoring		
**Reference**	**Citation**	**LoE**
1	Kutcher ME, Redick BJ, McCreery RC, Crane IM, Greenberg MD, Cachola LM, Nelson MF, Cohen MJ: **Characterization of platelet dysfunction after trauma**. *J Trauma Acute Care Surg* 2012, **73**(1):13-19.	3
2	Solomon C, Traintinger S, Ziegler B, Hanke A, Rahe-Meyer N, Voelckel W, Schöchl H: **Platelet function following trauma. A multiple electrode aggregometry study**. *Thromb Haemost* 2011, **106**(2):322-330.	4
3	Wohlauer MV, Moore EE, Thomas S, Sauaia A, Evans E, Harr J, Silliman CC, Ploplis V, Castellino FJ, Walsh M: **Early platelet dysfunction: an unrecognized role in the acute coagulopathy of trauma**. *J Am Coll Surg* 2012, **214**(5):739-746.	3
4	Connelly CR, Yonge JD, McCully SP, Hart KD, Hilliard TC, Lape DE, Watson JJ, Rick B, Houser B, Deloughery TG *et al*: **Assessment of three point-of-care platelet function assays in adult trauma patients**. *J Surg Res* 2017, **212**:260-269.	3
5	Stettler GR, Moore EE, Moore HB, Nunns GR, Huebner BR, Einersen P, Ghasabyan A, Silliman CC, Banerjee A, Sauaia A: **Platelet adenosine diphosphate receptor inhibition provides no advantage in predicting need for platelet transfusion or massive transfusion**. *Surgery* 2017, **162**(6):1286-1294.	3
6	Podda G, Scavone M, Femia EA, Cattaneo M: **Aggregometry in the settings of thrombocytopenia, thrombocytosis and antiplatelet therapy**. *Platelets* 2018:1-6.	5
7	Bachelani AM, Bautz JT, Sperry JL, Corcos A, Zenati M, Billiar TR, Peitzman AB, Marshall GT: **Assessment of platelet transfusion for reversal of aspirin after traumatic brain injury**. *Surgery* 2011, **150**(4):836-843.	4
8	Gozal YM, Carroll CP, Krueger BM, Khoury J, Andaluz NO: **Point-of-care testing in the acute management of traumatic brain injury: Identifying the coagulopathic patient**. *Surg Neurol Int* 2017, **8**:48.	4
9	Beynon C, Scherer M, Jakobs M, Jung C, Sakowitz OW, Unterberg AW: **Initial experiences with Multiplate(R) for rapid assessment of antiplatelet agent activity in neurosurgical emergencies**. *Clin Neurol Neurosurg* 2013, **115**(10):2003-2008.	4
10	Lindblad C, Thelin EP, Nekludov M, Frostell A, Nelson DW, Svensson M, Bellander BM: **Assessment of platelet function in traumatic brain injury-a retrospective observational study in the neuro-critical care setting**. *Front Neurol* 2018, **9**:15.	3
11	Parry PV, Choi PA, Bauer JS, Panczykowski DM, Puccio AM, Okonkwo DO: **Utility of the aspirin and p2y12 response assays to determine the effect of antiplatelet agents on platelet reactivity in traumatic brain injury**. *Neurosurgery* 2017, **80**(1):92-96.	3
12	Prinz V, Finger T, Bayerl S, Rosenthal C, Wolf S, Liman T, Vajkoczy P: **High prevalence of pharmacologically induced platelet dysfunction in the acute setting of brain injury**. *Acta Neurochir (Wien)* 2016, **158**(1):117-123.	4
13	Daley MJ, Enright Z, Nguyen J, Ali S, Clark A, Aydelotte JD, Teixeira PG, Coopwood TB, Brown CV: **Adenosine diphosphate platelet dysfunction on thromboelastogram is independently associated with increased morality in traumatic brain injury**. *Eur J Trauma Emerg Surg* 2017, **43**(1):105-111.	3
14	Bartels A, Sarpong Y, Coberly J, Hughes N, Litt J, Quick J, Kessel J, Nelson C, Coughenour J, Barnes SL *et al*: **Failure of the Platelet Function Assay (PFA)-100 to detect antiplatelet agents**. *Surgery* 2015, **158**(4):1012-1018; discussion 1018-1019.	3
15	Choi PA, Parry PV, Bauer JS, Zusman BE, Panczykowski DM, Puccio AM, Okonkwo DO: **Use of Aspirin and P2Y12 Response Assays in Detecting Reversal of Platelet Inhibition With Platelet Transfusion in Patients With Traumatic Brain Injury on Antiplatelet Therapy**. *Neurosurgery* 2017, **80**(1):98-104.	3

**R11 Platelet function monitoring**		
**Reference**	**Citation**	**LoE**
16	Short S, Kram B, Taylor S, Cheng J, Ali K, Vasquez D: **Effect of platelet inhibition on bleeding complications in trauma patients on preinjury clopidogrel**. *J Trauma Acute Care Surg* 2013, **74**(6):1419-1424.	3
17	George MJ, Burchfield J, MacFarlane B, Wang YW, Cardenas JC, White NJ, Gill BS, Wade CE: **Multiplate and TEG platelet mapping in a population of severely injured trauma patients**. *Transfus Med* 2017.	3
18	Henriksen HH, Grand AG, Viggers S, Baer LA, Solbeck S, Cotton BA, Matijevic N, Ostrowski SR, Stensballe J, Fox EE *et al*: **Impact of blood products on platelet function in patients with traumatic injuries: a translational study**. *J Surg Res* 2017, **214**:154-161.	3
19	Ramsey MT, Fabian TC, Shahan CP, Sharpe JP, Mabry SE, Weinberg JA, Croce MA, Jennings LK: **A prospective study of platelet function in trauma patients**. *J Trauma Acute Care Surg* 2016, **80**(5):726-732; discussion 732-723.	3
20	Castellino FJ, Chapman MP, Donahue DL, Thomas S, Moore EE, Wohlauer MV, Fritz B, Yount R, Ploplis V, Davis P *et al*: **Traumatic brain injury causes platelet adenosine diphosphate and arachidonic acid receptor inhibition independent of hemorrhagic shock in humans and rats**. *J Trauma Acute Care Surg* 2014, **76**(5):1169-1176.	3
21	Davis PK, Musunuru H, Walsh M, Cassady R, Yount R, Losiniecki A, Moore EE, Wohlauer MV, Howard J, Ploplis VA *et al*: **Platelet dysfunction is an early marker for traumatic brain injury-induced coagulopathy**. *Neurocrit Care* 2013, **18**(2):201-208.	3
22	Nekludov M, Bellander BM, Blomback M, Wallen HN: **Platelet dysfunction in patients with severe traumatic brain injury**. *J Neurotrauma* 2007, **24**(11):1699-1706.	3
23	Sirajuddin S, Valdez C, DePalma L, Maluso P, Singhal R, Schroeder M, Sarani B: **Inhibition of platelet function is common following even minor injury**. *J Trauma Acute Care Surg* 2016, **81**(2):328-332.	3
24	Hetherington JJ, Ford I, Ashcroft GP, Jansen JO: **Intraoperative changes in platelet function in relation to moderate haemorrhage**. *Thromb Res* 2015, **135**(6):1198-1202.	3
25	Moore HB, Moore EE, Chapman MP, Gonzalez E, Slaughter AL, Morton AP, D'Alessandro A, Hansen KC, Sauaia A, Banerjee A *et al*: **Viscoelastic measurements of platelet function, not fibrinogen function, predicts sensitivity to tissue-type plasminogen activator in trauma patients**. *J Thromb Haemost* 2015, **13**(10):1878-1887.	4
26	Joseph B, Pandit V, Sadoun M, Larkins CG, Kulvatunyou N, Tang A, Mino M, Friese RS, Rhee P: **A prospective evaluation of platelet function in patients on antiplatelet therapy with traumatic intracranial hemorrhage**. *J Trauma Acute Care Surg* 2013, **75**(6):990-994.	3
27	Briggs A, Gates JD, Kaufman RM, Calahan C, Gormley WB, Havens JM: **Platelet dysfunction and platelet transfusion in traumatic brain injury**. *J Surg Res* 2015, **193**(2):802-806.	3
28	Holzmacher JL, Reynolds C, Patel M, Maluso P, Holland S, Gamsky N, Moore H, Acquista E, Carrick M, Amdur R *et al*: **Platelet transfusion does not improve outcomes in patients with brain injury on antiplatelet therapy**. *Brain Inj* 2018, **32**(3):325-330.	4

**R12-14 Tissue oxygenation, restricted volume replacement, vasopressors and inotropic agents**		
**Reference**	**Citation**	**LoE**
1	Bickell WH, Wall MJ, Jr., Pepe PE, Martin RR, Ginger VF, Allen MK, Mattox KL: **Immediate versus delayed fluid resuscitation for hypotensive patients with penetrating torso injuries.** *N Engl J Med* 1994, **331**(17):1105-1109.	2
2	Sampalis JS, Tamim H, Denis R, Boukas S, Ruest SA, Nikolis A, Lavoie A, Fleiszer D, Brown R, Mulder D *et al*: **Ineffectiveness of on-site intravenous lines: is prehospital time the culprit?** *J Trauma* 1997, **43**(4):608-615; discussion 615-607.	3
3	Schreiber MA, Meier EN, Tisherman SA, Kerby JD, Newgard CD, Brasel K, Egan D, Witham W, Williams C, Daya M *et al*: **A controlled resuscitation strategy is feasible and safe in hypotensive trauma patients: results of a prospective randomized pilot trial.** *J Trauma Acute Care Surg* 2015, **78**(4):687-695; discussion 695-687.	2
4	Dutton RP, Mackenzie CF, Scalea TM: **Hypotensive resuscitation during active hemorrhage: impact on in-hospital mortality.** *J Trauma* 2002, **52**(6):1141-1146.	2
5	Turner J, Nicholl J, Webber L, Cox H, Dixon S, Yates D: **A randomised controlled trial of prehospital intravenous fluid replacement therapy in serious trauma.** *Health Technol Assess* 2000, **4**(31):1-57.	2
6	Lou X, Lu G, Zhao M, Jin P: **Preoperative fluid management in traumatic shock: A retrospective study for identifying optimal therapy of fluid resuscitation for aged patients.** *Medicine (Baltimore)* 2018, **97**(8):e9966.	3
7	Brenner M, Stein DM, Hu PF, Aarabi B, Sheth K, Scalea TM: **Traditional systolic blood pressure targets underestimate hypotension-induced secondary brain injury.** *J Trauma Acute Care Surg* 2012, **72**(5):1135-1139.	3
8	Brown JB, Cohen MJ, Minei JP, Maier RV, West MA, Billiar TR, Peitzman AB, Moore EE, Cuschieri J, Sperry JL *et al*: **Goal-directed resuscitation in the prehospital setting: a propensity-adjusted analysis.** *J Trauma Acute Care Surg* 2013, **74**(5):1207-1212; discussion 1212-1204.	2
9	Champion HR: **Prehospital intravenous fluid administration is associated with higher mortality in trauma patients.** *Annals of Surgery* 2014, **259**(2):e19.	N/A
10	Driessen A, Frohlich M, Schafer N, Mutschler M, Defosse JM, Brockamp T, Bouillon B, Sturmer EK, Lefering R, Maegele M *et al*: **Prehospital volume resuscitation--Did evidence defeat the crystalloid dogma? An analysis of the TraumaRegister DGU(R) 2002-2012.** *Scand J Trauma Resusc Emerg Med* 2016, **24**:42.	3
11	Harada MY, Ko A, Barmparas G, Smith EJ, Patel BK, Dhillon NK, Thomsen GM, Ley EJ: **10-Year trend in crystalloid resuscitation: Reduced volume and lower mortality.** *Int J Surg* 2017, **38**:78-82.	3
12	Haut ER, Kalish BT, Cotton BA, Efron DT, Haider AH, Stevens KA, Kieninger AN, Cornwell EE, 3rd, Chang DC: **Prehospital intravenous fluid administration is associated with higher mortality in trauma patients: a National Trauma Data Bank analysis.** *Ann Surg* 2011, **253**(2):371-377.	3
13	Hussmann B, Lefering R, Waydhas C, Touma A, Kauther MD, Ruchholtz S, Lendemans S: **The Trauma Registry of the German Society for Trauma S: Does increased prehospital replacement volume lead to a poor clinical course and an increased mortality? A matched-pair analysis of 1896 patients of the Trauma Registry of the German Society for Trauma Surgery who were managed by an emergency doctor at the accident site.** *Injury* 2013, **44**(5):611-617.	3
14	Joseph B, Azim A, Zangbar B, Bauman Z, O'Keeffe T, Ibraheem K, Kulvatunyou N, Tang A, Latifi R, Rhee P: **Improving mortality in trauma laparotomy through the evolution of damage control resuscitation: Analysis of 1,030 consecutive trauma laparotomies.** *J Trauma Acute Care Surg* 2017, **82**(2):328-333.	3

**R12-14 Tissue oxygenation, restricted volume replacement, vasopressors and inotropic agents**		
**Reference**	**Citation**	**LoE**
15	Kasotakis G, Sideris A, Yang Y, de Moya M, Alam H, King DR, Tompkins R, Velmahos G: **Inflammation, Host Response to Injury I: Aggressive early crystalloid resuscitation adversely affects outcomes in adult blunt trauma patients: an analysis of the Glue Grant database.** *J Trauma Acute Care Surg* 2013, **74**(5):1215-1221; discussion 1221-1212.	3
16	Madigan MC, Kemp CD, Johnson JC, Cotton BA: **Secondary abdominal compartment syndrome after severe extremity injury: are early, aggressive fluid resuscitation strategies to blame?** *J Trauma* 2008, **64**(2):280-285.	3
17	Maegele M, Lefering R, Yucel N, Tjardes T, Rixen D, Paffrath T, Simanski C, Neugebauer E, Bouillon B: **Early coagulopathy in multiple injury: an analysis from the German Trauma Registry on 8724 patients.** *Injury* 2007, **38**(3):298-304.	3
18	Morrison CA, Carrick MM, Norman MA, Scott BG, Welsh FJ, Tsai P, Liscum KR, Wall MJ, Jr., Mattox KL: **Hypotensive resuscitation strategy reduces transfusion requirements and severe postoperative coagulopathy in trauma patients with hemorrhagic shock: preliminary results of a randomized controlled trial.** *J Trauma* 2011, **70**(3):652-663.	2
19	Kwan I, Bunn F, Roberts I: **Timing and volume of fluid administration for patients with bleeding.** *Cochrane Database Syst Rev* 2003(3):CD002245.	1
20	Wang CH, Hsieh WH, Chou HC, Huang YS, Shen JH, Yeo YH, Chang HE, Chen SC, Lee CC: **Liberal versus restricted fluid resuscitation strategies in trauma patients: a systematic review and meta-analysis of randomized controlled trials and observational studies*.** *Crit Care Med* 2014, **42**(4):954-961.	1
21	Berry C, Ley EJ, Bukur M, Malinoski D, Margulies DR, Mirocha J, Salim A: **Redefining hypotension in traumatic brain injury.** *Injury* 2012, **43**(11):1833-1837.	3
22	Rhodes A, Evans LE, Alhazzani W, Levy MM, Antonelli M, Ferrer R, Kumar A, Sevransky JE, Sprung CL, Nunnally ME et al: **Surviving sepsis campaign: International guidelines for management of sepsis and septic shock 2016.** *Critical Care Medicine* 2017, **45**(3):486-552.	2
23	Imai Y, Satoh K, Taira N: **Role of the peripheral vasculature in changes in venous return caused by isoproterenol, norepinephrine, and methoxamine in anesthetized dogs.** *Circ Res* 1978, **43**(4):553-561.	N/A
24	Gelman S, Mushlin PS: **Catecholamine-induced changes in the splanchnic circulation affecting systemic hemodynamics.** *Anesthesiology* 2004, **100**(2):434-439.	2
25	Harrois A, Baudry N, Huet O, Kato H, Dupic L, Lohez M, Ziol M, Vicaut E, Duranteau J: **Norepinephrine decreases fluid requirements and blood loss while preserving intestinal villi microcirculation during fluid resuscitation of uncontrolled hemorrhagic shock in mice.** *Anesthesiology* 2015, **122**(5):1093-1102.	N/A
26	Poloujadoff MP, Borron SW, Amathieu R, Favret F, Camara MS, Lapostolle F, Vicaut E, Adnet F: **Improved survival after resuscitation with norepinephrine in a murine model of uncontrolled hemorrhagic shock.** *Anesthesiology* 2007, **107**(4):591-596.	N/A
27	Cohn SM, McCarthy J, Stewart RM, Jonas RB, Dent DL, Michalek JE: **Impact of low-dose vasopressin on trauma outcome: prospective randomized study.** *World J Surg* 2011, **35**(2):430-439.	2
28	Sperry JL, Minei JP, Frankel HL, West MA, Harbrecht BG, Moore EE, Maier RV, Nirula R: **Early use of vasopressors after injury: caution before constriction.** *J Trauma* 2008, 64(1):9-14.	3
29	Van Haren RM, Thorson CM, Valle EJ, Guarch GA, Jouria JM, Busko AM, Namias N, Livingstone AS, Proctor KG: **Vasopressor use during emergency trauma surgery.** *The American Surgeon* 2014, **80**(5):472-478.	3

**R12-14 Tissue oxygenation, restricted volume replacement, vasopressors and inotropic agents**		
**Reference**	**Citation**	**LoE**
30	Barmparas G, Dhillon NK, Smith EJ, Mason R, Melo N, Thomsen GM, Margulies DR, Ley EJ: **Patterns of vasopressor utilization during the resuscitation of massively transfused trauma patients.** *Injury* 2018, **49**(1):8-14.	3
31	Krishnamoorthy V, Rowhani-Rahbar A, Gibbons EF, Rivara FP, Temkin NR, Pontius C, Luk K, Graves M, Lozier D, Chaikittisilpa N *et al*: **Early systolic dysfunction following traumatic brain injury: A cohort study.** *Crit Care Med* 2017, **45**(6):1028-1036.	3

**R15 Type of fluid**		
**Reference**	**Citation**	**LoE**
1	Chowdhury AH, Cox EF, Francis ST, Lobo DN: **A randomized, controlled, double-blind crossover study on the effects of 2-L infusions of 0.9% saline and Plasma-Lyte(R) 148 on renal blood flow velocity and renal cortical tissue perfusion in healthy volunteers**. *Ann Surg* 2012, **256**(1):18-24.	2
2	Emrath ET, Fortenberry JD, Travers C, McCracken CE, Hebbar KB: **Resuscitation with balanced fluids is associated with improved survival in pediatric severe sepsis**. *Crit Care Med* 2017, **45**(7):1177-1183.	3
3	Raghunathan K, Shaw A, Nathanson B, Sturmer T, Brookhart A, Stefan MS, Setoguchi S, Beadles C, Lindenauer PK: **Association between the choice of IV crystalloid and in-hospital mortality among critically ill adults with sepsis***. *Crit Care Med* 2014, **42**(7):1585-1591.	3
4	Self WH, Semler MW, Wanderer JP, Wang L, Byrne DW, Collins SP, Slovis CM, Lindsell CJ, Ehrenfeld JM, Siew ED *et al*: **Balanced crystalloids versus saline in noncritically ill adults**. *N Engl J Med* 2018, **378**(9):819-828.	2
5	Semler MW, Self WH, Wanderer JP, Ehrenfeld JM, Wang L, Byrne DW, Stollings JL, Kumar AB, Hughes CG, Hernandez A *et al*: **Balanced crystalloids versus saline in critically ill adults**. *N Engl J Med* 2018, **378**(9):829-839.	2
6	Sen A, Keener CM, Sileanu FE, Foldes E, Clermont G, Murugan R, Kellum JA: **Chloride content of fluids used for large-volume resuscitation is associated with reduced survival**. *Crit Care Med* 2017, **45**(2):e146-e153.	3
7	Smith CA, Duby JJ, Utter GH, Galante JM, Scherer LA, Schermer CR: **Cost-minimization analysis of two fluid products for resuscitation of critically injured trauma patients**. *Am J Health Syst Pharm* 2014, **71**(6):470-475.	3
8	Young JB, Utter GH, Schermer CR, Galante JM, Phan HH, Yang Y, Anderson BA, Scherer LA: **Saline versus Plasma-Lyte A in initial resuscitation of trauma patients: a randomized trial**. *Ann Surg* 2014, **259**(2):255-262.	2
9	Yunos NM, Bellomo R, Hegarty C, Story D, Ho L, Bailey M: **Association between a chloride-liberal vs chloride-restrictive intravenous fluid administration strategy and kidney injury in critically ill adults**. *JAMA* 2012, **308**(15):1566-1572.	2
10	Reddy SK, Bailey MJ, Beasley RW, Bellomo R, Mackle DM, Psirides AJ, Young PJ: **Effect of 0.9% saline or Plasma-Lyte 148 as crystalloid fluid therapy in the intensive care unit on blood product use and postoperative bleeding after cardiac surgery**. *J Cardiothorac Vasc Anesth* 2017, **31**(5):1630-1638.	2
11	Rowell SE, Fair KA, Barbosa RR, Watters JM, Bulger EM, Holcomb JB, Cohen MJ, Rahbar MH, Fox EE, Schreiber MA: **The impact of pre-hospital administration of lactated ringer's solution versus normal saline in patients with traumatic brain injury**. *J Neurotrauma* 2016, **33**(11):1054-1059.	3
12	Aoki K, Yoshino A, Yoh K, Sekine K, Yamazaki M, Aikawa N: **A comparison of Ringer's lactate and acetate solutions and resuscitative effects on splanchnic dysoxia in patients with extensive burns**. *Burns* 2010, **36**(7):1080-1085.	2
13	Orbegozo Cortés D, Gamarano Barros T, Njimi H, Vincent J-L: **Crystalloids versus colloids: Exploring differences in fluid requirements by systematic review and meta-regression**. *Anesthesia & Analgesia* 2015, **120**(2):389-402.	1
14	Annane D, Siami S, Jaber S, Martin C, Elatrous S, Declere AD, Preiser JC, Outin H, Troche G, Charpentier C *et al*: **Effects of fluid resuscitation with colloids vs crystalloids on mortality in critically ill patients presenting with hypovolemic shock: the CRISTAL randomized trial**. *JAMA* 2013, **310**(17):1809-1817.	2

**R15 Type of fluid**		
**Reference**	**Citation**	**LoE**
15	Perel P, Roberts I: **Colloids versus crystalloids for fluid resuscitation in critically ill patients**. *Cochrane Database Syst Rev* 2011(3):CD000567.	1
16	Raiman M, Mitchell CG, Biccard BM, Rodseth RN: **Comparison of hydroxyethyl starch colloids with crystalloids for surgical patients: A systematic review and meta-analysis**. *Eur J Anaesthesiol* 2016, **33**(1):42-48.	1
17	Perel P, Roberts I, Ker K: **Colloids versus crystalloids for fluid resuscitation in critically ill patients**. *Cochrane Database Syst Rev* 2013, **2**:CD000567.	2
18	Rochwerg B, Alhazzani W, Sindi A, Heels-Ansdell D, Thabane L, Fox-Robichaud A, Mbuagbaw L, Szczeklik W, Alshamsi F, Altayyar S *et al*: **Fluid resuscitation in sepsis: a systematic review and network meta-analysis**. *Ann Intern Med* 2014, **161**(5):347-355.	1
19	Serpa Neto A, Veelo DP, Peireira VG, de Assuncao MS, Manetta JA, Esposito DC, Schultz MJ: **Fluid resuscitation with hydroxyethyl starches in patients with sepsis is associated with an increased incidence of acute kidney injury and use of renal replacement therapy: a systematic review and meta-analysis of the literature**. *J Crit Care* 2014, **29**(1):185 e181-187.	1
20	Gillies MA, Habicher M, Jhanji S, Sander M, Mythen M, Hamilton M, Pearse RM: **Incidence of postoperative death and acute kidney injury associated with i.v. 6% hydroxyethyl starch use: systematic review and meta-analysis**. *Br J Anaesth* 2014, **112**(1):25-34.	1
21	Kind SL, Spahn-Nett GH, Emmert MY, Eismon J, Seifert B, Spahn DR, Theusinger OM: **Is dilutional coagulopathy induced by different colloids reversible by replacement of fibrinogen and factor XIII concentrates?** *Anesth Analg* 2013, **117**(5):1063-1071.	3
22	James MF, Michell WL, Joubert IA, Nicol AJ, Navsaria PH, Gillespie RS: **Resuscitation with hydroxyethyl starch improves renal function and lactate clearance in penetrating trauma in a randomized controlled study: the FIRST trial (Fluids in Resuscitation of Severe Trauma)**. *Br J Anaesth* 2011, **107**(5):693-702.	2
23	Moeller C, Fleischmann C, Thomas-Rueddel D, Vlasakov V, Rochwerg B, Theurer P, Gattinoni L, Reinhart K, Hartog CS: **How safe is gelatin? A systematic review and meta-analysis of gelatin-containing plasma expanders vs crystalloids and albumin**. *J Crit Care* 2016, **35**:75-83.	1
24	Bulger EM, Jurkovich GJ, Nathens AB, Copass MK, Hanson S, Cooper C, Liu PY, Neff M, Awan AB, Warner K *et al*: **Hypertonic resuscitation of hypovolemic shock after blunt trauma: a randomized controlled trial**. *Arch Surg* 2008, **143**(2):139-148; discussion 149.	2
25	Battison C, Andrews PJ, Graham C, Petty T: **Randomized, controlled trial on the effect of a 20% mannitol solution and a 7.5% saline/6% dextran solution on increased intracranial pressure after brain injury**. *Crit Care Med* 2005, **33**(1):196-202; discussion 257-198.	2
26	Cooper DJ, Myles PS, McDermott FT, Murray LJ, Laidlaw J, Cooper G, Tremayne AB, Bernard SS, Ponsford J: **Prehospital hypertonic saline resuscitation of patients with hypotension and severe traumatic brain injury: a randomized controlled trial**. *JAMA* 2004, **291**(11):1350-1357.	2
27	Bulger EM, May S, Brasel KJ, Schreiber M, Kerby JD, Tisherman SA, Newgard C, Slutsky A, Coimbra R, Emerson S *et al*: **Out-of-hospital hypertonic resuscitation following severe traumatic brain injury: a randomized controlled trial**. *JAMA* 2010, **304**(13):1455-1464.	2
28	Bulger EM, May S, Kerby JD, Emerson S, Stiell IG, Schreiber MA, Brasel KJ, Tisherman SA, Coimbra R, Rizoli S *et al*: **Out-of-hospital hypertonic resuscitation after traumatic hypovolemic shock: a randomized, placebo controlled trial**. *Ann Surg* 2011, **253**(3):431-441.	2
29	Delano MJ, Rizoli SB, Rhind SG, Cuschieri J, Junger W, Baker AJ, Dubick MA, Hoyt DB, Bulger EM: **Prehospital resuscitation of traumatic**	2

**R15 Type of fluid**		
**Reference**	**Citation**	**LoE**
	**hemorrhagic shock with hypertonic solutions worsens hypocoagulation and hyperfibrinolysis**. *Shock* 2015, **44**(1):25-31.	
30	de Crescenzo C, Gorouhi F, Salcedo ES, Galante JM: **Prehospital hypertonic fluid resuscitation for trauma patients: A systematic review and meta-analysis**. *J Trauma Acute Care Surg* 2017, **82**(5):956-962.	1
31	Wu MC, Liao TY, Lee EM, Chen YS, Hsu WT, Lee MG, Tsou PY, Chen SC, Lee CC: **Administration of hypertonic solutions for hemorrhagic shock: A systematic review and meta-analysis of clinical trials**. *Anesth Analg* 2017, **125**(5):1549-1557.	1
32	Brakenridge SC, Phelan HA, Henley SS, Golden RM, Kashner TM, Eastman AE, Sperry JL, Harbrecht BG, Moore EE, Cuschieri J *et al*: **Early blood product and crystalloid volume resuscitation: risk association with multiple organ dysfunction after severe blunt traumatic injury**. *J Trauma* 2011, **71**(2):299-305.	3
33	Spoerke N, Michalek J, Schreiber M, Brasel KJ, Vercruysse G, MacLeod J, Dutton RP, Duchesne JC, McSwain NE, Muskat P *et al*: **Crystalloid resuscitation improves survival in trauma patients receiving low ratios of fresh frozen plasma to packed red blood cells**. *J Trauma* 2011, **71**(2 Suppl 3):S380-383.	3

**R16 Erythrocytes**		
**Reference**	**Citation**	**LoE**
1	Cabrales P, Martini J, Intaglietta M, Tsai AG: **Blood viscosity maintains microvascular conditions during normovolemic anemia independent of blood oxygen-carrying capacity**. *Am J Physiol Heart Circ Physiol* 2006, **291**(2):H581-590.	4
2	Tanaka S, Escudier E, Hamada S, Harrois A, Leblanc PE, Vicaut E, Duranteau J: **Effect of RBC transfusion on sublingual microcirculation in hemorrhagic shock patients: A pilot study**. *Crit Care Med* 2017, **45**(2):e154-e160.	4
3	Cabrales P, Tsai AG: **Plasma viscosity regulates systemic and microvascular perfusion during acute extreme anemic conditions**. *Am J Physiol Heart Circ Physiol* 2006, **291**(5):H2445-2452.	4
4	Ellsworth ML, Ellis CG, Goldman D, Stephenson AH, Dietrich HH, Sprague RS: **Erythrocytes: oxygen sensors and modulators of vascular tone**. *Physiology (Bethesda)* 2009, **24**:107-116.	5
5	Peyrou V, Lormeau JC, Herault JP, Gaich C, Pfliegger AM, Herbert JM: **Contribution of erythrocytes to thrombin generation in whole blood**. *Thromb Haemost* 1999, **81**(3):400-406.	4
6	Bombeli T, Spahn DR: **Updates in perioperative coagulation: physiology and management of thromboembolism and haemorrhage**. *Br J Anaesth* 2004, **93**(2):275-287.	5
7	Valeri CR, Cassidy G, Pivacek LE, Ragno G, Lieberthal W, Crowley JP, Khuri SF, Loscalzo J: **Anemia-induced increase in the bleeding time: implications for treatment of nonsurgical blood loss**. *Transfusion* 2001, **41**(8):977-983.	4
8	Quaknine-Orlando B, Samama CM, Riou B, Bonnin P, Guillosson JJ, Beaumont JL, Coriat P: **Role of the hematocrit in a rabbit model of arterial thrombosis and bleeding**. *Anesthesiology* 1999, **90**(5):1454-1461.	4
9	Iwata H, Kaibara M: **Activation of factor IX by erythrocyte membranes causes intrinsic coagulation**. *Blood Coagul Fibrinolysis* 2002, **13**(6):489-496.	4
10	Iwata H, Kaibara M, Dohmae N, Takio K, Himeno R, Kawakami S: **Purification, identification, and characterization of elastase on erythrocyte membrane as factor IX-activating enzyme**. *Biochem Biophys Res Commun* 2004, **316**(1):65-70.	4
11	Iselin BM, Willimann PF, Seifert B, Casutt M, Bombeli T, Zalunardo MP, Pasch T, Spahn DR: **Isolated reduction of haematocrit does not compromise in vitro blood coagulation**. *Br J Anaesth* 2001, **87**(2):246-249.	4
12	Hajjar LA, Vincent JL, Galas FR, Nakamura RE, Silva CM, Santos MH, Fukushima J, Kalil Filho R, Sierra DB, Lopes NH *et al*: **Transfusion requirements after cardiac surgery: the TRACS randomized controlled trial**. *JAMA* 2010, **304**(14):1559-1567.	2
13	Hebert PC, Wells G, Blajchman MA, Marshall J, Martin C, Pagliarello G, Tweeddale M, Schweitzer I, Yetisir E: **A multicenter, randomized, controlled clinical trial of transfusion requirements in critical care. Transfusion Requirements in Critical Care Investigators, Canadian Critical Care Trials Group**. *N Engl J Med* 1999, **340**(6):409-417.	2
14	Holst LB, Haase N, Wetterslev J, Wernerman J, Guttormsen AB, Karlsson S, Johansson PI, Aneman A, Vang ML, Winding R *et al*: **Lower versus higher hemoglobin threshold for transfusion in septic shock**. *N Engl J Med* 2014, **371**(15):1381-1391.	2
15	Murphy GJ, Pike K, Rogers CA, Wordsworth S, Stokes EA, Angelini GD, Reeves BC, Investigators TI: **Liberal or restrictive transfusion after cardiac surgery**. *N Engl J Med* 2015, **372**(11):997-1008.	2
16	Villanueva C, Colomo A, Bosch A, Concepcion M, Hernandez-Gea V, Aracil C, Graupera I, Poca M, Alvarez-Urturi C, Gordillo J *et al*: **Transfusion strategies for acute upper gastrointestinal bleeding**. *N Engl J Med* 2013, **368**(1):11-21.	2

**R16 Erythrocytes**		
**Reference**	**Citation**	**LoE**
17	Mazer CD, Whitlock RP, Fergusson DA, Hall J, Belley-Cote E, Connolly K, Khanykin B, Gregory AJ, de Medicis E, McGuinness S *et al*: **Restrictive or liberal red-cell transfusion for cardiac surgery**. *N Engl J Med* 2017, **377**(22):2133-2144.	2
18	McIntyre L, Hebert PC, Wells G, Fergusson D, Marshall J, Yetisir E, Blajchman MJ, Canadian Critical Care Trials G: **Is a restrictive transfusion strategy safe for resuscitated and critically ill trauma patients?** *J Trauma* 2004, **57**(3):563-568; discussion 568.	2
19	Charles A, Shaikh AA, Walters M, Huehl S, Pomerantz R: **Blood transfusion is an independent predictor of mortality after blunt trauma**. *Am Surg* 2007, **73**(1):1-5.	3
20	Croce MA, Tolley EA, Claridge JA, Fabian TC: **Transfusions result in pulmonary morbidity and death after a moderate degree of injury**. *J Trauma* 2005, **59**(1):19-23; discussion 23-14.	3
21	Malone DL, Dunne J, Tracy JK, Putnam AT, Scalea TM, Napolitano LM: **Blood transfusion, independent of shock severity, is associated with worse outcome in trauma**. *J Trauma* 2003, **54**(5):898-905; discussion 905-897.	3
22	Robinson WP, 3rd, Ahn J, Stiffler A, Rutherford EJ, Hurd H, Zarzaur BL, Baker CC, Meyer AA, Rich PB: **Blood transfusion is an independent predictor of increased mortality in nonoperatively managed blunt hepatic and splenic injuries**. *J Trauma* 2005, **58**(3):437-444; discussion 444-435.	3
23	Weinberg JA, McGwin G, Jr., Marques MB, Cherry SA, 3rd, Reiff DA, Kerby JD, Rue LW, 3rd: **Transfusions in the less severely injured: does age of transfused blood affect outcomes?** *J Trauma* 2008, **65**(4):794-798.	3
24	Chaiwat O, Lang JD, Vavilala MS, Wang J, MacKenzie EJ, Jurkovich GJ, Rivara FP: **Early packed red blood cell transfusion and acute respiratory distress syndrome after trauma**. *Anesthesiology* 2009, **110**(2):351-360.	3
25	Silverboard H, Aisiku I, Martin GS, Adams M, Rozycki G, Moss M: **The role of acute blood transfusion in the development of acute respiratory distress syndrome in patients with severe trauma**. *J Trauma* 2005, **59**(3):717-723.	3
26	Claridge JA, Sawyer RG, Schulman AM, McLemore EC, Young JS: **Blood transfusions correlate with infections in trauma patients in a dose-dependent manner**. *Am Surg* 2002, **68**(7):566-572.	3
27	Marik PE, Corwin HL: **Efficacy of red blood cell transfusion in the critically ill: a systematic review of the literature**. *Crit Care Med* 2008, **36**(9):2667-2674.	1
28	Bellapart J, Boots R, Fraser J: **Physiopathology of anemia and transfusion thresholds in isolated head injury**. *J Trauma Acute Care Surg* 2012, **73**(4):997-1005.	1
29	Desjardins P, Turgeon AF, Tremblay MH, Lauzier F, Zarychanski R, Boutin A, Moore L, McIntyre LA, English SW, Rigamonti A *et al*: **Hemoglobin levels and transfusions in neurocritically ill patients: a systematic review of comparative studies**. *Crit Care* 2012, **16**(2):R54.	1
30	Elterman J, Brasel K, Brown S, Bulger E, Christenson J, Kerby JD, Kannas D, Lin S, Minei JP, Rizoli S *et al*: **Transfusion of red blood cells in patients with a prehospital Glasgow Coma Scale score of 8 or less and no evidence of shock is associated with worse outcomes**. *J Trauma Acute Care Surg* 2013, **75**(1):8-14; discussion 14.	3
31	Robertson CS, Hannay HJ, Yamal JM, Gopinath S, Goodman JC, Tilley BC, Epo Severe TBITI, Baldwin A, Rivera Lara L, Saucedo-Crespo H *et al*: **Effect of erythropoietin and transfusion threshold on neurological recovery after traumatic brain injury: a randomized clinical trial**. *JAMA* 2014, **312**(1):36-47.	2
32	Hobisch-Hagen P, Wiedermann F, Mayr A, Fries D, Jelkmann W, Fuchs D, Hasibeder W, Mutz N, Klingler A, Schobersberger W: **Blunted**	3

**R16 Erythrocytes**		
**Reference**	**Citation**	**LoE**
	**erythropoietic response to anemia in multiply traumatized patients**. *Crit Care Med* 2001, **29**(4):743-747.	
33	Corwin HL, Gettinger A, Pearl RG, Fink MP, Levy MM, Shapiro MJ, Corwin MJ, Colton T, Group EPOCCT: **Efficacy of recombinant human erythropoietin in critically ill patients: a randomized controlled trial**. *JAMA* 2002, **288**(22):2827-2835.	2
34	Corwin HL, Gettinger A, Fabian TC, May A, Pearl RG, Heard S, An R, Bowers PJ, Burton P, Klausner MA *et al*: **Efficacy and safety of epoetin alfa in critically ill patients**. *N Engl J Med* 2007, **357**(10):965-976.	2
35	Nichol A, French C, Little L, Haddad S, Presneill J, Arabi Y, Bailey M, Cooper DJ, Duranteau J, Huet O *et al*: **Erythropoietin in traumatic brain injury (EPO-TBI): a double-blind randomised controlled trial**. *Lancet* 2015, **386**(10012):2499-2506.	2
36	French CJ, Glassford NJ, Gantner D, Higgins AM, Cooper DJ, Nichol A, Skrifvars MB, Imberger G, Presneill J, Bailey M *et al*: **Erythropoiesis-stimulating Agents in Critically Ill Trauma Patients: A Systematic Review and Meta-analysis**. *Ann Surg* 2017, **265**(1):54-62.	1
37	Garcia-Erce JA, Cuenca J, Munoz M, Izuel M, Martinez AA, Herrera A, Solano VM, Martinez F: **Perioperative stimulation of erythropoiesis with intravenous iron and erythropoietin reduces transfusion requirements in patients with hip fracture. A prospective observational study**. *Vox Sang* 2005, **88**(4):235-243.	3
38	Munoz M, Gomez-Ramirez S, Cuenca J, Garcia-Erce JA, Iglesias-Aparicio D, Haman-Alcober S, Ariza D, Naveira E: **Very-short-term perioperative intravenous iron administration and postoperative outcome in major orthopedic surgery: a pooled analysis of observational data from 2547 patients**. *Transfusion* 2014, **54**(2):289-299.	4
39	Cuenca J, Garcia-Erce JA, Munoz M, Izuel M, Martinez AA, Herrera A: **Patients with pertrochanteric hip fracture may benefit from preoperative intravenous iron therapy: a pilot study**. *Transfusion* 2004, **44**(10):1447-1452.	4
40	Bernabeu-Wittel M, Romero M, Ollero-Baturone M, Aparicio R, Murcia-Zaragoza J, Rincon-Gomez M, Monte-Secades R, Melero-Bascones M, Rosso CM, Ruiz-Cantero A *et al*: **Ferric carboxymaltose with or without erythropoietin in anemic patients with hip fracture: a randomized clinical trial**. *Transfusion* 2016, **56**(9):2199-2211.	2
41	Investigators I, Litton E, Baker S, Erber WN, Farmer S, Ferrier J, French C, Gummer J, Hawkins D, Higgins A *et al*: **Intravenous iron or placebo for anaemia in intensive care: the IRONMAN multicentre randomized blinded trial : A randomized trial of IV iron in critical illness**. *Intensive Care Med* 2016, **42**(11):1715-1722.	2

R17 Temperature management		
**Reference**	**Citation**	**LoE**
1	Rossaint R, Bouillon B, Cerny V, Coats TJ, Duranteau J, Fernandez-Mondejar E, Filipescu D, Hunt BJ, Komadina R, Nardi G *et al*: **The European guideline on management of major bleeding and coagulopathy following trauma: fourth edition**. *Crit Care* 2016, **20**:100.	5
2	DeLoughery TG: **Coagulation defects in trauma patients: etiology, recognition, and therapy**. *Crit Care Clin* 2004, **20**(1):13-24.	5
3	Kutcher ME, Howard BM, Sperry JL, Hubbard AE, Decker AL, Cuschieri J, Minei JP, Moore EE, Brownstein BH, Maier RV *et al*: **Evolving beyond the vicious triad: Differential mediation of traumatic coagulopathy by injury, shock, and resuscitation**. *J Trauma Acute Care Surg* 2015, **78**(3):516-523.	2
4	Watts DD, Trask A, Soeken K, Perdue P, Dols S, Kaufmann C: **Hypothermic coagulopathy in trauma: effect of varying levels of hypothermia on enzyme speed, platelet function, and fibrinolytic activity**. *J Trauma* 1998, **44**(5):846-854.	1
5	Bernabei AF, Levison MA, Bender JS: **The effects of hypothermia and injury severity on blood loss during trauma laparotomy**. *J Trauma* 1992, **33**(6):835-839.	3
6	Hoey BA, Schwab CW: **Damage control surgery**. *Scand J Surg* 2002, **91**(1):92-103.	2
7	Reynolds BR, Forsythe RM, Harbrecht BG, Cuschieri J, Minei JP, Maier RV, Moore EE, Billiar EE, Peitzman AB, Sperry JL *et al*: **Hypothermia in massive transfusion: have we been paying enough attention to it?** *J Trauma Acute Care Surg* 2012, **73**(2):486-491.	3
8	Rubiano AM, Sanchez AI, Estebanez G, Peitzman A, Sperry J, Puyana JC: **The effect of admission spontaneous hypothermia on patients with severe traumatic brain injury**. *Injury* 2013, **44**(9):1219-1225.	1
9	Barthel ER, Pierce JR: **Steady-state and time-dependent thermodynamic modeling of the effect of intravenous infusion of warm and cold fluids**. *J Trauma Acute Care Surg* 2012, **72**(6):1590-1600.	3
10	Eddy VA, Morris JA, Jr., Cullinane DC: **Hypothermia, coagulopathy, and acidosis**. *Surg Clin North Am* 2000, **80**(3):845-854.	5
11	Watts DD, Roche M, Tricarico R, Poole F, Brown JJ, Jr., Colson GB, Trask AL, Fakhry SM: **The utility of traditional prehospital interventions in maintaining thermostasis**. *Prehosp Emerg Care* 1999, **3**(2):115-122.	3
12	Bennett BL, Holcomb JB: **Battlefield trauma-induced hypothermia: Transitioning the preferred method of casualty rewarming**. *Wilderness Environ Med* 2017, 28(2S):S82-S89.	5
13	Allen PB, Salyer SW, Dubick MA, Holcomb JB, Blackbourne LH: **Preventing hypothermia: comparison of current devices used by the US Army in an in vitro warmed fluid model**. *J Trauma* 2010, **69 Suppl 1**:S154-161.	2

R18 Damage control surgery		
**Reference**	**Citation**	**LoE**
1	Stone HH, Strom PR, Mullins RJ: **Management of the major coagulopathy with onset during laparotomy**. *Ann Surg* 1983, **197**(5):532-535.	3
2	Morris JA, Jr., Eddy VA, Blinman TA, Rutherford EJ, Sharp KW: **The staged celiotomy for trauma. Issues in unpacking and reconstruction**. *Ann Surg* 1993, **217**(5):576-584; discussion 584-576.	3
3	Hirshberg A, Dugas M, Banez EI, Scott BG, Wall MJ, Jr., Mattox KL: **Minimizing dilutional coagulopathy in exsanguinating hemorrhage: a computer simulation**. *J Trauma* 2003, **54**(3):454-463.	3
4	Shapiro MB, Jenkins DH, Schwab CW, Rotondo MF: **Damage control: collective review**. *J Trauma* 2000, **49**(5):969-978.	4
5	Asensio JA, McDuffie L, Petrone P, Roldan G, Forno W, Gambaro E, Salim A, Demetriades D, Murray J, Velmahos G *et al*: **Reliable variables in the exsanguinated patient which indicate damage control and predict outcome**. *Am J Surg* 2001, **182**(6):743-751.	4
6	Moore EE, Burch JM, Franciose RJ, Offner PJ, Biffl WL: **Staged physiologic restoration and damage control surgery**. *World J Surg* 1998, **22**(12):1184-1190; discussion 1190-1181.	3
7	Johnson JW, Gracias VH, Schwab CW, Reilly PM, Kauder DR, Shapiro MB, Dabrowski GP, Rotondo MF: **Evolution in damage control for exsanguinating penetrating abdominal injury**. *J Trauma* 2001, **51**(2):261-269; discussion 269-271.	4
8	Rotondo MF, Schwab CW, McGonigal MD, Phillips GR, 3rd, Fruchterman TM, Kauder DR, Latenser BA, Angood PA: **'Damage control': an approach for improved survival in exsanguinating penetrating abdominal injury**. *J Trauma* 1993, **35**(3):375-382; discussion 382-373.	3
9	Braslow B: **Damage control in abdominal trauma**. *Contemp Surgery* 2006, **62**:65-74.	4
10	Hsu JM, Pham TN: **Damage control in the injured patient**. *Int J Crit Illn Inj Sci* 2011, **1**(1):66-72.	4
11	Scalea TM, Boswell SA, Scott JD, Mitchell KA, Kramer ME, Pollak AN: **External fixation as a bridge to intramedullary nailing for patients with multiple injuries and with femur fractures: damage control orthopedics**. *J Trauma* 2000, **48**(4):613-621; discussion 621-613.	4
12	Pape HC, Rixen D, Morley J, Husebye EE, Mueller M, Dumont C, Gruner A, Oestern HJ, Bayeff-Filoff M, Garving C *et al*: **Impact of the method of initial stabilization for femoral shaft fractures in patients with multiple injuries at risk for complications (borderline patients)**. *Ann Surg* 2007, **246**(3):491-499; discussion 499-501.	3
13	Caba-Doussoux P, Leon-Baltasar JL, Garcia-Fuentes C, Resines-Erasun C: **Damage control orthopaedics in severe polytrauma with femur fracture**. *Injury* 2012, **43 Suppl 2**:S42-46.	4
14	Wall MJ, Jr., Soltero E: **Damage control for thoracic injuries**. *Surg Clin North Am* 1997, **77**(4):863-878.	4
15	Rosenfeld JV: **Damage control neurosurgery**. *Injury* 2004, **35**(7):655-660.	4

R19-20 Pelvic ring closure and stabilisation - Packing, embolisation and surgery		
**Reference**	**Citation**	**LoE**
1	Brenner M, Hoehn M, Rasmussen TE: **Endovascular therapy in trauma**. *European Journal of Trauma and Emergency Surgery* 2014, **40**(6):671-678.	3
2	Ertel W, Keel M, Eid K, Platz A, Trentz O: **Control of severe hemorrhage using C-clamp and pelvic packing in multiply injured patients with pelvic ring disruption**. *J Orthop Trauma* 2001, **15**(7):468-474.	4
3	Costantini TW, Coimbra R, Holcomb JB, Podbielski JM, Catalano R, Blackburn A, Scalea TM, Stein DM, Williams L, Conflitti J *et al*: **Current management of hemorrhage from severe pelvic fractures: Results of an American Association for the Surgery of Trauma multi-institutional trial**. *J Trauma Acute Care Surg* 2016, **80**(5):717-723; discussion 723-715.	3
4	Scemama U, Dabadie A, Varoquaux A, Soussan J, Gaudon C, Louis G, Chaumoitre K, Vidal V: **Pelvic trauma and vascular emergencies**. *Diagn Interv Imaging* 2015, **96**(7-8):717-729.	4
5	Skitch S, Engels PT: **Acute management of the traumatically injured pelvis**. *Emerg Med Clin North Am* 2018, **36**(1):161-179.	3
6	Hagiwara A, Minakawa K, Fukushima H, Murata A, Masuda H, Shimazaki S: **Predictors of death in patients with life-threatening pelvic hemorrhage after successful transcatheter arterial embolization**. *J Trauma* 2003, **55**(4):696-703.	4
7	Verbeek DO, Zijlstra IA, van der Leij C, Ponsen KJ, van Delden OM, Goslings JC: **Management of pelvic ring fracture patients with a pelvic "blush" on early computed tomography**. *J Trauma Acute Care Surg* 2014, **76**(2):374-379.	4
8	Verbeek DO, Zijlstra IA, van der Leij C, Ponsen KJ, van Delden OM, Goslings JC: **Predicting the need for abdominal hemorrhage control in major pelvic fracture patients: the importance of quantifying the amount of free fluid**. *J Trauma Acute Care Surg* 2014, **76**(5):1259-1263.	3
9	Hornez E, Monchal T, Boddaert G, Chiron P, Danis J, Baudoin Y, Daban JL, Balandraud P, Bonnet S: **Penetrating pelvic trauma: Initial assessment and surgical management in emergency**. *J Visc Surg* 2016, **153**(4 Suppl):79-90.	3
10	Toth L, King KL, McGrath B, Balogh ZJ: **Efficacy and safety of emergency non-invasive pelvic ring stabilisation**. *Injury* 2012, **43**(8):1330-1334.	4
11	Tiemann AH, Schmidt C, Gonschorek O, Josten C: **[Use of the "c-clamp" in the emergency treatment of unstable pelvic fractures]**. *Zentralbl Chir* 2004, **129**(4):245-251.	4
12	Bakhshayesh P, Boutefnouchet T, Totterman A: **Effectiveness of non invasive external pelvic compression: a systematic review of the literature**. *Scand J Trauma Resusc Emerg Med* 2016, **24**:73.	1
13	Osborn PM, Smith WR, Moore EE, Cothren CC, Morgan SJ, Williams AE, Stahel PF: **Direct retroperitoneal pelvic packing versus pelvic angiography: A comparison of two management protocols for haemodynamically unstable pelvic fractures**. *Injury* 2009, **40**(1):54-60.	3
14	Smith WR, Moore EE, Osborn P, Agudelo JF, Morgan SJ, Parekh AA, Cothren C: **Retroperitoneal packing as a resuscitation technique for hemodynamically unstable patients with pelvic fractures: report of two representative cases and a description of technique**. *J Trauma* 2005, **59**(6):1510-1514.	4
15	Totterman A, Madsen JE, Skaga NO, Roise O: **Extraperitoneal pelvic packing: a salvage procedure to control massive traumatic pelvic hemorrhage**. *J Trauma* 2007, **62**(4):843-852.	4
16	Michailidou M, Velmahos GC, van der Wilden GM, Alam HB, de Moya M, Chang Y: **"Blush" on trauma computed tomography: not as bad as we think!** *J Trauma Acute Care Surg* 2012, **73**(3):580-584; discussion 584-586.	4
17	Lauerman MH, Dubose J, Cunningham K, Bruns B, Bradley M, Diaz J, Scalea T, Stein D: **Delayed interventions and mortality in trauma damage control laparotomy**. *Surgery* 2016, **160**(6):1568-1575.	3

**R19-20 Pelvic ring closure and stabilisation - Packing, embolisation and surgery**		
**Reference**	**Citation**	**LoE**
18	Manley JD, Mitchell BJ, DuBose JJ, Rasmussen TE: **A modern case series of resuscitative endovascular balloon occlusion of the aorta (REBOA) in an out-of-hospital, combat casualty care setting**. *J Spec Oper Med*, **17**(1):1-8.	4
19	Maruhashi T, Minehara H, Takeuchi I, Kataoka Y, Asari Y: **Resuscitative endovascular balloon occlusion of the aorta may increase the bleeding of minor thoracic injury in severe multiple trauma patients: a case report**. *J Med Case Rep* 2017, **11**(1):347.	5
20	Matsumoto H, Hara Y, Yagi T, Saito N, Mashiko K, Iida H, Motomura T, Nakayama F, Okada K, Yasumatsu H *et al*: **Impact of urgent resuscitative surgery for life-threatening torso trauma**. *Surg Today* 2017, **47**(7):827-835.	4
21	Verbeek D, Sugrue M, Balogh Z, Cass D, Civil I, Harris I, Kossmann T, Leibman S, Malka V, Pohl A *et al*: **Acute management of hemodynamically unstable pelvic trauma patients: time for a change? Multicenter review of recent practice**. *World J Surg* 2008, **32**(8):1874-1882.	3
22	Tanizaki S, Maeda S, Matano H, Sera M, Nagai H, Ishida H: **Time to pelvic embolization for hemodynamically unstable pelvic fractures may affect the survival for delays up to 60 min**. *Injury* 2014, **45**(4):738-741.	4
23	Geeraerts T, Chhor V, Cheisson G, Martin L, Bessoud B, Ozanne A, Duranteau J: **Clinical review: initial management of blunt pelvic trauma patients with haemodynamic instability**. *Crit Care* 2007, **11**(1):204.	3
24	Hauschild O, Aghayev E, von Heyden J, Strohm PC, Culemann U, Pohlemann T, Suedkamp NP, Schmal H: **Angioembolization for pelvic hemorrhage control: results from the German pelvic injury register**. *J Trauma Acute Care Surg* 2012, **73**(3):679-684.	3
25	van der Vlies CH, Saltzherr TP, Reekers JA, Ponsen KJ, van Delden OM, Goslings JC: **Failure rate and complications of angiography and embolization for abdominal and pelvic trauma**. *J Trauma Acute Care Surg* 2012, **73**(5):1208-1212.	4
26	Banerjee A, Duane TM, Wilson SP, Haney S, O'Neill PJ, Evans HL, Como JJ, Claridge JA: **Trauma center variation in splenic artery embolization and spleen salvage: a multicenter analysis**. *J Trauma Acute Care Surg* 2013, **75**(1):69-74; discussion 74-65.	3
27	Hagiwara A, Sakaki S, Goto H, Takenega K, Fukushima H, Matuda H, Shimazaki S: **The role of interventional radiology in the management of blunt renal injury: a practical protocol**. *J Trauma* 2001, **51**(3):526-531.	4
28	Hagiwara A, Yanagawa Y, Kaneko N, Takasu A, Hatanaka K, Sakamoto T, Okada Y: **Indications for transcatheter arterial embolization in persistent hemothorax caused by blunt trauma**. *J Trauma* 2008, **65**(3):589-594.	4
29	Nemoto C, Ikegami Y, Suzuki T, Tsukada Y, Abe Y, Shimada J, Tase C: **Repeated embolization of intercostal arteries after blunt chest injury**. *Gen Thorac Cardiovasc Surg* 2014, **62**(11):696-699.	4
30	Wang YC, Fu CY, Chen YF, Hsieh CH, Wu SC, Yeh CC: **Role of arterial embolization on blunt hepatic trauma patients with type I contrast extravasation**. *Am J Emerg Med* 2011, **29**(9):1147-1151.	4
31	Martinelli T, Thony F, Declety P, Sengel C, Broux C, Tonetti J, Payen JF, Ferretti G: **Intra-aortic balloon occlusion to salvage patients with life-threatening hemorrhagic shocks from pelvic fractures**. *J Trauma* 2010, **68**(4):942-948.	4
32	Morozumi J, Homma H, Ohta S, Noda M, Oda J, Mishima S, Yukioka T: **Impact of mobile angiography in the emergency department for controlling pelvic fracture hemorrhage with hemodynamic instability**. *J Trauma* 2010, **68**(1):90-95.	4
33	Brenner ML, Moore LJ, DuBose JJ, Tyson GH, McNutt MK, Albarado RP, Holcomb JB, Scalea TM, Rasmussen TE: **A clinical series of resuscitative endovascular balloon occlusion of the aorta for hemorrhage control and resuscitation**. *J Trauma Acute Care Surg* 2013, **75**(3):506-511.	4

**R21 Local haemostatic measures**		
**Reference**	**Citation**	**LoE**
1	Recinos G, Inaba K, Dubose J, Demetriades D, Rhee P: **Local and systemic hemostatics in trauma: a review**. *Ulus Travma Acil Cerrahi Derg* 2008, **14**(3):175-181.	3
2	Seyednejad H, Imani M, Jamieson T, Seifalian AM: **Topical haemostatic agents**. *Br J Surg* 2008, **95**(10):1197-1225.	3
3	CoStasis Multi-center Collaborative Writing Committee: **A novel collagen-based composite offers effective hemostasis for multiple surgical indications: Results of a randomized controlled trial**. *Surgery* 2001, **129**(4):445-450.	1
4	Drake DB, Wong LG: **Hemostatic effect of Vivostat patient-derived fibrin sealant on split-thickness skin graft donor sites**. *Ann Plast Surg* 2003, **50**(4):367-372.	3
5	King DR, Cohn SM, Proctor KG, Miami Clinical Trials G: **Modified rapid deployment hemostat bandage terminates bleeding in coagulopathic patients with severe visceral injuries**. *J Trauma* 2004, **57**(4):756-759.	2
6	Molloy DO, Archbold HA, Ogonda L, McConway J, Wilson RK, Beverland DE: **Comparison of topical fibrin spray and tranexamic acid on blood loss after total knee replacement: a prospective, randomised controlled trial**. *J Bone Joint Surg Br* 2007, **89**(3):306-309.	2
7	Ollinger R, Mihaljevic AL, Schuhmacher C, Bektas H, Vondran F, Kleine M, Sainz-Barriga M, Weiss S, Knebel P, Pratschke J *et al*: **A multicentre, randomized clinical trial comparing the Veriset haemostatic patch with fibrin sealant for the management of bleeding during hepatic surgery**. *HPB (Oxford)* 2013, **15**(7):548-558.	2
8	Oz MC, Cosgrove DM, 3rd, Badduke BR, Hill JD, Flannery MR, Palumbo R, Topic N: **Controlled clinical trial of a novel hemostatic agent in cardiac surgery. The Fusion Matrix Study Group**. *Ann Thorac Surg* 2000, **69**(5):1376-1382.	3
9	Pursifull NF, Morris MS, Harris RA, Morey AF: **Damage control management of experimental grade 5 renal injuries: further evaluation of FloSeal gelatin matrix**. *J Trauma* 2006, **60**(2):346-350.	3
10	Schenk WG, 3rd, Burks SG, Gagne PJ, Kagan SA, Lawson JH, Spotnitz WD: **Fibrin sealant improves hemostasis in peripheral vascular surgery: a randomized prospective trial**. *Ann Surg* 2003, **237**(6):871-876; discussion 876.	1
11	Sherman R, Chapman WC, Hannon G, Block JE: **Control of bone bleeding at the sternum and iliac crest donor sites using a collagen-based composite combined with autologous plasma: results of a randomized controlled trial**. *Orthopedics* 2001, **24**(2):137-141.	1
12	Smith KJ, Skelton HG, Barrett TL, Welch M, Beard J: **Histologic and immunohistochemical features in biopsy sites in which bovine collagen matrix was used for hemostasis**. *J Am Acad Dermatol* 1996, **34**(3):434-438.	4
13	Testini M, Marzaioli R, Lissidini G, Lippolis A, Logoluso F, Gurrado A, Lardo D, Poli E, Piccinni G: **The effectiveness of FloSeal matrix hemostatic agent in thyroid surgery: a prospective, randomized, control study**. *Langenbecks Arch Surg* 2009, **394**(5):837-842.	1
14	Weaver FA, Hood DB, Zatina M, Messina L, Badduke B: **Gelatin-thrombin-based hemostatic sealant for intraoperative bleeding in vascular surgery**. *Ann Vasc Surg* 2002, **16**(3):286-293.	3
15	Witte B, Kroeber SM, Hillebrand H, Wolf M, Huertgen M: **Cotton-derived oxidized cellulose in minimally invasive thoracic surgery: a clinicopathological study**. *Innovations (Phila)* 2013, **8**(4):296-301.	4
16	Woodworth BA, Chandra RK, LeBenger JD, Ilie B, Schlosser RJ: **A gelatin-thrombin matrix for hemostasis after endoscopic sinus surgery**. *Am J Otolaryngol* 2009, **30**(1):49-53.	4

**R21 Local haemostatic measures**		
**Reference**	**Citation**	**LoE**
17	Chapman WC, Clavien PA, Fung J, Khanna A, Bonham A: **Effective control of hepatic bleeding with a novel collagen-based composite combined with autologous plasma: results of a randomized controlled trial**. *Arch Surg* 2000, **135**(10):1200-1204; discussion 1205.	1
18	Choron RL, Hazelton JP, Hunter K, Capano-Wehrle L, Gaughan J, Chovanes J, Seamon MJ: **Intra-abdominal packing with laparotomy pads and QuikClot during damage control laparotomy: A safety analysis**. *Injury* 2017, **48**(1):158-164.	3

**R22 Antifibrinolytic agents**		
**Reference**	**Citation**	**LoE**
1	McCormack PL: **Tranexamic acid: a review of its use in the treatment of hyperfibrinolysis**. *Drugs* 2012, **72**(5):585-617.	1
2	Shakur H, Roberts I, Bautista R, Caballero J, Coats T, Dewan Y, El-Sayed H, Gogichaishvili T, Gupta S, Herrera J *et al*: **Effects of tranexamic acid on death, vascular occlusive events, and blood transfusion in trauma patients with significant haemorrhage (CRASH-2): a randomised, placebo-controlled trial**. *Lancet* 2010, **376**(9734):23-32.	2
3	Roberts I, Prieto-Merino D, Manno D: **Mechanism of action of tranexamic acid in bleeding trauma patients: an exploratory analysis of data from the CRASH-2 trial**. *Crit Care* 2014, **18**(6):685.	5
4	Roberts I, Shakur H, Ker K, Coats T, collaborators C-T: **Antifibrinolytic drugs for acute traumatic injury**. *Cochrane Database Syst Rev* 2012, **12**:CD004896.	1
5	Harvin JA, Peirce CA, Mims MM, Hudson JA, Podbielski JM, Wade CE, Holcomb JB, Cotton BA: **The impact of tranexamic acid on mortality in injured patients with hyperfibrinolysis**. *J Trauma Acute Care Surg* 2015, **78**(5):905-911.	4
6	Cole E, Davenport R, Willett K, Brohi K: **Tranexamic acid use in severely injured civilian patients and the effects on outcomes: a prospective cohort study**. *Ann Surg* 2015, **261**(2):390-394.	2
7	Henry DA, Carless PA, Moxey AJ, O'Connell D, Stokes BJ, Fergusson DA, Ker K: **Anti-fibrinolytic use for minimising perioperative allogeneic blood transfusion**. *Cochrane Database Syst Rev* 2011(1):CD001886.	1
8	Myles PS, Smith JA, Painter T: **Tranexamic acid in patients undergoing coronary-artery surgery**. *N Engl J Med* 2017, **376**(19):1893.	2
9	Poeran J, Rasul R, Suzuki S, Danninger T, Mazumdar M, Opperer M, Boettner F, Memtsoudis SG: **Tranexamic acid use and postoperative outcomes in patients undergoing total hip or knee arthroplasty in the United States: retrospective analysis of effectiveness and safety**. *BMJ* 2014, **349**:g4829.	3
10	Shakur H, Roberts I, Fawole B, Chaudhri R, El-Sheikh M, Akintan A, Qureshi Z, Kidanto H, Vwalika B, Abdulkadir A *et al*: **Effect of early tranexamic acid administration on mortality, hysterectomy, and other morbidities in women with post-partum haemorrhage (WOMAN): an international, randomised, double-blind, placebo-controlled trial**. *The Lancet* 2017, **389**(10084):2105-2116.	2
11	Kalavrouziotis D, Voisine P, Mohammadi S, Dionne S, Dagenais F: **High-dose tranexamic acid is an independent predictor of early seizure after cardiopulmonary bypass**. *Ann Thorac Surg* 2012, **93**(1):148-154.	4
12	Roberts I, Shakur H, Afolabi A, Brohi K, Coats T, Dewan Y, Gando S, Guyatt G, Hunt BJ, Morales C *et al*: **The importance of early treatment with tranexamic acid in bleeding trauma patients: an exploratory analysis of the CRASH-2 randomised controlled trial**. *Lancet* 2011, **377**(9771):1096-1101, 1101 e1091-1092.	2
13	Gayet-Ageron A, Prieto-Merino D, Ker K, Shakur H, Ageron FX, Roberts I, Antifibrinolytic Trials C: **Effect of treatment delay on the effectiveness and safety of antifibrinolytics in acute severe haemorrhage: a meta-analysis of individual patient-level data from 40 138 bleeding patients**. *Lancet* 2018, **391**(10116):125-132.	1
14	El-Menyar A, Sathian B, Asim M, Latifi R, Al-Thani H: **Efficacy of prehospital administration of tranexamic acid in trauma patients: A meta-analysis of the randomized controlled trials**. *Am J Emerg Med* 2018.	1
15	Stein P, Studt JD, Albrecht R, Muller S, von Ow D, Fischer S, Seifert B, Mariotti S, Spahn DR, Theusinger OM: **The impact of prehospital tranexamic acid on blood coagulation in trauma patients**. *Anesth Analg* 2018, **126**(2):522-529.	4

**R22 Antifibrinolytic agents**		
**Reference**	**Citation**	**LoE**
16	Roberts I, Perel P, Prieto-Merino D, Shakur H, Coats T, Hunt BJ, Lecky F, Brohi K, Willett K, on behalf of the C-c: **Effect of tranexamic acid on mortality in patients with traumatic bleeding: prespecified analysis of data from randomised controlled trial**. *BMJ* 2012, **345**:e5839.	2
17	Moore HB, Moore EE, Liras IN, Gonzalez E, Harvin JA, Holcomb JB, Sauaia A, Cotton BA: **Acute fibrinolysis shutdown after injury occurs frequently and increases mortality: A multicenter evaluation of 2,540 severely injured patients**. *J Am Coll Surg* 2016, **222**(4):347-355.	4
18	Raza I, Davenport R, Rourke C, Platton S, Manson J, Spoors C, Khan S, De'Ath HD, Allard S, Hart DP *et al*: **The incidence and magnitude of fibrinolytic activation in trauma patients**. *J Thromb Haemost* 2013, **11**(2):307-314.	3
19	Gall LS, Vulliamy P, Gillespie S, Jones TF, Pierre RSJ, Breukers SE, Gaarder C, Juffermans NP, Maegele M, Stensballe J *et al*: **The S100A10 pathway mediates an occult hyperfibrinolytic subtype in trauma patients**. *Annals of Surgery* 2018:1.	4
20	Guerriero C, Cairns J, Perel P, Shakur H, Roberts I: **Cost-effectiveness analysis of administering tranexamic acid to bleeding trauma patients using evidence from the CRASH-2 trial**. *PLoS One* 2011, **6**(5):e18987.	2
21	Roberts I, Shakur H, Coats T, Hunt B, Balogun E, Barnetson L, Cook L, Kawahara T, Perel P, Prieto-Merino D *et al*: **The CRASH-2 trial: a randomised controlled trial and economic evaluation of the effects of tranexamic acid on death, vascular occlusive events and transfusion requirement in bleeding trauma patients**. *Health Technol Assess* 2013, **17**(10):1-79.	2
22	Fergusson DA, Hebert PC, Mazer CD, Fremes S, MacAdams C, Murkin JM, Teoh K, Duke PC, Arellano R, Blajchman MA *et al*: **A comparison of aprotinin and lysine analogues in high-risk cardiac surgery**. *N Engl J Med* 2008, **358**(22):2319-2331.	2

**R23 Coagulation support**		
**Reference**	**Citation**	**LoE**
1	Levrat A, Gros A, Rugeri L, Inaba K, Floccard B, Negrier C, David JS: **Evaluation of rotation thrombelastography for the diagnosis of hyperfibrinolysis in trauma patients**. *British journal of anaesthesia* 2008, **100**(6):792-797.	3
2	Rugeri L, Levrat A, David JS, Delecroix E, Floccard B, Gros A, Allaouchiche B, Negrier C: **Diagnosis of early coagulation abnormalities in trauma patients by rotation thrombelastography**. *J Thromb Haemost* 2007, **5**(2):289-295.	3
3	Schöchl H, Frietsch T, Pavelka M, Jambor C: **Hyperfibrinolysis after major trauma: differential diagnosis of lysis patterns and prognostic value of thrombelastometry**. *J Trauma* 2009, **67**(1):125-131.	3
4	Theusinger OM, Baulig W, Seifert B, Emmert MY, Spahn DR, Asmis LM: **Relative concentrations of haemostatic factors and cytokines in solvent/detergent-treated and fresh-frozen plasma**. *British journal of anaesthesia* 2011, **106**(4):505-511.	5
5	Spahn DR: **TEG(R)- or ROTEM(R)-based individualized goal-directed coagulation algorithms: don't wait--act now!** *Crit Care* 2014, **18**(6):637.	5
6	Kashuk JL, Moore EE, Johnson JL, Haenel J, Wilson M, Moore JB, Cothren CC, Biffl WL, Banerjee A, Sauaia A: **Postinjury life threatening coagulopathy: is 1:1 fresh frozen plasma:packed red blood cells the answer?** *The Journal of trauma* 2008, **65**(2):261-270.	3
7	Innerhofer P, Fries D, Mittermayr M, Innerhofer N, von Langen D, Hell T, Gruber G, Schmid S, Friesenecker B, Lorenz IH *et al*: **Reversal of trauma-induced coagulopathy using first-line coagulation factor concentrates or fresh frozen plasma (RETIC): a single-centre, parallel-group, open-label, randomised trial**. *Lancet Haematol* 2017, **4**(6):e258-e271.	1
8	Nienaber U, Innerhofer P, Westermann I, Schöchl H, Attal R, Breitkopf R, Maegele M: **The impact of fresh frozen plasma vs coagulation factor concentrates on morbidity and mortality in trauma-associated haemorrhage and massive transfusion**. *Injury* 2011, **42**(7):697-701.	2
9	Schöchl H, Nienaber U, Maegele M, Hochleitner G, Primavesi F, Steitz B, Arndt C, Hanke A, Voelckel W, Solomon C: **Transfusion in trauma: thromboelastometry-guided coagulation factor concentrate-based therapy versus standard fresh frozen plasma-based therapy**. *Crit Care* 2011, **15**(2):R83.	2
10	Stein P, Kaserer A, Sprengel K, Wanner GA, Seifert B, Theusinger OM, Spahn DR: **Change of transfusion and treatment paradigm in major trauma patients**. *Anaesthesia* 2017, **72**(11):1317-1326.	2
11	Gonzalez E, Moore EE, Moore HB, Chapman MP, Chin TL, Ghasabyan A, Wohlauer MV, Barnett CC, Bensard DD, Biffl WL *et al*: **Goal-directed hemostatic resuscitation of trauma-induced coagulopathy: A pragmatic randomized clinical trial comparing a viscoelastic assay to conventional coagulation assays**. *Ann Surg* 2016, **263**(6):1051-1059.	1
12	Godier A, Bacus M, Kipnis E, Tavernier B, Guidat A, Rauch A, Drumez E, Susen S, Garrigue-Huet D: **Compliance with evidence-based clinical management guidelines in bleeding trauma patients**. *Br J Anaesth* 2016, **117**(5):592-600.	2
13	Riskin DJ, Tsai TC, Riskin L, Hernandez-Boussard T, Purtill M, Maggio PM, Spain DA, Brundage SI: **Massive transfusion protocols: the role of aggressive resuscitation versus product ratio in mortality reduction**. *Journal of the American College of Surgeons* 2009, **209**(2):198-205.	2
14	Schöchl H, Nienaber U, Hofer G, Voelckel W, Jambor C, Scharbert G, Kozek-Langenecker S, Solomon C: **Goal-directed coagulation management of major trauma patients using thromboelastometry (ROTEM)-guided administration of fibrinogen concentrate and prothrombin complex concentrate**. *Crit Care* 2010, **14**(2):R55.	2
15	Görlinger K, Dirkmann D, Hanke AA, Kamler M, Kottenberg E, Thielmann M, Jakob H, Peters J: **First-line therapy with coagulation factor concentrates**	2

R23 Coagulation support		
**Reference**	**Citation**	**LoE**
	**combined with point-of-care coagulation testing is associated with decreased allogeneic blood transfusion in cardiovascular surgery: a retrospective, single-center cohort study**. *Anesthesiology* 2011, **115**(6):1179-1191.	
16	Nakayama Y, Nakajima Y, Tanaka KA, Sessler DI, Maeda S, Iida J, Ogawa S, Mizobe T: **Thromboelastometry-guided intraoperative haemostatic management reduces bleeding and red cell transfusion after paediatric cardiac surgery**. *British journal of anaesthesia* 2015, **114**(1):91-102.	2
17	Weber CF, Gorlinger K, Meininger D, Herrmann E, Bingold T, Moritz A, Cohn LH, Zacharowski K: **Point-of-Care Testing: A Prospective, Randomized Clinical Trial of Efficacy in Coagulopathic Cardiac Surgery Patients**. *Anesthesiology* 2012, **117**(3):531-547.	1
18	Cotton BA, Au BK, Nunez TC, Gunter OL, Robertson AM, Young PP: **Predefined massive transfusion protocols are associated with a reduction in organ failure and postinjury complications**. *The Journal of trauma* 2009, **66**(1):41-48; discussion 48-49.	2
19	Maciel JD, Gifford E, Plurad D, de Virgilio C, Bricker S, Bongard F, Neville A, Smith J, Putnam B, Kim D: **The impact of a massive transfusion protocol on outcomes among patients with abdominal aortic injuries**. *Ann Vasc Surg* 2015, **29**(4):764-769.	2
20	Nascimento B, Callum J, Tien H, Rubenfeld G, Pinto R, Lin Y, Rizoli S: **Effect of a fixed-ratio (1:1:1) transfusion protocol versus laboratory-results-guided transfusion in patients with severe trauma: a randomized feasibility trial**. *Cmaj* 2013, **185**(12):E583-589.	1
21	Nardi G, Agostini V, Rondinelli B, Russo E, Bastianini B, Bini G, Bulgarelli S, Cingolani E, Donato A, Gambale G *et al*: **Trauma-induced coagulopathy: impact of the early coagulation support protocol on blood product consumption, mortality and costs**. *Crit Care* 2015, **19**(1):83.	2
22	Hendrickson JE, Shaz BH, Pereira G, Parker PM, Jessup P, Atwell F, Polstra B, Atkins E, Johnson KK, Bao G *et al*: **Implementation of a pediatric trauma massive transfusion protocol: one institution's experience**. *Transfusion* 2012, **52**(6):1228-1236.	2
23	Scalea TM, Bochicchio KM, Lumpkins K, Hess JR, Dutton R, Pyle A, Bochicchio GV: **Early aggressive use of fresh frozen plasma does not improve outcome in critically injured trauma patients**. *Annals of surgery* 2008, **248**(4):578-584.	2

**R24 Initial coagulation resuscitation**		
**Reference**	**Citation**	**LoE**
1	Holcomb JB, Jenkins D, Rhee P, Johannigman J, Mahoney P, Mehta S, Cox ED, Gehrke MJ, Beilman GJ, Schreiber M *et al*: **Damage control resuscitation: directly addressing the early coagulopathy of trauma**. *J Trauma* 2007, **62**(2):307-310.	5
2	Cannon JW, Khan MA, Raja AS, Cohen MJ, Como JJ, Cotton BA, Dubose JJ, Fox EE, Inaba K, Rodriguez CJ *et al*: **Damage control resuscitation in patients with severe traumatic hemorrhage: A practice management guideline from the Eastern Association for the Surgery of Trauma**. *J Trauma Acute Care Surg* 2017, **82**(3):605-617.	2
3	Innerhofer P, Fries D, Mittermayr M, Innerhofer N, von Langen D, Hell T, Gruber G, Schmid S, Friesenecker B, Lorenz IH *et al*: **Reversal of trauma-induced coagulopathy using first-line coagulation factor concentrates or fresh frozen plasma (RETIC): a single-centre, parallel-group, open-label, randomised trial**. *Lancet Haematol* 2017, **4**(6):e258-e271.	2
4	Henriksen HH, Rahbar E, Baer LA, Holcomb JB, Cotton BA, Steinmetz J, Ostrowski SR, Stensballe J, Johansson PI, Wade CE: **Pre-hospital transfusion of plasma in hemorrhaging trauma patients independently improves hemostatic competence and acidosis**. *Scand J Trauma Resusc Emerg Med* 2016, **24**(1):145.	3
5	Holcomb JB, Swartz MD, DeSantis SM, Greene TJ, Fox EE, Stein DM, Bulger EM, Kerby JD, Goodman M, Schreiber MA *et al*: **Multicenter observational prehospital resuscitation on helicopter study**. *J Trauma Acute Care Surg* 2017, **83**(1 Suppl 1):S83-S91.	3
6	Shlaifer A, Siman-Tov M, Radomislensky I, Peleg K, Shina A, Baruch EN, Glassberg E, Yitzhak A, Itg*: **Prehospital administration of freeze-dried plasma, is it the solution for trauma casualties?** *J Trauma Acute Care Surg* 2017, **83**(4):675-682.	4
7	Sunde GA, Vikenes B, Strandenes G, Flo KC, Hervig TA, Kristoffersen EK, Heltne JK: **Freeze dried plasma and fresh red blood cells for civilian prehospital hemorrhagic shock resuscitation**. *J Trauma Acute Care Surg* 2015, **78**(6 Suppl 1):S26-30.	4
8	A multicenter double-blind, placebo controlled, randomized, pilot trial to assess the efficacy of pre-hospital administration of fibrinogen concentrate (FGTW) in trauma patients, presumed to bleed (FI in TIC). [https://clinicaltrials.gov/ct2/show/record/NCT01475344]	N/A
9	Savage SA, Zarzaur BL, Croce MA, Fabian TC: **Time matters in 1: 1 resuscitations: concurrent administration of blood: plasma and risk of death**. *J Trauma Acute Care Surg* 2014, **77**(6):833-837; discussion 837-838.	3
10	Brown JB, Cohen MJ, Minei JP, Maier RV, West MA, Billiar TR, Peitzman AB, Moore EE, Cuschieri J, Sperry JL *et al*: **Goal-directed resuscitation in the prehospital setting: a propensity-adjusted analysis**. *J Trauma Acute Care Surg* 2013, **74**(5):1207-1212; discussion 1212-1204.	4
11	Holcomb JB, Tilley BC, Baraniuk S, Fox EE, Wade CE, Podbielski JM, del Junco DJ, Brasel KJ, Bulger EM, Callcut RA *et al*: **Transfusion of plasma, platelets, and red blood cells in a 1:1:1 vs a 1:1:2 ratio and mortality in patients with severe trauma: the PROPPR randomized clinical trial**. *JAMA* 2015, **313**(5):471-482.	2
12	Toy P, Popovsky MA, Abraham E, Ambruso DR, Holness LG, Kopko PM, McFarland JG, Nathens AB, Silliman CC, Stroncek D *et al*: **Transfusion-related acute lung injury: definition and review**. *Crit Care Med* 2005, **33**(4):721-726.	N/A
13	Schlimp CJ, Voelckel W, Inaba K, Maegele M, Ponschab M, Schochl H: **Estimation of plasma fibrinogen levels based on hemoglobin, base excess and Injury Severity Score upon emergency room admission**. *Crit Care* 2013, **17**(4):R137.	4
14	Rourke C, Curry N, Khan S, Taylor R, Raza I, Davenport R, Stanworth S, Brohi K: **Fibrinogen levels during trauma hemorrhage, response to**	3

**R24 Initial coagulation resuscitation**		
**Reference**	**Citation**	**LoE**
	**replacement therapy, and association with patient outcomes**. *J Thromb Haemost* 2012, **10**(7):1342-1351.	
15	Khan S, Davenport R, Raza I, Glasgow S, De'Ath HD, Johansson PI, Curry N, Stanworth S, Gaarder C, Brohi K: **Damage control resuscitation using blood component therapy in standard doses has a limited effect on coagulopathy during trauma hemorrhage**. *Intensive Care Med* 2015, **41**(2):239-247.	3
16	Kelly JM, Callum JL, Rizoli SB: **1:1:1 - Warranted or wasteful? Even where appropriate, high ratio transfusion protocols are costly: early transition to individualized care benefits patients and transfusion services**. *Expert Rev Hematol* 2013, **6**(6):631-633.	N/A
17	McQuilten ZK, Wood EM, Bailey M, Cameron PA, Cooper DJ: **Fibrinogen is an independent predictor of mortality in major trauma patients: A five-year statewide cohort study**. *Injury* 2017, **48**(5):1074-1081.	4
18	Ponschab M, Schochl H, Gabriel C, Sussner S, Cadamuro J, Haschke-Becher E, Gratz J, Zipperle J, Redl H, Schlimp CJ: **Haemostatic profile of reconstituted blood in a proposed 1:1:1 ratio of packed red blood cells, platelet concentrate and four different plasma preparations**. *Anaesthesia* 2015, **70**(5):528-536.	5
19	Allen CJ, Shariatmadar S, Meizoso JP, Hanna MM, Mora JL, Ray JJ, Namias N, Dudaryk R, Proctor KG: **Liquid plasma use during "super" massive transfusion protocol**. *J Surg Res* 2015, **199**(2):622-628.	4
20	Halmin M, Bostrom F, Brattstrom O, Lundahl J, Wikman A, Ostlund A, Edgren G: **Effect of plasma-to-RBC ratios in trauma patients: a cohort study with time-dependent data***. *Crit Care Med* 2013, **41**(8):1905-1914.	4
21	Nardi G, Agostini V, Rondinelli B, Russo E, Bastianini B, Bini G, Bulgarelli S, Cingolani E, Donato A, Gambale G *et al*: **Trauma-induced coagulopathy: impact of the early coagulation support protocol on blood product consumption, mortality and costs**. *Crit Care* 2015, **19**(1):83.	3
22	Zentai C, Braunschweig T, Schnabel J, Rose M, Rossaint R, Grottke O: **Fibrinogen concentrate does not suppress endogenous fibrinogen synthesis in a 24-hour porcine trauma model**. *Anesthesiology* 2014, **121**(4):753-764.	5

**R25 Goal-directed therapy**		
**Reference**	**Citation**	**LoE**
1	Hanke AA, Horstmann H, Wilhelmi M: **Point-of-care monitoring for the management of trauma-induced bleeding**. *Curr Opin Anaesthesiol* 2017, **30**(2):250-256.	5
2	Inaba K, Rizoli S, Veigas PV, Callum J, Davenport R, Hess J, Maegele M, Viscoelastic Testing in Trauma Consensus P: **2014 Consensus conference on viscoelastic test-based transfusion guidelines for early trauma resuscitation: Report of the panel**. *J Trauma Acute Care Surg* 2015, **78**(6):1220-1229.	5
3	Levi M, Hunt BJ: **A critical appraisal of point-of-care coagulation testing in critically ill patients**. *J Thromb Haemost* 2015, **13**(11):1960-1967.	5
4	Maegele M, Nardi G, Schochl H: **Hemotherapy algorithm for the management of trauma-induced coagulopathy: the German and European perspective**. *Curr Opin Anaesthesiol* 2017, **30**(2):257-264.	5
5	Schochl H, Maegele M, Voelckel W: **Fixed ratio versus goal-directed therapy in trauma**. *Curr Opin Anaesthesiol* 2016, **29**(2):234-244.	5
6	Walsh M, Fritz S, Hake D, Son M, Greve S, Jbara M, Chitta S, Fritz B, Miller A, Bader MK *et al*: **Targeted Thromboelastographic (TEG) blood component and pharmacologic hemostatic therapy in traumatic and acquired coagulopathy**. *Curr Drug Targets* 2016, **17**(8):954-970.	5
7	Yeung MC, Tong SY, Tong PY, Cheung BH, Ng JY, Leung GK: **Use of viscoelastic haemostatic assay in emergency and elective surgery**. *Hong Kong Med J* 2015, **21**(1):45-51.	5
8	Hilbert-Carius P, Hofmann G, Stuttmann R: **[Hemoglobin-oriented and coagulation factor-based algorithm : Effect on transfusion needs and standardized mortality rate in massively transfused trauma patients]**. *Anaesthesist* 2015, **64**(11):828-838.	4
9	Nascimento B, Callum J, Tien H, Rubenfeld G, Pinto R, Lin Y, Rizoli S: **Effect of a fixed-ratio (1:1:1) transfusion protocol versus laboratory-results-guided transfusion in patients with severe trauma: a randomized feasibility trial**. *CMAJ* 2013, **185**(12):E583-589.	2
10	Bogert JN, Harvin JA, Cotton BA: **Damage control resuscitation**. *J Intensive Care Med* 2016, **31**(3):177-186.	5
11	Mamczak CN, Maloney M, Fritz B, Boyer B, Thomas S, Evans E, Ploplis VA, Castellino FJ, McCollester J, Walsh M: **Thromboelastography in orthopaedic trauma acute pelvic fracture resuscitation: A descriptive pilot study**. *J Orthop Trauma* 2016, **30**(6):299-305.	3
12	Prat NJ, Meyer AD, Ingalls NK, Trichereau J, DuBose JJ, Cap AP: **Rotational thromboelastometry significantly optimizes transfusion practices for damage control resuscitation in combat casualties**. *J Trauma Acute Care Surg* 2017, **83**(3):373-380.	4
13	Stephens CT, Gumbert S, Holcomb JB: **Trauma-associated bleeding: management of massive transfusion**. *Curr Opin Anaesthesiol* 2016, **29**(2):250-255.	5
14	Albrecht V, Schafer N, Sturmer EK, Driessen A, Betsche L, Schenk M, Maegele M: **Practice management of acute trauma haemorrhage and haemostatic disorders across German trauma centres.** *Eur J Trauma Emerg Surg* 2017, **43**(2):201-214.	5
15	Wafaisade A, Wyen H, Mutschler M, Lendemans S, Bouillon B, Flohe S, Paffrath T, Maegele M, Tjardes T, Probst C *et al*: **[Current practice in coagulation and transfusion therapy in multiple trauma patients: A German nation-wide online survey]**. *Unfallchirurg* 2015, **118**(12):1033-1040.	5
16	Harr JN, Moore EE, Chin TL, Chapman MP, Ghasabyan A, Stringham JR, Banerjee A, Silliman CC: **Viscoelastic hemostatic fibrinogen assays detect fibrinolysis early**. *Eur J Trauma Emerg Surg* 2015, **41**(1):49-56.	3
17	Whiting P, Al M, Westwood M, Ramos IC, Ryder S, Armstrong N, Misso K, Ross J, Severens J, Kleijnen J: **Viscoelastic point-of-care testing to assist with the diagnosis, management and monitoring of haemostasis: a**	1

**R25 Goal-directed therapy**		
**Reference**	**Citation**	**LoE**
	**systematic review and cost-effectiveness analysis**. *Health Technol Assess* 2015, **19**(58):1-228, v-vi.	
18	David JS, Imhoff E, Parat S, Augey L, Geay-Baillat MO, Incagnoli P, Tazarourte K: **[Use of thrombelastography to guide posttraumatic hemostatic therapy: More coagulation factor concentrates and less allogenic blood transfusion?]**. *Transfus Clin Biol* 2016, **23**(4):205-211.	3
19	Ponschab M, Voelckel W, Pavelka M, Schlimp CJ, Schochl H: **Effect of coagulation factor concentrate administration on ROTEM(R) parameters in major trauma**. *Scand J Trauma Resusc Emerg Med* 2015, **23**:84.	4
20	Spahn DR, Spahn GH, Stein P: **Indications and risks of fibrinogen in surgery and trauma**. *Semin Thromb Hemost* 2016, **42**(2):147-154.	5
21	Taylor JR, 3rd, Fox EE, Holcomb JB, Rizoli S, Inaba K, Schreiber MA, Brasel K, Scalea TM, Wade CE, Bulger E *et al*: **The hyperfibrinolytic phenotype is the most lethal and resource intense presentation of fibrinolysis in massive transfusion patients**. *J Trauma Acute Care Surg* 2018, **84**(1):25-30.	2
22	Moore EE, Moore HB, Gonzalez E, Sauaia A, Banerjee A, Silliman CC: **Rationale for the selective administration of tranexamic acid to inhibit fibrinolysis in the severely injured patient**. *Transfusion* 2016, **56 Suppl 2**:S110-114.	5
23	Schochl H, Maegele M, Solomon C, Gorlinger K, Voelckel W: **Early and individualized goal-directed therapy for trauma-induced coagulopathy**. *Scand J Trauma Resusc Emerg Med* 2012, **20**:15.	5
24	Einersen PM, Moore EE, Chapman MP, Moore HB, Gonzalez E, Silliman CC, Banerjee A, Sauaia A: **Rapid thrombelastography thresholds for goal-directed resuscitation of patients at risk for massive transfusion**. *J Trauma Acute Care Surg* 2017, **82**(1):114-119.	4
25	Johansson PI, Stensballe J, Oliveri R, Wade CE, Ostrowski SR, Holcomb JB: **How I treat patients with massive hemorrhage**. *Blood* 2014, **124**(20):3052-3058.	5
26	Maegele M, Schochl H, Menovsky T, Marechal H, Marklund N, Buki A, Stanworth S: **Coagulopathy and haemorrhagic progression in traumatic brain injury: advances in mechanisms, diagnosis, and management**. *Lancet Neurol* 2017, **16**(8):630-647.	5
27	Rizoli S, Min A, Sanchez AP, Shek P, Grodecki R, Veigas P, Peng HT: **In trauma, conventional ROTEM and TEG results are not interchangeable but are similar in clinical applicability**. *Mil Med* 2016, **181**(5 Suppl):117-126.	2
28	David JS, Durand M, Levrat A, Lefevre M, Rugeri L, Geay-Baillat MO, Inaba K, Bouzat P: **Correlation between laboratory coagulation testing and thromboelastometry is modified during management of trauma patients**. *J Trauma Acute Care Surg* 2016, **81**(2):319-327.	5
29	Hunt H, Stanworth S, Curry N, Woolley T, Cooper C, Ukoumunne O, Zhelev Z, Hyde C: **Thromboelastography (TEG) and rotational thromboelastometry (ROTEM) for trauma induced coagulopathy in adult trauma patients with bleeding**. *Cochrane Database Syst Rev* 2015(2):CD010438.	1
30	Wikkelso A, Wetterslev J, Moller AM, Afshari A: **Thromboelastography (TEG) or thromboelastometry (ROTEM) to monitor haemostatic treatment versus usual care in adults or children with bleeding**. *Cochrane Database Syst Rev* 2016(8):CD007871.	1
31	Fahrendorff M, Oliveri RS, Johansson PI: **The use of viscoelastic haemostatic assays in goal-directing treatment with allogeneic blood products - A systematic review and meta-analysis**. *Scand J Trauma Resusc Emerg Med* 2017, **25**(1):39.	1
32	McQuilten ZK, Crighton G, Engelbrecht S, Gotmaker R, Brunskill SJ, Murphy MF, Wood EM: **Transfusion interventions in critical bleeding requiring**	1

R25 Goal-directed therapy		
**Reference**	**Citation**	**LoE**
	**massive transfusion: a systematic review**. *Transfus Med Rev* 2015, **29**(2):127-137.	
33	Gorlinger K, Saner FH: **Prophylactic plasma and platelet transfusion in the critically Ill patient: just useless and expensive or even harmful?** *BMC Anesthesiol* 2015, **15**:86.	5
34	Klages M, Zacharowski K, Weber CF: **Coagulation management in trauma-associated coagulopathy: allogenic blood products versus coagulation factor concentrates in trauma care**. *Curr Opin Anaesthesiol* 2016, **29**(2):245-249.	5
35	Curry N, Rourke C, Davenport R, Beer S, Pankhurst L, Deary A, Thomas H, Llewelyn C, Green L, Doughty H *et al*: **Early cryoprecipitate for major haemorrhage in trauma: a randomised controlled feasibility trial**. *Br J Anaesth* 2015, **115**(1):76-83.	2
36	Gonzalez E, Moore EE, Moore HB, Chapman MP, Chin TL, Ghasabyan A, Wohlauer MV, Barnett CC, Bensard DD, Biffl WL *et al*: **Goal-directed hemostatic resuscitation of trauma-induced coagulopathy: A pragmatic randomized clinical trial comparing a viscoelastic assay to conventional coagulation assays**. *Ann Surg* 2016, **263**(6):1051-1059.	2
37	Innerhofer P, Fries D, Mittermayr M, Innerhofer N, von Langen D, Hell T, Gruber G, Schmid S, Friesenecker B, Lorenz IH *et al*: **Reversal of trauma-induced coagulopathy using first-line coagulation factor concentrates or fresh frozen plasma (RETIC): a single-centre, parallel-group, open-label, randomised trial**. *Lancet Haematol* 2017, **4**(6):e258-e271.	2
38	Nardi G, Agostini V, Rondinelli B, Russo E, Bastianini B, Bini G, Bulgarelli S, Cingolani E, Donato A, Gambale G *et al*: **Trauma-induced coagulopathy: impact of the early coagulation support protocol on blood product consumption, mortality and costs**. *Crit Care* 2015, **19**(1):83.	4

R26 Fresh frozen plasma-based management		
**Reference**	**Citation**	**LoE**
1	Schott U, Solomon C, Fries D, Bentzer P: **The endothelial glycocalyx and its disruption, protection and regeneration: a narrative review**. *Scand J Trauma Resusc Emerg Med* 2016, **24**:48.	5
2	Holcomb JB, del Junco DJ, Fox EE, Wade CE, Cohen MJ, Schreiber MA, Alarcon LH, Bai Y, Brasel KJ, Bulger EM *et al*: **The prospective, observational, multicenter, major trauma transfusion (PROMMTT) study: comparative effectiveness of a time-varying treatment with competing risks**. *JAMA Surg* 2013, **148**(2):127-136.	2
3	Holcomb JB, Tilley BC, Baraniuk S, Fox EE, Wade CE, Podbielski JM, del Junco DJ, Brasel KJ, Bulger EM, Callcut RA *et al*: **Transfusion of plasma, platelets, and red blood cells in a 1:1:1 vs a 1:1:2 ratio and mortality in patients with severe trauma: the PROPPR randomized clinical trial**. *JAMA* 2015, **313**(5):471-482.	2
4	Inaba K, Branco BC, Rhee P, Blackbourne LH, Holcomb JB, Teixeira PG, Shulman I, Nelson J, Demetriades D: **Impact of plasma transfusion in trauma patients who do not require massive transfusion**. *J Am Coll Surg* 2010, **210**(6):957-965.	3
5	Johnson JL, Moore EE, Kashuk JL, Banerjee A, Cothren CC, Biffl WL, Sauaia A: **Effect of blood products transfusion on the development of postinjury multiple organ failure**. *Arch Surg* 2010, **145**(10):973-977.	3
6	Zhang LM, Li R, Zhao XC, Zhang Q, Luo XL: **Increased transfusion of fresh frozen plasma is associated with mortality or worse functional outcomes after severe traumatic brain injury: A retrospective study**. *World Neurosurg* 2017, **104**:381-389.	3
7	Eder AF, Dy BA, Perez JM, Rambaud M, Benjamin RJ: **The residual risk of transfusion-related acute lung injury at the American Red Cross (2008-2011): limitations of a predominantly male-donor plasma mitigation strategy**. *Transfusion* 2013, **53**(7):1442-1449.	2
8	Marietta M, Franchini M, Bindi ML, Picardi F, Ruggeri M, De Silvestro G: **Is solvent/detergent plasma better than standard fresh-frozen plasma? A systematic review and an expert consensus document**. *Blood Transfus* 2016, **14**(4):277-286.	2
9	Allen CJ, Shariatmadar S, Meizoso JP, Hanna MM, Mora JL, Ray JJ, Namias N, Dudaryk R, Proctor KG: **Liquid plasma use during "super" massive transfusion protocol**. *J Surg Res* 2015, **199**(2):622-628.	4
10	Stubbs JR, Zielinski MD, Berns KS, Badjie KS, Tauscher CD, Hammel SA, Zietlow SP, Jenkins D: **How we provide thawed plasma for trauma patients**. *Transfusion* 2015, **55**(8):1830-1837.	4
11	Zielinski MD, Johnson PM, Jenkins D, Goussous N, Stubbs JR: **Emergency use of prethawed Group A plasma in trauma patients**. *J Trauma Acute Care Surg* 2013, **74**(1):69-74; discussion 74-65.	3
12	Zielinski MD, Schrager JJ, Johnson P, Stubbs JR, Polites S, Zietlow SP, Jenkins DH, Robinson BR: **Multicenter comparison of emergency release group A versus AB plasma in blunt-injured trauma patients**. *Clin Transl Sci* 2015, **8**(1):43-47.	3
13	Stevens WT, Morse BC, Bernard A, Davenport DL, Sams VG, Goodman MD, Dumire R, Carrick MM, McCarthy P, Stubbs JR *et al*: **Incompatible type A plasma transfusion in patients requiring massive transfusion protocol: Outcomes of an Eastern Association for the Surgery of Trauma multicenter study**. *J Trauma Acute Care Surg* 2017, **83**(1):25-29.	3
14	Shlaifer A, Siman-Tov M, Radomislensky I, Peleg K, Shina A, Baruch EN, Glassberg E, Yitzhak A, Itg*: **Prehospital administration of freeze-dried plasma, is it the solution for trauma casualties?** *J Trauma Acute Care Surg* 2017, **83**(4):675-682.	4
15	Sunde GA, Vikenes B, Strandenes G, Flo KC, Hervig TA, Kristoffersen EK, Heltne JK: **Freeze dried plasma and fresh red blood cells for civilian prehospital hemorrhagic shock resuscitation**. *J Trauma Acute Care Surg* 2015, **78**(6 Suppl 1):S26-30.	4

**R26 Fresh frozen plasma-based management**		
**Reference**	**Citation**	**LoE**
16	Khan S, Brohi K, Chana M, Raza I, Stanworth S, Gaarder C, Davenport R, International Trauma Research N: **Hemostatic resuscitation is neither hemostatic nor resuscitative in trauma hemorrhage**. *J Trauma Acute Care Surg* 2014, **76**(3):561-567; discussion 567-568.	3
17	Khan S, Davenport R, Raza I, Glasgow S, De'Ath HD, Johansson PI, Curry N, Stanworth S, Gaarder C, Brohi K: **Damage control resuscitation using blood component therapy in standard doses has a limited effect on coagulopathy during trauma hemorrhage**. *Intensive Care Med* 2015, **41**(2):239-247.	3
18	Ponschab M, Schochl H, Gabriel C, Sussner S, Cadamuro J, Haschke-Becher E, Gratz J, Zipperle J, Redl H, Schlimp CJ: **Haemostatic profile of reconstituted blood in a proposed 1:1:1 ratio of packed red blood cells, platelet concentrate and four different plasma preparations**. *Anaesthesia* 2015, **70**(5):528-536.	5

**R27 Coagulation factor concentrate-based managment**		
**Reference**	**Citation**	**LoE**
1	Gall LS, Vulliamy P, Gillespie S, Jones TF, Pierre RSJ, Breukers SE, Gaarder C, Juffermans NP, Maegele M, Stensballe J *et al*: **The S100A10 Pathway Mediates an Occult Hyperfibrinolytic Subtype in Trauma Patients**. *Annals of surgery* 2018.	3
2	Davenport RA, Guerreiro M, Frith D, Rourke C, Platton S, Cohen M, Pearse R, Thiemermann C, Brohi K: **Activated Protein C Drives the Hyperfibrinolysis of Acute Traumatic Coagulopathy**. *Anesthesiology* 2017, **126**(1):115-127.	5
3	Hagemo JS, Christiaans SC, Stanworth SJ, Brohi K, Johansson PI, Goslings JC, Naess PA, Gaarder C: **Detection of acute traumatic coagulopathy and massive transfusion requirements by means of rotational thromboelastometry: an international prospective validation study**. *Crit Care* 2015, **19**(1):97.	3
4	Hagemo JS, Stanworth S, Juffermans NP, Brohi K, Cohen M, Johansson PI, Roislien J, Eken T, Naess PA, Gaarder C: **Prevalence, predictors and outcome of hypofibrinogenaemia in trauma: a multicentre observational study**. *Crit Care* 2014, **18**(2):R52.	3
5	Schochl H, Cotton B, Inaba K, Nienaber U, Fischer H, Voelckel W, Solomon C: **FIBTEM provides early prediction of massive transfusion in trauma**. *Crit Care* 2011, **15**(6):R265.	3
6	Rugeri L, Levrat A, David JS, Delecroix E, Floccard B, Gros A, Allaouchiche B, Negrier C: **Diagnosis of early coagulation abnormalities in trauma patients by rotation thrombelastography**. *J Thromb Haemost* 2007, **5**(2):289-295.	3
7	Rourke C, Curry N, Khan S, Taylor R, Raza I, Davenport R, Stanworth S, Brohi K: **Fibrinogen levels during trauma hemorrhage, response to replacement therapy, and association with patient outcomes**. *J Thromb Haemost* 2012, **10**(7):1342-1351.	3
8	Schlimp CJ, Voelckel W, Inaba K, Maegele M, Ponschab M, Schochl H: **Estimation of plasma fibrinogen levels based on hemoglobin, base excess and Injury Severity Score upon emergency room admission**. *Crit Care* 2013, **17**(4):R137.	3
9	Negrier C, Ducloy-Bouthors AS, Piriou V, De Maistre E, Stieltjes N, Borel-Derlon A, Colson P, Picard J, Lambert T, Claeyssens S *et al*: **Postauthorization safety study of Clottafact((R)) , a triply secured fibrinogen concentrate in acquired fibrinogen deficiency: a prospective observational study**. *Vox sanguinis* 2018, **113**(2):120-127.	3
10	Paydar S, Dalfardi B, Shayan Z, Shayan L, Saem J, Bolandparvaz S: **Early Predictive Factors of Hypofibrinogenemia in Acute Trauma Patients**. *J Emerg Trauma Shock* 2018, **11**(1):38-41.	3
11	Godier A, Bacus M, Kipnis E, Tavernier B, Guidat A, Rauch A, Drumez E, Susen S, Garrigue-Huet D: **Compliance with evidence-based clinical management guidelines in bleeding trauma patients**. *Br J Anaesth* 2016, **117**(5):592-600.	2
12	Stein P, Kaserer A, Sprengel K, Wanner GA, Seifert B, Theusinger OM, Spahn DR: **Change of transfusion and treatment paradigm in major trauma patients**. *Anaesthesia* 2017, **72**(11):1317-1326.	2
13	Stein P, Kaserer A, Spahn GH, Spahn DR: **Point-of-Care Coagulation Monitoring in Trauma Patients**. *Seminars in thrombosis and hemostasis* 2017, **43**(4):364-374.	5
14	Godier A, Greinacher A, Faraoni D, Levy JH, Samama CM: **Use of factor concentrates for the management of perioperative bleeding: guidance from the SSC of the ISTH**. *J Thromb Haemost* 2018, **16**(1):170-174.	5
15	Innerhofer P, Fries D, Mittermayr M, Innerhofer N, von Langen D, Hell T, Gruber G, Schmid S, Friesenecker B, Lorenz IH *et al*: **Reversal of trauma-induced coagulopathy using first-line coagulation factor concentrates or fresh frozen plasma (RETIC): a single-centre, parallel-group, open-label, randomised trial**. *The Lancet Haematology* 2017, **4(6)**:e258-e271.	1

**R27 Coagulation factor concentrate-based managment**		
**Reference**	**Citation**	**LoE**
16	Levy JH, Goodnough LT: **How I use fibrinogen replacement therapy in acquired bleeding**. *Blood* 2015, **125**(9):1387-1393.	5
17	Watson GA, Sperry JL, Rosengart MR, Minei JP, Harbrecht BG, Moore EE, Cuschieri J, Maier RV, Billiar TR, Peitzman AB: **Fresh frozen plasma is independently associated with a higher risk of multiple organ failure and acute respiratory distress syndrome**. *The Journal of trauma* 2009, **67**(2):221-227.	3
18	Chaiwat O, Lang JD, Vavilala MS, Wang J, MacKenzie EJ, Jurkovich GJ, Rivara FP: **Early packed red blood cell transfusion and acute respiratory distress syndrome after trauma**. *Anesthesiology* 2009, **110**(2):351-360.	3
19	Deras P, Villiet M, Manzanera J, Latry P, Schved JF, Capdevila X, Charbit J: **Early coagulopathy at hospital admission predicts initial or delayed fibrinogen deficit in severe trauma patients**. *J Trauma Acute Care Surg* 2014, **77**(3):433-440.	3
20	Schlimp CJ, Ponschab M, Voelckel W, Treichl B, Maegele M, Schochl H: **Fibrinogen levels in trauma patients during the first seven days after fibrinogen concentrate therapy: a retrospective study**. *Scand J Trauma Resusc Emerg Med* 2016, **24**:29.	3
21	Theusinger OM, Baulig W, Seifert B, Emmert MY, Spahn DR, Asmis LM: **Relative concentrations of haemostatic factors and cytokines in solvent/detergent-treated and fresh-frozen plasma**. *British journal of anaesthesia* 2011, **106**(4):505-511.	5
22	Wang Z, Liu H, Dou M, Du X, Hu J, Su N, Wang Y, Zhang R, Li C: **The quality changes in fresh frozen plasma of the blood donors at high altitude**. *PLoS One* 2017, **12**(4):e0176390.	5
23	Theusinger OM, Goslings D, Studt JD, Brand-Staufer B, Seifert B, Spahn DR, Frey BM: **Quarantine versus pathogen-reduced plasma-coagulation factor content and rotational thromboelastometry coagulation**. *Transfusion* 2017, **57**(3):637-645.	5
24	Williamson LM, Cardigan R, Prowse CV: **Methylene blue-treated fresh-frozen plasma: what is its contribution to blood safety?** *Transfusion* 2003, **43**(9):1322-1329.	5
25	Chunhui Y, Guohui B, Hong Y, Xiaopu X, Zherong B, Mingyuan W, Xinsheng Z, Juanjuan W, Changqing L, Wuping L: **Quantitative evaluation of plasma after methylene blue and white light treatment in four Chinese blood centers**. *Transfus Apher Sci* 2013, **49**(3):631-639.	5
26	Garrigue D, Godier A, Glacet A, Labreuche J, Kipnis E, Paris C, Duhamel A, Resch E, Bauters A, Machuron F *et al*: **French lyophilized plasma versus fresh frozen plasma for the initial management of trauma-induced coagulopathy: a randomized open-label trial**. *J Thromb Haemost* 2018, **16**(3):481-489.	2
27	Innerhofer P, Westermann I, Tauber H, Breitkopf R, Fries D, Kastenberger T, El Attal R, Strasak A, Mittermayr M: **The exclusive use of coagulation factor concentrates enables reversal of coagulopathy and decreases transfusion rates in patients with major blunt trauma**. *Injury* 2013, **44**(2):209-216.	3
28	Gorlinger K, Fries D, Dirkmann D, Weber CF, Hanke AA, Schochl H: **Reduction of Fresh Frozen Plasma Requirements by Perioperative Point-of-Care Coagulation Management with Early Calculated Goal-Directed Therapy**. *Transfus Med Hemother* 2012, **39**(2):104-113.	3
29	Sanders S, Tien H, Callum J, Nascimento B, Peng H, Funk C, Schmid J, Rizoli S, Rhind S, Beckett A: **Fibrinogen Concentrate in the Special Operations Forces Environment**. *Mil Med* 2018, **183**(1-2):e45-e50.	5
30	Edavettal M, Rogers A, Rogers F, Horst M, Leng W: **Prothrombin complex concentrate accelerates international normalized ratio reversal and diminishes the extension of intracranial hemorrhage in geriatric trauma patients**. *The American surgeon* 2014, **80**(4):372-376.	3

R27 Coagulation factor concentrate-based managment		
**Reference**	**Citation**	**LoE**
31	Huttner HB, Schellinger PD, Hartmann M, Kohrmann M, Juettler E, Wikner J, Mueller S, Meyding-Lamade U, Strobl R, Mansmann U *et al*: **Hematoma growth and outcome in treated neurocritical care patients with intracerebral hemorrhage related to oral anticoagulant therapy: comparison of acute treatment strategies using vitamin K, fresh frozen plasma, and prothrombin complex concentrates**. *Stroke; a journal of cerebral circulation* 2006, **37**(6):1465-1470.	3
32	Chai-Adisaksopha C, Hillis C, Siegal DM, Movilla R, Heddle N, Iorio A, Crowther M: **Prothrombin complex concentrates versus fresh frozen plasma for warfarin reversal. A systematic review and meta-analysis**. *Thromb Haemost* 2016, **116**(5):879-890.	1
33	Goldstein JN, Refaai MA, Milling TJ, Jr., Lewis B, Goldberg-Alberts R, Hug BA, Sarode R: **Four-factor prothrombin complex concentrate versus plasma for rapid vitamin K antagonist reversal in patients needing urgent surgical or invasive interventions: a phase 3b, open-label, non-inferiority, randomised trial**. *Lancet* 2015, **385**(9982):2077-2087.	1
34	Quinlan DJ, Eikelboom JW, Weitz JI: **Four-factor prothrombin complex concentrate for urgent reversal of vitamin K antagonists in patients with major bleeding**. *Circulation* 2013, **128**(11):1179-1181.	3
35	Sarode R, Milling TJ, Jr., Refaai MA, Mangione A, Schneider A, Durn BL, Goldstein JN: **Efficacy and safety of a 4-factor prothrombin complex concentrate in patients on vitamin K antagonists presenting with major bleeding: a randomized, plasma-controlled, phase IIIb study**. *Circulation* 2013, **128**(11):1234-1243.	3
36	Grassetto A, De Nardin M, Ganzerla B, Geremia M, Saggioro D, Serafini E, Zampieri S, Toffoli M, Penzo D, Bossi A *et al*: **ROTEM(R)-guided coagulation factor concentrate therapy in trauma: 2-year experience in Venice, Italy**. *Crit Care* 2012, **16**(3):428.	4
37	Ponschab M, Voelckel W, Pavelka M, Schlimp CJ, Schochl H: **Effect of coagulation factor concentrate administration on ROTEM(R) parameters in major trauma**. *Scand J Trauma Resusc Emerg Med* 2015, **23**:84.	3
38	Schöchl H, Forster L, Woidke R, Solomon C, Voelckel W: **Use of rotation thromboelastometry (ROTEM) to achieve successful treatment of polytrauma with fibrinogen concentrate and prothrombin complex concentrate**. *Anaesthesia* 2010, **65**(2):199-203.	3
39	Schochl H, Nienaber U, Maegele M, Hochleitner G, Primavesi F, Steitz B, Arndt C, Hanke A, Voelckel W, Solomon C: **Transfusion in trauma: thromboelastometry-guided coagulation factor concentrate-based therapy versus standard fresh frozen plasma-based therapy**. *Crit Care* 2011, **15**(2):R83.	3
40	Dunbar NM, Chandler WL: **Thrombin generation in trauma patients**. *Transfusion* 2009, **49**(12):2652-2660.	3
41	Schochl H, Voelckel W, Maegele M, Kirchmair L, Schlimp CJ: **Endogenous thrombin potential following hemostatic therapy with 4-factor prothrombin complex concentrate: a 7-day observational study of trauma patients**. *Crit Care* 2014, **18**(4):R147.	3
42	Schochl H, Nienaber U, Hofer G, Voelckel W, Jambor C, Scharbert G, Kozek-Langenecker S, Solomon C: **Goal-directed coagulation management of major trauma patients using thromboelastometry (ROTEM)-guided administration of fibrinogen concentrate and prothrombin complex concentrate**. *Crit Care* 2010, **14**(2):R55.	3
43	Schochl H, Voelckel W, Schlimp CJ: **Management of traumatic haemorrhage--the European perspective**. *Anaesthesia* 2015, **70 Suppl 1**:102-107, e135-107.	3
44	Dentali F, Marchesi C, Pierfranceschi MG, Crowther M, Garcia D, Hylek E, Witt DM, Clark NP, Squizzato A, Imberti D *et al*: **Safety of prothrombin complex concentrates for rapid anticoagulation reversal of vitamin K antagonists. A meta-analysis**. *Thromb Haemost* 2011, **106**(3):429-438.	1

R27 Coagulation factor concentrate-based managment		
**Reference**	**Citation**	**LoE**
45	Grottke O, Braunschweig T, Spronk HM, Esch S, Rieg AD, van Oerle R, ten Cate H, Fitzner C, Tolba R, Rossaint R: **Increasing concentrations of prothrombin complex concentrate induce disseminated intravascular coagulation in a pig model of coagulopathy with blunt liver injury**. *Blood* 2011, **118**(7):1943-1951.	5
46	Majeed A, Eelde A, Agren A, Schulman S, Holmstrom M: **Thromboembolic safety and efficacy of prothrombin complex concentrates in the emergency reversal of warfarin coagulopathy**. *Thrombosis research* 2012, **129**(2):146-151.	5
47	Pabinger I, Tiede A, Kalina U, Knaub S, Germann R, Ostermann H: **Impact of infusion speed on the safety and effectiveness of prothrombin complex concentrate: a prospective clinical trial of emergency anticoagulation reversal**. *Annals of hematology* 2010, **89**(3):309-316.	5
48	Park MS, Owen BA, Ballinger BA, Sarr MG, Schiller HJ, Zietlow SP, Jenkins DH, Ereth MH, Owen WG, Heit JA: **Quantification of hypercoagulable state after blunt trauma: microparticle and thrombin generation are increased relative to injury severity, while standard markers are not**. *Surgery* 2012, **151**(6):831-836.	5
49	Sorensen B, Spahn DR, Innerhofer P, Spannagl M, Rossaint R: **Clinical review: Prothrombin complex concentrates--evaluation of safety and thrombogenicity**. *Crit Care* 2011, **15**(1):201.	5
50	Joseph B, Khalil M, Harrison C, Swartz T, Kulvatunyou N, Haider AA, Jokar TO, Burk D, Mahmoud A, Latifi R *et al*: **Assessing the efficacy of prothrombin complex concentrate in multiply injured patients with high-energy pelvic and extremity fracturesa**. *J Orthop Trauma* 2016, **30**(12):653-658.	5
51	Younis M, Ray-Zack M, Haddad NN, Choudhry A, Hernandez MC, Wise K, Zielinski MD: **Prothrombin complex concentrate reversal of coagulopathy in emergency general surgery patients**. *World J Surg* 2018.	5
52	Schochl H, Schlimp CJ, Maegele M: **Tranexamic acid, fibrinogen concentrate, and prothrombin complex concentrate: data to support prehospital use?** *Shock* 2014, **41 Suppl 1**:44-46.	5
53	Mangram A, Oguntodu OF, Dzandu JK, Hollingworth AK, Hall S, Cung C, Rodriguez J, Yusupov I, Barletta JF: **Is there a difference in efficacy, safety, and cost-effectiveness between 3-factor and 4-factor prothrombin complex concentrates among trauma patients on oral anticoagulants?** *J Crit Care* 2016, **33**:252-256.	3
54	Turpie AG, Kreutz R, Llau J, Norrving B, Haas S: **Management consensus guidance for the use of rivaroxaban--an oral, direct factor Xa inhibitor**. *Thromb Haemost* 2012, **108**(5):876-886.	5
55	Philippou H, Adami A, Lane DA, MacGregor IR, Tuddenham EG, Lowe GD, Rumley A, Ludlam CA: **High purity factor IX and prothrombin complex concentrate (PCC): pharmacokinetics and evidence that factor IXa is the thrombogenic trigger in PCC**. *Thromb Haemost* 1996, **76**(1):23-28.	5
56	Dickneite G, Herwald H, Korte W, Allanore Y, Denton CP, Matucci Cerinic M: **Coagulation factor XIII: a multifunctional transglutaminase with clinical potential in a range of conditions**. *Thromb Haemost* 2015, **113**(4):686-697.	5
57	Hethershaw EL, Cilia La Corte AL, Duval C, Ali M, Grant PJ, Ariens RA, Philippou H: **The effect of blood coagulation factor XIII on fibrin clot structure and fibrinolysis**. *J Thromb Haemost* 2014, **12**(2):197-205.	5
58	von Rappard S, Hinnen C, Lussmann R, Rechsteiner M, Korte W: **Factor XIII deficiency and thrombocytopenia are frequent modulators of postoperative clot firmness in a surgical intensive care unit**. *Transfus Med Hemother* 2017, **44**(2):85-92.	5
59	Gerlach R, Tolle F, Raabe A, Zimmermann M, Siegemund A, Seifert V: **Increased risk for postoperative hemorrhage after intracranial surgery in patients with decreased factor XIII activity: implications of a prospective study**. *Stroke* 2002, **33**(6):1618-1623.	5

R27 Coagulation factor concentrate-based managment		
**Reference**	**Citation**	**LoE**
60	Gerlach R, Raabe A, Zimmermann M, Siegemund A, Seifert V: **Factor XIII deficiency and postoperative hemorrhage after neurosurgical procedures**. *Surg Neurol* 2000, **54**(3):260-264; discussion 264-265.	3
61	Shainoff JR, Estafanous FG, Yared JP, DiBello PM, Kottke-Marchant K, Loop FD: **Low factor XIIIA levels are associated with increased blood loss after coronary artery bypass grafting**. *J Thorac Cardiovasc Surg* 1994, **108**(3):437-445.	3
62	Godje O, Gallmeier U, Schelian M, Grunewald M, Mair H: **Coagulation factor XIII reduces postoperative bleeding after coronary surgery with extracorporeal circulation**. *Thorac Cardiovasc Surg* 2006, **54**(1):26-33.	3
63	Carcao M, Altisent C, Castaman G, Fukutake K, Kerlin BA, Kessler C, Lassila R, Nugent D, Oldenburg J, Garly ML *et al*: **Recombinant FXIII (rFXIII-A2) Prophylaxis Prevents Bleeding and Allows for Surgery in Patients with Congenital FXIII A-Subunit Deficiency**. *Thromb Haemost* 2018, **118**(3):451-460.	3
64	Levy JH, Gill R, Nussmeier NA, Olsen PS, Andersen HF, Booth FV, Jespersen CM: **Repletion of factor XIII following cardiopulmonary bypass using a recombinant A-subunit homodimer. A preliminary report**. *Thromb Haemost* 2009, **102**(4):765-771.	3
65	Karkouti K, von Heymann C, Jespersen CM, Korte W, Levy JH, Ranucci M, Sellke FW, Song HK: **Efficacy and safety of recombinant factor XIII on reducing blood transfusions in cardiac surgery: a randomized, placebo-controlled, multicenter clinical trial**. *J Thorac Cardiovasc Surg* 2013, **146**(4):927-939.	1
66	Lassila R: **Clinical Use of Factor XIII Concentrates**. *Semin Thromb Hemost* 2016, **42**(4):440-444.	5
67	Kozek-Langenecker SA, Ahmed AB, Afshari A, Albaladejo P, Aldecoa C, Barauskas G, De Robertis E, Faraoni D, Filipescu DC, Fries D *et al*: **Management of severe perioperative bleeding: guidelines from the European Society of Anaesthesiology: First update 2016**. *European jo*	5

**R28 Fibrinogen supplementation**		
**Reference**	**Citation**	**LoE**
1	Bombeli T, Spahn DR: **Updates in perioperative coagulation: physiology and management of thromboembolism and haemorrhage**. *Br J Anaesth* 2004, **93**(2):275-287.	4
2	Furie B, Furie BC: **Mechanisms of thrombus formation**. *N Engl J Med* 2008, **359**(9):938-949.	5
3	Nakamura Y, Ishikura H, Kushimoto S, Kiyomi F, Kato H, Sasaki J, Ogura H, Matsuoka T, Uejima T, Morimura N *et al*: **Fibrinogen level on admission is a predictor for massive transfusion in patients with severe blunt trauma: Analyses of a retrospective multicentre observational study**. *Injury* 2017, **48**(3):674-679.	2
4	Hiippala ST, Myllyla GJ, Vahtera EM: **Hemostatic factors and replacement of major blood loss with plasma-poor red cell concentrates**. *Anesth Analg* 1995, **81**(2):360-365.	3
5	McQuilten ZK, Bailey M, Cameron PA, Stanworth SJ, Venardos K, Wood EM, Cooper DJ: **Fibrinogen concentration and use of fibrinogen supplementation with cryoprecipitate in patients with critical bleeding receiving massive transfusion: a bi-national cohort study**. *Br J Haematol* 2017, **179**(1):131-141.	2
6	Ohmori T, Kitamura T, Tanaka K, Saisaka Y, Ishihara J, Onishi H, Nojima T, Yamamoto K, Matusmoto T, Tokioka T: **Admission fibrinogen levels in severe trauma patients: A comparison of elderly and younger patients**. *Injury* 2015, **46**(9):1779-1783.	3
7	Paydar S, Dalfardi B, Shayan Z, Shayan L, Saem J, Bolandparvaz S: **Early predictive factors of hypofibrinogenemia in acute trauma patients**. *J Emerg Trauma Shock* 2018, **11**(1):38-41.	3
8	Schlimp CJ, Voelckel W, Inaba K, Maegele M, Ponschab M, Schochl H: **Estimation of plasma fibrinogen levels based on hemoglobin, base excess and Injury Severity Score upon emergency room admission**. *Crit Care* 2013, **17**(4):R137.	3
9	Gonzalez-Guerrero C, Lozano-Andreu T, Roch-Santed M, Rivera-Sanchez L, Brandariz-Nunez D, Pasto-Cardona L, Juarez-Gimenez JC, Montoro-Ronsano JB: **Evaluation of the efficiency under current use of human fibrinogen concentrate in trauma patients with life-threatening hemorrhagic disorders**. *Blood Coagul Fibrinolysis* 2017, **28**(1):66-71.	3
10	Rourke C, Curry N, Khan S, Taylor R, Raza I, Davenport R, Stanworth S, Brohi K: **Fibrinogen levels during trauma hemorrhage, response to replacement therapy, and association with patient outcomes**. *J Thromb Haemost* 2012, **10**(7):1342-1351.	3
11	Rugeri L, Levrat A, David JS, Delecroix E, Floccard B, Gros A, Allaouchiche B, Negrier C: **Diagnosis of early coagulation abnormalities in trauma patients by rotation thrombelastography**. *J Thromb Haemost* 2007, **5**(2):289-295.	4
12	Schöchl H, Cotton B, Inaba K, Nienaber U, Fischer H, Voelckel W, Solomon C: **FIBTEM provides early prediction of massive transfusion in trauma**. *Crit Care* 2011, **15**(6):R265.	3
13	Charbit B, Mandelbrot L, Samain E, Baron G, Haddaoui B, Keita H, Sibony O, Mahieu-Caputo D, Hurtaud-Roux MF, Huisse MG *et al*: **The decrease of fibrinogen is an early predictor of the severity of postpartum hemorrhage**. *J Thromb Haemost* 2007, **5**(2):266-273.	3
14	Godier A, Greinacher A, Faraoni D, Levy JH, Samama CM: **Use of factor concentrates for the management of perioperative bleeding: guidance from the SSC of the ISTH**. *J Thromb Haemost* 2018, **16**(1):170-174.	1
15	Gorlinger K, Fries D, Dirkmann D, Weber CF, Hanke AA, Schochl H: **Reduction of fresh frozen plasma requirements by perioperative point-of-care coagulation management with early calculated goal-directed therapy**. *Transfus Med Hemother* 2012, **39**(2):104-113.	3
16	Weinstock N, Ntefidou M, Subcommittee ISF, Party GTHFW: **SSC International Collaborative Study to establish the first high fibrinogen**	5

R28 Fibrinogen supplementation		
**Reference**	**Citation**	**LoE**
	**plasma reference material for use with different fibrinogen assay techniques**. *J Thromb Haemost* 2006, **4**(8):1825-1827.	
17	Mackie IJ, Kitchen S, Machin SJ, Lowe GD, Haemostasis, Thrombosis Task Force of the British Committee for Standards in H: **Guidelines on fibrinogen assays**. *Br J Haematol* 2003, **121**(3):396-404.	1
18	Solomon C, Rahe-Meyer N, Schochl H, Ranucci M, Gorlinger K: **Effect of haematocrit on fibrin-based clot firmness in the FIBTEM test**. *Blood Transfus* 2013, **11**(3):412-418.	5
19	Schlimp CJ, Cadamuro J, Solomon C, Redl H, Schochl H: **The effect of fibrinogen concentrate and factor XIII on thromboelastometry in 33% diluted blood with albumin, gelatine, hydroxyethyl starch or saline in vitro**. *Blood Transfus* 2013, **11**(4):510-517.	5
20	Stinger HK, Spinella PC, Perkins JG, Grathwohl KW, Salinas J, Martini WZ, Hess JR, Dubick MA, Simon CD, Beekley AC *et al*: **The ratio of fibrinogen to red cells transfused affects survival in casualties receiving massive transfusions at an army combat support hospital**. *J Trauma* 2008, **64**(2 Suppl):S79-85; discussion S85.	3
21	Schöchl H, Nienaber U, Maegele M, Hochleitner G, Primavesi F, Steitz B, Arndt C, Hanke A, Voelckel W, Solomon C: **Transfusion in trauma: thromboelastometry-guided coagulation factor concentrate-based therapy versus standard fresh frozen plasma-based therapy**. *Crit Care* 2011, **15**(2):R83.	3
22	Schöchl H, Nienaber U, Hofer G, Voelckel W, Jambor C, Scharbert G, Kozek-Langenecker S, Solomon C: **Goal-directed coagulation management of major trauma patients using thromboelastometry (ROTEM)-guided administration of fibrinogen concentrate and prothrombin complex concentrate**. *Crit Care* 2010, **14**(2):R55.	3
23	Nardi G, Agostini V, Rondinelli B, Russo E, Bastianini B, Bini G, Bulgarelli S, Cingolani E, Donato A, Gambale G *et al*: **Trauma-induced coagulopathy: impact of the early coagulation support protocol on blood product consumption, mortality and costs**. *Crit Care* 2015, **19**(1):83.	4
24	Shaz BH, Dente CJ, Nicholas J, MacLeod JB, Young AN, Easley K, Ling Q, Harris RS, Hillyer CD: **Increased number of coagulation products in relationship to red blood cell products transfused improves mortality in trauma patients**. *Transfusion* 2010, **50**(2):493-500.	4
25	Innerhofer P, Fries D, Mittermayr M, Innerhofer N, von Langen D, Hell T, Gruber G, Schmid S, Friesenecker B, Lorenz IH *et al*: **Reversal of trauma-induced coagulopathy using first-line coagulation factor concentrates or fresh frozen plasma (RETIC): a single-centre, parallel-group, open-label, randomised trial**. *Lancet Haematol* 2017, **4**(6):e258-e271.	2
26	Olaussen A, Fitzgerald MC, Tan GA, Mitra B: **Cryoprecipitate administration after trauma**. *Eur J Emerg Med* 2016, **23**(4):269-273.	4
27	Morrison JJ, Ross JD, Dubose JJ, Jansen JO, Midwinter MJ, Rasmussen TE: **Association of cryoprecipitate and tranexamic acid with improved survival following wartime injury: findings from the MATTERs II Study**. *JAMA Surg* 2013, **148**(3):218-225.	3
28	Holcomb JB, Fox EE, Zhang X, White N, Wade CE, Cotton BA, del Junco DJ, Bulger EM, Cohen MJ, Schreiber MA *et al*: **Cryoprecipitate use in the PROMMTT study**. *J Trauma Acute Care Surg* 2013, **75**(1 Suppl 1):S31-39.	2
29	Khan S, Brohi K, Chana M, Raza I, Stanworth S, Gaarder C, Davenport R, International Trauma Research N: **Hemostatic resuscitation is neither hemostatic nor resuscitative in trauma hemorrhage**. *J Trauma Acute Care Surg* 2014, **76**(3):561-567; discussion 567-568.	2
30	Kozek-Langenecker SA, Afshari A, Albaladejo P, Santullano CA, De Robertis E, Filipescu DC, Fries D, Gorlinger K, Haas T, Imberger G *et al*: **Management of severe perioperative bleeding: guidelines from the European Society of Anaesthesiology**. *Eur J Anaesthesiol* 2013, **30**(6):270-382.	1

**R28 Fibrinogen supplementation**		
**Reference**	**Citation**	**LoE**
31	Meyer MA, Ostrowski SR, Sorensen AM, Meyer AS, Holcomb JB, Wade CE, Johansson PI, Stensballe J: **Fibrinogen in trauma, an evaluation of thrombelastography and rotational thromboelastometry fibrinogen assays**. *J Surg Res* 2015, **194**(2):581-590.	2
32	Curry N, Rourke C, Davenport R, Beer S, Pankhurst L, Deary A, Thomas H, Llewelyn C, Green L, Doughty H *et al*: **Early cryoprecipitate for major haemorrhage in trauma: a randomised controlled feasibility trial**. *Br J Anaesth* 2015, **115**(1):76-83.	2
33	Schöchl H, Voelckel W, Maegele M, Kirchmair L, Schlimp CJ: **Endogenous thrombin potential following hemostatic therapy with 4-factor prothrombin complex concentrate: a 7-day observational study of trauma patients**. *Crit Care* 2014, **18**(4):R147.	3
34	Thompson GH, Florentino-Pineda I, Armstrong DG, Poe-Kochert C: **Fibrinogen levels following Amicar in surgery for idiopathic scoliosis**. *Spine (Phila Pa 1976)* 2007, **32**(3):368-372.	4
35	Wei KL, Lin CJ, Lai KA: **Changes in coagulatory profile after orthopedic surgery**. *J Formos Med Assoc* 1995, **94**(9):541-547.	3
36	Solomon C, Hagl C, Rahe-Meyer N: **Time course of haemostatic effects of fibrinogen concentrate administration in aortic surgery**. *Br J Anaesth* 2013, **110**(6):947-956.	2
37	Karlsson M, Ternstrom L, Hyllner M, Baghaei F, Flinck A, Skrtic S, Jeppsson A: **Prophylactic fibrinogen infusion reduces bleeding after coronary artery bypass surgery. A prospective randomised pilot study**. *Thromb Haemost* 2009, **102**(1):137-144.	2
38	Jensen NH, Stensballe J, Afshari A: **Comparing efficacy and safety of fibrinogen concentrate to cryoprecipitate in bleeding patients: a systematic review**. *Acta Anaesthesiol Scand* 2016, **60**(8):1033-1042.	1

**R29 Platelets**		
**Reference**	**Citation**	**LoE**
1	Hess JR, Lindell AL, Stansbury LG, Dutton RP, Scalea TM: **The prevalence of abnormal results of conventional coagulation tests on admission to a trauma center**. *Transfusion* 2009, **49**(1):34-39.	3
2	Stansbury LG, Hess AS, Thompson K, Kramer B, Scalea TM, Hess JR: **The clinical significance of platelet counts in the first 24 hours after severe injury**. *Transfusion* 2013, **53**(4):783-789.	3
3	Hiippala ST, Myllyla GJ, Vahtera EM: **Hemostatic factors and replacement of major blood loss with plasma-poor red cell concentrates**. *Anesth Analg* 1995, **81**(2):360-365.	3
4	Schnuriger B, Inaba K, Abdelsayed GA, Lustenberger T, Eberle BM, Barmparas G, Talving P, Demetriades D: **The impact of platelets on the progression of traumatic intracranial hemorrhage**. *J Trauma* 2010, **68**(4):881-885.	3
5	Joseph B, Aziz H, Zangbar B, Kulvatunyou N, Pandit V, O'Keeffe T, Tang A, Wynne J, Friese RS, Rhee P: **Acquired coagulopathy of traumatic brain injury defined by routine laboratory tests: which laboratory values matter?** *J Trauma Acute Care Surg* 2014, **76**(1):121-125.	3
6	Joseph B, Pandit V, Meyer D, Butvidas L, Kulvatunyou N, Khalil M, Tang A, Zangbar B, O'Keeffe T, Gries L *et al*: **The significance of platelet count in traumatic brain injury patients on antiplatelet therapy**. *J Trauma Acute Care Surg* 2014, **77**(3):417-421.	3
7	Johansson PI, Stensballe J, Rosenberg I, Hilslov TL, Jorgensen L, Secher NH: **Proactive administration of platelets and plasma for patients with a ruptured abdominal aortic aneurysm: evaluating a change in transfusion practice**. *Transfusion* 2007, **47**(4):593-598.	3
8	Vulliamy P, Gillespie S, Gall LS, Green L, Brohi K, Davenport RA: **Platelet transfusions reduce fibrinolysis but do not restore platelet function during trauma hemorrhage**. *J Trauma Acute Care Surg* 2017, **83**(3):388-397.	3
9	Anglin CO, Spence JS, Warner MA, Paliotta C, Harper C, Moore C, Sarode R, Madden C, Diaz-Arrastia R: **Effects of platelet and plasma transfusion on outcome in traumatic brain injury patients with moderate bleeding diatheses**. *J Neurosurg* 2013, **118**(3):676-686.	3
10	Estcourt LJ, Birchall J, Allard S, Bassey SJ, Hersey P, Kerr JP, Mumford AD, Stanworth SJ, Tinegate H, British Committee for Standards in H: **Guidelines for the use of platelet transfusions**. *Br J Haematol* 2017, **176**(3):365-394.	5
11	Kaufman RM, Djulbegovic B, Gernsheimer T, Kleinman S, Tinmouth AT, Capocelli KE, Cipolle MD, Cohn CS, Fung MK, Grossman BJ *et al*: **Platelet transfusion: a clinical practice guideline from the AABB**. *Ann Intern Med* 2015, **162**(3):205-213.	5
12	Wohlauer MV, Moore EE, Thomas S, Sauaia A, Evans E, Harr J, Silliman CC, Ploplis V, Castellino FJ, Walsh M: **Early platelet dysfunction: an unrecognized role in the acute coagulopathy of trauma**. *J Am Coll Surg* 2012, **214**(5):739-746.	3
13	Jacoby RC, Owings JT, Holmes J, Battistella FD, Gosselin RC, Paglieroni TG: **Platelet activation and function after trauma**. *J Trauma* 2001, **51**(4):639-647.	3
14	Kutcher ME, Redick BJ, McCreery RC, Crane IM, Greenberg MD, Cachola LM, Nelson MF, Cohen MJ: **Characterization of platelet dysfunction after trauma**. *J Trauma Acute Care Surg* 2012, **73**(1):13-19.	3
15	Solomon C, Traintinger S, Ziegler B, Hanke A, Rahe-Meyer N, Voelckel W, Schöchl H: **Platelet function following trauma. A multiple electrode aggregometry study**. *Thromb Haemost* 2011, **106**(2):322-330.	4
16	Briggs A, Gates JD, Kaufman RM, Calahan C, Gormley WB, Havens JM: **Platelet dysfunction and platelet transfusion in traumatic brain injury**. *J Surg Res* 2015, **193**(2):802-806.	3

**R29 Platelets**		
**Reference**	**Citation**	**LoE**
17	Bachelani AM, Bautz JT, Sperry JL, Corcos A, Zenati M, Billiar TR, Peitzman AB, Marshall GT: **Assessment of platelet transfusion for reversal of aspirin after traumatic brain injury**. *Surgery* 2011, **150**(4):836-843.	4
18	Perkins JG, Cap AP, Spinella PC, Blackbourne LH, Grathwohl KW, Repine TB, Ketchum L, Waterman P, Lee RE, Beekley AC *et al*: **An evaluation of the impact of apheresis platelets used in the setting of massively transfused trauma patients**. *J Trauma* 2009, **66**(4 Suppl):S77-84; discussion S84-75.	3
19	Pidcoke HF, Aden JK, Mora AG, Borgman MA, Spinella PC, Dubick MA, Blackbourne LH, Cap AP: **Ten-year analysis of transfusion in Operation Iraqi Freedom and Operation Enduring Freedom: increased plasma and platelet use correlates with improved survival**. *J Trauma Acute Care Surg* 2012, **73**(6 Suppl 5):S445-452.	3
20	Holcomb JB, Wade CE, Michalek JE, Chisholm GB, Zarzabal LA, Schreiber MA, Gonzalez EA, Pomper GJ, Perkins JG, Spinella PC *et al*: **Increased plasma and platelet to red blood cell ratios improves outcome in 466 massively transfused civilian trauma patients**. *Ann Surg* 2008, **248**(3):447-458.	3
21	Holcomb JB, Zarzabal LA, Michalek JE, Kozar RA, Spinella PC, Perkins JG, Matijevic N, Dong JF, Pati S, Wade CE *et al*: **Increased platelet:RBC ratios are associated with improved survival after massive transfusion**. *J Trauma* 2011, **71**(2 Suppl 3):S318-328.	3
22	Inaba K, Lustenberger T, Rhee P, Holcomb JB, Blackbourne LH, Shulman I, Nelson J, Talving P, Demetriades D: **The impact of platelet transfusion in massively transfused trauma patients**. *J Am Coll Surg* 2010, **211**(5):573-579.	3
23	Shaz BH, Dente CJ, Nicholas J, MacLeod JB, Young AN, Easley K, Ling Q, Harris RS, Hillyer CD: **Increased number of coagulation products in relationship to red blood cell products transfused improves mortality in trauma patients**. *Transfusion* 2010, **50**(2):493-500.	3
24	Johansson PI, Oliveri RS, Ostrowski SR: **Hemostatic resuscitation with plasma and platelets in trauma**. *J Emerg Trauma Shock* 2012, **5**(2):120-125.	2
25	Hallet J, Lauzier F, Mailloux O, Trottier V, Archambault P, Zarychanski R, Turgeon AF: **The use of higher platelet: RBC transfusion ratio in the acute phase of trauma resuscitation: a systematic review**. *Crit Care Med* 2013, **41**(12):2800-2811.	2
26	Brown JB, Cohen MJ, Minei JP, Maier RV, West MA, Billiar TR, Peitzman AB, Moore EE, Cushieri J, Sperry JL *et al*: **Debunking the survival bias myth: Characterization of mortality during the initial 24 hours for patients requiring massive transfusion**. *J Trauma Acute Care Surg* 2012, **73**(2):358-364.	3
27	Ponschab M, Schochl H, Gabriel C, Sussner S, Cadamuro J, Haschke-Becher E, Gratz J, Zipperle J, Redl H, Schlimp CJ: **Haemostatic profile of reconstituted blood in a proposed 1:1:1 ratio of packed red blood cells, platelet concentrate and four different plasma preparations**. *Anaesthesia* 2015, **70**(5):528-536.	3
28	Rowell SE, Barbosa RR, Allison CE, Van PY, Schreiber MA, Trauma Outcomes G, Holcomb JB, Wade CE, Brasel KJ, Vercruysse G *et al*: **Gender-based differences in mortality in response to high product ratio massive transfusion**. *J Trauma* 2011, **71**(2 Suppl 3):S375-379.	3
29	Rowell SE, Barbosa RR, Diggs BS, Schreiber MA, Trauma Outcomes G, Holcomb JB, Wade CE, Brasel KJ, Vercruysse G, MacLeod J *et al*: **Effect of high product ratio massive transfusion on mortality in blunt and penetrating trauma patients**. *J Trauma* 2011, **71**(2 Suppl 3):S353-357.	3
30	Lustenberger T, Frischknecht A, Bruesch M, Keel MJ: **Blood component ratios in massively transfused, blunt trauma patients--a time-dependent**	3

**R29 Platelets**		
**Reference**	**Citation**	**LoE**
	**covariate analysis**. *J Trauma* 2011, **71**(5):1144-1150; discussion 1150-1141.	
31	Dirks J, Jorgensen H, Jensen CH, Ostrowski SR, Johansson PI: **Blood product ratio in acute traumatic coagulopathy--effect on mortality in a Scandinavian level 1 trauma centre**. *Scand J Trauma Resusc Emerg Med* 2010, **18**:65.	3
32	Brasel KJ, Vercruysse G, Spinella PC, Wade CE, Blackbourne LH, Borgman MA, Zarzabal LA, Du F, Perkins JG, Maegele M *et al*: **The association of blood component use ratios with the survival of massively transfused trauma patients with and without severe brain injury**. *J Trauma* 2011, **71**(2 Suppl 3):S343-352.	3
33	Sambasivan CN, Kunio NR, Nair PV, Zink KA, Michalek JE, Holcomb JB, Schreiber MA, Trauma Outcomes G, Wade CE, Brasel KJ *et al*: **High ratios of plasma and platelets to packed red blood cells do not affect mortality in nonmassively transfused patients**. *J Trauma* 2011, **71**(2 Suppl 3):S329-336.	3
34	Holcomb JB, del Junco DJ, Fox EE, Wade CE, Cohen MJ, Schreiber MA, Alarcon LH, Bai Y, Brasel KJ, Bulger EM *et al*: **The prospective, observational, multicenter, major trauma transfusion (PROMMTT) study: comparative effectiveness of a time-varying treatment with competing risks**. *JAMA Surg* 2013, **148**(2):127-136.	2
35	Peralta R, Vijay A, El-Menyar A, Consunji R, Afifi I, Mahmood I, Asim M, Latifi R, Al-Thani H: **Early high ratio platelet transfusion in trauma resuscitation and its outcomes**. *Int J Crit Illn Inj Sci* 2016, **6**(4):188-193.	3
36	Holcomb JB, Tilley BC, Baraniuk S, Fox EE, Wade CE, Podbielski JM, del Junco DJ, Brasel KJ, Bulger EM, Callcut RA *et al*: **Transfusion of plasma, platelets, and red blood cells in a 1:1:1 vs a 1:1:2 ratio and mortality in patients with severe trauma: the PROPPR randomized clinical trial**. *JAMA* 2015, **313**(5):471-482.	1
37	Dzik W: **Misunderstanding the PROPPR trial**. *Transfusion* 2017, **57**(8):2056.	5
38	McQuilten ZK, Crighton G, Brunskill S, Morison JK, Richter TH, Waters N, Murphy MF, Wood EM: **Optimal dose, timing and ratio of blood products in massive transfusion: results from a systematic review**. *Transfus Med Rev* 2018, **32**(1):6-15.	1
39	Henriksen HH, Grand AG, Viggers S, Baer LA, Solbeck S, Cotton BA, Matijevic N, Ostrowski SR, Stensballe J, Fox EE *et al*: **Impact of blood products on platelet function in patients with traumatic injuries: a translational study**. *J Surg Res* 2017, **214**:154-161.	3
40	Maurer-Spurej E, Chipperfield K: **Could microparticles be the universal quality indicator for platelet viability and function?** *J Blood Transfus* 2016, **2016**:6140239.	5
41	Johnson JL, Moore EE, Kashuk JL, Banerjee A, Cothren CC, Biffl WL, Sauaia A: **Effect of blood products transfusion on the development of postinjury multiple organ failure**. *Arch Surg* 2010, **145**(10):973-977.	3
42	Howard BM, Kornblith LZ, Hendrickson CM, Redick BJ, Conroy AS, Nelson MF, Callcut RA, Calfee CS, Cohen MJ: **Differences in degree, differences in kind: Characterizing lung injury in trauma**. *J Trauma Acute Care Surg* 2015, **78**(4):735-741.	3
43	Kasotakis G, Starr N, Nelson E, Sarkar B, Burke PA, Remick DG, Tompkins RG, Inflammation, Host Response to Injury I: **Platelet transfusion increases risk for acute respiratory distress syndrome in non-massively transfused blunt trauma patients**. *Eur J Trauma Emerg Surg* 2018.	3
44	Engele LJ, Straat M, van Rooijen IHM, de Vooght KMK, Cremer OL, Schultz MJ, Bos LDJ, Juffermans NP, Consortium M: **Transfusion of platelets, but not of red blood cells, is independently associated with nosocomial infections in the critically ill**. *Ann Intensive Care* 2016, **6**(1):67.	3

**R29 Platelets**		
**Reference**	**Citation**	**LoE**
45	Arbaeen AF, Schubert P, Serrano K, Carter CJ, Culibrk B, Devine DV: **Pathogen inactivation treatment of plasma and platelet concentrates and their predicted functionality in massive transfusion protocols**. *Transfusion* 2017, **57**(5):1208-1217.	3
46	Hess JR, Pagano MB, Barbeau JD, Johannson PI: **Will pathogen reduction of blood components harm more people than it helps in developed countries?** *Transfusion* 2016, **56**(5):1236-1241.	5
47	Inaba K, Branco BC, Rhee P, Blackbourne LH, Holcomb JB, Spinella PC, Shulman I, Nelson J, Demetriades D: **Impact of the duration of platelet storage in critically ill trauma patients**. *J Trauma* 2011, **71**(6):1766-1773; discussion 1773-1764.	3
48	Eker I, Yilmaz S, Cetinkaya RA, Unlu A, Pekel A, Acikel C, Yilmaz S, Gursel O, Avci IY: **Is one-size-fits-all strategy adequate for platelet storage?** *Transfus Apher Sci* 2016, **55**(3):323-328.	3
49	Meissner A, Schlenke P: **Massive bleeding and massive transfusion**. *Transfus Med Hemother* 2012, **39**(2):73-84.	5

R30 Calcium		
**Reference**	**Citation**	**LoE**
1	Lier H, Krep H, Schroeder S, Stuber F: **Preconditions of hemostasis in trauma: a review. The influence of acidosis, hypocalcemia, anemia, and hypothermia on functional hemostasis in trauma**. *J Trauma* 2008, **65**(4):951-960.	5
2	Perkins JG, Cap AP, Weiss BM, Reid TJ, Bolan CD: **Massive transfusion and nonsurgical hemostatic agents**. *Crit Care Med* 2008, **36**(7 Suppl):S325-339.	5
3	Magnotti LJ, Bradburn EH, Webb DL, Berry SD, Fischer PE, Zarzaur BL, Schroeppel TJ, Fabian TC, Croce MA: **Admission ionized calcium levels predict the need for multiple transfusions: a prospective study of 591 critically ill trauma patients**. *J Trauma* 2011, **70**(2):391-395; discussion 395-397.	3
4	Ho KM, Leonard AD: **Concentration-dependent effect of hypocalcaemia on mortality of patients with critical bleeding requiring massive transfusion: a cohort study**. *Anaesth Intensive Care* 2011, **39**(1):46-54.	3
5	Giancarelli A, Birrer KL, Alban RF, Hobbs BP, Liu-DeRyke X: **Hypocalcemia in trauma patients receiving massive transfusion**. *J Surg Res* 2016, **202**(1):182-187.	3
6	Lehmann M, Wallbank AM, Dennis KA, Wufsus AR, Davis KM, Rana K, Neeves KB: **On-chip recalcification of citrated whole blood using a microfluidic herringbone mixer**. *Biomicrofluidics* 2015, **9**(6):064106.	5

R31 Recombinant activated coagulation factor VII		
**Reference**	**Citation**	**LoE**
1	Hoffman M: **A cell-based model of coagulation and the role of factor VIIa**. *Blood Rev* 2003, **17 Suppl 1**:S1-5.	5
2	Hoffman M, Monroe DM, 3rd: **A cell-based model of hemostasis**. *Thromb Haemost* 2001, **85**(6):958-965.	5
3	DeLoughery TG: **Coagulation defects in trauma patients: etiology, recognition, and therapy**. *Crit Care Clin* 2004, **20**(1):13-24.	5
4	Luna GK, Maier RV, Pavlin EG, Anardi D, Copass MK, Oreskovich MR: **Incidence and effect of hypothermia in seriously injured patients**. *J Trauma* 1987, **27**(9):1014-1018.	3
5	Watts DD, Trask A, Soeken K, Perdue P, Dols S, Kaufmann C: **Hypothermic coagulopathy in trauma: effect of varying levels of hypothermia on enzyme speed, platelet function, and fibrinolytic activity**. *J Trauma* 1998, **44**(5):846-854.	3
6	Schochl H, Voelckel W, Maegele M, Kirchmair L, Schlimp CJ: **Endogenous thrombin potential following hemostatic therapy with 4-factor prothrombin complex concentrate: a 7-day observational study of trauma patients**. *Crit Care* 2014, **18**(4):R147.	3
7	Knudson MM, Cohen MJ, Reidy R, Jaeger S, Bacchetti P, Jin C, Wade CE, Holcomb JB: **Trauma, transfusions, and use of recombinant factor VIIa: A multicenter case registry report of 380 patients from the Western Trauma Association**. *J Am Coll Surg* 2011, **212**(1):87-95.	3
8	Mitra B, Cameron PA, Parr MJ, Phillips L: **Recombinant factor VIIa in trauma patients with the 'triad of death'**. *Injury* 2012, **43**(9):1409-1414.	3
9	Zatta A, McQuilten Z, Kandane-Rathnayake R, Isbister J, Dunkley S, McNeil J, Cameron P, Phillips L: **The Australian and New Zealand Haemostasis Registry: ten years of data on off-licence use of recombinant activated factor VII**. *Blood Transfus* 2015, **13**(1):86-99.	3
10	Vivien B, Langeron O, Morell E, Devilliers C, Carli PA, Coriat P, Riou B: **Early hypocalcemia in severe trauma**. *Crit Care Med* 2005, **33**(9):1946-1952.	4
11	James MF, Roche AM: **Dose-response relationship between plasma ionized calcium concentration and thrombelastography**. *J Cardiothorac Vasc Anesth* 2004, **18**(5):581-586.	5
12	Payen JF, Berthet M, Genty C, Declety P, Garrigue-Huet D, Morel N, Bouzat P, Riou B, Bosson JL, Novoseven Trauma i: **Reduced mortality by meeting guideline criteria before using recombinant activated factor VII in severe trauma patients with massive bleeding**. *Br J Anaesth* 2016, **117**(4):470-476.	3
13	Dutton RP, McCunn M, Hyder M, D'Angelo M, O'Connor J, Hess JR, Scalea TM: **Factor VIIa for correction of traumatic coagulopathy**. *J Trauma* 2004, **57**(4):709-718; discussion 718-709.	4
14	Harrison TD, Laskosky J, Jazaeri O, Pasquale MD, Cipolle M: **"Low-dose" recombinant activated factor VII results in less blood and blood product use in traumatic hemorrhage**. *J Trauma* 2005, **59**(1):150-154.	3
15	Martinowitz U, Kenet G, Segal E, Luboshitz J, Lubetsky A, Ingerslev J, Lynn M: **Recombinant activated factor VII for adjunctive hemorrhage control in trauma**. *J Trauma* 2001, **51**(3):431-438; discussion 438-439.	4
16	Boffard KD, Riou B, Warren B, Choong PI, Rizoli S, Rossaint R, Axelsen M, Kluger Y: **Recombinant factor VIIa as adjunctive therapy for bleeding control in severely injured trauma patients: two parallel randomized, placebo-controlled, double-blind clinical trials**. *J Trauma* 2005, **59**(1):8-15; discussion 15-18.	2
17	McQuay N, Jr., Cipolla J, Franges EZ, Thompson GE: **The use of recombinant activated factor VIIa in coagulopathic traumatic brain injuries requiring emergent craniotomy: is it beneficial?** *J Neurosurg* 2009, **111**(4):666-671.	4
18	Morse BC, Dente CJ, Hodgman EI, Shaz BH, Nicholas JM, Wyrzykowski AD, Salomone JP, Vercruysse GA, Rozycki GS, Feliciano DV: **The effects of protocolized use of recombinant factor VIIa within a massive**	4

R31 Recombinant activated coagulation factor VII		
**Reference**	**Citation**	**LoE**
	**transfusion protocol in a civilian level I trauma center**. *Am Surg* 2011, **77**(8):1043-1049.	
19	Nascimento B, Lin Y, Callum J, Reis M, Pinto R, Rizoli S: **Recombinant factor VIIa is associated with an improved 24-hour survival without an improvement in inpatient survival in massively transfused civilian trauma patients**. *Clinics (Sao Paulo)* 2011, **66**(1):101-106.	3
20	Hauser CJ, Boffard K, Dutton R, Bernard GR, Croce MA, Holcomb JB, Leppaniemi A, Parr M, Vincent JL, Tortella BJ *et al*: **Results of the CONTROL trial: efficacy and safety of recombinant activated Factor VII in the management of refractory traumatic hemorrhage**. *J Trauma* 2010, **69**(3):489-500.	2
21	Simpson E, Lin Y, Stanworth S, Birchall J, Doree C, Hyde C: **Recombinant factor VIIa for the prevention and treatment of bleeding in patients without haemophilia**. *Cochrane Database Syst Rev* 2012(3):CD005011.	1
22	Levi M, Levy JH, Andersen HF, Truloff D: **Safety of recombinant activated factor VII in randomized clinical trials**. *N Engl J Med* 2010, **363**(19):1791-1800.	1
23	O'Connell KA, Wood JJ, Wise RP, Lozier JN, Braun MM: **Thromboembolic adverse events after use of recombinant human coagulation factor VIIa**. *JAMA* 2006, **295**(3):293-298.	2
24	European Medicines Agency: NovoSeven® (rFVIIa; eptacog alfa) Summary of Product Characteristics. http://wwwemaeuropaeu/ 2017:Version updated 16/01/2017.	N/A
25	Dutton RP, Parr M, Tortella BJ, Champion HR, Bernard GR, Boffard K, Bouillon B, Croce MA, Dimsits J, Holcomb JB *et al*: **Recombinant activated factor VII safety in trauma patients: results from the CONTROL trial**. *J Trauma* 2011, **71**(1):12-19.	2
26	Bucklin MH, Acquisto NM, Nelson C: **The effects of recombinant activated factor VII dose on the incidence of thromboembolic events in patients with coagulopathic bleeding**. *Thromb Res* 2014, **133**(5):768-771.	3
27	DeLoughery EP, Lenfesty B, DeLoughery TG: **A retrospective case control study of recombinant factor VIIa in patients with intracranial haemorrhage caused by trauma**. *Br J Haematol* 2011, **152**(5):667-669.	4
28	DeLoughery EP, Lenfesty B, DeLoughery TG: **The use of recombinant factor VIIa in warfarin patients with traumatic brain injury: a retrospective case-control study**. *Blood Coagul Fibrinolysis* 2013, **24**(3):317-320.	4
29	Perel P, Roberts I, Shakur H, Thinkhamrop B, Phuenpathom N, Yutthakasemsunt S: **Haemostatic drugs for traumatic brain injury**. *Cochrane Database Syst Rev* 2010(1):CD007877.	1

R32 Reversal antithrombotics - not applicable - no references cited

R33 Reversal of vitamin K-dependent oral anticoagulants		
**Reference**	**Citation**	**LoE**
1	Levi M, Eerenberg E, Kamphuisen PW: **Bleeding risk and reversal strategies for old and new anticoagulants and antiplatelet agents**. *Journal of Thrombosis and Haemostasis* 2011, **9**(9):1705-1712.	1
2	Brekelmans MPA, van Ginkel K, Daams JG, Hutten BA, Middeldorp S, Coppens M: **Benefits and harms of 4-factor prothrombin complex concentrate for reversal of vitamin K antagonist associated bleeding: a systematic review and meta-analysis**. *Journal of Thrombosis and Thrombolysis* 2017, **44**(1):118-129.	1
3	Goldstein JN, Refaai MA, Milling TJ, Jr., Lewis B, Goldberg-Alberts R, Hug BA, Sarode R: **Four-factor prothrombin complex concentrate versus plasma for rapid vitamin K antagonist reversal in patients needing urgent surgical or invasive interventions: a phase 3b, open-label, non-inferiority, randomised trial**. *Lancet* 2015, **385**(9982):2077-2087.	2
4	Dowlatshahi D, Butcher KS, Asdaghi N, Nahirniak S, Bernbaum ML, Giulivi A, Wasserman JK, Poon MC, Coutts SB, Canadian PCCRI: **Poor prognosis in warfarin-associated intracranial hemorrhage despite anticoagulation reversal**. *Stroke* 2012, **43**(7):1812-1817.	4
5	Edavettal M, Rogers A, Rogers F, Horst M, Leng W: **Prothrombin complex concentrate accelerates international normalized ratio reversal and diminishes the extension of intracranial hemorrhage in geriatric trauma patients**. *Am Surg* 2014, **80**(4):372-376.	4
6	Fang MC, Go AS, Chang Y, Hylek EM, Henault LE, Jensvold NG, Singer DE: **Death and disability from warfarin-associated intracranial and extracranial hemorrhages**. *Am J Med* 2007, **120**(8):700-705.	3
7	Kuramatsu JB, Gerner ST, Schellinger PD, Glahn J, Endres M, Sobesky J, Flechsenhar J, Neugebauer H, Juttler E, Grau A *et al*: **Anticoagulant reversal, blood pressure levels, and anticoagulant resumption in patients with anticoagulation-related intracerebral hemorrhage**. *JAMA* 2015, **313**(8):824-836.	3
8	Steiner T, Poli S, Griebe M, Husing J, Hajda J, Freiberger A, Bendszus M, Bosel J, Christensen H, Dohmen C *et al*: **Fresh frozen plasma versus prothrombin complex concentrate in patients with intracranial haemorrhage related to vitamin K antagonists (INCH): a randomised trial**. *Lancet Neurol* 2016, **15**(6):566-573.	2
9	Hunt BJ, Levi M: **Urgent reversal of vitamin K antagonists**. *BMJ* 2018, **360**:j5424.	1
10	Dentali F, Ageno W, Crowther M: **Treatment of coumarin-associated coagulopathy: a systematic review and proposed treatment algorithms**. *J Thromb Haemost* 2006, **4**(9):1853-1863.	1
11	Dezee KJ, Shimeall WT, Douglas KM, Shumway NM, O'Malley P G: **Treatment of excessive anticoagulation with phytonadione (vitamin K): a meta-analysis**. *Arch Intern Med* 2006, **166**(4):391-397.	1
12	Britt RB, Brown JN: **Characterizing the Severe Reactions of Parenteral Vitamin K1**. *Clin Appl Thromb Hemost* 2018, **24**(1):5-12.	5
13	Mangram A, Oguntodu OF, Dzandu JK, Hollingworth AK, Hall S, Cung C, Rodriguez J, Yusupov I, Barletta JF: **Is there a difference in efficacy, safety, and cost-effectiveness between 3-factor and 4-factor prothrombin complex concentrates among trauma patients on oral anticoagulants?** *J Crit Care* 2016, **33**:252-256.	4

**R34-35 Direct oral anticoagulants: direct thrombin inhibitors; direct thrombin inhibitors**		
**Reference**	**Citation**	**LoE**
1	Levy JH, Key NS, Azran MS: **Novel oral anticoagulants: implications in the perioperative setting**. *Anesthesiology* 2010, **113**(3):726-745.	5
2	Dezman ZD, Comer AC, Smith GS, Narayan M, Hess JR, Hirshon JM: **The Severity of Bleeding and Mortality in Trauma Patients Taking Dabigatran**. *J Emerg Med* 2016, **51**(3):238-245.	3
3	Franklin NA, Ali A, Hurley RK, Mir HR, Beltran MJ: **The Outcomes of Early Surgical Intervention in Geriatric Proximal Femur Fractures among Patients Receiving Direct Oral Anticoagulation**. *J Orthop Trauma* 2018.	3
4	Kobayashi L, Barmparas G, Bosarge P, Brown CV, Bukur M, Carrick MM, Catalano RD, Holly-Nicolas J, Inaba K, Kaminski S *et al*: **Novel oral anticoagulants and trauma: The results of a prospective American Association for the Surgery of Trauma Multi-Institutional Trial**. *J Trauma Acute Care Surg* 2017, **82**(5):827-835.	3
5	Li Q, Dai B, Xu J, Yao Y, Song K, Zhang H, Chen D, Jiang Q: **Can patients with femoral neck fracture benefit from preoperative thromboprophylaxis?: A prospective randomized controlled trial**. *Medicine (Baltimore)* 2017, **96**(29):e7604.	2
6	Myers SP, Dadashzadeh ER, Cheung J, Alarcon L, Kutcher M, Brown JB, Neal MD: **Management of anticoagulation with rivaroxaban in trauma and acute care surgery: Complications and reversal strategies as compared to warfarin therapy**. *J Trauma Acute Care Surg* 2017, **82**(3):542-549.	3
7	Pozzessere A, Grotts J, Kaminski S: **Dabigatran Use Does Not Increase Intracranial Hemorrhage in Traumatic Geriatric Falls When Compared with Warfarin**. *The American surgeon* 2015, **81**(10):1039-1042.	3
8	Uccella L, Zoia C, Bongetta D, Gaetani P, Martig F, Candrian C, Rosso R: **Are Antiplatelet and Anticoagulants Drugs A Risk Factor for Bleeding in Mild Traumatic Brain Injury?** *World Neurosurg* 2018, **110**:e339-e345.	4
9	Asmis LM, Alberio L, Angelillo-Scherrer A, Korte W, Mendez A, Reber G, Seifert B, Stricker H, Tsakiris DA, Wuillemin WA: **Rivaroxaban: Quantification by anti-FXa assay and influence on coagulation tests: a study in 9 Swiss laboratories**. *Thromb Res* 2012, **129**(4):492-498.	5
10	Bliden KP, Chaudhary R, Mohammed N, Muresan AA, Lopez-Espina CG, Cohen E, Raviv G, Doubleday M, Zaman F, Mathew B *et al*: **Determination of non-Vitamin K oral anticoagulant (NOAC) effects using a new-generation thrombelastography TEG 6s system**. *J Thromb Thrombolysis* 2017, **43**(4):437-445.	5
11	Fontana P, Alberio L, Angelillo-Scherrer A, Asmis LM, Korte W, Mendez A, Schmid P, Stricker H, Studt JD, Tsakiris DA *et al*: **Impact of rivaroxaban on point-of-care assays**. *Thrombosis research* 2017, **153**:65-70.	5
12	Seyve L, Richarme C, Polack B, Marlu R: **Impact of four direct oral anticoagulants on rotational thromboelastometry (ROTEM)**. *International journal of laboratory hematology* 2018, **40**(1):84-93.	5
13	Solbeck S, Ostrowski SR, Stensballe J, Johansson PI: **Thrombelastography detects dabigatran at therapeutic concentrations in vitro to the same extent as gold-standard tests**. *International journal of cardiology* 2016, **208**:14-18.	5
14	Herzog E, Kaspereit F, Krege W, Mueller-Cohrs J, Doerr B, Niebl P, Dickneite G: **Correlation of Coagulation Markers and 4F-PCC-Mediated Reversal of Rivaroxaban in a Rabbit Model of Acute Bleeding**. *Thromb Res* 2015, **135**(3):554-560.	5
15	Pernod G, Albaladejo P, Godier A, Samama CM, Susen S, Gruel Y, Blais N, Fontana P, Cohen A, Llau JV *et al*: **Management of major bleeding complications and emergency surgery in patients on long-term treatment with direct oral anticoagulants, thrombin or factor-Xa inhibitors: proposals of the working group on perioperative**	5

R34-35 Direct oral anticoagulants: direct thrombin inhibitors; direct thrombin inhibitors		
**Reference**	**Citation**	**LoE**
	**haemostasis (GIHP) - March 2013**. *Archives of cardiovascular diseases* 2013, **106**(6-7):382-393.	
16	Sorensen B, Spahn DR, Innerhofer P, Spannagl M, Rossaint R: **Clinical review: Prothrombin complex concentrates--evaluation of safety and thrombogenicity**. *Crit Care* 2011, **15**(1):201.	5
17	Siegal DM, Curnutte JT, Connolly SJ, Lu G, Conley PB, Wiens BL, Mathur VS, Castillo J, Bronson MD, Leeds JM *et al*: **Andexanet Alfa for the Reversal of Factor Xa Inhibitor Activity**. *N Engl J Med* 2015, **373**(25):2413-2424.	1
18	Godier A, Miclot A, Le Bonniec B, Durand M, Fischer AM, Emmerich J, Marchand-Leroux C, Lecompte T, Samama CM: **Evaluation of prothrombin complex concentrate and recombinant activated factor VII to reverse rivaroxaban in a rabbit model**. *Anesthesiology* 2012, **116**(1):94-102.	5
19	Glund S, Stangier J, Schmohl M, Gansser D, Norris S, van Ryn J, Lang B, Ramael S, Moschetti V, Gruenenfelder F *et al*: **Safety, tolerability, and efficacy of idarucizumab for the reversal of the anticoagulant effect of dabigatran in healthy male volunteers: a randomised, placebo-controlled, double-blind phase 1 trial**. *Lancet* 2015.	2
20	Pollack CV, Jr., Reilly PA, Eikelboom J, Glund S, Verhamme P, Bernstein RA, Dubiel R, Huisman MV, Hylek EM, Kamphuisen PW *et al*: **Idarucizumab for dabigatran reversal**. *N Engl J Med* 2015, **373**(6):511-520.	1
21	Hegemann I, Ganter C, Widmer CC, Becker M, Müller D, Spahn DR: **Ongoing redistribution of dabigatran necessitates repetitive application of idarucizumab**. *Br J Anaesthesia* 2018:UPDATE WHEN AVAILABLE IN PUBMED.	5
22	Eikelboom JW, Quinlan DJ, van Ryn J, Weitz JI: **Idarucizumab: The Antidote for Reversal of Dabigatran**. *Circulation* 2015, **132**(25):2412-2422.	5

R36 Antiplatelet agents		
**Reference**	**Citation**	**LoE**
1	Conti A, Renzi N, Molesti D, Bianchi S, Bogazzi I, Bongini G, Pepe G, Frosini F, Bertini A, Santini M: **Short and long-term mortality of patients presenting with bleeding events to the Emergency Department**. *Am J Emerg Med* 2017, **35**(12):1867-1872.	3
2	Ott MM, Eriksson E, Vanderkolk W, Christianson D, Davis A, Scholten D: **Antiplatelet and anticoagulation therapies do not increase mortality in the absence of traumatic brain injury**. *J Trauma* 2010, **68**(3):560-563.	3
3	Ohmori T, Kitamura T, Onishi H, Ishihara J, Nojima T, Yamamoto K: **Effect of pre-injury anticoagulant and antiplatelet agents on blood loss in elderly patients with severe trauma**. *Acute Med Surg* 2016, **3**(2):114-119.	3
4	Ohmori T, Kitamura T, Ishihara J, Onishi H, Nojima T, Yamamoto K, Tamura R, Muranishi K, Matsumoto T, Tokioka T: **Early predictors for massive transfusion in older adult severe trauma patients**. *Injury* 2017, **48**(5):1006-1012.	3
5	Kudo D, Kushimoto S, Shiraishi A, Ogura H, Hagiwara A, Saitoh D, Investigators JO: **The impact of preinjury antithrombotic medication on hemostatic interventions in trauma patients**. *Am J Emerg Med* 2017, **35**(1):62-65.	4
6	Battle C, Hutchings H, Bouamra O, Evans PA: **The effect of pre-injury anti-platelet therapy on the development of complications in isolated blunt chest wall trauma: a retrospective study**. *PLoS One* 2014, **9**(3):e91284.	3
7	Coleman J, Baldawi M, Heidt D: **The effect anticoagulation status on geriatric fall trauma patients**. *Am J Surg* 2016, **212**(6):1237-1242.	3
8	Kragh AM, Walden M, Apelqvist A, Wagner P, Atroshi I: **Bleeding and first-year mortality following hip fracture surgery and preoperative use of low-dose acetylsalicylic acid: an observational cohort study**. *BMC Musculoskelet Disord* 2011, **12**:254.	3
9	Christy JM, Stawicki SP, Jarvis AM, Evans DC, Gerlach AT, Lindsey DE, Rhoades P, Whitmill ML, Steinberg SM, Phieffer LS *et al*: **The impact of antiplatelet therapy on pelvic fracture outcomes**. *J Emerg Trauma Shock* 2011, **4**(1):64-69.	3
10	Doleman B, Moppett IK: **Is early hip fracture surgery safe for patients on clopidogrel? Systematic review, meta-analysis and meta-regression**. *Injury* 2015, **46**(6):954-962.	2
11	Soo CG, Della Torre PK, Yolland TJ, Shatwell MA: **Clopidogrel and hip fractures, is it safe? A systematic review and meta-analysis**. *BMC Musculoskelet Disord* 2016, **17**:136.	2
12	Mattesi L, Noailles T, Rosencher N, Rouvillain JL: **Discontinuation of Plavix((R)) (clopidogrel) for hip fracture surgery. A systematic review of the literature**. *Orthop Traumatol Surg Res* 2016, **102**(8):1097-1101.	5
13	Pailleret C, Ait Hamou Z, Rosencher N, Samama CM, Eyraud V, Chilot F, Baillard C: **A retrospective comparison between delayed and early hip fracture surgery in patients taking clopidogrel: same total bleeding but different timing of blood transfusion**. *Int Orthop* 2017, **41**(9):1839-1844.	3
14	Purushothaman B, Webb M, Weusten A, Bonczek S, Ramaskandhan J, Nanu A: **Decision making on timing of surgery for hip fracture patients on clopidogrel**. *Ann R Coll Surg Engl* 2016, **98**(2):91-95.	4
15	Akaoka Y, Yamazaki H, Kodaira H, Kato H: **Risk factors for the effect of anticoagulant and antiplatelet agents on perioperative blood loss following proximal femoral fractures**. *Medicine (Baltimore)* 2016, **95**(27):e4120.	3
16	Zhang J, Chen X, Wang J, Liu Z, Wang X, Ren J, Sun T: **Poor prognosis after surgery for intertrochanteric fracture in elderly patients with clopidogrel treatment: A cohort study**. *Medicine (Baltimore)* 2017, **96**(39):e8169.	4
17	van den Brand CL, Tolido T, Rambach AH, Hunink MG, Patka P, Jellema K: **Systematic review and meta-analysis: is pre-injury antiplatelet therapy**	2

R36 Antiplatelet agents		
**Reference**	**Citation**	**LoE**
	**associated with traumatic intracranial hemorrhage?** *J Neurotrauma* 2017, **34**(1):1-7.	
18	Nishijima DK, Gaona SD, Waechter T, Maloney R, Bair T, Blitz A, Elms AR, Farrales RD, Howard C, Montoya J *et al*: **Out-of-hospital triage of older adults with head injury: a retrospective study of the effect of adding "anticoagulation or antiplatelet medication use" as a criterion**. *Ann Emerg Med* 2017, **70**(2):127-138 e126.	3
19	Gaist D, Garcia Rodriguez LA, Hellfritzsch M, Poulsen FR, Halle B, Hallas J, Pottegard A: **Association of antithrombotic drug use with subdural hematoma risk**. *JAMA* 2017, **317**(8):836-846.	3
20	Bhattacharya B, Maung A, Schuster K, Davis KA: **The older they are the harder they fall: Injury patterns and outcomes by age after ground level falls**. *Injury* 2016, **47**(9):1955-1959.	3
21	Nishijima DK, Gaona SD, Waechter T, Maloney R, Blitz A, Elms AR, Farrales RD, Montoya J, Bair T, Howard C *et al*: **The incidence of traumatic intracranial hemorrhage in head-injured older adults transported by EMS with and without anticoagulant or antiplatelet use**. *J Neurotrauma* 2017.	3
22	Chenoweth JA, Gaona SD, Faul M, Holmes JF, Nishijima DK, Sacramento County Prehospital Research C: **Incidence of delayed intracranial hemorrhage in older patients after blunt head trauma**. *JAMA Surg* 2018.	3
23	Ganetsky M, Lopez G, Coreanu T, Novack V, Horng S, Shapiro NI, Bauer KA: **Risk of Intracranial Hemorrhage in Ground-level Fall With Antiplatelet or Anticoagulant Agents**. *Acad Emerg Med* 2017, **24**(10):1258-1266.	3
24	Wurtz M, Schmidt M, Grove EL, Horvath-Puho E, Henderson VW, Christiansen CF, Sorensen HT: **Preadmission use of platelet inhibitors and short-term stroke mortality: A population-based cohort study**. *Eur Heart J Cardiovasc Pharmacother* 2018.	3
25	Li X, Sun Z, Zhao W, Zhang J, Chen J, Li Y, Ye Y, Zhao J, Yang X, Xiang Y *et al*: **Effect of acetylsalicylic acid usage and platelet transfusion on postoperative hemorrhage and activities of daily living in patients with acute intracerebral hemorrhage**. *J Neurosurg* 2013, **118**(1):94-103.	1
26	Stein M, Misselwitz B, Hamann GF, Kolodziej M, Reinges MH, Uhl E: **In-hospital mortality after pre-treatment with antiplatelet agents or oral anticoagulants and hematoma evacuation of intracerebral hematomas**. *J Clin Neurosci* 2016, **26**:42-45.	3
27	Camps-Renom P, Alejaldre-Monforte A, Delgado-Mederos R, Martinez-Domeno A, Prats-Sanchez L, Pascual-Goni E, Marti-Fabregas J: **Does prior antiplatelet therapy influence hematoma volume and hematoma growth following intracerebral hemorrhage? Results from a prospective study and a meta-analysis**. *Eur J Neurol* 2017, **24**(2):302-308.	2
28	van Ginneken V, Engel P, Fiebach JB, Audebert HJ, Nolte CH, Rocco A: **Prior antiplatelet therapy is not associated with larger hematoma volume or hematoma growth in intracerebral hemorrhage**. *Neurol Sci* 2018, **39**(4):745-748.	3
29	Yu HH, Pan C, Tang YX, Liu N, Zhang P, Hu Y, Zhang Y, Wu Q, Deng H, Li GG *et al*: **Effects of Prior Antiplatelet Therapy on the Prognosis of Primary Intracerebral Hemorrhage: A Meta-analysis**. *Chin Med J (Engl)* 2017, **130**(24):2969-2977.	2
30	Khan NI, Siddiqui FM, Goldstein JN, Cox M, Xian Y, Matsouaka RA, Heidenreich PA, Peterson ED, Bhatt DL, Fonarow GC *et al*: **Association Between Previous Use of Antiplatelet Therapy and Intracerebral Hemorrhage Outcomes**. *Stroke* 2017, **48**(7):1810-1817.	3
31	Batchelor JS, Grayson A: **A meta-analysis to determine the effect of preinjury antiplatelet agents on mortality in patients with blunt head trauma**. *Br J Neurosurg* 2013, **27**(1):12-18.	2

**R36 Antiplatelet agents**		
**Reference**	**Citation**	**LoE**
32	Fabbri A, Servadei F, Marchesini G, Bronzoni C, Montesi D, Arietta L, Societa Italiana di Medicina d'Emergenza Urgenza Study G: **Antiplatelet therapy and the outcome of subjects with intracranial injury: the Italian SIMEU study**. *Crit Care* 2013, **17**(2):R53.	3
33	Joseph B, Sadoun M, Aziz H, Tang A, Wynne JL, Pandit V, Kulvatunyou N, O'Keeffe T, Friese RS, Rhee P: **Repeat head computed tomography in anticoagulated traumatic brain injury patients: still warranted**. *Am Surg* 2014, **80**(1):43-47.	3
34	Farsi D, Karimi P, Mofidi M, Mahshidfar B, Rezai M, Hafezimoghadam P, Abbasi S: **Effects of pre-injury anti-platelet agents on short-term outcome of patients with mild traumatic brain injury: A cohort study**. *Bull Emerg Trauma* 2017, **5**(2):110-115.	3
35	Grandhi R, Harrison G, Voronovich Z, Bauer J, Chen SH, Nicholas D, Alarcon LH, Okonkwo DO: **Preinjury warfarin, but not antiplatelet medications, increases mortality in elderly traumatic brain injury patients**. *J Trauma Acute Care Surg* 2015, **78**(3):614-621.	3
36	Narum S, Brors O, Stokland O, Kringen MK: **Mortality among head trauma patients taking preinjury antithrombotic agents: a retrospective cohort analysis from a Level 1 trauma centre**. *BMC Emerg Med* 2016, **16**(1):29.	3
37	Okazaki T, Hifumi T, Kawakita K, Nakashima R, Matsumoto A, Shishido H, Ogawa D, Okauchi M, Shindo A, Kawanishi M *et al*: **Association between comorbidities, nutritional status, and anticlotting drugs and neurologic outcomes in geriatric patients with traumatic brain injury**. *World Neurosurg* 2016, **93**:336-340.	3
38	Lewis PR, Dunne CE, Wallace JD, Brill JB, Calvo RY, Badiee J, Sise MJ, Bansal V, Sise CB, Shackford SR: **Routine neurosurgical consultation is not necessary in mild blunt traumatic brain injury**. *J Trauma Acute Care Surg* 2017, **82**(4):776-780.	3
39	Han H, Koh EJ, Choi H, Kim BC, Yang SY, Cho KT: **The effect of preoperative antiplatelet therapy on hemorrhagic complications after decompressive craniectomy in patients with traumatic brain injury**. *Korean J Neurotrauma* 2016, **12**(2):61-66.	3
40	Bauman ZM, Ruggero JM, Squindo S, McEachin C, Jaskot M, Ngo W, Barnes S, Lopez PP: **Repeat head CT? Not necessary for patients with a negative initial head CT on anticoagulation or antiplatelet therapy suffering low-altitude falls**. *Am Surg* 2017, **83**(5):429-435.	3
41	Nishijima DK, Shahlaie K, Sarkar K, Rudisill N, Holmes JF: **Risk of unfavorable long-term outcome in older adults with traumatic intracranial hemorrhage and anticoagulant or antiplatelet use**. *Am J Emerg Med* 2013, **31**(8):1244-1247.	3
42	Joseph B, Pandit V, Aziz H, Kulvatunyou N, Hashmi A, Tang A, O'Keeffe T, Wynne J, Vercruysse G, Friese RS *et al*: **Clinical outcomes in traumatic brain injury patients on preinjury clopidogrel: a prospective analysis**. *J Trauma Acute Care Surg* 2014, **76**(3):817-820.	3
43	Bachelani AM, Bautz JT, Sperry JL, Corcos A, Zenati M, Billiar TR, Peitzman AB, Marshall GT: **Assessment of platelet transfusion for reversal of aspirin after traumatic brain injury**. *Surgery* 2011, **150**(4):836-843.	3
44	Joseph B, Aziz H, Pandit V, Kulvatunyou N, O'Keeffe T, Tang A, Wynne J, Hashmi A, Vercruysse G, Friese RS *et al*: **Low-dose aspirin therapy is not a reason for repeating head computed tomographic scans in traumatic brain injury: a prospective study**. *J Surg Res* 2014, **186**(1):287-291.	3
45	Lee AT, Gagnidze A, Pan SR, Sookplung P, Nair B, Newman SF, Ben-Ari A, Zaky A, Cain K, Vavilala MS *et al*: **Preoperative low-dose aspirin exposure and outcomes after emergency neurosurgery for traumatic intracranial hemorrhage in elderly patients**. *Anesth Analg* 2017, **125**(2):514-520.	3
46	Marincowitz C, Lecky FE, Townend W, Borakati A, Fabbri A, Sheldon TA: **The Risk of Deterioration in GCS13-15 Patients with Traumatic Brain**	2

**R36 Antiplatelet agents**		
**Reference**	**Citation**	**LoE**
	**Injury Identified by Computed Tomography Imaging: A Systematic Review and Meta-Analysis**. *J Neurotrauma* 2018, **35**(5):703-718.	
47	Yuan Q, Sun YR, Wu X, Yu J, Li ZQ, Du ZY, Wu XH, Zhou LF, Hu J: **Coagulopathy in traumatic brain injury and its correlation with progressive hemorrhagic injury: A systematic review and meta-analysis**. *J Neurotrauma* 2016, **33**(14):1279-1291.	2
48	Carnevale JA, Segar DJ, Powers AY, Shah M, Doberstein C, Drapcho B, Morrison JF, Williams JR, Collins S, Monteiro K *et al*: **Blossoming contusions: identifying factors contributing to the expansion of traumatic intracerebral hemorrhage**. *J Neurosurg* 2018:1-12.	3
49	Joseph B, Pandit V, Meyer D, Butvidas L, Kulvatunyou N, Khalil M, Tang A, Zangbar B, O'Keeffe T, Gries L *et al*: **The significance of platelet count in traumatic brain injury patients on antiplatelet therapy**. *J Trauma Acute Care Surg* 2014, **77**(3):417-421.	3
50	Vilahur G, Choi BG, Zafar MU, Viles-Gonzalez JF, Vorchheimer DA, Fuster V, Badimon JJ: **Normalization of platelet reactivity in clopidogrel-treated subjects**. *J Thromb Haemost* 2007, **5**(1):82-90.	3
51	Hansson EC, Shams Hakimi C, Astrom-Olsson K, Hesse C, Wallen H, Dellborg M, Albertsson P, Jeppsson A: **Effects of ex vivo platelet supplementation on platelet aggregability in blood samples from patients treated with acetylsalicylic acid, clopidogrel, or ticagrelor**. *Br J Anaesth* 2014, **112**(3):570-575.	3
52	Martin AC, Berndt C, Calmette L, Philip I, Decouture B, Gaussem P, Gouin-Thibault I, Samama CM, Bachelot-Loza C, Godier A: **The effectiveness of platelet supplementation for the reversal of ticagrelor-induced inhibition of platelet aggregation: An in-vitro study**. *Eur J Anaesthesiol* 2016, **33**(5):361-367.	3
53	O'Connor SA, Amour J, Mercadier A, Martin R, Kerneis M, Abtan J, Brugier D, Silvain J, Barthelemy O, Leprince P *et al*: **Efficacy of ex vivo autologous and in vivo platelet transfusion in the reversal of P2Y12 inhibition by clopidogrel, prasugrel, and ticagrelor: the APTITUDE study**. *Circ Cardiovasc Interv* 2015, **8**(11):e002786.	3
54	Zafar MU, Santos-Gallego C, Vorchheimer DA, Viles-Gonzalez JF, Elmariah S, Giannarelli C, Sartori S, Small DS, Jakubowski JA, Fuster V *et al*: **Platelet function normalization after a prasugrel loading-dose: time-dependent effect of platelet supplementation**. *J Thromb Haemost* 2013, **11**(1):100-106.	4
55	Zafar MU, Smith DA, Baber U, Sartori S, Chen K, Lam DW, Linares-Koloffon CA, Rey-Mendoza J, Jimenez Britez G, Escolar G *et al*: **Impact of timing on the functional recovery achieved with platelet supplementation after treatment with Ticagrelor**. *Circ Cardiovasc Interv* 2017, **10**(8).	3
56	Teng R, Carlson GF, Nylander S, Andersson TL: **Effects of autologous platelet transfusion on platelet inhibition in ticagrelor-treated and clopidogrel-treated subjects**. *J Thromb Haemost* 2016, **14**(12):2342-2352.	2
57	Briggs A, Gates JD, Kaufman RM, Calahan C, Gormley WB, Havens JM: **Platelet dysfunction and platelet transfusion in traumatic brain injury**. *J Surg Res* 2015, **193**(2):802-806.	3
58	Lindblad C, Thelin EP, Nekludov M, Frostell A, Nelson DW, Svensson M, Bellander BM: **Assessment of platelet function in traumatic brain injury-a retrospective observational study in the neuro-critical care setting**. *Front Neurol* 2018, **9**:15.	3
59	Joseph B, Pandit V, Sadoun M, Larkins CG, Kulvatunyou N, Tang A, Mino M, Friese RS, Rhee P: **A prospective evaluation of platelet function in patients on antiplatelet therapy with traumatic intracranial hemorrhage**. *J Trauma Acute Care Surg* 2013, **75**(6):990-994.	3
60	Choi PA, Parry PV, Bauer JS, Zusman BE, Panczykowski DM, Puccio AM, Okonkwo DO: **Use of Aspirin and P2Y12 Response Assays in Detecting Reversal of Platelet Inhibition With Platelet Transfusion in Patients With**	3

R36 Antiplatelet agents		
**Reference**	**Citation**	**LoE**
	**Traumatic Brain Injury on Antiplatelet Therapy**. *Neurosurgery* 2017, **80**(1):98-104.	
61	Holzmacher JL, Reynolds C, Patel M, Maluso P, Holland S, Gamsky N, Moore H, Acquista E, Carrick M, Amdur R *et al*: **Platelet transfusion does not improve outcomes in patients with brain injury on antiplatelet therapy**. *Brain Inj* 2018, **32**(3):325-330.	4
62	Taylor G, Osinski D, Thevenin A, Devys JM: **Is platelet transfusion efficient to restore platelet reactivity in patients who are responders to aspirin and/or clopidogrel before emergency surgery?** *J Trauma Acute Care Surg* 2013, **74**(5):1367-1369.	3
63	Bertling A, Fender AC, Schungel L, Rumpf M, Mergemeier K, Geissler G, Sibrowski W, Kelsch R, Waltenberger J, Jakubowski JA *et al*: **Reversibility of platelet P2Y12 inhibition by platelet supplementation: ex vivo and in vitro comparisons of prasugrel, clopidogrel and ticagrelor**. *J Thromb Haemost* 2018.	4
64	Baschin M, Selleng S, Hummel A, Diedrich S, Schroeder HW, Kohlmann T, Westphal A, Greinacher A, Thiele T: **Preoperative platelet transfusions to reverse antiplatelet therapy for urgent non-cardiac surgery: an observational cohort study**. *J Thromb Haemost* 2018, **16**(4):709-717.	3
65	Nishijima DK, Zehtabchi S, Berrong J, Legome E: **Utility of platelet transfusion in adult patients with traumatic intracranial hemorrhage and preinjury antiplatelet use: a systematic review**. *J Trauma Acute Care Surg* 2012, **72**(6):1658-1663.	2
66	Batchelor JS, Grayson A: **A meta-analysis to determine the effect on survival of platelet transfusions in patients with either spontaneous or traumatic antiplatelet medication-associated intracranial haemorrhage**. *BMJ Open* 2012, **2**(2):e000588.	2
67	Leong LB, David TK: **Is platelet transfusion effective in patients taking antiplatelet agents who suffer an intracranial hemorrhage?** *J Emerg Med* 2015, **49**(4):561-572.	2
68	Pandya U, Malik A, Messina M, Albeiruti AR, Spalding C: **Reversal of antiplatelet therapy in traumatic intracranial hemorrhage: Does timing matter?** *J Clin Neurosci* 2018, **50**:88-92.	3
69	Baharoglu MI, Cordonnier C, Salman RA, de Gans K, Koopman MM, Brand A, Majoie CB, Beenen LF, Marquering HA, Vermeulen M *et al*: **Platelet transfusion versus standard care after acute stroke due to spontaneous cerebral haemorrhage associated with antiplatelet therapy (PATCH): a randomised, open-label, phase 3 trial**. *Lancet* 2016, **387**(10038):2605-2613.	1
70	Arnone GD, Kumar P, Wonais MC, Esfahani DR, Campbell-Lee SA, Charbel FT, Amin-Hanjani S, Alaraj A, Seicean A, Mehta AI: **Impact of platelet transfusion on intracerebral hemorrhage in patients on antiplatelet therapy-an analysis based on intracerebral hemorrhage score**. *World Neurosurg* 2018, **111**:e895-e904.	3
71	Guerrero WR, Gonzales NR, Sekar P, Kawano-Castillo J, Moomaw CJ, Worrall BB, Langefeld CD, Martini SR, Flaherty ML, Sheth KN *et al*: **Variability in the use of platelet transfusion in patients with intracerebral hemorrhage: Observations from the ethnic/racial variations of intracerebral hemorrhage study**. *J Stroke Cerebrovasc Dis* 2017, **26**(9):1974-1980.	3
72	Baschin M, Selleng S, Zeden JP, Westphal A, Kohlmann T, Schroeder HW, Greinacher A, Thiele T: **Platelet transfusion to reverse antiplatelet therapy before decompressive surgery in patients with intracranial haemorrhage**. *Vox Sang* 2017, **112**(6):535-541.	3
73	Frontera JA, Lewin JJ, 3rd, Rabinstein AA, Aisiku IP, Alexandrov AW, Cook AM, del Zoppo GJ, Kumar MA, Peerschke EI, Stiefel MF *et al*: **Guideline for reversal of antithrombotics in intracranial hemorrhage: a statement for**	5

R36 Antiplatelet agents		
Reference	Citation	LoE
	**healthcare professionals from the neurocritical care society and society of critical care medicine**. *Neurocrit Care* 2016, **24**(1):6-46.	
74	Altman R, Scazziota A, M DELH, Gonzalez C: **Recombinant factor VIIa reverses the inhibitory effect of aspirin or aspirin plus clopidogrel on in vitro thrombin generation**. *J Thromb Haemost* 2006, **4**(9):2022-2027.	3
75	Skolnick BE, Shenouda M, Khutoryansky NM, Pusateri AE, Gabriel D, Carr ME: **Reversal of clopidogrel-induced bleeding with rFVIIa in healthy subjects: a randomized, placebo-controlled, double-blind, exploratory study**. *Anesth Analg* 2011, **113**(4):703-710.	2
76	Weber CF, Görlinger K, Byhahn C, Moritz A, Hanke AA, Zacharowski K, Meininger D: **Tranexamic acid partially improves platelet function in patients treated with dual antiplatelet therapy**. *Eur J Anaesthesiol* 2011, **28**(1):57-62.	3
77	Van Aelbrouck C, Jorquera-Vasquez S, Beukinga I, Pradier O, Ickx B, Barvais L, Van Obbergh L, Faraoni D: **Tranexamic acid decreases the magnitude of platelet dysfunction in aspirin-free patients undergoing cardiac surgery with cardiopulmonary bypass: a pilot study**. *Blood Coagul Fibrinolysis* 2016, **27**(8):855-861.	3
78	Leissinger C, Carcao M, Gill JC, Journeycake J, Singleton T, Valentino L: **Desmopressin (DDAVP) in the management of patients with congenital bleeding disorders**. *Haemophilia* 2014, **20**(2):158-167.	5
79	Orsini S, Noris P, Bury L, Heller PG, Santoro C, Kadir RA, Butta NC, Falcinelli E, Cid AR, Fabris F *et al*: **Bleeding risk of surgery and its prevention in patients with inherited platelet disorders**. *Haematologica* 2017, **102**(7):1192-1203.	5
80	Crescenzi G, Landoni G, Biondi-Zoccai G, Pappalardo F, Nuzzi M, Bignami E, Fochi O, Maj G, Calabro MG, Ranucci M *et al*: **Desmopressin reduces transfusion needs after surgery: a meta-analysis of randomized clinical trials**. *Anesthesiology* 2008, **109**(6):1063-1076.	2
81	Desborough MJ, Oakland KA, Landoni G, Crivellari M, Doree C, Estcourt LJ, Stanworth SJ: **Desmopressin for treatment of platelet dysfunction and reversal of antiplatelet agents: a systematic review and meta-analysis of randomized controlled trials**. *J Thromb Haemost* 2017, **15**(2):263-272.	1
82	Desborough MJ, Oakland K, Brierley C, Bennett S, Doree C, Trivella M, Hopewell S, Stanworth SJ, Estcourt LJ: **Desmopressin use for minimising perioperative blood transfusion**. *Cochrane Database Syst Rev* 2017, **7**:CD001884.	1
83	Steinlechner B, Zeidler P, Base E, Birkenberg B, Ankersmit HJ, Spannagl M, Quehenberger P, Hiesmayr M, Jilma B: **Patients with severe aortic valve stenosis and impaired platelet function benefit from preoperative desmopressin infusion**. *Ann Thorac Surg* 2011, **91**(5):1420-1426.	3
84	Weber CF, Dietrich W, Spannagl M, Hofstetter C, Jambor C: **A point-of-care assessment of the effects of desmopressin on impaired platelet function using multiple electrode whole-blood aggregometry in patients after cardiac surgery**. *Anesth Analg* 2010, **110**(3):702-707.	3
85	Pearson K, Jensen H, Kander T, Schott U: **Desmopressin in vitro effects on platelet function, monitored with Multiplate, ROTEM and Sonoclot**. *Scand J Clin Lab Invest* 2016, **76**(4):282-290.	4
86	Tsui PY, Cheung CW, Lee Y, Leung SW, Ng KF: **The effectiveness of low-dose desmopressin in improving hypothermia-induced impairment of primary haemostasis under influence of aspirin - a randomized controlled trial**. *BMC Anesthesiol* 2015, **15**:80.	3
87	Leithauser B, Zielske D, Seyfert UT, Jung F: **Effects of desmopressin on platelet membrane glycoproteins and platelet aggregation in volunteers on clopidogrel**. *Clin Hemorheol Microcirc* 2008, **39**(1-4):293-302.	3
88	Teng R, Mitchell PD, Butler K: **The effect of desmopressin on bleeding time and platelet aggregation in healthy volunteers administered ticagrelor**. *J Clin Pharm Ther* 2014, **39**(2):186-191.	3

**R36 Antiplatelet agents**		
**Reference**	**Citation**	**LoE**
89	Bonhomme F, Lecompte T, Samama CM, Godier A, Fontana P: **Evaluation of recombinant factor VIIa, tranexamic acid and desmopressin to reduce prasugrel-related bleeding: A randomised, placebo-controlled study in a rabbit model**. *Eur J Anaesthesiol* 2018, **35**(3):208-214.	5
90	Kapapa T, Rohrer S, Struve S, Petscher M, Konig R, Wirtz CR, Woischneck D: **Desmopressin acetate in intracranial haemorrhage**. *Neurol Res Int* 2014, **2014**:298767.	4
91	Naidech AM, Maas MB, Levasseur-Franklin KE, Liotta EM, Guth JC, Berman M, Rosenow JM, Lindholm PF, Bendok BR, Prabhakaran S *et al*: **Desmopressin improves platelet activity in acute intracerebral hemorrhage**. *Stroke* 2014, **45**(8):2451-2453.	4
92	Kim DY, O'Leary M, Nguyen A, Kaji A, Bricker S, Neville A, Bongard F, Putnam B, Plurad D: **The effect of platelet and desmopressin administration on early radiographic progression of traumatic intracranial hemorrhagea**. *J Neurotrauma* 2015, **32**(22):1815-1821.	3
93	Ng KF, Cheung CW, Lee Y, Leung SW: **Low-dose desmopressin improves hypothermia-induced impairment of primary haemostasis in healthy volunteers**. *Anaesthesia* 2011, **66**(11):999-1005.	4
94	Hanke AA, Dellweg C, Kienbaum P, Weber CF, Gorlinger K, Rahe-Meyer N: **Effects of desmopressin on platelet function under conditions of hypothermia and acidosis: an in vitro study using multiple electrode aggregometry***. *Anaesthesia* 2010, **65**(7):688-691.	4
95	Singleton T, Kruse-Jarres R, Leissinger C: **Emergency department care for patients with hemophilia and von Willebrand disease**. *J Emerg Med* 2010, **39**(2):158-165.	5

R37 Thromboprophylaxis		
**Reference**	**Citation**	**LoE**
1	Geerts WH, Code KI, Jay RM, Chen E, Szalai JP: **A prospective study of venous thromboembolism after major trauma**. *N Engl J Med* 1994, **331**(24):1601-1606.	2
2	Velmahos GC, Kern J, Chan L, Oder D, Murray JA, Shekelle P: **Prevention of venous thromboembolism after injury**. *Evid Rep Technol Assess (Summ)* 2000(22):1-3.	1
3	CLOTS (Clots in legs or stockings after stroke) Trials Collaboration, Dennis M, Sandercock P, Reid J, Graham C, Forbes J, Murray G: **Effectiveness of intermittent pneumatic compression in reduction of risk of deep vein thrombosis in patients who have had a stroke (CLOTS 3): a multicentre randomised controlled trial**. *The Lancet* 2013, **382**(9891):516-524.	2
4	Kakkos SK, Caprini JA, Geroulakos G, Nicolaides AN, Stansby G, Reddy DJ, Ntouvas I: **Combined intermittent pneumatic leg compression and pharmacological prophylaxis for prevention of venous thromboembolism**. *Cochrane Database Syst Rev* 2016, **9**:CD005258.	1
5	Alhazzani W, Lim W, Jaeschke RZ, Murad MH, Cade J, Cook DJ: **Heparin thromboprophylaxis in medical-surgical critically ill patients: a systematic review and meta-analysis of randomized trials**. *Crit Care Med* 2013, **41**(9):2088-2098.	1
6	Lim W, Meade M, Lauzier F, Zarychanski R, Mehta S, Lamontagne F, Dodek P, McIntyre L, Hall R, Heels-Ansdell D *et al*: **Failure of anticoagulant thromboprophylaxis: risk factors in medical-surgical critically ill patients***. *Crit Care Med* 2015, **43**(2):401-410.	2
7	Lubenow N, Hinz P, Thomaschewski S, Lietz T, Vogler M, Ladwig A, Junger M, Nauck M, Schellong S, Wander K *et al*: **The severity of trauma determines the immune response to PF4/heparin and the frequency of heparin-induced thrombocytopenia**. *Blood* 2010, **115**(9):1797-1803.	2
8	Connelly CR, Van PY, Hart KD, Louis SG, Fair KA, Erickson AS, Rick EA, Simeon EC, Bulger EM, Arbabi S *et al*: **Thrombelastography-based dosing of enoxaparin for thromboprophylaxis in trauma and surgical patients: A randomized clinical trial**. *JAMA Surg* 2016, **151**(10):e162069.	2
9	Ko A, Harada MY, Barmparas G, Chung K, Mason R, Yim DA, Dhillon N, Margulies DR, Gewertz BL, Ley EJ: **Association between enoxaparin dosage adjusted by anti-factor Xa trough level and clinically evident venous thromboembolism after trauma**. *JAMA Surg* 2016, **151**(11):1006-1013.	3
10	Singer GA, Riggi G, Karcutskie CA, Vaghaiwalla TM, Lieberman HM, Ginzburg E, Namias N, Lineen EB: **Anti-Xa-guided enoxaparin thromboprophylaxis reduces rate of deep venous thromboembolism in high-risk trauma patients**. *J Trauma Acute Care Surg* 2016, **81**(6):1101-1108.	3
11	Hoffmeyer P, Simmen H, Jakob M, Sommer C, Platz A, Ilchmann T, Grossen E, Ryf C, Christofilopoulos P, Schueler M *et al*: **Rivaroxaban for thromboprophylaxis after nonelective orthopedic trauma surgery in Switzerland**. *Orthopedics* 2017, **40**(2):109-116.	3
12	Shen X, Dutcher SK, Palmer J, Liu X, Kiptanui Z, Khokhar B, Al-Jawadi MH, Zhu Y, Zuckerman IH: **A systematic review of the benefits and risks of anticoagulation following traumatic brain injury**. *J Head Trauma Rehabil* 2015, **30**(4):E29-37.	1
13	Ho KM, Chavan S, Pilcher D: **Omission of early thromboprophylaxis and mortality in critically ill patients: a multicenter registry study**. *Chest* 2011, **140**(6):1436-1446.	3
14	Jacobsen AF, Skjeldestad FE, Sandset PM: **Ante- and postnatal risk factors of venous thrombosis: a hospital-based case-control study**. *J Thromb Haemost* 2008, **6**(6):905-912.	3

R38-39 Guideline implemtation and outcome assessment		
**Reference**	**Citation**	**LoE**
1	Borns J, Ersch J, Dobrovoljac M, Staubli G, Brotschi B: **Video recordings to analyze preventable management errors in pediatric resuscitation bay**. *Pediatr Emerg Care* 2018.	5
2	Brolliar SM, Moore M, Thompson HJ, Whiteside LK, Mink RB, Wainwright MS, Groner JI, Bell MJ, Giza CC, Zatzick DF *et al*: **A qualitative study exploring factors associated with provider adherence to severe pediatric traumatic brain injury guidelines**. *J Neurotrauma* 2016, **33**(16):1554-1560.	3
3	Cnossen MC, Scholten AC, Lingsma HF, Synnot A, Tavender E, Gantner D, Lecky F, Steyerberg EW, Polinder S: **Adherence to guidelines in adult patients with traumatic brain injury: A living systematic review**. *J Neurotrauma* 2016.	3
4	Lee JC, Rittenhouse K, Bupp K, Gross B, Rogers A, Rogers FB, Horst M, Estrella L, Thurmond J: **An analysis of Brain Trauma Foundation traumatic brain injury guideline compliance and patient outcome**. *Injury* 2015, **46**(5):854-858.	2
5	Riney LC, Frey TM, Fain ET, Duma EM, Bennett BL, Murtagh Kurowski E: **Standardizing the evaluation of nonaccidental trauma in a large pediatric emergency department**. *Pediatrics* 2018, **141**(1).	3
6	Godier A, Bacus M, Kipnis E, Tavernier B, Guidat A, Rauch A, Drumez E, Susen S, Garrigue-Huet D: **Compliance with evidence-based clinical management guidelines in bleeding trauma patients**. *Br J Anaesth* 2016, **117**(5):592-600.	2
7	Stein P, Kaserer A, Sprengel K, Wanner GA, Seifert B, Theusinger OM, Spahn DR: **Change of transfusion and treatment paradigm in major trauma patients**. *Anaesthesia* 2017, **72**(11):1317-1326.	2
8	Maegele M, Lefering R, Wafaisade A, Theodorou P, Wutzler S, Fischer P, Bouillon B, Paffrath T, Trauma Registry of Deutsche Gesellschaft fur U: **Revalidation and update of the TASH-Score: a scoring system to predict the probability for massive transfusion as a surrogate for life-threatening haemorrhage after severe injury**. *Vox Sang* 2011, **100**(2):231-238.	5
9	Haynes AB, Weiser TG, Berry WR, Lipsitz SR, Breizat AH, Dellinger EP, Herbosa T, Joseph S, Kibatala PL, Lapitan MC *et al*: **A surgical safety checklist to reduce morbidity and mortality in a global population**. *N Engl J Med* 2009, **360**(5):491-499.	1
10	Gillespie BM, Chaboyer W, Thalib L, John M, Fairweather N, Slater K: **Effect of using a safety checklist on patient complications after surgery: a systematic review and meta-analysis**. *Anesthesiology* 2014, **120**(6):1380-1389.	1
11	Damiani E, Donati A, Serafini G, Rinaldi L, Adrario E, Pelaia P, Busani S, Girardis M: **Effect of performance improvement programs on compliance with sepsis bundles and mortality: a systematic review and meta-analysis of observational studies**. *PLoS One* 2015, **10**(5):e0125827.	1
12	Levy MM, Rhodes A, Phillips GS, Townsend SR, Schorr CA, Beale R, Osborn T, Lemeshow S, Chiche JD, Artigas A *et al*: **Surviving Sepsis Campaign: association between performance metrics and outcomes in a 7.5-year study**. *Intensive Care Med* 2014, **40**(11):1623-1633.	2
