# Supplementary material for: The European guideline on management of major bleeding and coagulopathy following trauma: fifth edition
Source: Crit Care. 2019 Mar 27;23:98. doi: 10.1186/s13054-019-2347-3 (PMC6436241; doi:10.1186/s13054-019-2347-3)
Supplement: Supplementary file 3 — Summary of recommendations. (PDF 103 kb) [file 13054_2019_2347_MOESM3_ESM.pdf]

**The European guideline on management of major bleeding and coagulopathy following trauma: Fifth edition**

Spahn DR, Bouillon B, Cerny V, Duranteau J, Filipescu D, Hunt BJ, Komadina R, Maegele M, Nardi G, Riddez L, Samama C-M, Vincent J-L, Rossaint R

## SUMMARY OF RECOMMENDATIONS

| # | Title | Recommendation | Grade |
|---|---|---|---|
| **I. Initial resuscitation and prevention of further bleeding** | | | |
| **R1** | **Minimal elapsed time** | We recommend that severely injured patients be transported directly to an appropriate trauma facility. | **1B** |
| | | We recommend that the time elapsed between injury and bleeding control be minimised. | **1A** |
| **R2** | **Local bleeding management** | We recommend local compression to limit life-threatening bleeding. | **1A** |
| | | We recommend adjunct tourniquet use to stop life-threatening bleeding from open extremity injuries in the pre-surgical setting. | **1B** |
| | | We recommend the adjunct use of a pelvic binder to limit life-threatening bleeding in the presence of a suspected pelvic fracture in the pre-surgical setting. | **1B** |
| **R3** | **Ventilation** | We recommend the avoidance of hypoxaemia. | **1A** |
| | | We recommend normoventilation of trauma patients | **1B** |
| | | We suggest hyperventilation in the presence of signs of imminent cerebral herniation. | **2C** |
| **II. Diagnosis and monitoring of bleeding** | | | |
| **R4** | **Initial assessment** | We recommend that the physician clinically assess the extent of traumatic haemorrhage using a combination of patient physiology, anatomical injury pattern, mechanism of injury and the patient response to initial resuscitation. | **1C** |
| | | We suggest that the shock index (SI) be used to assess the degree of hypovolaemic shock. | **2C** |
| **R5** | **Immediate intervention** | We recommend that patients with an obvious bleeding source and those presenting with haemorrhagic shock in extremis and a suspected source of bleeding undergo an immediate bleeding control procedure. | **1C** |
| **R6** | **Further investigation** | We recommend that patients without a need for immediate bleeding control and an unidentified source of bleeding undergo immediate further investigation. | **1C** |
| **R7** | **Imaging** | We recommend the use of focused assessment with sonography in trauma (FAST) ultrasound for the detection of free fluid in patients with torso trauma. | **1C** |
| | | We recommend early imaging using contrast-enhanced whole-body CT (WBCT) for the detection and identification of type of injury and potential source of bleeding. | **1B** |
| **R8** | **Haemoglobin** | We recommend that a low initial Hb be considered an indicator for severe bleeding associated with coagulopathy. | **1B** |
| | | We recommend the use of repeated Hb measurements as a laboratory marker for bleeding, as an initial Hb value in the normal range may mask bleeding. | **1B** |
| **R9** | **Serum lactate and base deficit** | We recommend serum lactate and/or base deficit measurements as a sensitive test to estimate and monitor the extent of bleeding and shock. | **1B** |

| # | Title | Recommendation | Grade |
|---|---|---|---|
| **R10** | **Coagulation monitoring** | We recommend that routine practice include the early and repeated monitoring of haemostasis, using either a combined traditional laboratory determination [prothrombin time (PT), platelet counts and Clauss fibrinogen level] and/or point-of-care (POC) PT/international normalised ratio (INR) and/or a viscoelastic method (VEM). | **1C** |
| | | We recommend laboratory screening of patients treated or suspected of being treated with anticoagulant agents. | **1C** |
| **R11** | **Platelet function monitoring** | We suggest the use of POC platelet function devices as an adjunct to standard laboratory and/or POC coagulation monitoring in patients with suspected platelet dysfunction. | **2C** |
| **III. Tissue oxygenation, volume, fluids and temperature** | | | |
| **R12** | **Tissue oxygenation** | We recommend permissive hypotension with a target systolic blood pressure of 80-90 mmHg (mean arterial pressure 50-60 mmHg) until major bleeding has been stopped in the initial phase following trauma without brain injury. | **1C** |
| | | In patients with severe TBI (GCS≤8), we recommend that a mean arterial pressure ≥80 mmHg be maintained. | **1C** |
| **R13** | **Restricted volume replacement** | We recommend use of a restricted volume replacement strategy to achieve target blood pressure until bleeding can be controlled. | **1B** |
| **R14** | **Vasopressors and inotropic agents** | In the presence of life-threatening hypotension, we recommend administration of vasopressors in addition to fluids to maintain target arterial pressure. | **1C** |
| | | We recommend infusion of an inotropic agent in the presence of myocardial dysfunction. | **1C** |
| **R15** | **Type of fluid** | We recommend that fluid therapy using isotonic crystalloid solutions be initiated in the hypotensive bleeding trauma patient. | **1A** |
| | | We recommend the use of balanced electrolyte solutions and the avoidance of saline solutions. | **1B** |
| | | We recommend that hypotonic solutions such as Ringer's lactate be avoided in patients with severe head trauma. | **1B** |
| | | We recommend that the use of colloids be restricted due to the adverse effects on haemostasis. | **1C** |
| **R16** | **Erythrocytes** | We recommend a target Hb of 70 to 90 g/L. | **1C** |
| **R17** | **Temperature management** | In order to optimise coagulation, we recommend early application of measures to reduce heat loss and warm the hypothermic patient to achieve and maintain normothermia. | **1C** |
| **IV. Rapid control of bleeding** | | | |
| **R18** | **Damage control surgery** | We recommend that damage control surgery be employed in the severely injured patient presenting with deep haemorrhagic shock, signs of ongoing bleeding and coagulopathy. | **1B** |
| | | Other factors that should trigger a damage control approach are hypothermia, acidosis, inaccessible major anatomic injury, a need for time-consuming procedures or concomitant major injury outside the abdomen. | **1C** |
| | | We recommend primary definitive surgical management in the haemodynamically stable patient and in the absence of any of the factors above. | **1C** |

| # | Title | Recommendation | Grade |
|---|---|---|---|
| **R19** | **Pelvic ring closure and stabilisation** | We recommend that patients with pelvic ring disruption in haemorrhagic shock undergo immediate pelvic ring closure and stabilisation. | **1B** |
| **R20** | **Packing, embolisation and surgery** | We recommend that patients with ongoing haemodynamic instability, despite adequate pelvic ring stabilisation, receive early surgical bleeding control and/or pre-peritoneal packing and/or angiographic embolisation. | **1B** |
| | | We suggest that the use of aortic balloon occlusion be considered only under extreme circumstances in patients with pelvic fracture in order to gain time until appropriate bleeding control measures can be implemented. | **2C** |
| **R21** | **Local haemostatic measures** | We recommend the use of topical haemostatic agents in combination with other surgical measures or with packing for venous or moderate arterial bleeding associated with parenchymal injuries. | **1B** |
| **V. Initial management of bleeding & coagulopathy** | | | |
| **R22** | **Antifibrinolytic agents** | We recommend that TXA be administered to the trauma patient who is bleeding or at risk of significant haemorrhage as soon as possible and within 3 h after injury at a loading dose of 1 g infused over 10 min, followed by an i.v. infusion of 1 g over 8 h. | **1A** |
| | | We recommend that protocols for the management of bleeding patients consider administration of the first dose of TXA en route to the hospital. | **1C** |
| | | We recommend that the administration of TXA not await results from a viscoelastic assessment. | **1B** |
| **R23** | **Coagulation support** | We recommend that monitoring and measures to support coagulation be initiated immediately upon hospital admission. | **1B** |
| **R24** | **Intial coagulation resuscitation** | In the initial management of patients with expected massive haemorrhage, we recommend one of the two following strategies: | |
| | | • FFP or pathogen-inactivated FFP in a FFP:RBC ratio of at least 1:2 as needed. | **1C** |
| | | • Fibrinogen concentrate and RBC. | **1C** |
| **VI. Further goal-directed coagulation management** | | | |
| **R25** | **Goal-directed therapy** | We recommend that resuscitation measures be continued using a goal-directed strategy, guided by standard laboratory coagulation values and/or VEM. | **1B** |
| **R26** | **Fresh frozen plasma-based management** | If a FFP-based coagulation resuscitation strategy is used, we recommend that further use of FFP be guided by standard laboratory coagulation screening parameters (PT and/or APTT >1.5 times normal and/or viscoelastic evidence of a coagulation factor deficiency). | **1C** |
| | | We recommend that FFP transfusion be avoided in patients without major bleeding. | **1B** |
| | | We recommend that the use of FFP be avoided for the treatment of hypofibrinogenaemia. | **1C** |
| **R27** | | If a CFC-based strategy is used, we recommend treatment with factor concentrates based on standard laboratory coagulation parameters and/or viscoelastic evidence of a functional coagulation factor deficiency. | **1C** |

| # | Title | Recommendation | Grade |
|---|---|---|---|
| | **Coagulation factor concentrate-based management** | Provided that fibrinogen levels are normal, we suggest that PCC is administered to the bleeding patient based on evidence of delayed coagulation initiation using VEM. | **2C** |
| | | We suggest that monitoring of FXIII be included in coagulation support algorithms and that FXIII be supplemented in bleeding patients with a functional FXIII deficiency. | **2C** |
| **R28** | **Fibrinogen supplementation** | We recommend treatment with fibrinogen concentrate or cryoprecipitate if major bleeding is accompanied by hypofibrinogenaemia (viscoelastic signs of a functional fibrinogen deficit or a plasma Clauss fibrinogen level ≤1.5 g/L). | **1C** |
| | | We suggest an initial fibrinogen supplementation of 3-4 g. This is equivalent to 15-20 single donor units of cryoprecipitate or 3-4 g fibrinogen concentrate. Repeat doses should be guided by VEM and laboratory assessment of fibrinogen levels. | **2C** |
| **R29** | **Platelets** | We recommend that platelets be administered to maintain a platelet count above 50 × $10^9$/L. | **1C** |
| | | We suggest maintenance of a platelet count above 100 × $10^9$/L in patients with ongoing bleeding and/or TBI. | **2C** |
| | | If administered, we suggest an initial dose of four to eight single platelet units or one aphaeresis pack. | **2C** |
| **R30** | **Calcium** | We recommend that ionised calcium levels be monitored and maintained within the normal range during massive transfusion. | **1C** |
| | | We suggest the administration of calcium chloride to correct hypocalcaemia. | **2C** |
| **R31** | **Recombinant activated coagulation factor VII** | We do not recommend the use of recombinant activated coagulation factor VII (rFVIIa) as first-line treatment. | **1B** |
| | | We suggest that the off-label use of rFVIIa be considered only if major bleeding and traumatic coagulopathy persist despite all other attempts to control bleeding and best-practice use of conventional haemostatic measures. | **2C** |
| **VII. Reversal of antithrombotic agents** | | | |
| **R32** | **Antithrombotic agent reversal** | We recommend reversal of the effect of antithrombotic agents in patients with ongoing bleeding. | **1C** |
| **R33** | **Reversal of vitamin K-dependent oral anticoagulants** | In the bleeding trauma patient, we recommend the emergency reversal of vitamin K-dependent oral anticoagulants with the early use of both PCC and 5 mg i.v. phytomenadione (vitamin $K_1$). | **1A** |
| **R34** | **Direct oral anticoagulants – factor Xa inhibitors** | We suggest the measurement of plasma levels of oral direct anti-factor Xa agents such as apixaban, edoxaban or rivaroxaban in patients treated or suspected of being treated with one of these agents. | **2C** |
| | | We suggest that measurement of anti-Xa activity be calibrated for the specific agent. If measurement is not possible or available, we suggest that advice from an expert haematologist be sought. | **2C** |
| | | If bleeding is life-threatening, we suggest administration of TXA 15 mg/kg (or 1 g) intravenously and that the use of PCC (25-50 U/kg) be considered until specific antidotes are available. | **2C** |

| # | Title | Recommendation | Grade |
|---|---|---|---|
| **R35** | **Direct oral anticoagulants – direct thrombin inhibitors** | We suggest the measurement of dabigatran plasma levels using diluted thrombin time in patients treated or suspected of being treated with dabigatran. | **2C** |
| | | If measurement is not possible or available, we suggest measurement of the standard thrombin time to allow a qualitative estimation of the presence of dabigatran. | **2C** |
| | | If bleeding is life-threatening in those receiving dabigatran, we recommend treatment with idarucizumab (5 g intravenously) / suggest treatment with TXA 15 mg/kg (or 1 g) intravenously. | **1B / 2C** |
| **R36** | **Antiplatelet agents** | We suggest treatment with platelet concentrates if platelet dysfunction is documented in a patient with continued bleeding who has been treated with APA. | **2C** |
| | | We suggest administration of platelets in patients with ICH who have been treated with APA and will undergo surgery. | **2B** |
| | | We suggest that the administration of platelets in patients with ICH who have been treated with APA and will not undergo surgical intervention be avoided. | **2B** |
| | | We suggest that the administration of desmopressin (0.3 µg/kg) be considered in patients treated with platelet-inhibiting drugs or von Willebrand disease. | **2C** |
| **VIII. Thromboprophylaxis** | | | |
| **R37** | **Thromboprophylaxis** | We recommend early mechanical thromboprophylaxis with intermittent pneumatic compression (IPC) while the patient is immobile and has a bleeding risk. | **1C** |
| | | We recommend combined pharmacological and IPC thromboprophylaxis within 24 h after bleeding has been controlled and until the patient is mobile. | **1B** |
| | | We do not recommend the use of graduated compression stockings for thromboprophylaxis. | **1C** |
| | | We do not recommend the routine use of inferior vena cava filters as thromboprophylaxis. | **1C** |
| **IX. Guideline implementation and quality control** | | | |
| **R38** | **Guideline implementation** | We recommend the local implementation of evidence-based guidelines for management of the bleeding trauma patient. | **1B** |
| **R39** | **Assessment of bleeding control and outcome** | We recommend that local clinical quality and safety management systems include parameters to assess key measures of bleeding control and outcome. | **1B** |
